# An induced-fit model for asymmetric organocatalytic reactions: a case study of the activation of olefins *via* chiral Brønsted acid catalysts†

**DOI:** 10.1039/d2sc02274e

**Published:** 2022-07-04

**Authors:** Ingolf Harden, Frank Neese, Giovanni Bistoni

**Affiliations:** Max-Planck-Institut für Kohlenforschung Kaiser-Wilhelm Platz 1 45470 Mülheim an der Ruhr Germany; Department of Chemistry, Biology and Biotechnology, University of Perugia Via Elce di Sotto, 8 06123 Perugia Italy giovanni.bistoni@unipg.it

## Abstract

We elucidate the stereo-controlling factors of the asymmetric intramolecular hydroalkoxylation of terminal olefins catalyzed by bulky Brønsted acids [*Science***2018**, *359* (6383), 1501–1505] using high-level electronic structure methods. The catalyst–substrate interaction is described using a dispersion-driven induced-fit model, in which the conformational changes of the catalyst and of the substrate in the transition states are governed to a large extent by London dispersion forces. The distortion energy of the catalyst is dominated by the change in the intramolecular dispersion interactions, while intermolecular catalyst–substrate dispersion interactions are the major stabilizing contribution in the transition state. This model provides a new general framework in which to discuss the stereoselectivity of transformations catalyzed by such confined organocatalysts.

## Abstract 2

We elucidate the stereo-controlling factors of the asymmetric intramolecular hydroalkoxylation of terminal olefins catalyzed by bulky Brønsted acids [*Science***2018**, *359* (6383), 1501–1505] using high-level electronic structure methods.

## Graphical abstract

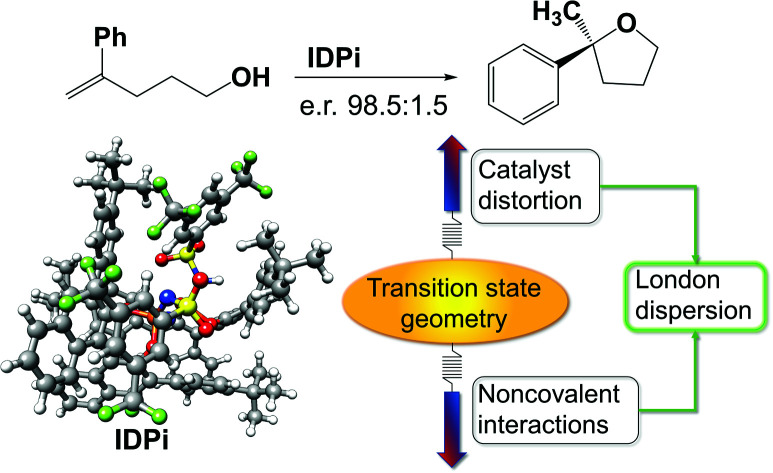

## Introduction

Catalysis is involved in the processing of most manufactured products and hence the design of new catalytic processes plays a central role in chemical research.^1^ The selectivity of a catalyst is one of the most important criteria in this context: selective reactions generate fewer undesired byproducts and are thus both environmentally sustainable and economical.

In biocatalysis, reactions occur inside structurally elaborate pockets within the enzyme active site, leading to high degrees of selectivity and activity.^2^ This stimulated chemists of all disciplines to develop synthetic hosts that emulate these natural biological pockets. As a prominent example, Yoshizawa *et al.*^3^ carried out Diels–Alder reactions of anthracene and phthalimide guests in confined aqueous organopalladium cage complexes, which led to unprecedented regioselectivity. Hastings *et al.* used the tetrahedral assembly K_12_Ga_4_L_6_ (L = 1,5-biscatecholamidenaphthalene) to catalyze Nazarov cyclizations within a confined space. This led to an increase of rate constants of six orders of magnitude,^4^ showing a catalytic activity that is also reminiscent of that of enzymes. Similarly, under heterogeneous conditions, reactions in confined zeolite spaces are also well-known for their high activity and selectivity.^5,6^ Notably, in the field of asymmetric organocatalysis, the List group developed a series of confined chiral Brønsted acids with enzyme-like activity and selectivity. These encompass imidodiphosphates (IDP),^7–10^ imino-imidodiphosphates (iIDP),^11^ and imidodiphosphorimidates (IDPi) Brønsted acids,^12–15^ differing in their acidity as well as in the shape of their active site. As a recent example, IDPi catalysts were used to catalyze asymmetric Mukaiyama aldolizations of silylenolethers with Benzaldehyde and it was suggested that the size of the catalyst pocket determines the benzaldehyde enantiofacial preference.^16^

Based on these findings, List and coworkers suggested that “confinement”, *i.e.*, the shaping of the active site, should be regarded as a unifying element in selective catalysis.^17^ But how does confinement induce selectivity, and how this information can be used in the design of new catalysts? In biocatalysis, a complex pattern of interactions within the active site forces the substrates into specific orientations, thus favoring and/or disfavoring specific reaction pathways. A similar situation is expected to occur in any kind of selective catalysis, and harnessing the power of such interactions is one of the key challenges in contemporary chemical research.^18^ These interactions can be either covalent or noncovalent, either attractive or repulsive, depending on the nature of the substrates and of the catalytic system.

A ubiquitous interaction energy component that is commonly associated with the concept of confinement is “steric repulsion”, *i.e.*, the short-range repulsion of atoms, molecules or functional groups. This interaction has often been advocated as the key factor controlling the stereoselectivity of a large number of chemical transformations.^19–23^ The key idea behind this assumption is that the introduction of bulky groups in a catalytic system can be used to block all the undesirable reaction pathways. However, the increase of catalytic activity that is often associated with confined reaction spaces is difficult to explain using steric repulsion alone,^24^ and in fact several recent works have emphasized the importance of attractive noncovalent interactions (NCIs) in this context.^25,26^

In contrast to steric repulsion, attractive NCIs are expected to influence the selectivity of a chemical process by lowering the energy barrier for specific reaction pathways, rather than by blocking those that are undesirable. Among the various types of NCIs, London dispersion (LD) has recently emerged for its ubiquitous importance in selective catalysis.^27–36^ This attractive interaction is always present between atoms, molecules or groups, irrespective of their nature. Its magnitude increases with the polarizability of the interacting species and decreases with their distance. Its fundamental role in stereoselective catalysis has been emphasized by the group of Schreiner on the Dakin–West reaction^32^ and the Corey–Bakshi–Shibata reduction.^33^ The group of Houk has demonstrated its importance for intramolecular Aldol reactions^34^ as well as in the dual transition metal catalysis of α-allylation reactions.^35^ In addition, Fürstner and coworkers used London dispersion as a key design element for confined Bismuth–Rhodium catalysts with improved activity and selectivity.^36^

Thus, steric repulsion and LD are two ubiquitous interaction energy components of opposite sign that increase in absolute terms with the internuclear distance, and hence are expected to play an especially important role in reactions occurring in confined spaces. Their interplay was recently investigated by our group on challenging stereoselective organocatalytic Diels–Alder reactions catalyzed by List's enzyme-like Brønsted acids,^37^ and it was found that both components must be considered for a thorough understanding of the stereo-controlling factors.

**Scheme 1:**
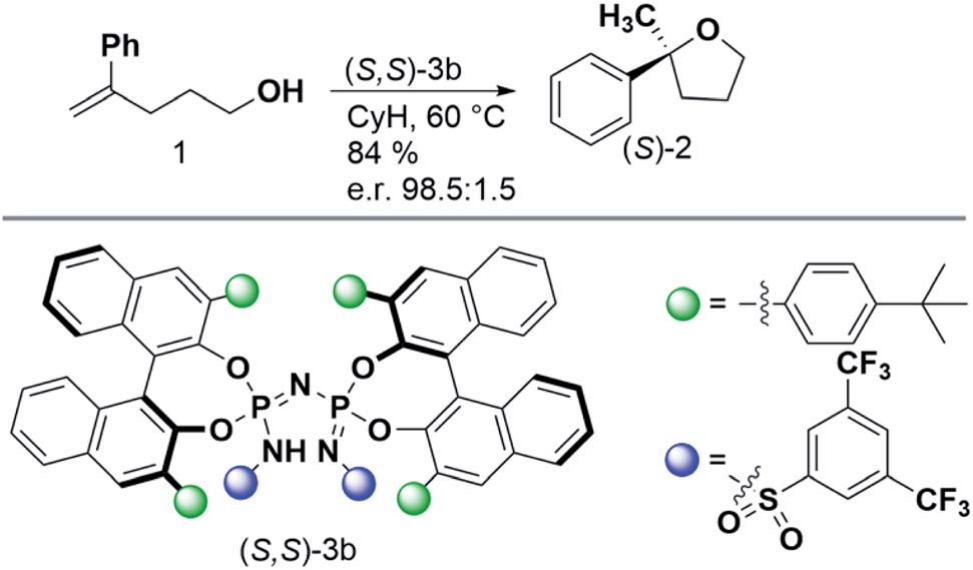
Reaction and experimental conditions considered in this work. Details can be found in ref. 38.

In this paper, we seek to reveal how increasing confinement quantitatively influences dispersion and steric repulsion, and how these energy components can in principle be harnessed to increase the selectivity of asymmetric transformations. As a case study, we consider the intramolecular hydroalkoxylation of unactivated alkenes published by the List group in 2018.^38^ The reaction conditions and the catalyst used are reported in Scheme 1. Remarkably enough, an in-depth analysis of the key stereo-controlling factors for this reaction is still missing.

## Theoretical aspects

### General considerations

Predicting the outcome of stereoselective reactions catalyzed by large and flexible catalysts would potentially require the calculation of a huge number of relative reaction rates with extremely high accuracy. Although several computational approaches have been suggested to this aim, none of them has so far established itself as the method of choice in this context.^18^ Among the most promising tools used in virtual screening for catalyst design, we mention CatVS^39^ (Catalyst Virtual Screening) AARON^40,41^ and ACE (Asymmetric Catalyst Evaluation).^42^ Alternatively, statistical and machine learning methods have also been introduced.^43^ In this work, we rely on a slightly modified version of the computational protocol developed in our group for the calculation of selectivities of asymmetric transformations,^37^ which combines advanced conformational sampling techniques, highly correlated wavefunction based methods and quantitative tools for the analysis of the key noncovalent interactions. Unless otherwise specified, all calculations were carried out with ORCA 4.2.1.^44^

### Computational protocol

**Scheme 2:**
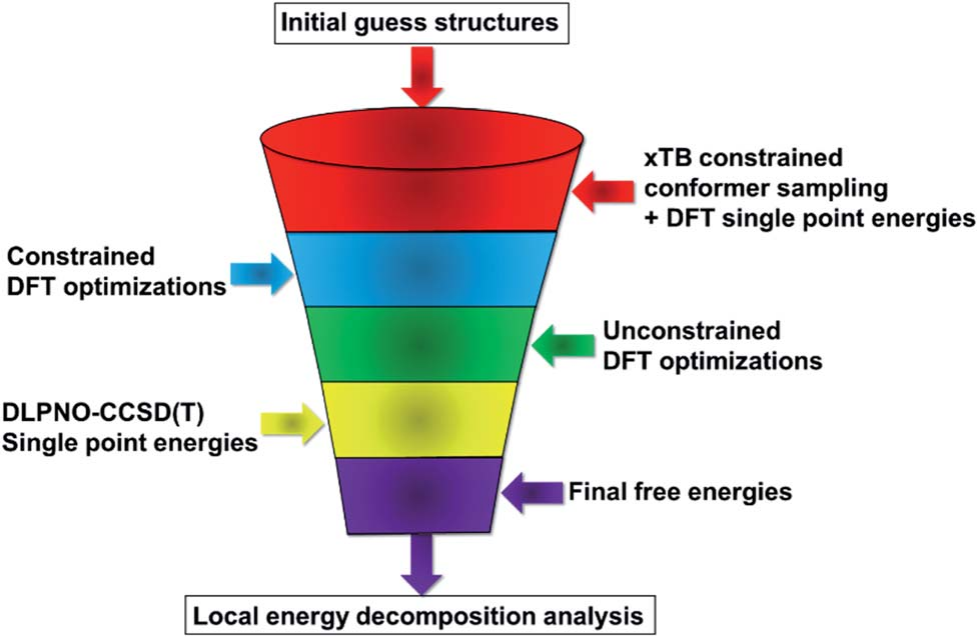
Computational protocol used in this work.

Our computational protocol is illustrated in Scheme 2.

As initial guess structures for the transition states leading to the major and minor enantiomers, we used those computed by List and coworkers in their original work.^38^ Importantly, the stereo-controlling factors of this important transformation were not discussed in this work, and no extensive conformational sampling procedure was carried out for the relevant transition states.

In the first step of our protocol, an initial conformational sampling at the transition states was carried out using the Conformer–Rotamer Ensemble Sampling Tool^45–47^ (CREST) at the xTB-GFN1 and xTB-GFN2 levels of theory, as implemented in the Extended Tight-Binding Program Package^48–50^ (xTB). Due to the large number of conformers obtained from various sampling runs, the protocol defined in ref. 37 was slightly changed to reduce the computational time. Instead of performing DFT geometry optimizations for the entire structural ensemble (which consists of 6485 structures), the conformers were initially sorted in energy by means of DFT single point calculations. The Perdew–Burke–Ernzerhof (PBE) functional^51^ was used in conjunction with the Ahlrichs double-zeta def2-SVP basis set.^52^ Grimme's dispersion correction D3 (ref. 53) was used together with Becke–Johnson damping.^54^ To speed up the calculation, the resolution of identity^55^ in the Split-RJ variant^56^ was used together with the appropriate Coulomb-fitting basis set.^57^ This computational protocol is denoted hereafter as PBE-D3/def2-SVP.

**Fig. 1:**
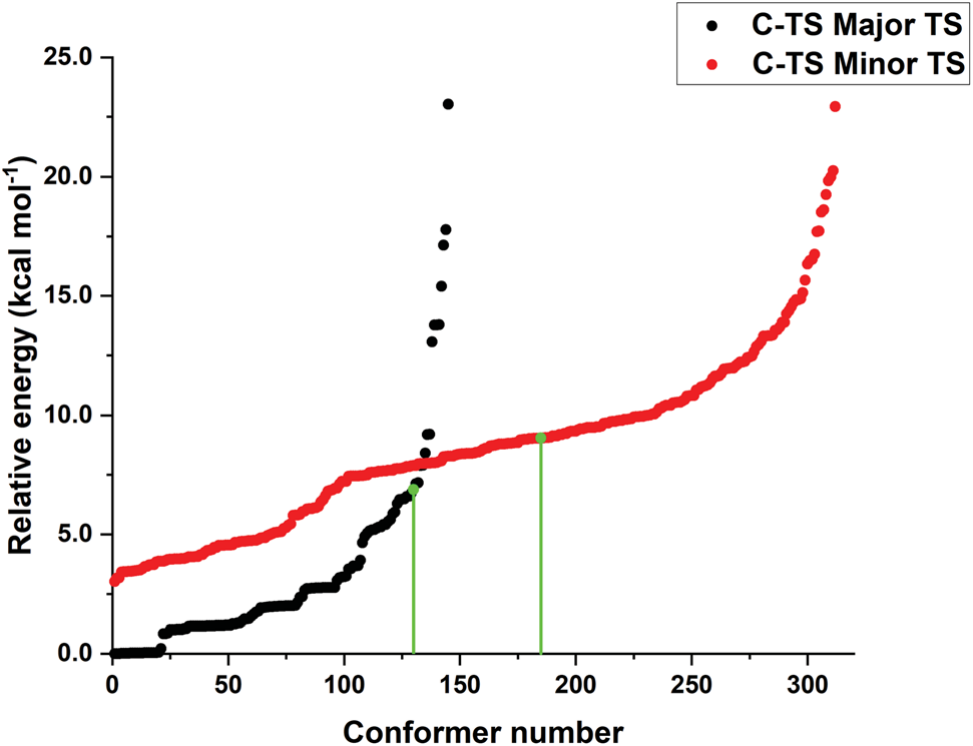
Relative energy obtained from constrained PBE-D3/def2-SVP geometry optimizations leading to the major (black) and minor (red) enantiomeric product for the energetically favored concerted mechanism “A”. The transition state energies of the conformers originally discussed in ref. 38 at the same level of theory are shown in green to emphasize the importance of the conformer sampling procedure.

The low energy candidate structures were included in the subsequent constrained geometry optimization step. In particular, a cutoff value of 4 kcal mol^−1^ was used for the relative electronic energies. In addition, to make the protocol more robust, higher-energy structures with significantly different structural features were also included. This step reduced the number of candidate transition state structures from 6485 to 655. Constrained geometry optimizations at the same level of theory were performed for all the 655 structures that survived the initial screening. The N–H bond distance was selected as only constraint. Tight DFT grids were used for the geometry optimizations (GRID6). The relative energies of the structures that lead to the desired products (459 conformers) are shown in Fig. 1.

Remarkably enough, this protocol identifies more than 100 transition state structures lower in energy than those originally discussed in ref. 38 at the same level of theory. Note that, as discussed in detail in ref. 37, the energy difference between the “constrained” and fully relaxed transition states is typically very small when meaningful constraints are defined.

The low-energy conformers with significantly different structural features were optimized without constraints using the larger def2-TZVP(-f) basis set. Thermochemical corrections were obtained at the same level of theory. Final single point energy calculations were carried out with the domain-based-local-pair-natural-orbitals coupled cluster method with single, double and perturbative triple excitations (DLPNO-CCSD(T))^58–63^ together with the def2-TZVP basis set. The corresponding correlation-fitting basis set (def2-TZVP/C) was used. The NormalPNO settings with a tightened TCutPairs value of 1 × 10^−5^ Hartree were used. In addition, the RIJCOSX approximation^64^ to the exchange integrals was used together with the matching Coulomb-fitting basis set. Tight Grids (GRIDX7) and very tight SCF settings (VerytightSCF) were used. Solvation corrections were included at the B3LYP-D3/def2-TZVP^51,65–67^ level of theory with the solvation model based on density (SMD).^68^ Cyclohexane (CyH) was used as solvent. The final Gibbs free energy 
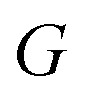
 was calculated *via*:1
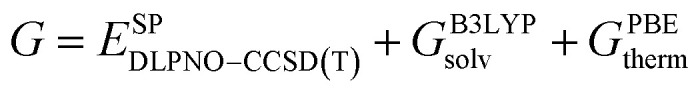


For the analysis of the key non-covalent interactions, the local energy decomposition (LED)^69–74^ was used to decompose free energy reaction barriers Δ*G*^‡^ into contributions associated with the interaction of the catalyst and the substrate:2
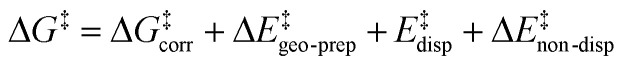


In which 
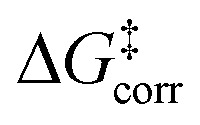
 represents the thermostatistical correction to the barrier, including all thermal corrections, entropic and solvent contributions. 
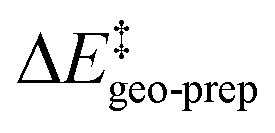
 is the geometric preparation energy, that is, the needed energy in order to distort the catalyst and the substrate from their equilibrium geometries to the geometries they have in the transition state. 
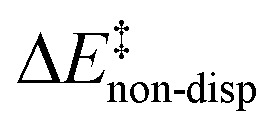
 represents the non-dispersive part of the interaction between the catalyst and the substrate, including all steric and polarization effects, while 
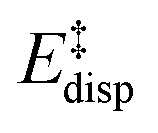
 denotes the London dispersion component of the interaction.

Steric maps were obtained with the SambVca 2.1 web tool.^75^ The protonated nitrogen atom of the catalyst was used as the center of the sphere with a radius of 7 Å. The 3D models are oriented in the same way as the steric maps, with the *x*-axis pointing to the right, the *y*-axis pointing upward and the *z*-axis pointing towards the reader. All distances in the steric maps are given in Å. The percentage of free volume within the sphere, % V_free_, is used in this work as a qualitative measure of “free” volume in the catalyst pocket that is accessible to the substrate.

For calculating the noncovalent interaction (NCI) plots, we used the NCIPLOT program.^76–78^ They were computed based on the electron density at the B3LYP-D3/def2-TZVP level of theory.

## Results and discussion

### Mechanism

**Fig. 2:**
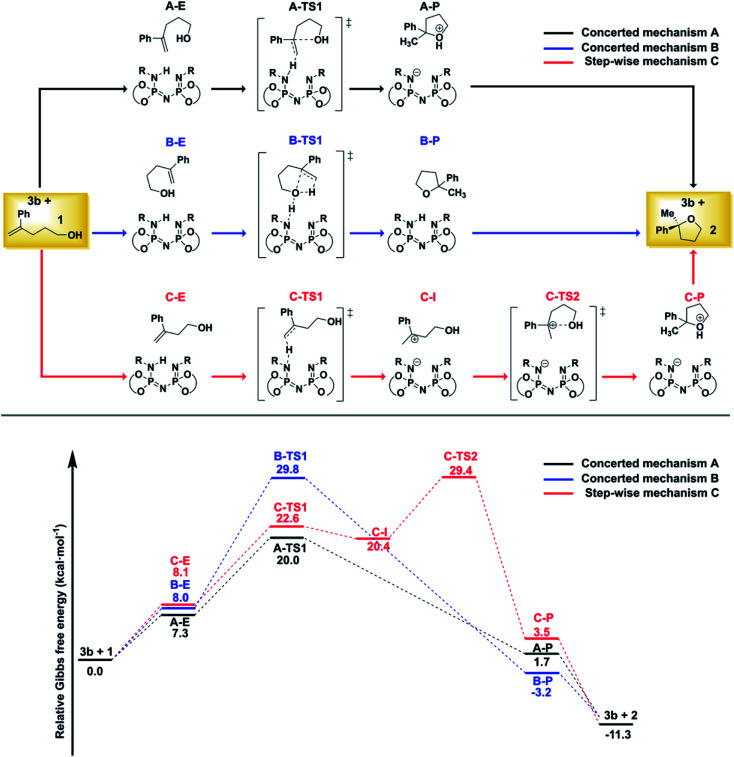
Computed mechanisms at the DLPNO-CCSD(T)/def2-TZVP level of theory. All energies are in kcal mol^−1^. See text for details.

For the reaction illustrated in Scheme 1, three different pathways were explored (Fig. 2).

While the substrate–catalyst adducts A–E, B–E and C–E are all very close in energy, the different conformations of the substrate in these complexes lead to completely different reaction pathways. In the “concerted mechanism A”, the proton from the catalyst is transferred to the terminal methylene group of the folded substrate with simultaneous C–O bond formation, leading to the five-membered oxacycle in a single step. In contrast, the “step-wise mechanism C” involves the unfolded carbocationic species C–I as reaction intermediate. Finally, the “concerted mechanism B” pathway involves a hydrogen-bond assisted proton transfer from the O–H group of the folded substrate to the methylene group, with simultaneous C–O bond formation.

The comparison of the energy profiles associated with the three competing pathways indicates that the “concerted mechanism A” is preferred. As discussed in ref. 38, this pathway is consistent with the experimental observation that the non-terminal isomer of 1 does not react under the experimental conditions (both isomers would proceed through the same carbo-cationic intermediate within a step-wise mechanism).

**Fig. 3:**
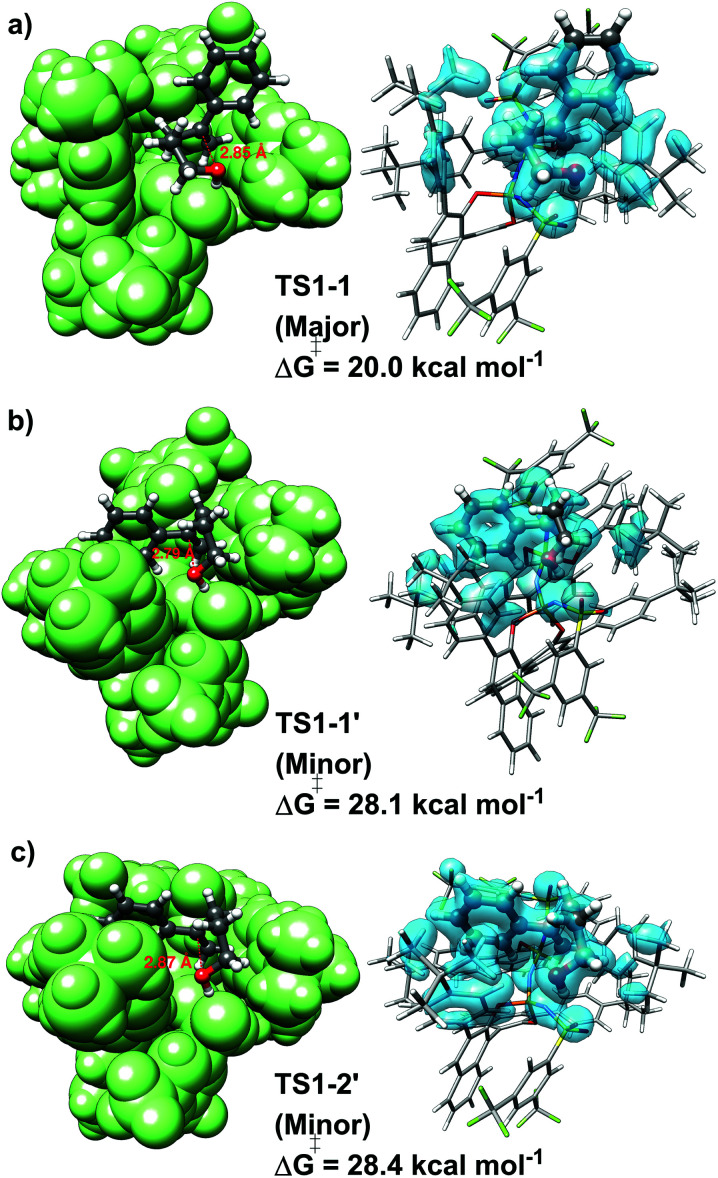
Low-energy transition state conformers leading to the major (panel a, TS1-1) and minor (panel b, TS1-1′, panel c, TS1-2′) enantiomeric products. The corresponding free energy barriers (Δ*G*^‡^) are given in kcal mol^−1^. On the left, a green-colored space filling model is used for the catalyst to emphasize the conformation of the substrate inside the catalyst pocket. On the right, dispersion interaction density (DID) surfaces are shown in light blue. The density value for the isosurface is 0.01 kcal (mol^−1^ bohr^3^).

For the “concerted mechanism A”, the lowest-in-energy transition states leading to the major and minor enantiomeric product are shown in Fig. 3.

It is important to emphasize here that, for the pathway leading to the major enantiomeric product, all the low-energy TS conformers show the same qualitative structural features. Hence, only the lowest in energy structure is shown in Fig. 3 (TS1-1). In contrast, for the minor enantiomeric pathway, two transition states with significantly different structural features and similar energies were identified (TS1-1′, TS1-2′). This aspect is discussed in more detail in the next section. The higher in energy TS conformers are given in the ESI.†

Consistent with the experimental findings, the pathway leading to the major enantiomeric product is energetically favored. However, the difference between the computed free energy barriers ΔΔ*G*^‡^ associated with the major and minor pathways amounts to *ca.* 8 kcal mol^−1^, which is significantly larger than the experimentally determined value of 2.8 kcal mol^−1^. This deviation might have different origins, including the details of the chosen computational settings, such as the use of an implicit solvation scheme. Importantly, even though the use of different electronic structure methods, solvation schemes or basis sets influences the absolute magnitude of ΔΔ*G*^‡^, it remains the case that TS1-1 is always more stable than TS-1′ and TS1-2′, irrespective of the computational settings used (see Table S3, ESI†). Importantly, extrapolation of the coupled cluster electronic energies to the complete basis set limit reduces the ΔΔ*G*^‡^ by around 1 kcal mol^−1^, thus shifting the computed stereoselectivity closer to the experimental one (see Tables S5 and S6, ESI†). In the following, we will provide an in-depth, quantitative analysis of the physical and chemical origin of this energy difference, which will allow us to achieve a thorough understanding of the stereo-controlling factors of this transformation.

### Elucidating the stereo-controlling factors: induced fit *vs.* lock and key model

**Fig. 4:**
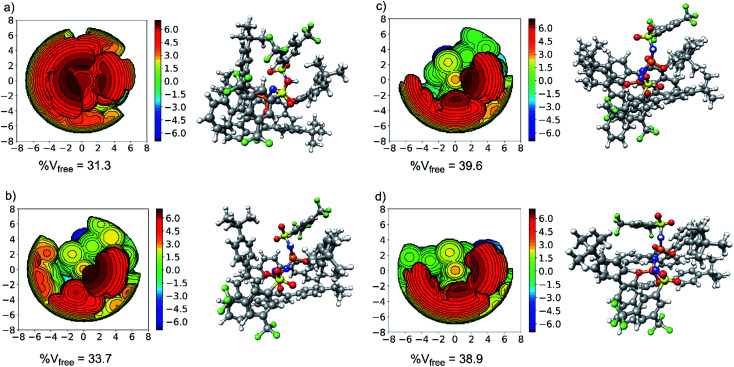
Steric map (left) and 3D model (right) for: (a) the catalyst in its equilibrium geometry; (b) the catalyst in its major transition state geometry; (c) the catalyst in its most stable minor transition state geometry (TS1-1′); (d) the catalyst in its second most stable minor transition state geometry (TS1-2′).

The aim of this section is to elucidate the mechanism through which the shape of the catalysts active site determines the selectivity of the transformation. The structure of the catalyst (3b) in its equilibrium geometry is shown in Fig. 4a. Steric maps are also shown in the same figure to emphasize its key structural features. In particular, the percentage of free volume within the catalyst pocket % V_free_ is given: smaller (larger) values of % V_free_ correlate with increasing (decreasing) confinement.

Our results show that the catalyst in its ground state is described by a closed and compact structure, which shields the active site. To enable interaction with the substrate 1, the catalyst pocket has to open up, which raises the catalyst energy with respect to its ground state of an amount that is expected to be roughly proportional to % V_free_. The “open” structure assumed by the catalyst in the transition state is shown in Fig. 4b for TS1-1 and Fig. 4c and d for TS1-1′ and TS1-2′, respectively.

In the transition states, the energy needed to distort the catalyst might be, at least partially, compensated by weak noncovalent interactions between the catalyst and the substrate upon adduct formation. Hence, a thorough understanding of the stereo-controlling factors in this and similar transformations can only be achieved through an in-depth quantitative understanding of both effects. The LED analysis is clearly an extremely useful tool in this context. The LED decomposition of the activation barriers Δ*G*^‡^ (eqn (2)) for the three pathways just discussed is shown in Table 1.

**Table 1:** Decomposition of the reaction barriers for the most stable major (TS1-1) and the two minor (TS1-1′, TS1-2′) transition state conformers at the DLPNO-CCSD(T) level of theory into geometric preparation, dispersive and non-dispersive interaction contributions. For the definition of the symbols, see Section on methodology. All energies are in kcal mol^−1^

	TS1-1	TS1-1′	TS1-2′	TS1-1′-TS1-1	TS1-2′-TS1-1
Δ*G*^‡^	20.0	28.1	28.4	8.1 (exp. 2.8)^38^	8.4 (exp. 2.8)^38^
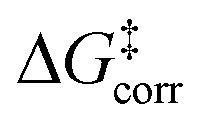	20.0	21.2	19.6	1.2	−0.4
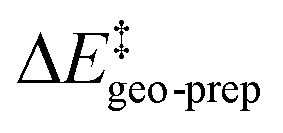	50.5 (41.3^a^ + 9.2^b^)	56.6 (43.2^a^ + 13.4^b^)	63.6 (50.0^a^ + 13.6^b^)	6.1 (1.9^a^ + 4.2^b^)	13.1 (8.7^a^ + 4.4^b^)
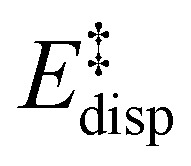	−38.2	−43.2	−45.4	−5.0	−7.2
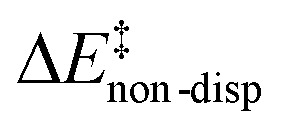	−12.2	−6.5	−9.5	5.7	2.8

a Contribution associated with the catalyst distortion.

b Contribution associated with the distortion of the substrate.

The first important finding of this analysis is that the activation barrier for the pathway leading to the major enantiomeric product (Δ*G*^‡^ = 20.0 kcal mol^−1^) is identical in magnitude to the thermal and entropic contribution 
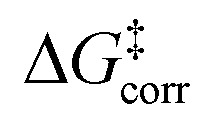
. This interesting effect originates from the fact that the increase in energy obtained when the catalyst structure is distorted from its ground state to the one that is optimal for interacting with the substrate (the dominant contribution in 
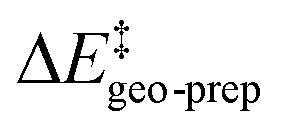
) is entirely compensated by the energy gain due to attractive substrate–catalyst interactions. Importantly, London dispersion between the catalyst and the substrate is the dominant stabilizing factor in this case 
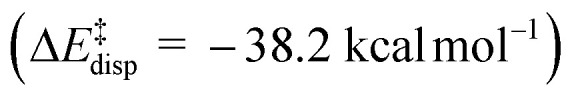
, while non-dispersive interactions provide a relatively minor but still noticeable contribution 
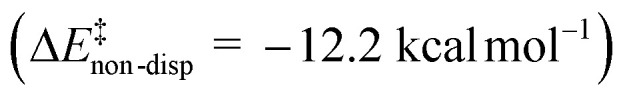
. In particular, there are strong dispersive interactions between the substrate and (i) both sulfonyl-groups of the catalyst, (ii) the aromatic rings of the aryl ligands and (iii) the *tert*-butyl groups of the pocket, as clearly shown *via* the DID plots^69,80^ in Fig. 3.

The pathways leading to the minor enantiomeric product show values of 
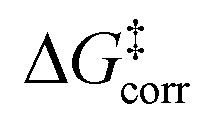
 that are essentially identical to those obtained for the major pathway, while the overall free energy barriers are much larger. This effect originates from the fact that the 
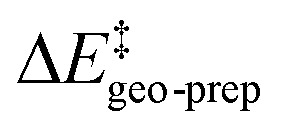
 terms for the minor pathways are significantly larger compared to TS1-1. The interaction energy between the catalyst and the substrate is almost identical for TS1-1 and TS1-1′, while TS1-2′ features a stronger substrate–catalyst interaction, which partially compensates the corresponding increase in 
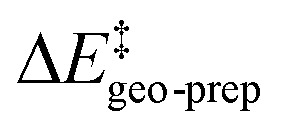
.

Thus, the catalyst–substrate interaction mechanism should be described as an “induced-fit” model, in which both the substrate and the catalyst adopt the conformation and orientation that is optimal for the interaction. The major pathway is the one that minimizes the energy penalty associated with the distortion of the catalyst and of the substrate 
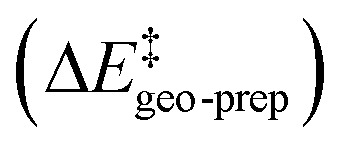
, while maximizing at the same time the catalyst–substrate interaction.

For the systems studied in this work, the distortion energy of the substrate correlates with the sp^3^-character of the methylene carbon atom. The factors contributing to the distortion energy of the catalyst, which is the dominant term in 
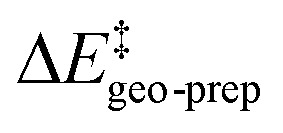
, are discussed in the next Section.

### Understanding catalyst distortion within the induced fit model

We now seek to discuss the factors that determine the increase in energy associated with the catalyst distortion, as quantified by the 
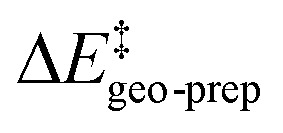
 term in the LED, which can be regarded as the most important contribution influencing the stereoselectivity of the catalyst within the induced-fit model, alongside with the catalyst–substrate interaction.

In order to understand the importance of individual groups or ligands, we performed a series of calculations in which we computed the catalyst contribution to 
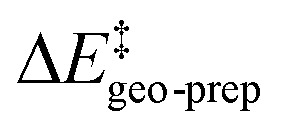
 for a series of subsystems (see the ESI† for details). These subsystems were obtained by sequentially substituting the groups of the catalyst 3b with hydrogen atoms. During these calculations, the catalyst geometry was not optimized, and only the positions of the newly added hydrogen atoms were relaxed.

**Fig. 5:**
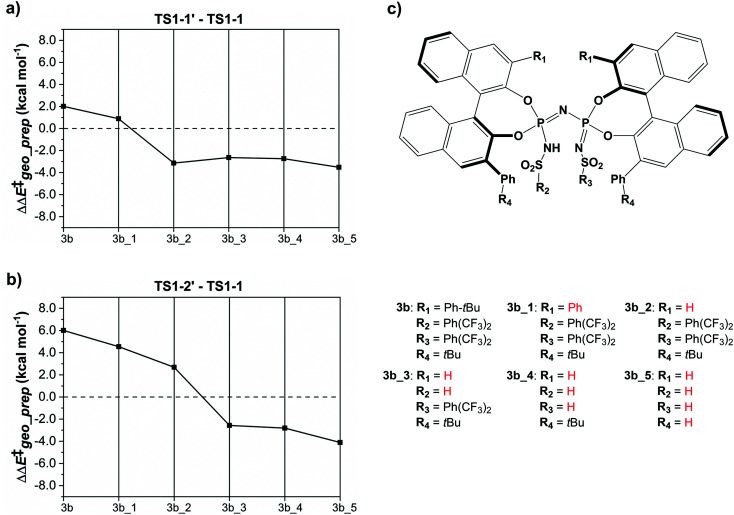
The energy difference between the catalyst structure in (a) TS1-1′ and TS1-1 (b) TS1-2′ and TS1-1 for a series of “subsystems” (3b, 3b_1, 3b_2, 3b_3, 3b_4, 3b_5). (c) Definition of the subsystems. Calculations were performed at the B3LYP-D3/def2-TZVP level of theory. See text for details.

The results of this study are shown in Fig. 5. In this figure, the subsystems are numbered from 3b_1 to 3b_5; 
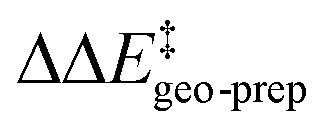
 denotes the difference between the catalyst contribution to 
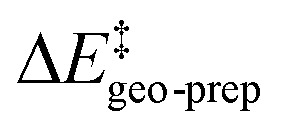
 of the minor and major pathways.

Consistent with the results discussed in the previous section, 
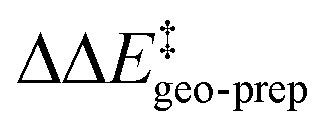
 is positive for the original catalyst 3b, which indicates that the increase in energy associated with the distortion of the catalyst within the induced fit model is smaller for TS1-1 than for TS1-1′ and TS1-2′. Interestingly, by progressively replacing bulky groups with hydrogen atoms, 
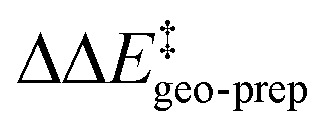
 becomes smaller and smaller. For TS1-1′, the largest effect is associated with the substitution of the phenyl rings of the aryl-ligands (3b_1 → 3b_2), while for TS1-2′ it stems from the substitution of the Ph-(CF_3_)_2_ group at the catalyst's amine group (3b_2 → 3b_3). In both cases, the substitution leads to a change in the sign of 
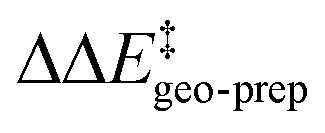
.

For TS1-1′, the 3b_1 → 3b_2 substitution reduces 
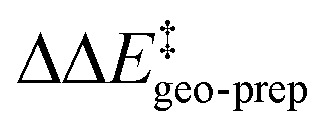
 by −4.0 kcal mol^−1^. About 50% of this energy change (−1.9 kcal mol^−1^) is associated with the corresponding change in the intra-catalyst dispersion forces (see ESI† for details). For TS1-2′, the 3b_2 → 3b_3 substitution reduces 
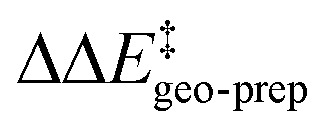
 by −5.3 kcal mol^−1^. Again, intra-catalyst dispersion provides a fundamental contribution (−5.0 kcal mol^−1^). Thus, within the induced-fit model, intra-catalyst dispersion forces play an especially important role in determining the selectivity of this transformation, as they are largely responsible for the magnitude of 
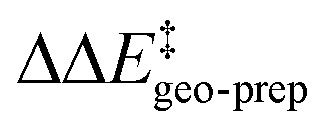
.

**Fig. 6:**
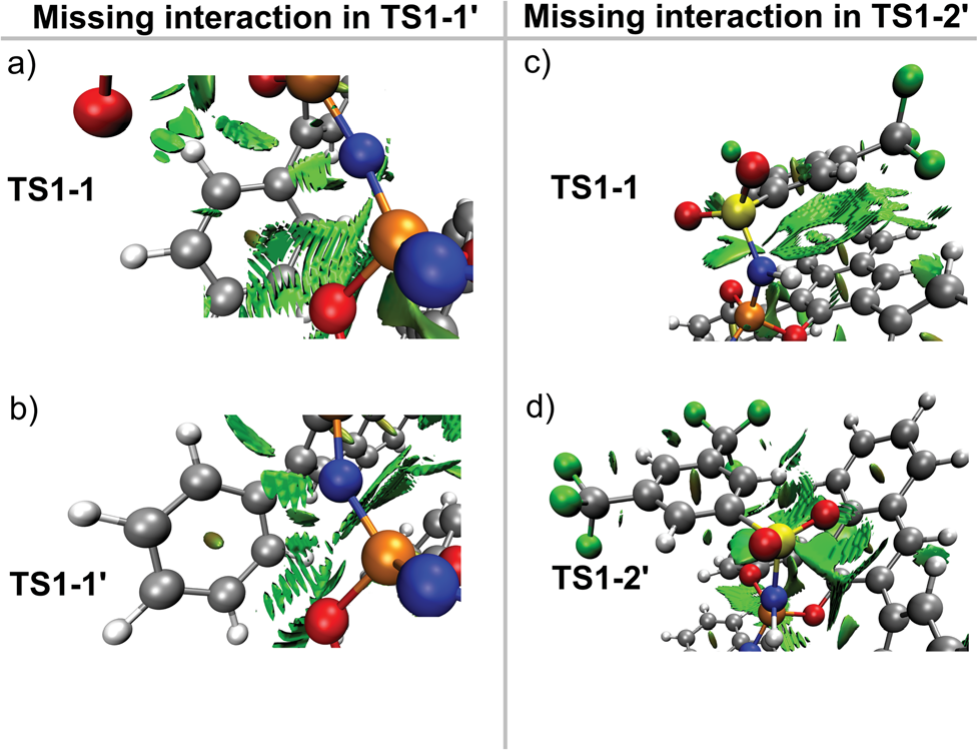
(a) NCI plot associated with the interaction between the Ph group and the catalyst backbone in TS1-1 (major pathway). (b) The analogous NCI plot for TS1-1′ (minor pathway). (c) NCI plot associated with the interaction between the Ph-(CF_3_)_2_ group and the catalyst backbone in TS1-1 (major pathway). (d) The analogous NCI plot for TS1-2′ (minor pathway). The density isovalue for the NCI plots is 0.5 a.u.

To illustrate the origin of this effect, we show in Fig. 6 the NCI plots obtained for the intramolecular interaction of the backbone of catalyst 3b with either the Ph ring at the aryl ligand (Fig. 6a and b) or the Ph-(CF_3_)_2_ group (Fig. 6c and d) in the relevant transition states. These plots show that interactions that are strong and attractive in TS1-1 are essentially absent in TS1-1′ (Fig. 6b) and TS1-2′ (Fig. 6d).

Thus, for the major reaction pathway, the catalyst distortion associated with the interaction with the substrate does not disrupt the stabilizing Ph-⋯backbone or Ph-(CF_3_)_2_⋯backbone London dispersion interactions. In contrast, for each of the minor reaction pathways, some of the stabilizing intra-catalyst interactions are missing to facilitate the interaction with the substrate. In other words, only if the substrate and the catalyst are in the correct relative orientation (the one that leads to the formation of the major enantiomeric product) the energy penalty associated with the catalyst distortion can be minimized.

To summarize the results obtained so far, we have demonstrated that: (i) the energy required to distort the catalyst is a key factor controlling the selectivity of the transformation within an induced-fit model; (ii) this energy penalty is dominated by the change in the intra-catalyst dispersion interactions on going from the ground state of the catalyst to the structure that is optimal for the interaction with the substrate. Thus, a simple possible path towards rational catalyst design is the identification of confined catalysts that are largely stabilized by dispersion forces. This can be done by introducing bulky groups that act as dispersion energy donors,^24,73,79^ such as π systems, whose interaction with the catalyst backbone might or might not be disrupted upon the interaction with the substrate along specific reaction pathways.

In the next Section we will show how this simple argument can be used to rationalize catalyst selectivity in a series of previously published asymmetric transformations.

These results demonstrate that intra-catalyst dispersion forces are a fundamental factor contributing to the structural stability of these confined catalysts. In addition, as they are responsible for the magnitude of 
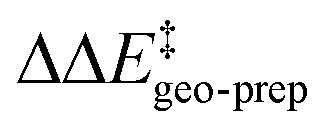
, they control to a large extent the selectivity of this transformation.

### Testing the model

**Fig. 7:**
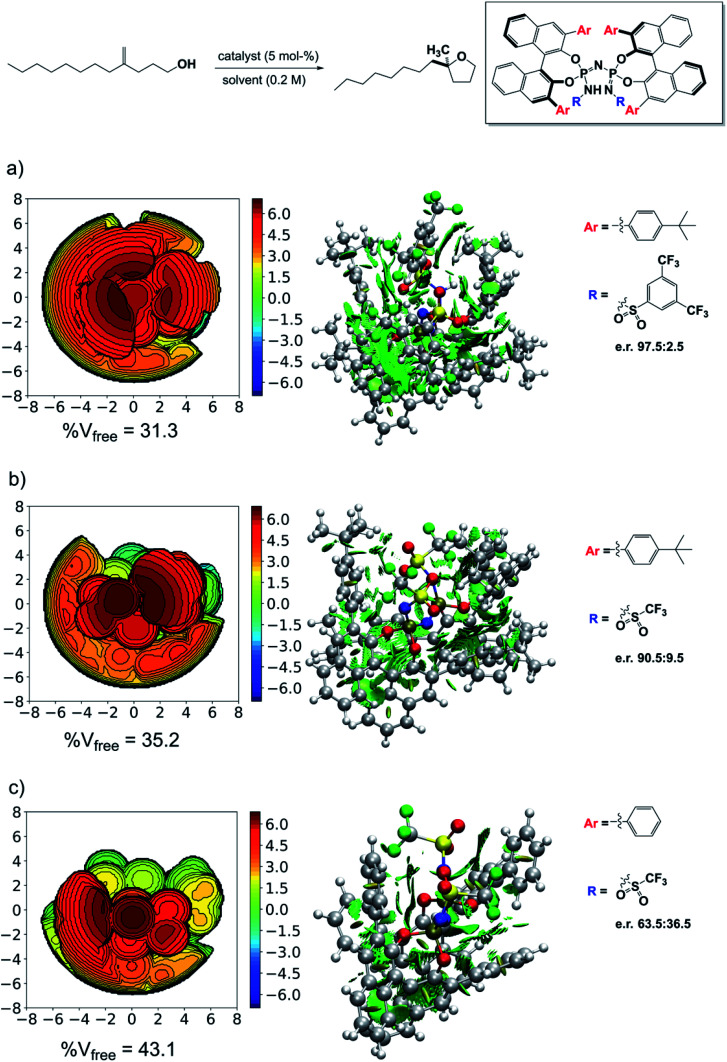
Steric maps, NCI plots and experimental enantiomeric ratios for different catalysts in their geometric ground state. (a) Catalyst 3b. (b) Catalyst 3a. (c) Catalyst 3d. The isovalue for the density in the NCI plots is 0.5 a.u.

The results just discussed can be used to rationalize previously published experimental findings and will hopefully aid in the design of new catalysts with improved selectivity. As an example, we show in Fig. 7 three catalysts with different experimental selectivity for the same reaction investigated in this work but using a different substrate, *i.e.*, 4-methylenedodecan-1-ol. In the figure, the catalyst equilibrium geometry is shown alongside with the associated steric maps and NCI plots.

The experimental results were taken from ref. 38. Catalyst 3d showed almost no stereoselectivity (e.r. 63.5 : 36.5), catalyst 3a is significantly more selective (e.r. 90.5 : 9.5), and catalyst 3b is the most selective (e.r. 97.5 : 2.5). Remarkably enough, analysis of the steric maps reveal that the catalyst selectivity does correlate with catalyst confinement as quantified by % V_free_. As expected, based on our previous analysis, the NCI plots also reveal an increase of stabilizing noncovalent interactions with increasing confinement, which fully supports our dispersion-based induced-fit model for these systems.

## Conclusions

We elucidated the mechanism and stereo-controlling factors of the stereoselective intramolecular hydroalkoxylation of terminal olefins catalyzed by confined IDPi Brønsted acids using state of the art computational techniques. It was found that the catalyst–substrate adduct formation is better described using an “induced-fit” model rather than a “key-and-lock” mechanism, in which both the substrate and the catalyst adopt the conformation and orientation that is optimal for the interaction. Thus, the major pathway minimizes the energy penalty associated with the distortion of the catalyst, while maximizing at the same time the attractive catalyst–substrate interactions. Remarkably enough, the energy penalty associated with the catalyst distortion is dominated by the change in the intramolecular dispersion interactions within the catalyst, while intermolecular catalyst–substrate dispersion interactions are the major stabilizing contribution in the transition state. Thus, a simple possible path towards rational catalyst design is the identification of confined catalysts that are largely stabilized by dispersion forces, *e.g*., *via* the use of appropriate dispersion energy donors.

## Data availability

The Cartesian coordinates of all structures, the results of benchmark calculations on transition state geometries, energies, and thermochemical corrections, local energy decomposition analyses for the low-energy transition states and a detailed analysis of the key noncovalent interactions are provided in the ESI.†

## Author contributions

I. H. performed all the calculations. G. B. conceived and directed the project. I. H. and G. B. wrote the original draft of the manuscript and prepared the figures. G. B., F. N. and I. H. contributed to the data analysis and to the writing of the manuscript.

## Conflicts of interest

There are no conflicts to declare.

## Supplementary Material

SC-013-D2SC02274E-s001
