# Supplementary material for: An induced-fit model for asymmetric organocatalytic reactions: a case study of the activation of olefins via chiral Brønsted acid catalysts
Source: Chem Sci. 2022 Jul 4;13(30):8848–59. doi: 10.1039/d2sc02274e (PMC9350588; doi:10.1039/d2sc02274e)
Supplement: SC-013-D2SC02274E-s001 [file SC-013-D2SC02274E-s001.pdf]

Electronic Supplementary Material (ESI) for Chemical Science.
This journal is © The Royal Society of Chemistry 2022

An induced-fit model for asymmetric organocatalytic reactions: a case study of the activation of olefins *via* chiral Brønsted acid catalysts – Supporting Information

Ingolf Harden [a], Frank Neese [a], Giovanni Bistoni [a][b]

[a]*Max-Planck-Institut für Kohlenforschung, Kaiser-Wilhelm Platz 1, 45470 Mülheim an der Ruhr (Germany)*

[b]*Department of Chemistry, Biology and Biotechnology, University of Perugia Via Elce di Sotto, 8, 06123 Perugia (Italy). E-mail: giovanni.bistoni@unipg.it*

Dependency of transition state geometry on computational settings

Here we discuss the influence of different density functionals as well as basis sets on key internal coordinates of the low energy conformers.

Table S1. Key bond lengths for the transition state conformers **TS1-1** and **TS1-2'** for different density functionals and basis sets. All bond lengths are in Å.

**TS1-1**	PBE-D3/ def2-SVP	PBE-D3/ def2-TZVP(-f)	B3LYP-D3/ def2-TZVP(-f)
N-H	0.1337	0.1358	0.1363
C-H	0.1370	0.1360	0.1364
C-O	0.2686	0.2855	0.2886
H---O	0.1792	0.1891	0.1900
RMSD	0.1210	0.0000	0.1010

**TS1-2'**	PBE-D3/<br>def2-SVP	PBE-D3/<br>def2-TZVP(-f)	B3LYP-D3/<br>def2-TZVP(-f)
N-H	0.1367	0.1370	0.1368
C-H	0.1330	0.1329	0.1337
C-O	0.2787	0.2868	0.2858
H---O	0.1800	0.1924	0.2004
RMSD	0.4560	0.0000	0.2540

From **Table S1** it can be seen that the basis set size has a larger impact on the geometry than the density functional. While the bond lengths of the TS mode (N-H/C-H bonds) are quite uneffected of both basis set and density functional, the C-O bond length is affected the most. However, the overall changes in geometry are quite small as reflected by the RMSD values. For the larger def2-TZVP(-f) basis set, we seem to be justified in using the computationally less demanding PBE density functional. The constrained geometry optimizations as well as the transition state optimizations were performed *in vacuo* and implicit solvation corrections added to the final Gibbs free energy were obtained from DFT single point energy calculations. In **Table S2** we show the influence of implicit solvation on the transition state geometry of **TS1-1**. For this, the TS geometry was reoptimized using implicit solvation with Cyclohexane as solvent.

Table S2. Important bond lengths for the most stable TS conformer **TS1-1** optimized with and without implicit solvation at the PBE-D3/def2-TZVP(-f) level of theory. All bond lengths are in Å.

**TS1-1**	Vacuum	CPCM(Cyclo-<br>hexane)
N-H	0.1358	0.1367

C-H	0.1360	0.1354
C-O	0.2855	0.2929
H---O	0.1891	0.1887
RMSD	0.0000	0.0760

The influence of implicit solvation on the TS geometry is fairly small as reflected by the RMSD values. The critical internal coordinates show some moderate changes, but overall the conformation of the system is unchanged. This justifies the use of *in vacuo* optimized geometries for further calculations and analyzes.

Energy ordering of the most stable transition states at different levels of theories

In **Table S3** we introduce 9 different low-energy conformers leading to the major or minor enantiomeric product, respectively. In the main manuscript we discussed the most stable major **TS1-1** and the two two most stable minor conformers **TS1-1'** and **TS1-2'**. We showed there only the DLPNO-CCSD(T) electronic energies. Here, we discuss the electronic energy ordering at different levels of theories. The results can be found in **Table S3**.

Table S3. Relative electronic energies of low-energy transition states at various levels of theory. All energies are obtained for the PBE-D3/def2-TZVP(-f) optimized geometries. All energies are in kcal mol^{-1}.

	Hartree-Fock/ def2-TZVP	DLPNO-CCSD/ def2-TZVP	DLPNO-CCSD(T)/ def2-TZVP	PBE-D3/ def2-TZVP(-f)	B3LYP-D3/ def2-TZVP
**TS1-1**	0.0	0.0	0.0	0.0	0.0
**TS1-2**	10.2	9.7	9.4	7.8	8.0

**TS1-3**	1.0	0.4	0.2	-0.1	0.0
**TS1-4**	5.2	3.3	2.8	1.2	1.6
**TS1-1'**	17.7	8.4	6.9	4.6	3.6
**TS1-2'**	17.7	10.1	8.7	5.8	4.4
**TS1-3'**	13.5	9.0	8.0	4.9	4.6
**TS1-4'**	13.3	10.7	10.1	8.3	8.9
**TS1-5'**	10.0	8.9	8.5	6.9	7.1

We first discuss the results for the transition states leading to major enantiomeric product (**TS1-1** – **TS1-4**). All methods predict the guess conformer **TS1-2** (reoptimized at the PBE-D3/def2-TZVP(-f) level of theory) to be highly unfavored in energy with respect to the other conformers, reflecting the potential importance of exploration of the conformational space for large and flexible systems. Correlated wave function methods (DLPNO-CCSD and DLPNO-CCSD(T)) as well as DFT predict smaller relative energy differences for **TS1-2** accordingly with respect to Hartree-Fock. For the conformers **TS1-3** and **TS1-4**, the DLPNO-CCSD(T) and DFT almost yield the same relative energies as well. It is important to note that the low-energy conformers (**TS1-1**, **TS1-3**, **TS1-4**) show quite similar structural features. For the transition states leading to the minor enantiomeric product (**TS1-1'** – **TS1-5'**), we see that Hartree-Fock predicts very high relative energies, indicating the inclusion of correlation stabilizes the minor enantiomers relative to the major transition states. The initial guess conformer **TS1-4'** is significantly higher in energy for Coupled-Cluster and DFT methods. This effect is considerably large for the B3LYP functional, predicting **TS1-4'** to be 5.3 kcal mol^{-1} higher in energy than the most stable transition state leading to minor enantiomeric product (**TS1-1'**). In total, DFT and DLPNO-CCSD(T) predict the same energy ordering of the major transition states, while for the minor conformers DFT predicts smaller relative energies. Interestingly, **TS1-1'** is the most stable

minor transition state at both, DFT and DLPNO-CCSD(T) level of theory, while for the other minor conformers the energy ordering might change with respect to the method.

Thermochemical corrections at the r2SCAN-3c level of theory

In order to test the reliability of the thermochemical corrections calculated at the PBE-D3/def2-TZVP(-f) level of theory, the frequencies and thermochemical corrections were also computed at the r2SCAN-3c level.[1] The r2SCAN-3c calculations were carried out with ORCA 5.0.3.[2]

Table S4. Difference of thermochemical corrections between the major (**TS1-1**) and minor (**TS1-1'**, **TS1-2'**, **TS1-3'**, **TS1-4'**, **TS1-5'**) transition states. All energies are in kcal mol^{-1}.

	**PBE-D3(BJ)/def2-TZVP(-f)**	**r2SCAN-3c**
**TS1-1' - TS1-1**	1.26	1.32
**TS1-2' - TS1-1**	-0.03	0.04
**TS1-3' - TS1-1**	1.02	0.34
**TS1-4' - TS1-1**	-0.04	-0.38
**TS1-5' - TS1-1**	0.30	0.63

For all transition state conformers, the difference in thermochemical correction between PBE-D3/def2-TZVP(-f) and r2SCAN-3c is rather small. The largest influence is on **TS1-3' – TS1-1** (-0.68 kcal mol^{-1}), while for all other transition states the difference between the methods is below 0.5 kcal mol^{-1}.

Extrapolation of the DLPNO-CCSD(T) energies to the basis set limit

The DLPNO-CCSD(T) energies of **TS1-1**, **TS1-1'** and **TS1-2'** were extrapolated to the basis set limit in order to quantify the error of incomplete basis set to the stereoselectivity. The extrapolation was

performed with ORCA 5.0.3 and the def2-SVP (cardinal number X=2) and def2-TZVP (X=3) basis sets were used. The triples amplitudes were computed iteratively (T1) and implicit solvation was included *via* SMD. The results are shown in **Table S5**.

Table S5. Difference of electronic energies at the DLPNO-CCSD(T) level between the major (**TS1-1**) and minor (**TS1-1'**, **TS1-2'**) transition states. All energies are in kcal mol^{-1}.

	**TS1-1'-TS1-1**	**TS1-2'- TS1-1**
ORCA 4.2.1 def2-TZVP	6.880	8.743
ORCA 5.0.3 def2-TZVP	7.086	8.433
ORCA 5.0.3 (2/3)-extrapolated	6.228	8.299

As can be seen, the ORCA version has a rather small influence on the stereoselectivity. For both minor transition states, basis set extrapolation reduces the stereoselectivity and therefore brings it closer to the experimental value, while in general the stereoselectivity still is overestimated.

Extrapolation of the DLPNO-CCSD energies to the pair natural orbital space limit

In addition to the extrapolation towards the basis set limit, extrapolation towards the pair natural orbital (PNO) space limit was carried out (Complete PNO Space, CPS).[3] For this, the PNO(6/7)-scheme was used: DLPNO-CCSD calculations with the def2-SVP basis set were carried out once with TCutPNO = 1e-6 and again with TCutPNO=1e-7. The correlation energy was extrapolated *via:*

$$E_{corr,PNO(6,7)} = E_{corr,PNO6} + 1.5 * [E_{corr,PNO7} - E_{corr,PNO6}]. \quad \text{(I)}$$

The results are shown in **Table S6**.

Table S6. Difference of correlation energies at the DLPNO-CCSD/def2-SVP level between the major (**TS1-1**) and minor (**TS1-1'**, **TS1-2'**) transition states for different values of TCutPNO. All energies are in kcal mol^{-1}.

	**TS1-1'-TS1-1**	**TS1-2'- TS1-1**
TcutPNO=1e-6	-8.016	-7.120
TCutPNO=1e-7	-7.997	-7.263
PNO(6/7)-extrapolation	-7.987	-7.335

The influence of the extrapolation of the TCutPNO value on the difference in correlation energy is only marginally for **TS1-1'** (0.010 kcal mol^{-1} with respect to TCutPNO=1e-7) while for **TS1-2'** there is a larger, but in total still small influence (-0.072 kcal mol^{-1} with respect to TCutPNO=1e-7). The value of TCutPNO used in the DLPNO-CCSD(T) calculations reported in the main manuscript was 3.33e-7, so that the net stabilization of **TS1-2'** with respect to **TS1-1** due to the CPS-extrapolation is expected to be around 0.1 kcal mol^{-1}.

Transition state optimizations at the B3LYP-D3(BJ)/def2-TZVP(-f) level of theory

The most stable major transition state and the low-energy minor transition states were reoptimized using ORCA 5.0.3 and the B3LYP functional. In **Table S7** the relative energies of the reoptimized transition states at the B3LYP-D3/def2-TZVP(-f) level of theory are compared to the previously obtained transition states at the PBE-D3/def2-TZVP(-f) level of theory.

Table S7. Relative electronic energies of low-energy transition states. Geometries and energies are at the PBE-D3/def2-TZVP(-f) or B3LYP-D3/def2-TZVP(-f) level of theory, respectively. All energies are in kcal mol^{-1}.

	PBE-D3/def2-TZVP(-f)	B3LYP-D3/def2-TZVP(-f)
**TS1-1**	0.0	0.0
**TS1-1'**	4.6	4.3
**TS1-2'**	5.8	5.1
**TS1-3'**	4.9	5.2
**TS1-4'**	8.3	9.0
**TS1-5'**	6.9	7.1

As **Table S7** demonstrates, for both methods quite similar relative energies are obtained. Noticeably, the most stable minor conformers in terms of free energies (**TS1-1'** and **TS1-2'**) show lower relative energies at the B3LYP-D3 level of theory with respect to the PBE-D3 level of theory.

Local Energy Decomposition for most stable transition states

In **Table S8** we report the LED results for the transition state conformers listed in **Table S3**. In the main manuscript conformers **TS1-1**, **TS1-1'** and **TS1-2'** were considered (see **Table 1**).

Table S8. Decomposition of the reaction barriers for 9 transition state conformers at the DLPNO-CCSD(T) level of theory into geometric preparation, dispersive and non-dispersive interaction contributions. For the definition of the symbols, see the main manuscript. All energies are in kcal mol^{-1}.

	**TS1-1**	**TS1-2**	**TS1-3**	**TS1-4**	**TS1-1'**	**TS1-2'**	**TS1-3'**	**TS1-4'**	**TS1-5'**
$\Delta G^{\ddagger}$	20.0	29.1	21.1	23.3	28.1	28.4	28.6	28.8	29.1
$\Delta G^{\ddagger}_{corr}$	20.0	19.7	20.8	20.5	21.2	19.6	20.5	18.6	20.5
$\Delta E^{\ddagger}$	0.0	9.5	0.3	2.9	6.9	8.8	8.1	10.2	8.6
$\Delta E^{\ddagger}_{geo-prep}$	50.4	57.5	51.0	49.8	56.6	63.6	61.4	58.6	64.9

Cat.	41.3	46.9	43.0	40.6	43.2	50.0	49.3	48.8	55.6
Substrate	9.2	10.6	8.0	9.2	13.4	13.6	12.1	9.8	9.3
$\Delta E^{\ddagger}_{int}$	-50.4	-49.4	-52.1	-48.3	-49.7	-54.8	-54.7	-49.8	-56.4
$E^{\ddagger}_{disp}$	-38.2	-38.8	-39.6	-41.0	-43.2	-45.4	-45.7	-38.0	-38.3
$\Delta E^{\ddagger}_{non-disp}$	-12.2	-10.6	-12.5	-7.4	-6.5	-9.4	-9.0	-11.8	-18.1

We start by discussing the conformers leading to the major enantiomeric product. The conformers **TS1-1**, **TS1-3** and **TS1-4** have DLPNO-CCSD(T) energies in the same range and it can be seen that geometric preparation is cancelled by the interaction energy between the catalyst and substrate (see main manuscript for detailed discussion). For the guess conformer **TS1-2** we see that the high relative DLPNO-CCSD(T) energy is mostly caused by geometric preparation. The increased geometric preparation for **TS1-2** is caused by the Ph-$(CF_3)_2$ group at the amine group of the catalyst, which does not allow for attractive π-π interactions (this is the same effect that causes the increased geometric preparation of **TS1-2'**, see *vide infra* and **Figure 6** for details). For the minor transition state conformers, it can be seen that geometric preparation is the most significant contribution to the difference in electronic energies with respect to **TS1-1**. In fact, the interaction between the catalyst and the substrate is even stronger for most of the minor transition states with respect to **TS1-1**. Interestingly, the most stable minor conformer **TS1-1'**, has the lowest geometric preparation energies of all minor TS conformers, but also the weakest interactions between the two fragments. For **TS1-2'** and **TS1-3'** the geometric preparation but also the attractive interactions between catalyst and substrate are increased with respect to **TS1-1'**. The stabilization is carried out by dispersive attraction between the catalyst and the substrate, while the non-dispersive interactions are decreased with respect to **TS1-1.** In conclusion, the geometric preparation is significantly increased for all minor conformers with respect to the major TS conformers. The strengthened attractive interactions for the minor conformers cannot counteract the geometric preparation leading to large energy differences

between the major and the minor TSs.

Visualization of discussed transition state conformers

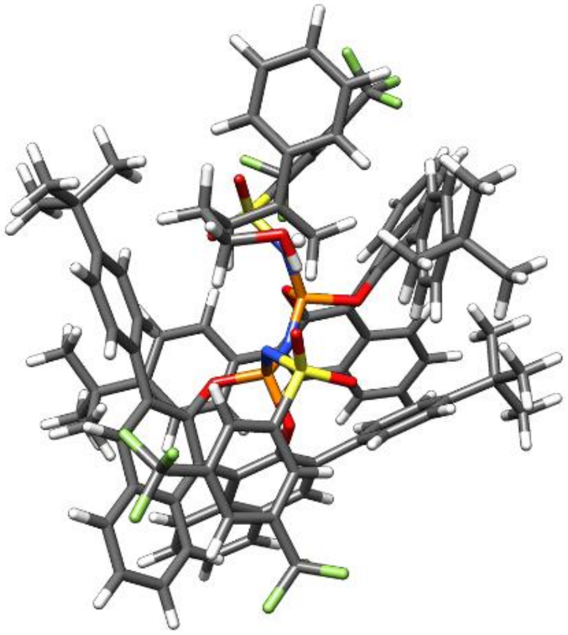

TS1-1

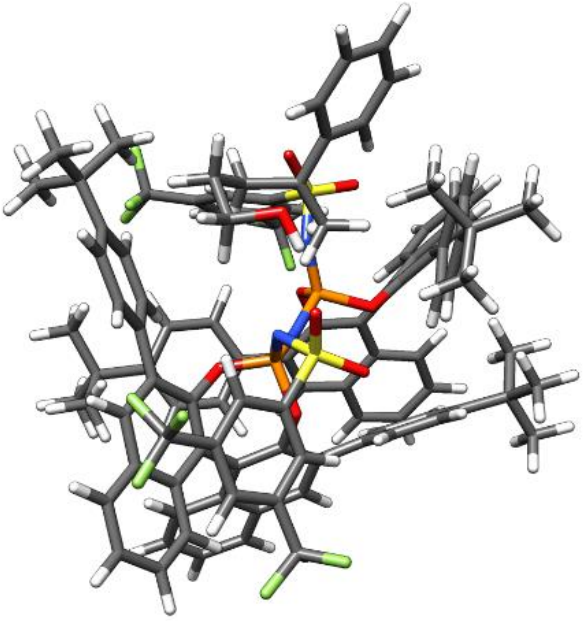

TS1-2

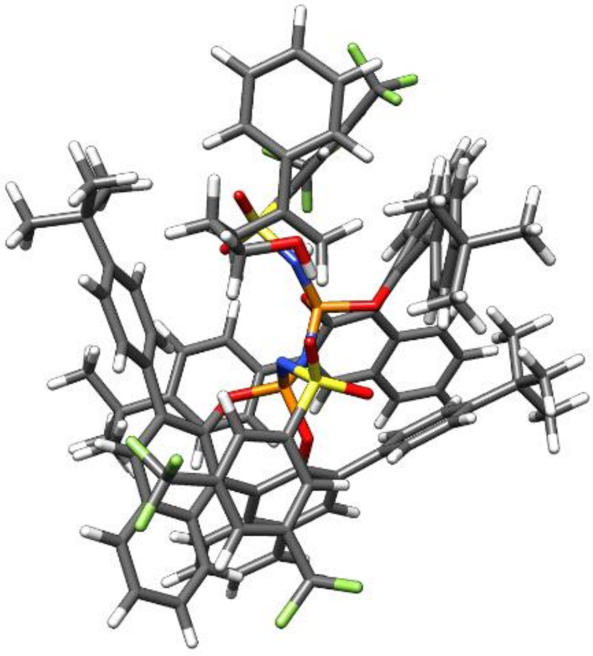

TS1-3

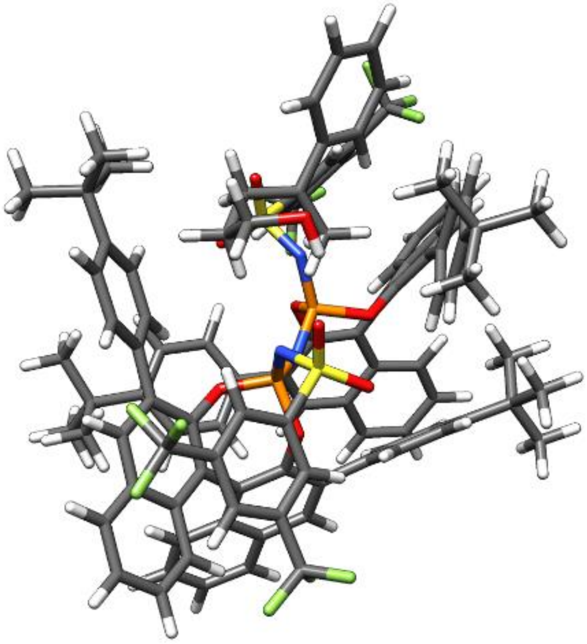

TS1-4

Figure S1. Four different transition state conformers leading to major enantiomeric product.

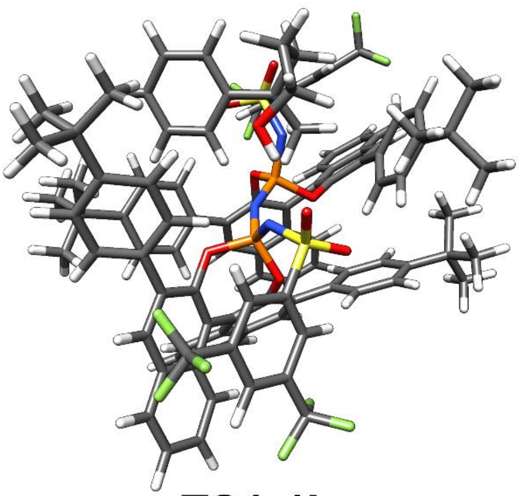

TS1-1'

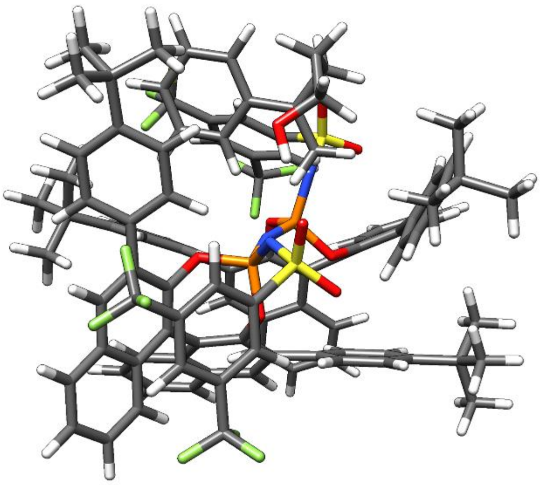

TS1-2'

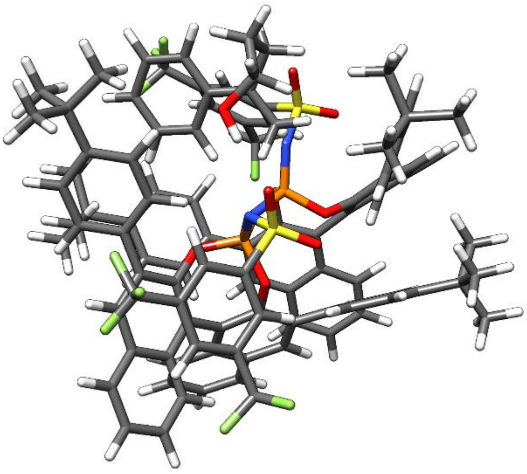

TS1-3'

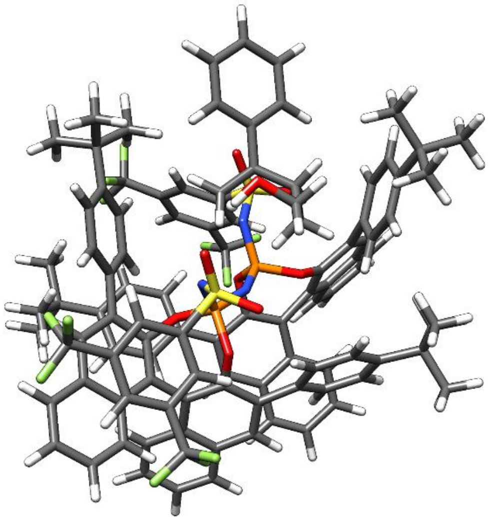

TS1-4'

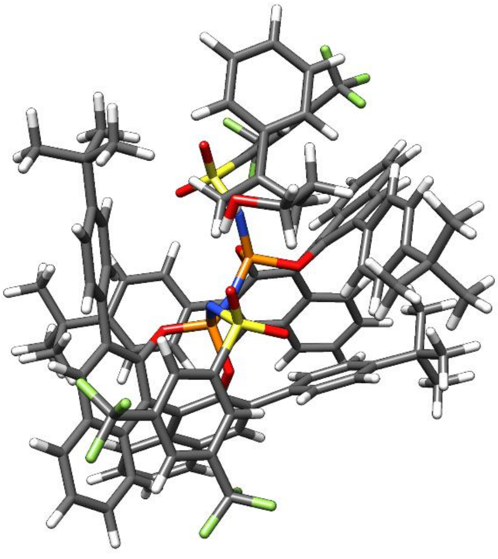

TS1-5'

Figure S2. Five different transition state conformers leading to the minor enantiomeric product.

Confinement analysis

We now discuss the results from the confinement analysis of the transition state conformers **TS1-1, TS1-1'** and **TS1-2'**. In the main manuscript we have already discussed the general procedure for the deconstruction of the catalyst and how we achieved analysis of the noncovalent interactions (see **Figure 5** and **Figure 6**). In **Table S9** we present the geometric preparation energies for TS conformers **TS1-1** and **TS1-1'** for the different deconstruction points labeled as **3b**, **3b_1**, **3b_2**, **3b_3**, **3b_4**, **3b_5**. The corresponding results for the comparison of **TS1-1** and **TS1-2'** can be found in **Table S10**. For measuring the influence of London dispersion, the geometric preparation was calculated with and without Grimme's D3(BJ) dispersion correction.

Table S9. Geometric preparation energies for TS conformers **TS1-1** and **TS1-1'** obtained from B3LYP-D3/def2-TZVP calculations. During the calculations only the relaxation of the newly added hydrogen atoms was allowed. For definition of the structures **3b** to **3b_5** see **Figure 5** of the main manuscript. In order to obtain the geometric preparation of the catalyst backbone, the geometric preparation energies given below were obtained by subtracting the needed energy for the N-H bond elongation (30.2 kcal mol^{-1} for **TS1-1**, 29.0 kcal mol^{-1} for **TS1-1'** with D3BJ, 30.6 kcal mol^{-1} for **TS1-1**, 29.2 kcal mol^{-1} for **TS1-1'** without D3BJ) from the total geometric preparation energy. All energies are in kcal mol^{-1}.

	Geo-prep (with D3BJ)			Geo-prep (without D3BJ)		
	**TS1-1**	**TS1-1'**	**TS1-1' - TS1-1**	**TS1-1**	**TS1-1'**	**TS1-1' – TS1-1**
**3b**	12.5	14.5	2.0	4.4	9.4	5.0
**3b_1**	13.3	14.2	0.9	4.7	11.0	6.3
**3b_2**	13.6	10.4	-3.2	4.8	9.0	4.2
**3b_3**	11.7	9.0	-2.7	3.4	7.0	3.6

**3b_4**	8.7	5.9	-2.8	2.8	5.7	2.9
**3b_5**	7.2	3.6	-3.6	2.3	5.2	2.9

It can be seen, that London dispersion has a large impact on the total geometric preparation. For **3b**, 65% for **TS1-1** and 35% for **TS1-1’** of geometric preparation stems from London dispersion. This underlines, that geometric preparation must not be exclusively understood as steric hindrance, since weakened or strengthened noncovalent interactions contribute to geometric preparation as well.

Interestingly, without the D3 dispersion correction, the difference in geometric preparation between **TS1-1’** and **TS1-1** remains much more constant for all six different structures and the difference always remains positive. When dispersive interactions are taken into account, the difference in geometric preparation becomes smaller from step to step and starting from **3b_2** even becomes negative. Starting from **3b_2**, the difference in geometric preparation remains negative and varies within a window of 0.5 kcal mol^{-1}. While substituting groups with hydrogen atoms, the difference in geometric preparation (and therefore the stereoselectivity) becomes smaller, the difference of non-dispersive geometric preparation remains constant. This illustrates, that for the major TS conformer **TS1-1** the removed groups must have had additional dispersive interactions that were not present for the minor TS conformer **TS1-2’**. If the group is removed, the additional dispersive interactions for **TS1-1** cease, leading to decreased difference in geometric preparation.

For all involved groups that are replaced during the deconstruction procedure, we now determine the attractive interactions for **TS1-1** and **TS1-1’** by using NCI plots. In order to analyze the interactions of a particular group, we use the 3D structure of the subsystem before this group is removed. The atomic color code for the **Figures S3 – S12** is: Grey, hydrogen; cyan, carbon; red, oxygen; gold, phosphorus; pink, fluorine; yellow, sulfur.

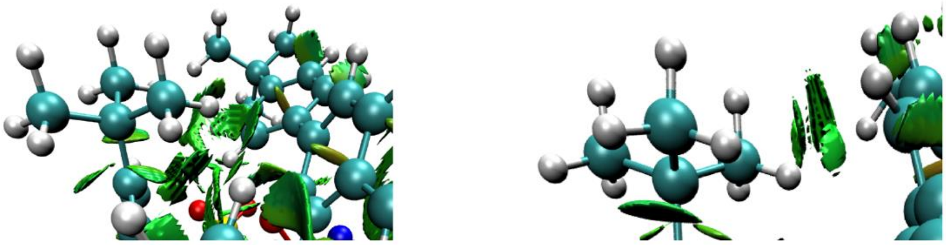

TS1-1 **TS1-1‘**

Figure S3. Noncovalent interactions of the *t*-Bu group of the BINOL aryl-substituent that does not form the catalyst pocket with the BINOL backbone (**3b_1**, see **Figure 5**). It can be seen, that for **TS1-1** additional dispersive interactions are present, which is why removal of this group leads do a relative stabilization of **TS1-1’**.

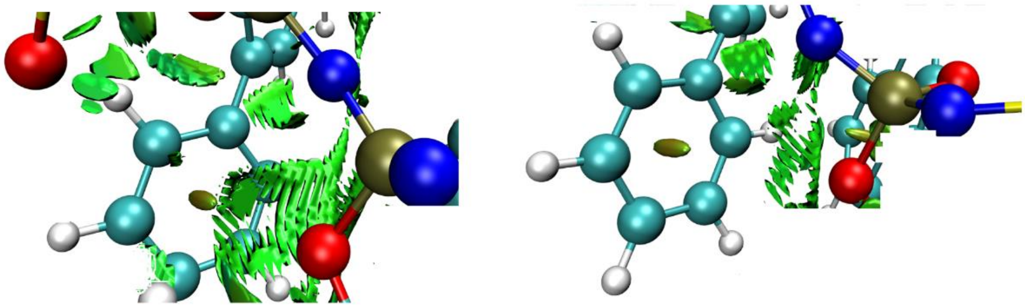

TS1-1 **TS1-1‘**

Figure S4. Noncovalent interactions of the Ph-ring bound to the BINOL substituent that is not forming the catalyst pocket (**3b_2**, see **Figure 5**). Due to geometric distortion, **TS1-1** is able to form stronger interactions with the catalyst’s backbone, especially with the core catalyst’s oxygen atom. Only for **TS1-1** strong attractive interactions of this group are present. Its removal therefore energetically favors **TS1-1’** with respect to **TS1-1**.

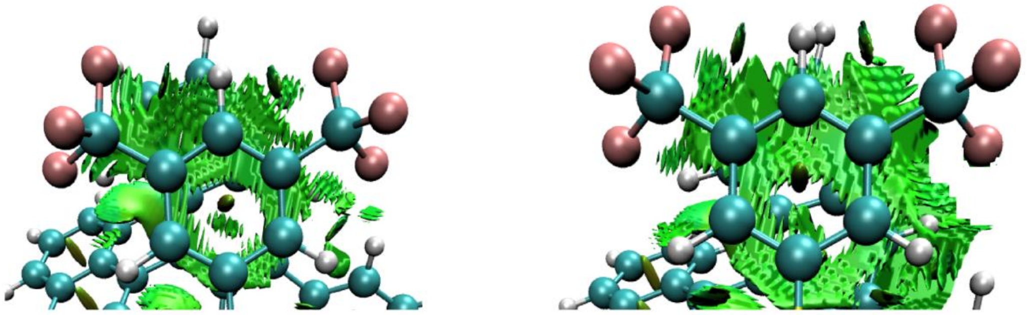

TS1-1 **TS1-1'**

Figure S5. Noncovalent interactions between the aminyl-Ph-$(CF_3)_2$ group and the residue (**3b_3**, see **Figure 5**). For both conformers there are equally strong interactions with the ligands Ph-rings. That is why removal of this group does not affect the difference in geometric preparation.

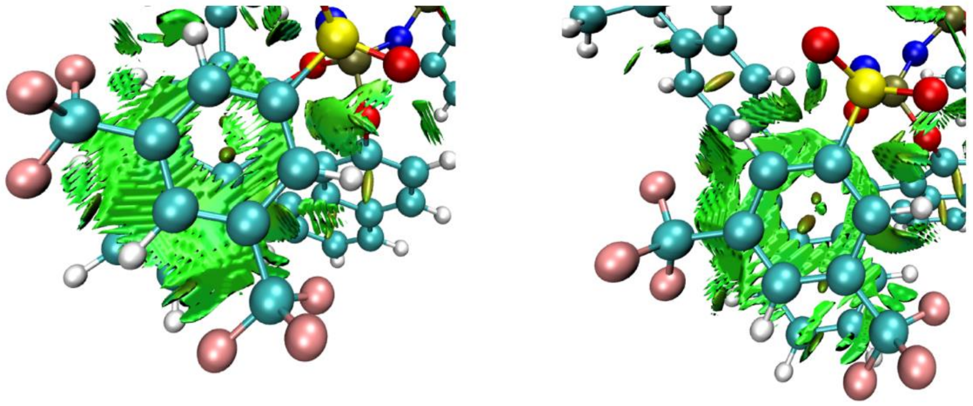

TS1-1 **TS1-1'**

Figure S6. Noncovalent interactions between the iminyl-Ph-$(CF_3)_2$ group with the residue (**3b_4**, see **Figure 5**). For both TS conformers π-π interactions with the phenyl-rings of the residue are present which is why the stereoselectivity is not significantly affected by the removal of this group.

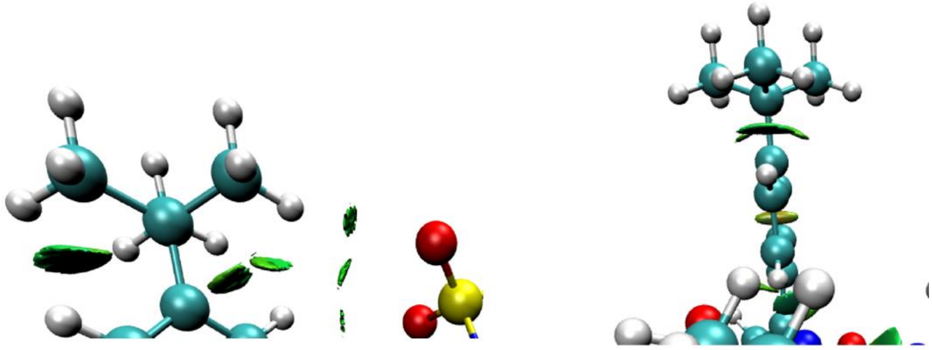

TS1-1 **TS1-1'**

Figure S7. Non-covalent interactions between the *t*-Bu group of the pocket forming substituent with the residue (**3b_5**, see **Figure 5**). For the major TS conformer **TS1-1** additional attractive interactions with the SO_2 group of the core catalyst are present. These stabilizing interactions are missing for the minor TS conformer **TS1-1'**. Removing this group therefore energetically favors **TS1-1'**.

The same analyses were performed for the second most stable minor transition state conformer (**TS1-2'**) which is only 0.3 kcal mol^{-1} less stable than **TS1-1'** and therefore should be significantly occupied as well.

Table S10. Geometric preparation energies for TS conformers **TS1-1** and **TS1-2'** obtained from B3LYP-D3/def2-TZVP calculations. During the calculations only the relaxation of the newly added hydrogen atoms was allowed. For definition of the structures **3b** to **3b_5** see **Figure 5** of the main manuscript. In order to obtain the geometric preparation of the catalyst backbone, the geometric preparation energies given below were obtained by subtracting the needed energy for the N-H bond elongation (30.2 kcal mol^{-1} for **TS1-1**, 31.3 kcal mol^{-1} for **TS1-2'** with D3BJ, 30.6 kcal mol^{-1} for **TS1-1**, 31.8 kcal mol^{-1} for **TS1-2'** without D3BJ) from the total geometric preparation energy. All energies are in kcal mol^{-1}.

	Geo-prep (with D3BJ)			Geo-prep (without D3BJ)		
	**TS1-1**	**TS1-2‘**	**TS1-2‘ - TS1-1**	**TS1-1**	**TS1-2‘**	**TS1-2‘ – TS1-1**
**3b**	12.5	18.5	6.0	4.4	10.8	6.4
**3b_1**	13.3	17.9	4.6	4.7	13.2	8.5
**3b_2**	13.6	16.3	2.7	4.8	10.6	5.8
**3b_3**	11.7	9.1	-2.6	3.4	9.0	5.6
**3b_4**	8.7	5.9	-2.8	2.8	7.5	4.7
**3b_5**	7.2	3.1	-4.1	2.3	6.2	3.9

As for **TS1-1’**, London dispersion has a large impact on the total geometric preparation. For **3b**, 65% for **TS1-1** and 42% for **TS1-2’** of geometric preparation stems from London dispersion.

Again, without the D3 dispersion correction, the difference in geometric preparation between **TS1-2’** and **TS1-1** almost remains constant for all six different structures. When dispersive interactions are taken into account, the difference in geometric preparation becomes smaller from step to step and starting from **3b_3** even becomes negative. This already hints to the great importance of dispersive interactions for the geometric preparation and for stereoselectivity as well.

For all involved groups that are replaced during the deconstruction procedure, we now determine the attractive interactions for **TS1-1** and **TS1-2’** by using NCI plots. In order to analyze the interactions of a particular group, we use the 3D structure of the subsystem before this group is removed.

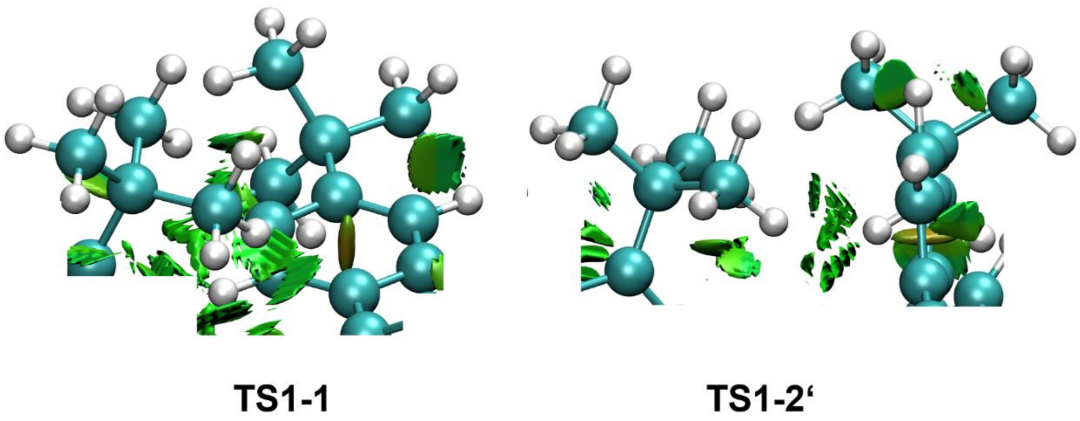

Figure S8. Noncovalent interactions of the *t*-Bu group of the BINOL aryl-substituent that does not form the catalyst pocket with the BINOL backbone (**3b_1**, see **Figure 5**). It can be seen, that for **TS1-1** additional dispersive interactions are present, which is why removal of this group leads do a relative stabilization of **TS1-2'**.

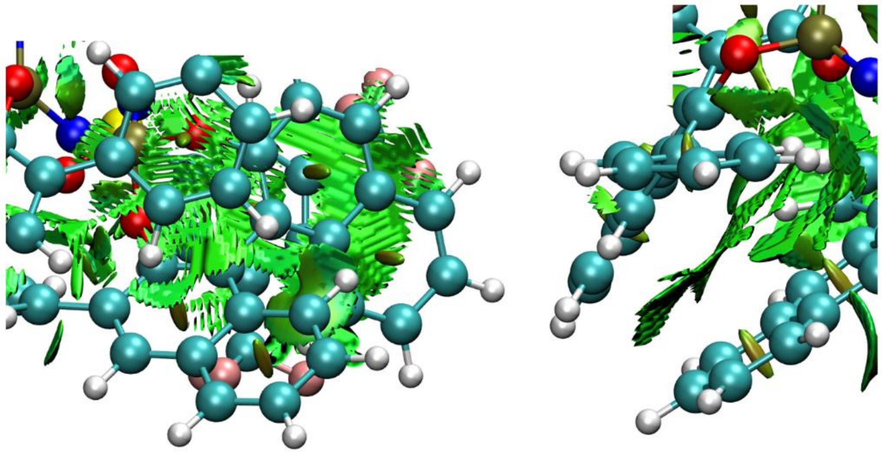

TS1-1 TS1-2'

Figure S9. Noncovalent Interactions of the Ph-ring bound to the BINOL substituent that is not forming the catalyst pocket (**3b_2**, see **Figure 5**). Due to geometric distortion, **TS1-1** forms stronger interactions with the catalyst's backbone. For **TS1-2'** the distance to the next aryl-ligand is too large to allow for dispersive interactions. For **TS1-1** more attractive interactions of this group are present. Its removal therefore energetically favors **TS1-2'**.

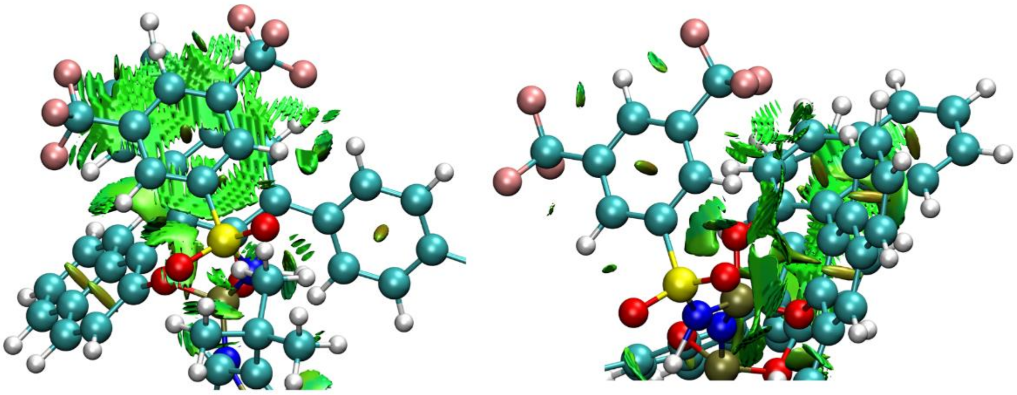

TS1-1 **TS1-2'**

Figure S10. Noncovalent interactions between the aminyl-Ph-(CF_3)$_2$ group and the residue (**3b_3**, see **Figure 5**). The aminyl-Ph-(CF_3)$_2$ group of **TS1-1** has strong attractive interactions with the phenyl-rings of the BINOL substituents. For **TS1-2'** the corresponding aminyl-Ph-(CF_3)$_2$ group has rotated and therefore does not allow for such interaction. Because of the strong interactions only present for **TS1-1**, the removal of this group has large influence on the stereoselectivity.

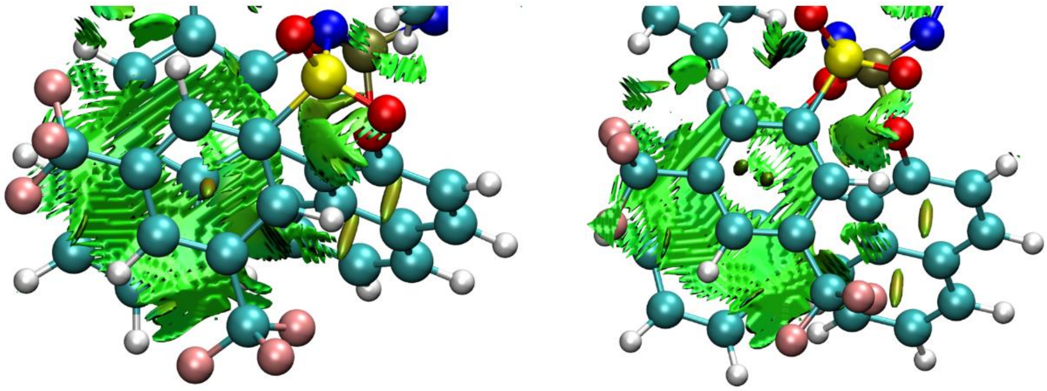

TS1-1 **TS1-2'**

Figure S11. Noncovalent interactions between the iminyl-Ph-(CF_3)$_2$-group with the residue (**3b_4**, see **Figure 5**). For both TS conformers π-π interactions with the phenyl-rings of the residue are present, which is why the stereoselectivity is not affected by the removal of this group.

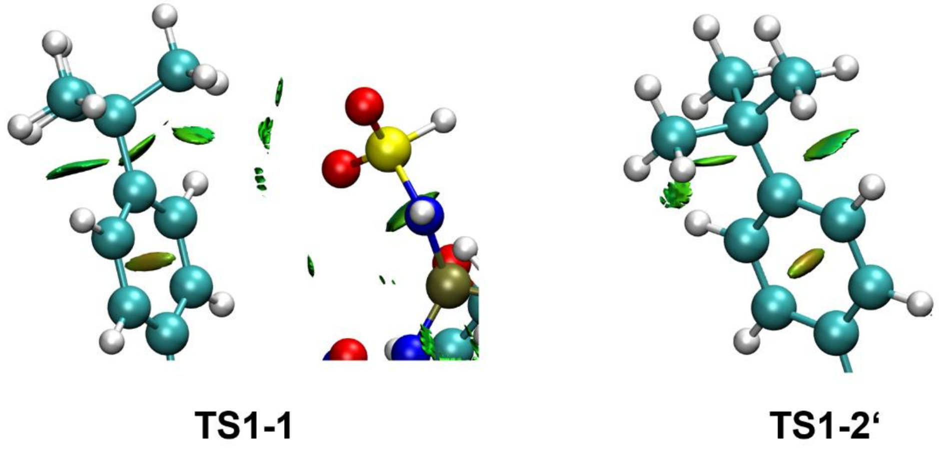

Figure S12. Non-covalent interactions between the *t*-Bu group of the pocket forming substituent with the residue (**3b_5**, see **Figure 5**). For the major TS conformer **TS1-1** additional attractive interactions with the SO_2 group of the core catalyst are present. These stabilizing interactions are missing for the minor TS conformer **TS1-2'**. Removing this group therefore energetically favors **TS1-2'**.

Literature

(1) Grimme, S.; Hansen, A.; Ehlert, S.; Mewes, J.-M. r2SCAN-3c: A "Swiss army knife" composite electronic-structure method. *The Journal of Chemical Physics* **2021**, *154* (6), 064103. DOI: 10.1063/5.0040021.
(2) Neese, F. Software update: The ORCA program system—Version 5.0. *WIREs Computational Molecular Science n/a* (n/a), e1606. DOI: https://doi.org/10.1002/wcms.1606.
(3) Altun, A.; Neese, F.; Bistoni, G. Extrapolation to the Limit of a Complete Pair Natural Orbital Space in Local Coupled-Cluster Calculations. *Journal of Chemical Theory and Computation* **2020**, *16* (10), 6142-6149. DOI: 10.1021/acs.jctc.0c00344.

Cartesian coordinates of important structures

```
26
1
  C   -1.01908820361449      0.79792049035890     -2.03193581732639
  C   -0.63634276459056     -0.09420080450966     -1.09654749265114
  C    0.72241556678069     -0.04520218819730     -0.50069642846183
  C    1.35227902864235      1.18549961891112     -0.24555952072227
  C    2.63633841505473      1.23845832462114      0.29172631758097
  C    3.32143821852200      0.05896852162420      0.59329976079004
  C    2.70524038049900     -1.17131024511474      0.35876819899496
  C    1.41729007005784     -1.22226721083652     -0.17373781677047
  H    0.95534506978284     -2.19331461826676     -0.35676478067466
  H    3.22927428484150     -2.09927256157846      0.59378130390326
  H    4.32519443843007      0.09914092717349      1.01893364496001
  H    3.09940575581407      2.20639432315137      0.49040206214662
  H    0.81098823677665      2.11171102236131     -0.44522421400723
  C   -1.58138806920529     -1.17447097828202     -0.61874071722792
  C   -1.98628240963467     -1.05608349859203      0.86211602396382
  C   -2.84316024197817      0.17268906098547      1.16758993677025
  H   -3.71724907054804      0.19100673869853      0.48466203173878
  H   -3.23370765254813      0.10800096616981      2.19395520289056
  O   -2.12724698727059      1.40857161933745      1.10733852932261
  H   -1.76072939309116      1.47579432412964      0.20454206809482
  H   -2.56219540119296     -1.95537093595829      1.13849162397643
  H   -1.08943387258415     -1.03607836704355      1.50107981180797
  H   -1.11203483596668     -2.15915743265567     -0.77861745111873
  H   -2.48617976197796     -1.15884306003720     -1.24677071316642
  H   -0.33619964206188      1.55792995170801     -2.41377998397549
  H   -2.02189819863705      0.76608638034176     -2.46154500673856
26
2
  O   -2.84895405365497     -0.03423062449586     -0.10568543421516
  C   -4.01210775499212      0.79588070692340     -0.05840820208392
  C   -3.53986037771518      2.26499775164735     -0.08445196696827
  C   -2.00351627176745      2.18348274618222      0.04238788576502
  C   -1.68459447120063      0.69956632337185      0.36153386977163
  C   -0.51641769415571      0.18558062767732     -0.47086615546020
  C   -1.50055656084385      0.46713072685882      1.86764482064725
  C   -2.43255247220355      0.98615372791866      2.78318937771398
  C   -2.30967113175948      0.75810217664513      4.15294922945978
  C   -1.24057494308566      0.00831542485200      4.64877535036976
  C   -0.30078486541615     -0.50488547586213      3.75622560399522
  C   -0.43286405722948     -0.27840527809550      2.38358047116309
  H   -4.60312960196440      0.57440755877063      0.84888922657190
  H   -4.62730980009245      0.53002445015080     -0.93121669801515
  H   -3.99273222257856      2.84121330835427      0.73391302295850
  H   -3.82751258192139      2.75880700523638     -1.02179878647783
  H   -1.61226414649203      2.85456434077096      0.81707510418719
  H   -1.52119495490920      2.46155450720657     -0.90446183051762
  H   -0.74464478672589      0.34997602899048     -1.53200042468694
  H   -0.36080943347378     -0.89076907098951     -0.32145140887308
  H    0.41316948803505      0.71722326172107     -0.22360868840393
  H   -3.27057238597264      1.58776059527091      2.43140809331532
  H   -3.05297154186007      1.17193857145994      4.83683766030294
  H   -1.13970839310485     -0.16858270073609      5.72066295745823
  H    0.54441994145970     -1.08816942595486      4.12618479758095
  H    0.31851507362480     -0.69430726387484      1.71300212444131
```

202
3b

H	6.67640427518166	-0.86627629891181	4.46175548580739
C	5.63455179041630	-1.08231214089813	4.18427400712352
C	5.60901795912090	-2.15847070219067	3.08998358523203
C	4.18345668990326	-2.51115995519065	2.65123999430598
C	3.04296192364119	-1.95603304373837	3.24535026804180
C	1.75719391830104	-2.34314384126744	2.86734020083900
C	1.55677618690678	-3.30942798690960	1.87145410303887
C	2.69792531850300	-3.84504269189816	1.24852615912467
C	3.97581292297224	-3.45696167081602	1.63289467904927
H	4.82808136772308	-3.90209693233284	1.11756953923994
H	2.57998230216739	-4.56028854171420	0.43310196083483
C	0.21411365181658	-3.80058303747282	1.49078233828594
C	0.00385697357298	-5.13986140838379	1.19957606108571
C	-1.25097749168725	-5.63644951994662	0.77498305630122
C	-1.41871780848215	-7.00242974857612	0.42254798397431
C	-2.62642923521358	-7.47150602887634	-0.04642408666677
C	-3.72117149893765	-6.58703693557738	-0.18108491969173
C	-3.59716704355485	-5.25799791340160	0.16883039031667
C	-2.37045858527947	-4.74485397651327	0.66550251860405
C	-2.18319742827530	-3.36683101291706	1.00670072649713
C	-0.91758457940954	-2.94597411782502	1.37971166024476
O	-0.75090123808282	-1.58187934729226	1.64662556534826
P	-0.68401622720473	-0.59113275144918	0.36668181575390
N	-0.93474259499548	0.90380664743076	0.82917467898743
S	-0.65496148948796	1.46620023463561	2.31422094989079
O	-0.73287475528452	2.92171449397702	2.24803747914282
O	0.51071674065739	0.85799807851551	2.95700778642758
C	-2.09426540946579	0.90075973954233	3.24050483227379
C	-1.97207177870529	-0.19688985635132	4.08716152662494
C	-3.10502754448502	-0.68643761643296	4.74160856769154
C	-4.34557619234706	-0.07403092734366	4.56953090621642
C	-4.44295410595381	1.04568000091162	3.73975778113035
C	-3.32272724058893	1.53683608787993	3.06690560426481
H	-3.39684007829491	2.40640165733873	2.41580595266798
C	-5.75376897597242	1.77554866933189	3.59659344044783
F	-6.02273146084862	2.09390446516907	2.29623696734240
F	-6.80789457009859	1.05245124751141	4.05672965784560
F	-5.74567719025063	2.95142232068021	4.28669922040360
H	-5.22807414672847	-0.46736387636704	5.06947599937911
C	-2.94618607025976	-1.87880135524876	5.64913051826240
F	-2.32245186276945	-2.91385025006768	5.01484790132545
F	-4.13308695958034	-2.34638081365098	6.11354945088479
F	-2.18073040513321	-1.57693531140258	6.73760930741473
H	-0.99795943187097	-0.66431747771075	4.22044429688961
N	0.59804201384849	-0.97145997433411	-0.49359906618997
P	1.81167837512537	-0.24307783960472	-1.10150161884499
N	1.75216223448835	1.41528884729686	-1.30399878739679
S	0.95367343289511	2.16173766313114	-2.59887578916961
O	-0.13264583492557	1.26476538965888	-2.93608426897107
O	1.94283029405554	2.56892403862413	-3.57842792729971
C	0.33269476504904	3.65563684794681	-1.83732181776459
C	-0.82238877118294	3.63976898622264	-1.04691160289841
C	-1.28555997482745	4.85707606686595	-0.54989198738826
C	-0.61206996376892	6.05265667051491	-0.82377833076528
C	0.53838811690024	6.03751056681775	-1.60443499708941
C	1.01761569140536	4.83291947478991	-2.12356828042918

H	1.90293498020178	4.80440460838376	-2.75767243125589
C	1.30150253445267	7.30410026787660	-1.89816536067685
F	0.69569041153742	8.40450524578868	-1.39013387382655
F	2.56096428676929	7.25491411365997	-1.36740440651165
F	1.45235362532947	7.49899268866471	-3.23602403527956
H	-0.98633397837469	6.99272483266579	-0.42033898497540
C	-2.54070003699297	4.95646092173202	0.28420745786210
F	-2.30560968817251	5.58442968405919	1.46547446188241
F	-3.09592210090786	3.75817608952038	0.55030117061943
F	-3.48714678128959	5.70546584126865	-0.36816682426543
H	-1.33501053346996	2.70473362612141	-0.81918209889340
H	2.56957858634138	1.93409006546517	-0.94893648520472
O	2.20455135004985	-0.86058146226977	-2.53797824499448
C	2.71400596387383	-2.18008852909264	-2.46266262822210
C	4.04333716217308	-2.32257454459632	-2.08784312392027
C	4.57380526212481	-3.64340271671687	-1.93364911399349
C	5.88490154337848	-3.89941038478616	-1.45339536757908
C	6.34145724735761	-5.19102995758131	-1.29078757600474
C	5.51136243737688	-6.29221814820078	-1.60360993381116
C	4.22883818688771	-6.07786154569643	-2.05821401848436
C	3.72150013980500	-4.76054348168029	-2.22046669236850
C	2.38507845647726	-4.53626986000924	-2.62187159651239
C	1.83753089159234	-3.26589388068076	-2.73056387935719
C	0.40790743479966	-3.10507891550904	-3.07292921502645
C	-0.06046921006600	-2.14470547696191	-3.97947372581773
C	-1.40744917819939	-2.10687443085466	-4.34480811850445
C	-2.34065004014646	-3.01112558200102	-3.81932348618704
C	-1.86977497819201	-3.93845731942146	-2.87473529060346
C	-0.53112881598970	-3.98603239741936	-2.50874506343376
H	-0.20879181981518	-4.70338365366567	-1.75258086876848
H	-2.55932456602207	-4.64239652855740	-2.40653228629165
C	-3.80758816720576	-3.05385731328542	-4.26135146653284
C	-4.15823048579024	-1.92575419283805	-5.24162682974293
H	-5.21733412831513	-2.00555740448923	-5.52636075506677
H	-3.56168496201346	-1.98651073577037	-6.16364764592870
H	-4.00952254230887	-0.93356150266808	-4.79239165603894
C	-4.73779169063514	-2.94231780955279	-3.03705859569002
H	-5.78778847364134	-3.02669635044198	-3.35542806978037
H	-4.61418698112227	-1.97658608449050	-2.53023951303770
H	-4.54296879686441	-3.73720795909298	-2.30454280027036
C	-4.05947332148719	-4.40670066867089	-4.96149551716720
H	-5.10644788352080	-4.47159976153251	-5.29492623095808
H	-3.86389143574593	-5.25014570495806	-4.28455123650765
H	-3.40890711697649	-4.51992666077560	-5.84086183977227
H	-1.72381633842993	-1.35446814399334	-5.06681741065556
H	0.62996207187300	-1.42760956643494	-4.42204836774157
H	1.75479608092521	-5.39902602718510	-2.84528143309303
H	3.57261397963954	-6.91954434268335	-2.28937944306173
H	5.88614281062551	-7.30861960894446	-1.47566300386209
H	7.35029943723662	-5.36349448528614	-0.91294121349206
H	6.53258744521084	-3.06034571122057	-1.20375076541385
C	4.84322035054308	-1.10947582990526	-1.78278733753935
C	4.36894187949865	-0.19738542646949	-0.85308712169654
C	5.02716176244406	1.01551758184944	-0.51570242608061
C	6.24495760368034	1.25894692472227	-1.12899214421168
C	6.78779612610889	0.38108203200966	-2.09557724333198
C	8.01576133000180	0.67437351833234	-2.74621772309432
C	8.49932166723468	-0.14133018444075	-3.74511991913928

C	7.76274843282562	-1.28032896806432	-4.14385639680751
C	6.57635903195344	-1.60422976620455	-3.51934423673164
C	6.06734876622926	-0.80502633607621	-2.46268401970986
H	6.01408375252009	-2.48044209719690	-3.83954081448026
H	8.13314368976506	-1.90620052610518	-4.95698598873236
H	9.44039410297410	0.09815246491567	-4.24203636728060
H	8.56146227494463	1.57172279165278	-2.44752147135489
H	6.79533690220271	2.16643511772973	-0.87297713755335
C	4.39842866907937	2.01494813668385	0.37809521932424
C	3.78359317395107	1.66780000796433	1.59158098031870
C	3.18859289988086	2.63857409185003	2.39446156975953
C	3.16945017127193	3.99032171916047	2.02519485743022
C	3.76858597845641	4.33098319533319	0.80008912050126
C	4.37342540707408	3.37069541773467	-0.00671945550906
H	4.81603710753064	3.66401652427099	-0.96134357315702
H	3.75808590897334	5.36763175662052	0.45896613918179
C	2.52661443964572	5.07372014928069	2.89489454855772
C	1.92415695244125	4.49951332643431	4.18462420119580
H	1.12889182188865	3.77331861679781	3.96824717373924
H	1.48180742696221	5.31671103848284	4.77237457075529
H	2.68827374330687	4.01531507869457	4.81062870911758
C	3.60037296098863	6.11633668166550	3.26809042196237
H	4.03366905793791	6.59202023467076	2.37694728007224
H	4.41849098901131	5.65147238522396	3.83745767185879
H	3.15472153398049	6.90725933035893	3.88970602187325
C	1.39861444075676	5.75802378925350	2.09723423716322
H	1.78179745068340	6.22556966856681	1.17979184855634
H	0.93234696618677	6.54614151333373	2.70752762860490
H	0.62355045776693	5.02840770761987	1.82892961830247
H	2.70195059009564	2.31523083491111	3.31195568714249
H	3.75503639584456	0.62660555277922	1.91117476249697
O	3.13486668117572	-0.46196125148023	-0.21738318479445
O	-1.88953797114062	-1.10326596481892	-0.58293507061384
C	-3.13794723015287	-1.28838867933806	0.02786862925653
C	-3.28483967547049	-2.37624989978901	0.87928633479153
C	-4.50320151533268	-2.50726961046730	1.61734237194128
C	-4.68084049169302	-3.47850590999069	2.63581002602844
C	-5.85993514031734	-3.54590333845996	3.34896989732233
C	-6.92161592103465	-2.65480130383152	3.06965065765003
C	-6.77715625147170	-1.69605163460638	2.09031451322781
C	-5.56816687517617	-1.58425855614810	1.35374980503851
C	-5.39176532839051	-0.57662207553517	0.37926765714235
C	-4.18919607111694	-0.37806254194474	-0.28381932964433
C	-4.05546750976004	0.70639791651442	-1.28256898780961
C	-3.26420365397993	0.57659455559539	-2.43856237028565
C	-3.21494581282111	1.59118593494832	-3.38690123835519
C	-3.93565005839917	2.78637416247794	-3.23811116431109
C	-4.72109743916608	2.91275042057300	-2.08503930573006
C	-4.77977338481125	1.89883185189659	-1.13001121954193
H	-5.37323249134423	2.05755987671244	-0.22901013126934
H	-5.28611418163395	3.82624747891735	-1.90384415722465
C	-3.83479103486783	3.87791664260864	-4.30791559240816
C	-2.36741584621127	4.33714584520617	-4.43633784981156
H	-2.02132697694053	4.79851077840541	-3.50070990080363
H	-2.27213912685487	5.08281791485608	-5.24002671742335
H	-1.69662564170335	3.49859776830342	-4.66889268145096
C	-4.30711185264207	3.30215786356621	-5.65894979234969
H	-5.35117972464866	2.96320138216508	-5.59470523797071

H	-3.69271860471160	2.44640482541051	-5.97094374739030
H	-4.24092917876395	4.07100807031215	-6.44362498383180
C	-4.69626847851429	5.10301890882481	-3.97289262802197
H	-4.59045166288231	5.85511295368061	-4.76808367948156
H	-4.38849210716065	5.57071821907416	-3.02653649187918
H	-5.76228570486365	4.84271772324614	-3.89927330613959
H	-2.57613136652912	1.44568745866779	-4.25898697178424
H	-2.67630882364205	-0.32323185768055	-2.60376049089760
H	-6.23702942105628	0.07337610285921	0.14974016185981
H	-7.58139665234032	-0.98773141850202	1.88250872055572
H	-7.84941667668197	-2.71999293657770	3.63982936213797
H	-5.96823587371964	-4.28471008430594	4.14411630293311
H	-3.86203266541436	-4.15827529323868	2.86884560973653
H	-4.44671325097761	-4.58555039501345	0.05819928127596
H	-4.67199777785327	-6.95708956522399	-0.56761275416455
H	-2.73900927565903	-8.52143062906943	-0.32036445567215
H	-0.56223369382101	-7.67307752479708	0.52027274772854
H	0.83585554636791	-5.83991492130204	1.29774254113846
H	0.90538331352453	-1.88468713458302	3.36430909056794
H	3.14038122860886	-1.20770358198146	4.03126584031522
C	6.42257954986919	-1.64450563548620	1.88623921855490
H	5.99639299334521	-0.71445890167388	1.48663948602177
H	7.45992765787758	-1.43945221172378	2.19118631298427
H	6.44880212861455	-2.38052873190318	1.07155615461040
C	6.27980397737577	-3.43399530763408	3.64255391789974
H	5.72121387347040	-3.82748389795154	4.50396480730151
H	7.30777578224690	-3.21292618336233	3.96814983017558
H	6.32582577479584	-4.22420796817391	2.88015201151505
H	5.17965173266795	-0.14073200503903	3.84351055568908
H	5.10917661794891	-1.41106420179934	5.09246572967950

176

3a

H	-4.19374944727435	1.40755845754065	5.74337365091787
C	-4.76297415812402	1.38159471521984	4.80287524559929
H	-4.62479158463767	0.39469062957896	4.33880381202047
H	-5.82807435813883	1.48252728119777	5.05683868872278
C	-4.35302523386681	2.52049029000729	3.85874853088389
C	-2.87483687837751	2.44658455196743	3.45941866211533
C	-1.98171132905085	1.50746698909577	3.99362355787339
C	-0.62302572258802	1.51617386564291	3.67033551704947
C	-0.10040863243890	2.47972427128497	2.79608981258419
C	-0.99985940011680	3.39691006090544	2.22409768660156
C	-2.34989543526815	3.38019941825554	2.55027196693777
H	-3.00526861800012	4.11464353454958	2.07962282184825
H	-0.63582178285840	4.12138375414495	1.49391975857992
C	1.34116377891059	2.59948036899705	2.48426744597002
C	1.92690907855997	3.85260035362881	2.38195700600952
C	3.27403014886891	4.03359363629712	1.99379372268171
C	3.82382868534781	5.33415761849432	1.83462731114623
C	5.11439551270809	5.50853787924504	1.38543328056707
C	5.90939119440313	4.38127553173246	1.07481685087387
C	5.41156364282016	3.10468075158079	1.23635828782379
C	4.09154325458319	2.88878898157229	1.71309318787221
C	3.52128669766541	1.58379162050794	1.86815639623471
C	2.18535308225660	1.48234926157456	2.23274882171106
O	1.63488630508418	0.19095731989603	2.32125764125345
P	1.25626838492478	-0.49157077686960	0.90377308558916
N	0.10444000786271	0.37767398854687	0.24322196865865

P	-1.14403778607825	0.12626485537711	-0.63599643259458
N	-1.43681247233792	-1.43444272889494	-1.16979788647255
H	-1.41557900493857	-2.15781032285772	-0.38877244313860
S	-1.17710479048908	-2.10288579410003	-2.67115768100826
O	-0.31141754889137	-1.27438143037445	-3.47770640740959
O	-0.96173659460519	-3.51526103616315	-2.46314304768834
C	-2.89458536607165	-1.95402099357041	-3.43575302237194
F	-3.79215424055753	-2.61521348928408	-2.68824378269475
F	-3.26205003228141	-0.66651671013234	-3.53517130705233
F	-2.85113557316565	-2.49886925803907	-4.66379616244139
O	-1.21399523230931	0.99326513124149	-1.97603259725407
C	-1.34019457874213	2.38510274262896	-1.73330972112381
C	-2.59326902902204	2.85195588549713	-1.37329912992373
C	-2.72488271448620	4.23496121449208	-1.02449784197487
C	-3.93324226193012	4.79312458081379	-0.53180094301659
C	-4.00364523639460	6.12241006913085	-0.16917089712903
C	-2.87141032717979	6.96111899846967	-0.28670025918166
C	-1.68029107855899	6.44690762731406	-0.75002625001114
C	-1.56752906985393	5.07834714920248	-1.11464714143112
C	-0.33101811876733	4.53262943197864	-1.53087609911567
C	-0.17254088303138	3.18754413934343	-1.82917847590634
C	1.15303212907080	2.64551109326058	-2.19545211376404
C	1.32901583641969	1.71460596018895	-3.22874669574243
C	2.60602799996896	1.28941737968702	-3.59660710453524
C	3.75567413013582	1.76737610994194	-2.95325409596628
C	3.56685943874892	2.67453262282136	-1.89705791712666
C	2.29927193535857	3.10281088690656	-1.52291381530790
H	2.19222064579058	3.78431585799015	-0.67742987791181
H	4.42537055831934	3.05635563539152	-1.34315760621162
C	5.17445469084631	1.38031556586298	-3.38444072904090
C	5.17978526254711	0.30917211967306	-4.48416551167510
H	4.69267471294934	-0.61898290015270	-4.15157383718576
H	6.21817185093478	0.06334457009172	-4.74942515151956
H	4.67835668805270	0.65863591495798	-5.39838290687785
C	5.97169363744359	0.84147190210500	-2.18068091252379
H	5.51937092616954	-0.07568408298476	-1.78170364534216
H	6.02421899630946	1.57500346269374	-1.36495348031957
H	7.00163956807014	0.60645497901689	-2.48857120634148
C	5.87430239052693	2.64556501232821	-3.92534548850879
H	5.93004953540496	3.42958491670545	-3.15699675063395
H	5.33059864126973	3.05558948474982	-4.78878792277680
H	6.89994459817116	2.40513377440436	-4.24442632829348
H	2.69313294484297	0.57328782939830	-4.41316883022224
H	0.46695512074983	1.32163218632069	-3.76567823917107
H	0.53339524725978	5.19441727737438	-1.61119009023045
H	-0.79514142607621	7.08116851472106	-0.83204071000819
H	-2.94196796015956	8.01178535955711	-0.00196329106792
H	-4.94046590986366	6.52816342074490	0.21549157433814
H	-4.81044754584762	4.15548039464804	-0.43219764208793
C	-3.73364130259420	1.90567857900383	-1.26425950789906
C	-3.63345046676317	0.80266235123855	-0.42982173930828
C	-4.68956496717728	-0.11612832934448	-0.18411620170119
C	-5.87434161463795	0.11199648102194	-0.86822519208162
C	-6.02583969823101	1.17004124267525	-1.79335868298449
C	-7.23252490838785	1.34597229491928	-2.52180775496134
C	-7.35835743825215	2.35648607503058	-3.44960141300640
C	-6.27464067953227	3.23166124876102	-3.69267048292164
C	-5.09491845561553	3.09964591172545	-2.99008345884708

C	-4.94093993922488	2.08310154566725	-2.01212104596149
H	-4.26302395750995	3.77484464651188	-3.18853872300259
H	-6.37057262073914	4.01503938781678	-4.44581790339226
H	-8.28815710230844	2.47631615893401	-4.00740149793690
H	-8.05637193572780	0.65299367650768	-2.33901072738375
H	-6.72359627412057	-0.54919652424820	-0.68638051128549
C	-4.55058211793122	-1.25784429193646	0.74412859553452
C	-5.13340684920027	-2.49682172243182	0.42489658110671
C	-5.03106123828610	-3.57845096011906	1.29115131341443
C	-4.34388480545584	-3.48206024482609	2.51282363382594
C	-3.78087720018580	-2.24131926934772	2.83203598029659
C	-3.87455968936545	-1.15227101239507	1.96849088021779
H	-3.40386026057007	-0.21315724477116	2.25502354995474
H	-3.21840704707636	-2.11716692905260	3.75604456008283
C	-4.21221643288601	-4.70959289799996	3.41711421583186
C	-3.56688937541831	-5.86004688539595	2.61675231652413
H	-3.43469690752883	-6.74029113231427	3.26383152527629
H	-4.18960728014253	-6.16151834049330	1.76313773275973
H	-2.58372632359159	-5.56192558681734	2.22968417341776
C	-3.33896246452464	-4.42874099422913	4.64822395626108
H	-3.77663009824711	-3.64779779022861	5.28764923991224
H	-2.32417706286109	-4.11518953172286	4.36472786417993
H	-3.25510357055706	-5.34337578001842	5.25282157542716
C	-5.61510969451529	-5.13960836083620	3.89205061750520
H	-6.09325936940674	-4.33942414330130	4.47573182931343
H	-6.27215134295405	-5.37916225481856	3.04399557478493
H	-5.54260729245799	-6.03458207968167	4.52862460775680
H	-5.47953046991516	-4.52818270211721	0.99475564306181
H	-5.64173977978109	-2.62054448724773	-0.53293305782731
O	-2.40991005239324	0.58535572755145	0.23385177151220
N	1.09411360924004	-2.05578516361645	1.08640933148842
S	-0.04855664189035	-2.84737446313045	1.83348361324289
O	-0.29835874848826	-2.46549987005920	3.21458814164159
O	-1.23371978645397	-3.06727668250523	0.97798114717426
C	0.78094074487402	-4.53297259947104	1.88923675287257
F	1.97276476338390	-4.45427978342155	2.50722639716675
F	-0.01178891176680	-5.37622615731661	2.58444966098487
F	0.95606860118084	-5.02237486207321	0.65199528430962
O	2.56105489731279	-0.22453911199301	0.00091597650436
C	3.80186163479980	-0.52182439420735	0.58920193323070
C	4.29056664087333	0.35343191736124	1.54845064466594
C	5.51574014733434	0.02058276627517	2.21030938655322
C	6.03090326243295	0.77376224290011	3.29710937145874
C	7.21778607730747	0.41969348665008	3.90460840682718
C	7.94847585981598	-0.70412434506355	3.45487966491818
C	7.46116824086833	-1.47191178095051	2.41993528904688
C	6.23316703755493	-1.14672132767048	1.78441126054992
C	5.69902440464900	-1.96142292936970	0.75952766274575
C	4.47674179716854	-1.69734268197851	0.16091374570321
C	3.90749947843552	-2.61427118828206	-0.84945570369948
C	4.03042866134690	-4.00294070809509	-0.67401243656190
C	3.50901347188290	-4.89119556529796	-1.60667826006809
C	2.83955007897031	-4.44338482942444	-2.75743414010525
C	2.72684157649241	-3.05804162329976	-2.93075856143427
C	3.24481864387604	-2.15922932796938	-1.99892024303883
H	3.12374761473307	-1.09108920649547	-2.17348292302846
H	2.20885556023270	-2.65481142506688	-3.79993277955158
C	2.25555836823629	-5.45776440498580	-3.74424833753327

C	1.55697264500985	-4.77862113035205	-4.93046790515244
H	1.16038813963960	-5.54729601645744	-5.60953893394358
H	0.71417209146620	-4.15589489862157	-4.60069424575890
H	2.25188103704686	-4.15028562526031	-5.50704063750999
C	3.39220207240075	-6.34599366666704	-4.29036191405158
H	3.90669246070433	-6.88774389035639	-3.48451227615846
H	4.14055410785528	-5.74097729676888	-4.82294211411997
H	2.98692044694337	-7.09015831520597	-4.99276762974466
C	1.22218913423992	-6.33810397295038	-3.01010507317957
H	1.67925890512863	-6.88620493368501	-2.17454020742148
H	0.40456524315297	-5.72446912562551	-2.60887883112324
H	0.79520827337714	-7.07690291452392	-3.70550832296302
H	3.60680081436150	-5.96182011492568	-1.41879020860108
H	4.50417793896256	-4.38858113732773	0.23052755715292
H	6.26868457772053	-2.83442078637273	0.43520292960799
H	8.00505054576105	-2.35541270390247	2.07910649371640
H	8.88936616983257	-0.96934504188056	3.93916776201316
H	7.59257407882656	1.00840985290548	4.74319330320437
H	5.47136111648202	1.63754192686130	3.65501130523668
H	6.03365299597281	2.24547271076668	0.99045810865231
H	6.92477637203325	4.52108819287031	0.70079045897105
H	5.52202965543368	6.51264565960457	1.26026645487145
H	3.19344645264644	6.19611530964100	2.06376672507481
H	1.32292326434449	4.73597870674462	2.59787476343683
H	0.03292192771505	0.77093277269000	4.11862265409825
H	-2.33788112430739	0.74977947465011	4.69132412520482
C	-5.24954509843433	2.46228225372232	2.60578330312493
H	-5.01336921950613	3.27006802163601	1.89986871887560
H	-5.13562136466142	1.50646529621280	2.07773555777954
H	-6.30618240059669	2.56553569604016	2.89530602064395
C	-4.59011946843294	3.86385350670302	4.58181768571884
H	-4.35049349859199	4.71630401543404	3.93074972716077
H	-3.96382391468734	3.93927105654192	5.48247852417224
H	-5.64463921444487	3.95083923647399	4.88521275325946

128
3d

C	-2.14736307521752	1.12796960777195	4.55995744472603
C	-2.21269080079390	2.12502885596579	3.58428009071708
C	-1.07580798599039	2.46333208520452	2.85421170012653
C	0.14959176933863	1.81282830848285	3.07861502962461
C	0.20334149161172	0.81085947023487	4.06303503155070
C	-0.93486968016321	0.47508241691702	4.79468422781194
H	-0.87169365246691	-0.30463529167820	5.55532556900312
H	1.14075034862405	0.29431162415116	4.26176105144241
C	1.33363904800822	2.22117389934415	2.28675970990528
C	1.55235604754892	3.55673154926424	1.98951001621622
C	2.62022639810615	3.98284419325918	1.16789515177818
C	2.78362565592574	5.35352784320246	0.83278880506989
C	3.77922817390481	5.75954542490719	-0.02875593548678
C	4.65558550585459	4.80289779679324	-0.59145415071662
C	4.54122913416043	3.46750490172734	-0.26406826809246
C	3.53693523483747	3.01725571232473	0.63402303153407
C	3.36557721677279	1.63696916796850	0.98120053505343
C	2.27305449923316	1.28692961392235	1.76439238156885
O	2.08942490687409	-0.06826300182134	2.06589623744996
P	1.50603886029539	-1.01723554918038	0.89350404679855
N	0.02191919041945	-0.59379570980515	0.57446467658240
P	-1.28749586137285	-0.59157834204322	-0.23196329277926

N	-1.65787715165737	-2.11960550941528	-0.77184598491368
S	-2.92043895889532	-2.64159290794023	-1.71846926507902
O	-3.35243613067050	-3.92901298841977	-1.23328286910051
O	-3.81111665655446	-1.53300315311173	-1.97613761505945
C	-2.02478826683265	-3.00165615830200	-3.33775133367045
F	-1.46513145842163	-1.88356810927703	-3.83411682775114
F	-1.06811573957576	-3.92086791068388	-3.13855881074026
F	-2.92271525176824	-3.47275829091812	-4.21878327864744
H	-1.04857763081249	-2.87567043231505	-0.34732591620570
O	-1.39494687752972	0.30202904790224	-1.56319250596683
C	-1.26346020152609	1.69952889031018	-1.37416248879911
C	-2.28961249907744	2.37856052580277	-0.73609087259194
C	-2.06646331866870	3.74982643008180	-0.37882211683857
C	-2.97720024605211	4.48800157008935	0.42153057220774
C	-2.70258373870577	5.78925009453554	0.78887362463227
C	-1.51323218508150	6.42270882934065	0.36013414885400
C	-0.60643379913144	5.73120212160580	-0.41322788875153
C	-0.84528804031486	4.38162495876528	-0.78452765964383
C	0.09576031889061	3.65650473086667	-1.55041673237321
C	-0.07779971576906	2.31772523643831	-1.86124245774158
C	0.90504660631874	1.61629691434620	-2.71760458853063
C	2.27644616141254	1.87445974232400	-2.56702911462508
C	3.21132734113429	1.29475603213094	-3.42149958295244
C	2.79149278593989	0.45012183267807	-4.45150842918844
C	1.42898799172789	0.18577330329642	-4.61198865608033
C	0.49348019531754	0.75873365282812	-3.75190001949456
H	-0.56660769860945	0.55675557989242	-3.89807450103993
H	1.08988862058644	-0.47236314030198	-5.41355167338153
H	3.52215231116397	-0.01106222187036	-5.11734777154980
H	4.27377993345555	1.49501954997118	-3.27337357270384
H	2.61351681334378	2.52086415164294	-1.75787317520139
H	0.98624903978131	4.17370327387950	-1.91177046681097
H	0.32207575434232	6.20134146653281	-0.74215297062084
H	-1.31496181371143	7.45626131492688	0.64775562297676
H	-3.40912223256714	6.33336681437081	1.41717145683955
H	-3.89451206975998	4.00753050060412	0.75974231666510
C	-3.57422274331830	1.70090170551657	-0.42704373725773
C	-3.60104833504702	0.53906779586782	0.32414528692643
C	-4.78807368213070	-0.14174835033189	0.70417207260735
C	-5.98328129585977	0.39971366318418	0.26547255551480
C	-6.03451939053108	1.54705150042504	-0.55994139586178
C	-7.27176591284573	2.04811963651689	-1.04398675014182
C	-7.31389740195423	3.13396169027100	-1.89074454975094
C	-6.11184726868336	3.75680806392728	-2.29604176553613
C	-4.89405732739986	3.30551794999675	-1.83016731791336
C	-4.81705452259792	2.20620073834429	-0.93606426263227
H	-3.97578689751131	3.79054866760868	-2.15846887744130
H	-6.14575692873272	4.59878717864867	-2.98897639281245
H	-8.27040507492245	3.50479620406131	-2.26159359823471
H	-8.19109214174778	1.54273067563288	-0.74108919286007
H	-6.91900923227582	-0.08353830555050	0.55312209704948
C	-4.75988344733384	-1.39875216517631	1.48539574294483
C	-3.97001677225432	-1.54152317215489	2.63849378834076
C	-3.98172104012376	-2.73497158291581	3.35750265646588
C	-4.76944555141404	-3.80771125640775	2.93425449630031
C	-5.55301459538274	-3.67798573808509	1.78645675149723
C	-5.55050433086177	-2.48284095356643	1.06946316391563
H	-6.13182387030671	-2.39607060631905	0.15027906751901

H	-6.15390805491283	-4.51754734886301	1.43438152334522
H	-4.76129809150326	-4.74649643142994	3.48988179380251
H	-3.35906561658487	-2.83071303207446	4.24811914135952
H	-3.34366130645369	-0.71550116767223	2.97397162024869
O	-2.38575047539357	-0.00178387585953	0.76873325320842
N	1.88800501270709	-2.46437204962466	1.41734394654902
S	1.09437747053856	-3.82285177136147	1.30819910480757
O	0.08999839106354	-3.88011536451469	0.22872060209266
O	1.98489521680640	-4.95306013560558	1.44638940458215
C	0.05779213929599	-3.80750536211321	2.87827384563164
F	0.84563602674799	-3.77120801867990	3.96846472509077
F	-0.71245140168690	-4.90908101270128	2.93217887313310
F	-0.74576802966476	-2.71738588574457	2.89545093089576
O	2.36580855852111	-0.56289029101380	-0.39882996873273
C	3.75729292162449	-0.50121327272510	-0.22347399458267
C	4.27913340581045	0.57790876767459	0.47671251310822
C	5.68921515054683	0.59906497930861	0.73307888070162
C	6.30799246433025	1.57907987237377	1.55253450920710
C	7.66939527445553	1.56143224587000	1.77516025800422
C	8.48131462890735	0.56288821514331	1.19060361701288
C	7.90761155159133	-0.42112012752193	0.41560273639627
C	6.50713307519325	-0.44347969989080	0.18082145296400
C	5.90622212098832	-1.49010133307155	-0.55828207777601
C	4.53798757284213	-1.56193599190002	-0.75569214164419
C	3.91504951356124	-2.71709355999695	-1.44110150745383
C	2.94185681140650	-2.54547070209069	-2.43918808854183
C	2.36997739099871	-3.65083596426938	-3.06483031839925
C	2.74688840320905	-4.94424405017326	-2.69606974097746
C	3.70923366077707	-5.12498416473086	-1.70182482840137
C	4.29210847084451	-4.02058248631267	-1.08223831057761
H	5.00987291005889	-4.16573543981933	-0.27357236635182
H	3.98896683255062	-6.13041965712951	-1.38527494631195
H	2.27983464735877	-5.80847413540155	-3.17051943289705
H	1.61576113254807	-3.49915731132582	-3.83857017637480
H	2.63730991375699	-1.54215697417303	-2.73102237169220
H	6.54253790837638	-2.27579177928752	-0.97077695802138
H	8.51994031353901	-1.21340692289537	-0.02006899098319
H	9.55752581835251	0.56389895205155	1.36900788936792
H	8.12189893746875	2.32128289518645	2.41405765846705
H	5.69202250816358	2.34812919181758	2.01710950651028
H	5.22245558089899	2.74011879587937	-0.70389602457776
H	5.42972463557321	5.12296752868639	-1.29032661014316
H	3.89012172386876	6.81452747941167	-0.28378834904694
H	2.08943842361568	6.07727007617015	1.26445728387139
H	0.86887714116353	4.30895278449654	2.38732239635645
H	-1.14380928505573	3.22420619261097	2.07706570091056
H	-3.15590840393967	2.63464302198283	3.37923184866308
H	-3.03675302396352	0.85781866959539	5.13162056255453

228

A-E

C	1.08210904223429	-4.23710061291993	-0.22207131552033
C	-0.14473501501807	-4.47326810821779	0.62345095118567
C	-0.74934392207649	-5.88509344443209	0.52375855850941
C	-1.68201136325688	-6.07820490495288	-0.67097691651608
O	-1.03917207671579	-5.87872361670111	-1.92950668208808
H	0.13436820165764	-4.27807724394495	1.67050705671165
H	-1.33297678263682	-6.08797337823897	1.43813561521457
H	-2.54863335881850	-5.39880145780016	-0.57325729345339

C	2.26643820326746	-5.10849712475874	-0.02779236275757
C	3.12489482149995	-5.38642050543235	-1.10662070865301
C	2.58340745975872	-5.66060523941335	1.22562908481624
C	4.27830766335745	-6.14773036765451	-0.93260426932327
H	2.86662693975671	-5.00779442508940	-2.09521563921262
C	3.74006734792739	-6.41898237346144	1.40275045665893
H	1.93780809470795	-5.46388864541084	2.08026503402185
C	4.59729722989168	-6.66116287190486	0.32693956849815
H	4.92730823548311	-6.35032637128840	-1.78671472452462
H	3.97406895893720	-6.82221677642306	2.38954603113534
H	5.50138134400826	-7.25578842716465	0.46673818150840
C	1.13701587785102	-3.21514777779194	-1.10563844784223
H	2.02970699096113	-3.01856163148811	-1.69866490562432
H	0.24818719154304	-2.62404880988932	-1.31940801941810
H	2.77952189822210	5.31115210949955	-5.13881760786082
C	2.60625082928246	4.49827003929883	-5.85799644685453
C	2.49938480391232	3.13182655529416	-5.14834642112497
H	3.44321045028479	4.49028663784855	-6.57283571284588
H	1.68280742862237	4.72562925667492	-6.40987836664261
C	1.32463290689536	3.18691295847607	-4.16561760950656
C	3.81186694583291	2.85577829106825	-4.38904750142177
C	2.32009082969344	2.04129274166831	-6.21390920694532
C	2.35626613738074	-0.69290931968486	-3.36777932806799
C	1.34027464269975	4.10381791838428	-3.10067232169713
C	0.18825083372850	2.37685552456215	-4.29036971302176
H	4.00401235019524	3.61890005127678	-3.62325887213066
H	3.78418863896700	1.87598220028976	-3.89448116925898
H	4.66026407530202	2.85811438645739	-5.09009126215150
H	1.42067472975381	2.21046831275526	-6.82354171191743
H	3.18638134782413	2.04668405345400	-6.89118387748668
H	2.25462267567436	1.03921173672746	-5.76618282133297
C	3.58539819599147	-0.93804755399140	-2.72299470422062
C	1.79199113192984	-1.63783184297109	-4.21553574451961
H	1.82462244870409	0.24247362282024	-3.20906996975870
C	0.26700207316764	4.22047041115460	-2.22481772678600
H	2.21036151344834	4.74255921338117	-2.94141630427321
C	-0.89248311487581	2.48799582505220	-3.41527270428529
H	0.12552017434773	1.63885383709227	-5.08943512360279
C	4.23545732019499	0.05581411977068	-1.83857384786472
C	4.22271277916530	-2.15911324754672	-2.99671588170293
C	2.42328966748546	-2.86284446494810	-4.48804388610192
H	0.81641202468109	-1.41650012974080	-4.65056411746633
C	-0.88228039056192	3.42447220935329	-2.37157477849043
H	0.32961245950642	4.92551252417452	-1.39459954631233
H	-1.75061571829399	1.83480251857070	-3.55877502567620
C	3.50066470477186	0.98046005870024	-1.04112438281706
C	5.61797706846965	0.16701558573267	-1.78865078529074
C	3.66040052482319	-3.08990140438530	-3.86984936648062
H	5.16060371964223	-2.40454006834594	-2.49753120514758
C	1.73601911504948	-3.89264550956103	-5.38557910078769
C	-2.02873022494815	3.62078932027314	-1.45620867907560
O	2.10385976615605	0.86553122627002	-1.05388164037062
C	4.07540074358118	2.02531762800146	-0.32775550035954
H	6.22592413659639	-0.49070183709469	-2.41152244291776
C	6.27545528467385	1.11129854703326	-0.96871589272698
H	4.19433124112032	-4.02377539542429	-4.04311088662348
C	1.42145115693833	-3.25136416619999	-6.75253143334113
C	0.42172266304255	-4.33575324733111	-4.70865295202413

C	2.60783701274888	-5.13386732464261	-5.61983823121402
C	-2.77478118772680	2.53607268916949	-0.91814847583148
C	-2.41163494333713	4.89155598110157	-1.05721020553698
P	1.28480339162047	0.54184060218197	0.29061230573568
C	5.50325973760948	2.06074902611388	-0.22116499625510
C	3.22403345236408	3.09698286350580	0.25582663902009
C	7.69240392442634	1.14889705412045	-0.87703257222760
H	2.34214085980337	-2.91639438068400	-7.25272484641263
H	0.92144558778438	-3.98366002199068	-7.40366404487597
H	0.75453012832308	-2.38474544025955	-6.64978805494643
H	-0.24812297296564	-3.48552914470184	-4.52095708573035
H	-0.10956571358186	-5.05439500668155	-5.35037772333249
H	0.62036454364230	-4.82819852187746	-3.74625838366664
H	2.81882723554959	-5.66792437184964	-4.68157152905802
H	2.07679338584225	-5.83340529752968	-6.28075525656257
H	3.56453491156658	-4.87872798281849	-6.09997128314476
O	-2.41846934092955	1.24366071499965	-1.31397230297422
C	-3.83244315255364	2.67254941627765	-0.03076571284370
H	-1.88579727089222	5.75653930775766	-1.46629501621181
C	-3.46103586447930	5.11045886710712	-0.13415772836527
O	1.99510449782161	1.42020200422343	1.42517617966211
N	-0.17707432836277	0.91431111783883	-0.02087083076126
N	1.63598417789332	-0.98779389022324	0.84778461222416
C	6.18353355589693	2.97625641260959	0.62198986168926
C	3.38938262287720	4.47132732376374	-0.12469351935477
C	2.18383987341241	2.78352299326993	1.11355247712541
H	8.27056040368323	0.42643734653499	-1.45568449632775
C	8.32389757389969	2.05933751049399	-0.05792670076597
P	-1.61995103302689	0.30783282786306	-0.26205078535768
C	-4.50868378714034	1.46225615541870	0.50975886273109
C	-4.18455688007919	3.99297327020433	0.40339264350635
C	-3.79110365019091	6.42285286696460	0.29847469303340
S	2.23066281301859	-1.43438497556438	2.36431224908554
H	1.47405820209617	-1.81824441198936	0.21730863989748
C	7.56031785173631	2.97232379844202	0.70491309627676
H	5.60333227555384	3.67650970499147	1.22166029484490
C	4.34709919586792	4.90209783572683	-1.07932233875801
C	2.50386525034251	5.45036098389898	0.43935839840389
C	1.28629397083026	3.72349731188934	1.68665378866254
H	9.41250240332697	2.07150978386862	0.01161136741272
O	-2.36331062069035	0.61264123570736	1.14134917868268
N	-1.78495435607863	-1.20819756169563	-0.69581429696272
C	-3.75685275903553	0.47371974547595	1.13218519556283
C	-5.92408040342506	1.26824012476351	0.40667150972307
C	-5.18043599193807	4.24674046706805	1.38294249397833
H	-3.23906721298760	7.26350593260157	-0.12738402991593
C	-4.77191662309626	6.63447930546529	1.24240872202045
O	2.06223795740296	-2.87067564494545	2.40599121466730
O	1.67320974600879	-0.57624593842171	3.38749055100275
H	8.06277638975714	3.67098669275878	1.37477505456983
H	5.00754917635922	4.16936447900338	-1.53876163286200
C	4.44307389493369	6.22985695816129	-1.44223878511402
C	2.64116833420395	6.81138107470782	0.05604907582260
C	1.48853810346734	5.04946738837420	1.33898090081762
C	0.17861936892807	3.32945937640868	2.58367326401519
S	-2.22577419835633	-1.72523997150106	-2.15433031135326
C	-4.30921534386680	-0.66695341300910	1.77680954698707
C	-6.51259019974511	0.12869075655017	1.05007746810699

C	-6.76308639640913	2.12905560323052	-0.34654375047192
C	-5.46514045174066	5.53290341087349	1.79371649539882
H	-5.72187747941178	3.40880664199104	1.81853730759870
H	-5.01031934035536	7.64716028452217	1.57041089224185
H	5.18123823614502	6.53291683264716	-2.18621688563705
C	3.58968821482627	7.19736312944058	-0.86523977946761
H	1.96582329414931	7.54584036112151	0.49979927462822
H	0.83747188321273	5.81263844113333	1.76967716678683
C	0.34263234645053	2.39212035716968	3.61222426102854
C	-1.08400251247319	3.92903432565074	2.43994295784778
O	-1.60590047314313	-0.97826458462090	-3.24781099205969
O	-2.08864352020138	-3.18125498915262	-2.14709712253800
C	-5.68939552360168	-0.78987821468212	1.74130865653515
C	-3.47938528051223	-1.66910714804799	2.48266138289963
C	-7.91633160309129	-0.06625030347076	0.96096921401521
C	-8.12065282673734	1.89826259794724	-0.43217417686475
H	-6.31928177767740	2.96993548519407	-0.87776880587909
H	-6.23030086956024	5.69922273351098	2.55350563821894
H	3.67861239172678	8.24466387660176	-1.15690933241250
C	-0.70937043994938	2.08279477681755	4.47415944560473
H	1.30038440726599	1.89540056990277	3.75348597350206
C	-2.12589639062167	3.61842514994529	3.30581298412932
H	-1.25903812664654	4.62692292634445	1.61967671299795
H	-6.16138129374452	-1.62903270701826	2.25426728836093
C	-2.36883907292753	-1.31146956684323	3.26953884241413
C	-3.83246870578120	-3.02587332602540	2.44061408868781
H	-8.35353636811098	-0.93470513193525	1.45716566144880
C	-8.70678492364743	0.79970484604860	0.23677910238518
H	-8.74359337425133	2.56215209098351	-1.03295557818602
F	5.84986725757125	2.31879563122338	3.82486178551627
C	-1.96456655586715	2.69277512883123	4.35080922893409
H	-0.52985608339084	1.35059530010868	5.26073042342428
H	-3.08924853116960	4.10598573249068	3.15144660453152
C	-1.68922645256651	-2.26611733029500	4.01821887887679
H	-2.04139206942200	-0.27390353236390	3.31540754601340
C	-3.14410132560523	-3.97683784320009	3.19449021274275
H	-4.65924720636226	-3.34590459110719	1.80377487507413
H	-9.78193797644642	0.62973401303783	0.16444587829063
C	6.53780319215988	1.14524325735964	3.86813179180121
C	-3.12206708201948	2.41664454281733	5.31631403982426
C	-2.07324354887416	-3.61791141176125	4.02378289992109
H	-0.84211075151201	-1.93782077432809	4.62336177953249
H	-3.46424766020843	-5.01655061907277	3.13297828885613
F	6.51773082266635	0.72945003724964	5.16724921158051
C	5.92633178040414	0.10218728900708	2.96452495076334
F	7.82979331916626	1.41259348572870	3.55559124902838
C	-2.77758761659817	1.33133391824223	6.34587301746926
C	-4.36886917995830	1.96131296070740	4.53324056482848
C	-3.44994746205068	3.72389264465127	6.06863352487722
C	-1.36950793339754	-4.61229370947007	4.95368421043701
C	6.72631123437295	-0.70434682154689	2.15852990305825
C	4.54165205423240	-0.08894096558024	3.00814651998204
H	-3.64008141229183	1.16889276199719	7.00820676225327
H	-2.54459177083547	0.37126753838788	5.86256916142939
H	-1.92459779544028	1.62102893292275	6.97673164883353
H	-4.18534709176582	1.01443694447765	4.00877125399833
H	-5.21156086812596	1.80913219196911	5.22436795000562
H	-4.67371714232959	2.70754042144367	3.78795414316991

H	-3.74699306017891	4.52130330699713	5.37302494882200
H	-4.27953690632525	3.55855220315746	6.77282207424939
H	-2.57836275665559	4.07786356994138	6.63809976393018
C	0.14634352976968	-4.62377337683356	4.68261584273254
C	-1.90115608401468	-6.04245333650917	4.78810317201999
C	-1.61732721024663	-4.16657312065333	6.41132363228301
C	6.13837222935769	-1.72135097611354	1.39709979702190
H	7.80206654149382	-0.54168075756744	2.11821144156892
C	3.98247684100442	-1.10663375158713	2.24504897043115
H	3.90363809963310	0.53287460538196	3.63494185479020
H	0.59276182474653	-3.62527992956950	4.77234747589027
H	0.65137213541438	-5.28972962062063	5.39852815122887
H	0.35829609332148	-4.99024894245826	3.66904661463760
H	-1.36366434285678	-6.71517322804146	5.47181035466721
H	-2.97239037705628	-6.11170898155148	5.02678727726650
H	-1.74905916663360	-6.41727109185472	3.76518529806112
H	-1.13267027104500	-4.86568508076754	7.10973233484738
H	-1.21114943411012	-3.16277459046658	6.59768745263440
H	-2.69354549631346	-4.14339612119895	6.63578530267177
C	4.76335172425545	-1.93749769771202	1.43764329545532
C	7.01948395844714	-2.64285804843046	0.58829209171066
H	4.30645839312001	-2.74634965327013	0.86551513786618
F	8.07746908646257	-1.98266794750635	0.04074016125948
F	6.34368825685879	-3.23880022236090	-0.42639992138374
F	7.53483456932064	-3.63587286156759	1.36494558606762
C	-4.87323084285720	-2.31154212431041	-1.63618523574949
C	-3.99637563493113	-1.42561938044107	-2.26031232370833
H	-4.47885530553030	-3.16597491855333	-1.08624951504677
C	-6.24817372488763	-2.09956349526638	-1.75023638316616
C	-4.47265685555574	-0.34243346929167	-2.99244240444552
C	-6.74480922654180	-1.00886097288958	-2.46737396819888
C	-7.18983681596791	-3.10701613209496	-1.14141357222567
C	-5.85073695573819	-0.13396182022536	-3.08383377564006
H	-3.76776533800844	0.32108726126255	-3.49053403042259
H	-7.81705106326888	-0.84021568799552	-2.53873926439423
F	-6.83258443306759	-3.43250110275580	0.13690728788447
F	-7.19263808766865	-4.27243575014596	-1.84764420644723
F	-8.47285031037170	-2.66503093470191	-1.09752904047579
C	-6.34517818495426	1.03222271701744	-3.90067429953458
F	-7.67912007544844	1.24275525386369	-3.75967009872229
F	-5.71216942354698	2.19115579485649	-3.55762312622306
F	-6.10859137161848	0.84240292489236	-5.23061029311870
H	-1.16574373625030	-4.93735748564060	-2.15785176118222
H	-2.06868043308664	-7.11094548318294	-0.66578668440184
H	0.05273021085180	-6.63855786250945	0.48489129657112
H	-0.90394778634984	-3.71718841044267	0.37227327707068

228

A-TS1

C	0.954186	-4.171081	-0.174685
C	-0.229143	-4.264120	0.735844
C	-0.957058	-5.616705	0.718866
C	-1.756069	-5.814272	-0.565051
O	-0.927735	-5.660601	-1.720360
H	0.149875	-4.073884	1.754547
H	-1.643318	-5.661073	1.579062
H	-2.588634	-5.091851	-0.599696
C	2.023372	-5.174769	-0.082946
C	2.750157	-5.523315	-1.235735

C	2.352001	-5.800537	1.134090
C	3.779188	-6.458369	-1.174882
H	2.476709	-5.073270	-2.189029
C	3.394119	-6.720903	1.197482
H	1.823048	-5.521464	2.043884
C	4.109663	-7.055552	0.044045
H	4.322098	-6.727123	-2.082693
H	3.652953	-7.178678	2.153309
H	4.921011	-7.783323	0.095715
C	1.117247	-3.063159	-0.994723
H	1.958299	-3.035399	-1.689802
H	0.215593	-2.523445	-1.293692
H	2.784268	5.304498	-5.192393
C	2.612879	4.488197	-5.908068
C	2.519183	3.123694	-5.192966
H	3.446099	4.483804	-6.627371
H	1.684815	4.707380	-6.455551
C	1.350768	3.174119	-4.202585
C	3.838184	2.858455	-4.441033
C	2.340034	2.028414	-6.253692
C	2.408096	-0.730613	-3.400324
C	1.369900	4.092159	-3.138952
C	0.218357	2.357187	-4.316767
H	4.030929	3.626027	-3.679853
H	3.819403	1.881441	-3.940489
H	4.682151	2.862265	-5.147433
H	1.435404	2.189469	-6.857815
H	3.201892	2.036792	-6.936621
H	2.283807	1.027517	-5.801879
C	3.623415	-0.959372	-2.725251
C	1.853747	-1.702168	-4.224771
H	1.881250	0.213905	-3.282667
C	0.305472	4.200629	-2.251677
H	2.236967	4.736666	-2.987575
C	-0.854302	2.460983	-3.430915
H	0.151872	1.618866	-5.115322
C	4.266871	0.067925	-1.874758
C	4.250376	-2.198729	-2.929407
C	2.481685	-2.940646	-4.440137
H	0.892577	-1.487053	-4.694253
C	-0.839181	3.395879	-2.385758
H	0.372992	4.904887	-1.421367
H	-1.707827	1.800307	-3.565591
C	3.519227	0.998163	-1.097136
C	5.646948	0.195795	-1.834703
C	3.697203	-3.159393	-3.777351
H	5.176531	-2.428729	-2.400669
C	1.820982	-3.982494	-5.345845
C	-1.976391	3.583840	-1.457215
O	2.127872	0.871620	-1.110429
C	4.083851	2.051291	-0.388107
H	6.260965	-0.468787	-2.444071
C	6.294377	1.159606	-1.028804
H	4.225657	-4.104346	-3.901198
C	1.605767	-3.370258	-6.745530
C	0.456498	-4.379495	-4.744731
C	2.675542	-5.248307	-5.498523
C	-2.720593	2.495154	-0.923814

C	-2.355200	4.851295	-1.044042
P	1.349177	0.460578	0.243678
C	5.511786	2.104428	-0.285448
C	3.216001	3.100875	0.210787
C	7.710646	1.211490	-0.937544
H	2.563523	-3.068648	-7.194369
H	1.129711	-4.107322	-7.409311
H	0.955838	-2.485804	-6.704577
H	-0.198487	-3.509325	-4.603171
H	-0.057267	-5.088178	-5.411460
H	0.581065	-4.870388	-3.768961
H	2.826967	-5.759763	-4.536250
H	2.164541	-5.956001	-6.166593
H	3.661270	-5.027298	-5.934241
O	-2.374500	1.207099	-1.337670
C	-3.773146	2.625537	-0.029418
H	-1.831972	5.719475	-1.449567
C	-3.396884	5.063362	-0.110935
O	2.024695	1.392234	1.371541
N	-0.129392	0.816102	-0.054768
N	1.761509	-1.052754	0.707041
C	6.183800	3.029382	0.554049
C	3.359364	4.483378	-0.146248
C	2.185716	2.753968	1.069821
H	8.296105	0.490567	-1.511261
C	8.333755	2.131198	-0.122087
P	-1.573727	0.247791	-0.306532
C	-4.456382	1.414074	0.499647
C	-4.117895	3.942354	0.422392
C	-3.721427	6.372576	0.335420
S	2.299072	-1.483176	2.199772
H	1.470549	-2.119631	-0.081854
C	7.560861	3.039264	0.637119
H	5.597006	3.725173	1.152208
C	4.303423	4.944406	-1.100602
C	2.467726	5.440975	0.444229
C	1.282904	3.674927	1.667810
H	9.422177	2.152731	-0.051568
O	-2.319964	0.525870	1.102713
N	-1.786389	-1.258658	-0.781752
C	-3.712515	0.410129	1.107705
C	-5.875625	1.242118	0.410416
C	-5.103516	4.189268	1.413924
H	-3.171110	7.215794	-0.087599
C	-4.693949	6.577744	1.289298
O	2.291085	-2.939355	2.204185
O	1.624925	-0.760164	3.266427
H	8.055816	3.745714	1.304559
H	4.967742	4.227991	-1.580132
C	4.381918	6.280162	-1.437599
C	2.586850	6.810945	0.087377
C	1.465860	5.010047	1.344928
C	0.191591	3.252907	2.572820
S	-2.234912	-1.708122	-2.255402
C	-4.278027	-0.717304	1.767180
C	-6.476213	0.113994	1.062045
C	-6.708345	2.116946	-0.333436
C	-5.383052	5.472293	1.837914

H	-5.639930	3.348410	1.849938
H	-4.928106	7.587963	1.627830
H	5.110462	6.606390	-2.181355
C	3.523090	7.226379	-0.833785
H	1.907288	7.528683	0.551775
H	0.812291	5.757780	1.798337
C	0.367912	2.278172	3.564573
C	-1.069899	3.864802	2.477884
O	-1.614100	-0.934101	-3.328730
O	-2.110874	-3.168027	-2.313054
C	-5.661105	-0.813804	1.749945
C	-3.460660	-1.724044	2.482507
C	-7.884107	-0.056886	0.989600
C	-8.070585	1.909448	-0.403390
H	-6.256122	2.950379	-0.869229
H	-6.140335	5.632917	2.606810
H	3.598210	8.280415	-1.104474
C	-0.668597	1.945591	4.436391
H	1.321847	1.765829	3.667629
C	-2.096580	3.531013	3.353367
H	-1.257083	4.593241	1.687617
H	-6.144266	-1.636213	2.279165
C	-2.300385	-1.385350	3.204748
C	-3.876967	-3.063685	2.524587
H	-8.330510	-0.916808	1.492524
C	-8.667877	0.821632	0.273325
H	-8.688866	2.583822	-0.997294
F	5.439173	2.655943	3.789567
C	-1.921477	2.567774	4.361308
H	-0.477906	1.183516	5.191307
H	-3.058791	4.030795	3.235408
C	-1.627769	-2.336886	3.963557
H	-1.924255	-0.364306	3.192732
C	-3.204620	-4.008199	3.300983
H	-4.744911	-3.375670	1.940648
H	-9.746597	0.670269	0.213746
C	6.207477	1.545116	3.967616
C	-3.060436	2.265664	5.340961
C	-2.075553	-3.665273	4.057124
H	-0.732527	-2.021740	4.502584
H	-3.583589	-5.030021	3.315269
F	6.081451	1.191897	5.279190
C	5.779671	0.418189	3.060588
F	7.500758	1.908104	3.775487
C	-2.709073	1.130283	6.312734
C	-4.332601	1.864546	4.569139
C	-3.349813	3.542492	6.158401
C	-1.360582	-4.650870	4.987830
C	6.718032	-0.308228	2.329124
C	4.424357	0.081270	3.012629
H	-3.559087	0.949931	6.986614
H	-2.499862	0.190373	5.781560
H	-1.838418	1.380012	6.936459
H	-4.172042	0.948333	3.985775
H	-5.157020	1.679305	5.274216
H	-4.652380	2.655107	3.877475
H	-3.646316	4.375475	5.505486
H	-4.167524	3.359721	6.872274

H	-2.460308	3.855405	6.724160
C	0.109959	-4.817206	4.557255
C	-2.022937	-6.035319	4.990412
C	-1.405850	-4.088448	6.424819
C	6.293080	-1.382483	1.542528
H	7.770950	-0.036390	2.363832
C	4.022729	-0.988570	2.220043
H	3.683494	0.632972	3.589646
H	0.632415	-3.854862	4.475936
H	0.649571	-5.439536	5.287208
H	0.168997	-5.318494	3.580705
H	-1.479765	-6.701120	5.676460
H	-3.068270	-5.987477	5.328674
H	-2.002526	-6.499597	3.993285
H	-0.914977	-4.786129	7.120100
H	-0.890592	-3.120923	6.495471
H	-2.444596	-3.944042	6.755317
C	4.943786	-1.730078	1.480029
C	7.300056	-2.234053	0.810562
H	4.609408	-2.576288	0.878848
F	8.496145	-1.610573	0.657267
F	6.863795	-2.584480	-0.433388
F	7.539399	-3.397697	1.479153
C	-4.880812	-2.274145	-1.706850
C	-4.003911	-1.393929	-2.339036
H	-4.489316	-3.135812	-1.166555
C	-6.254803	-2.046926	-1.796886
C	-4.479656	-0.297987	-3.052105
C	-6.750824	-0.944451	-2.496400
C	-7.196387	-3.052156	-1.184589
C	-5.856900	-0.073656	-3.118635
H	-3.775490	0.364013	-3.553531
H	-7.822093	-0.763051	-2.548533
F	-6.817714	-3.402992	0.080560
F	-7.226101	-4.206886	-1.909496
F	-8.472914	-2.597529	-1.109384
C	-6.351700	1.106617	-3.915178
F	-7.682114	1.325978	-3.754872
F	-5.705093	2.255702	-3.566571
F	-6.133464	0.930006	-5.250128
H	-1.169615	-4.795344	-2.111482
H	-2.187532	-6.829526	-0.582516
H	-0.233482	-6.438018	0.832519
H	-0.928704	-3.445667	0.511028

228

A-P

C	0.35000118224245	-4.40574651798565	-0.84692258760425
C	-0.07991260806752	-4.18930730737286	0.59915820062740
C	-0.93422331025604	-5.42219940956308	0.90656330180369
C	-1.70489247886237	-5.66381149924050	-0.38796231432321
O	-0.96330918742090	-4.92632000951641	-1.45091546040406
H	0.77947481178347	-4.07778700379730	1.26796744249500
H	-1.61572374398652	-5.24317351480055	1.74620289875251
H	-2.71284757018176	-5.23200499870241	-0.38180248062186
C	1.38427709014258	-5.50476640951105	-1.02190200666280
C	1.20319028418002	-6.58440169386709	-1.89259754650338
C	2.59862144390027	-5.37984915722037	-0.33073498900035
C	2.21656401494228	-7.53126059746390	-2.06565939538685

H	0.27126849540155	-6.68299946095524	-2.44948444430524
C	3.60902565484700	-6.32333872480482	-0.51034337407854
H	2.75325618116512	-4.53992192890302	0.35008825656178
C	3.42300822454206	-7.40305435193345	-1.37780361507609
H	2.06093152964411	-8.36778147416125	-2.74887662693589
H	4.54848533666780	-6.21279859604333	0.03378823903000
H	4.21486666585052	-8.14076208768321	-1.51560719990820
C	0.74145928245590	-3.17053540362282	-1.62593890209375
H	0.79444139502581	-3.38397863074405	-2.69842222936459
H	0.05682113731927	-2.33871545528036	-1.43600250186020
H	2.73510767081449	5.07836282358726	-5.38427283482570
C	2.58423739427331	4.22408065724782	-6.05880476789047
C	2.53392961066001	2.89294586447318	-5.27847086996594
H	3.41391551286026	4.20907095645156	-6.78204193579743
H	1.64746318538670	4.38977519331718	-6.61036257644176
C	1.37096158922147	2.96088217698761	-4.28256977327317
C	3.86607848924172	2.70401758540647	-4.52460607563385
C	2.37658386309259	1.74768054079980	-6.28831082464818
C	2.79007985189853	-0.88217447225085	-3.43168624652731
C	1.38133051592542	3.93175368399615	-3.26795329866175
C	0.25330982453440	2.11768524330003	-4.34260808473999
H	4.04434342408082	3.51884541511557	-3.81017502460401
H	3.88000225659939	1.75837607575155	-3.96671033231382
H	4.70382532058116	2.69029911954459	-5.23809194542816
H	1.46011282341888	1.85588001651344	-6.88643177912717
H	3.22971619498613	1.75170541308097	-6.98208296959771
H	2.35758429236545	0.76592412625967	-5.79499849637168
C	3.84444450266045	-1.08122434530609	-2.52250845913299
C	2.42787358574365	-1.88078611602993	-4.32848203655134
H	2.26001489846489	0.06929672822905	-3.44951478382405
C	0.32129893756708	4.06963215024855	-2.38179110723764
H	2.23834286406452	4.59643572787777	-3.15372911681070
C	-0.81734595672214	2.25015782232549	-3.45597886939515
H	0.19513050753129	1.33710889916011	-5.10111078897661
C	4.37278767758972	0.01914970543218	-1.68523691647677
C	4.45802331288398	-2.34202897861605	-2.50619728643791
C	3.06764916427221	-3.13204314556597	-4.34766989841246
H	1.61811163915366	-1.67414611368106	-5.03130021600603
C	-0.81336527825763	3.24360852737077	-2.46555354578618
H	0.38504586060060	4.81628589665583	-1.58974720848688
H	-1.66066957884730	1.56893441926255	-3.55013832127173
C	3.53272656087195	0.96194217210725	-1.02918334129456
C	5.74010870763835	0.20386759911345	-1.56391186725701
C	4.07113240766808	-3.34716475416417	-3.39438606913555
H	5.25098963209557	-2.54259633868243	-1.78319016891450
C	2.67078501084773	-4.18022109100086	-5.39296430535378
C	-1.94209737432569	3.48797259549796	-1.53879589291560
O	2.15351491826729	0.77577958258835	-1.12266414208381
C	4.00853885500082	2.06959101381036	-0.33880076004976
H	6.41599001268895	-0.47832038710952	-2.08177033811780
C	6.29345212260363	1.24757912791550	-0.78760354214360
H	4.57083121285207	-4.31326328025376	-3.33313310199348
C	2.94299797759058	-3.59979174070902	-6.79676311714749
C	1.17061322685982	-4.51452040837283	-5.26688134157587
C	3.46840572761102	-5.48258500392007	-5.23952510779746
C	-2.69936069126582	2.44622032547758	-0.93446289114349
C	-2.29017829814000	4.78369406391298	-1.18802375119374
P	1.37063586275608	0.30753921550424	0.22280487728998

C	5.42438566213938	2.19539193905769	-0.15216721827583
C	3.06139415785809	3.08601745658269	0.19132631944728
C	7.69734765250017	1.37052831310637	-0.61564042160520
H	4.00665247998883	-3.34739009322343	-6.91502392857215
H	2.67353419087528	-4.33482323011027	-7.57038573760000
H	2.35740367996820	-2.68756447447714	-6.97670562376972
H	0.53983084014891	-3.61889578397757	-5.35330044800691
H	0.87267665243320	-5.21412750658410	-6.06250194457662
H	0.95810196827203	-4.99195389266188	-4.30001782866814
H	3.30544317601381	-5.94550650185823	-4.25569404036986
H	3.14642982489577	-6.20203304251430	-6.00655303726670
H	4.54747345793305	-5.31607801564596	-5.37158567508814
O	-2.41404792953658	1.13006473790338	-1.30876580262605
C	-3.71551099332871	2.64947550555454	-0.00997728728989
H	-1.76181090348245	5.61745181912987	-1.65390361786154
C	-3.29787822062852	5.06986038239853	-0.23914898461057
O	1.90511695280148	1.35194237918372	1.34657570079395
N	-0.11779410462565	0.59923291110386	-0.17710609233176
N	1.83119062505145	-1.14640814808385	0.67425524524342
C	6.00312698904368	3.19242641339266	0.67481585483766
C	3.16109107684802	4.46677569976186	-0.18120051520247
C	2.02485408186597	2.70451269637222	1.03087958732108
H	8.34733180864317	0.64495229800560	-1.10834593408820
C	8.22870029515343	2.36246432067775	0.17988085576826
P	-1.60628248657928	0.17852892801664	-0.27111789877596
C	-4.41103100055077	1.48678871497069	0.60401285966400
C	-4.01430258251755	3.99529595601653	0.38615210189288
C	-3.58552786682841	6.40924747324461	0.13836658914938
S	2.34633046285971	-1.58440286331488	2.12594706600342
H	1.73774022276397	-2.85656254659296	-1.29529952435755
C	7.37072230713521	3.27154977202030	0.83931117906821
H	5.35169963792033	3.88913282392594	1.20008653262141
C	4.10348828575963	4.94704249978952	-1.12789640602365
C	2.23336509736065	5.40158566467550	0.38841177616049
C	1.09874842276445	3.60929726259885	1.62253325625434
H	9.30864707391136	2.43785528505659	0.31476887687785
O	-2.27762423871099	0.55296250804497	1.15181189219657
N	-2.01215559050102	-1.33549416683218	-0.66221984868937
C	-3.67303853871576	0.48317671714488	1.22012858828971
C	-5.83860497661852	1.36280429165571	0.58937730395943
C	-4.94898664955983	4.31732761810543	1.40517572999046
H	-3.03964917170483	7.21571987200815	-0.35592883623424
C	-4.51535134555197	6.68729696971193	1.11576444375728
O	2.54090008631342	-3.03666866322262	2.09492334704954
O	1.56046816471364	-1.02389062058559	3.22561653527755
H	7.79098966895700	4.03445159585533	1.49589405525194
H	4.79574897489066	4.24470859215703	-1.58953417578052
C	4.14485779978720	6.28106757719204	-1.47828146684460
C	2.31391690391566	6.77097234383554	0.01847193583071
C	1.24350429975003	4.94726294726795	1.29020369203316
C	0.03879029348048	3.17631661026784	2.56013681443120
S	-2.59183824345653	-1.76311017846040	-2.06468694106415
C	-4.24648087035304	-0.60547494370815	1.93787909516122
C	-6.44617824047682	0.28668805744304	1.31757939957330
C	-6.67712536988948	2.23806520487308	-0.14780377733001
C	-5.19312120075821	5.62779540180958	1.76097813353934
H	-5.47303160272604	3.51305570026787	1.91852756818419
H	-4.72195907265849	7.71971472044336	1.40106735786504

H	4.87335448684533	6.62304084744643	-2.21510874837491
C	3.24840908734557	7.20603058418461	-0.89572426443485
H	1.60723225002855	7.47213112460768	0.46778847093163
H	0.57461495373837	5.68142206500992	1.74323317619957
C	0.24296411699121	2.18533455341982	3.53217267655146
C	-1.21468578563815	3.81148624400097	2.53746556584640
O	-1.97639599964576	-1.15609904840884	-3.23640599420979
O	-2.63450237221114	-3.26744816301703	-2.06668158507872
C	-5.63108116021912	-0.65060685149827	1.99212542649418
C	-3.42822580395450	-1.62504504619488	2.63226665716417
C	-7.86174084786333	0.17403077459497	1.33173803175893
C	-8.04862366331926	2.08732546151623	-0.13239707425923
H	-6.22364900458344	3.02981515865193	-0.74229232267489
H	-5.91194464413474	5.84654585325063	2.55215022025977
H	3.29468531035638	8.25928530913323	-1.17603706671561
C	-0.75358090383433	1.86623698171226	4.45440716934465
H	1.18700811700181	1.64805384551030	3.58069545060880
C	-2.20172885008521	3.48848592912233	3.46077188298290
H	-1.42781096865718	4.55530792676945	1.76872922596992
H	-6.11584822400853	-1.43672288988554	2.57274511337685
C	-2.21513062588121	-1.30858665075461	3.27125292951711
C	-3.87960793188746	-2.95106352383447	2.72688912926109
H	-8.31281657345564	-0.64396977342459	1.89653102145123
C	-8.64977302936192	1.05648304448131	0.62493257238837
H	-8.67178679406361	2.76331124847210	-0.71935918482280
F	4.88450246585522	2.91546733986574	3.83369013892509
C	-1.99628806127494	2.51267600063004	4.44962757067365
H	-0.53876593240156	1.09349520354314	5.19192984782488
H	-3.15684717034419	4.01076972777114	3.39745160264816
C	-1.50781187541271	-2.26984853780045	3.98273360193803
H	-1.81828101716390	-0.29717010944548	3.22655695414416
C	-3.17124643193229	-3.90706324325261	3.45791812801607
H	-4.80148989146739	-3.24376711134916	2.21961034485315
H	-9.73550695444075	0.95073354834605	0.63484922870357
C	5.73144598139124	1.89604052051181	4.15371015787543
C	-3.09319612775540	2.22216245634101	5.47905163232239
C	-1.97141250980380	-3.58891944102429	4.11130337059733
H	-0.56379185504334	-1.97224583926948	4.44040068205533
H	-3.57435751674522	-4.91865896063253	3.51635237048837
F	5.49140174994949	1.59101118893603	5.46220796056411
C	5.52238173218542	0.69638185055113	3.26558949283640
F	6.99713629223427	2.38425457077790	4.08625935562971
C	-2.72124058451007	1.06425828691139	6.41598446792124
C	-4.40873927358261	1.85902230401972	4.76260561461152
C	-3.31398904931279	3.49212895783001	6.32770113793799
C	-1.18819120536782	-4.58695647721358	4.96956917212411
C	6.60440424648598	0.04792337786801	2.67101955930834
C	4.22147224393613	0.21706073186223	3.09348438699530
H	-3.54299499408647	0.88898047873252	7.12546077700108
H	-2.55331069337351	0.13001821507137	5.86067810679621
H	-1.81825984178222	1.28744069344884	7.00260665052139
H	-4.29523010710385	0.94389585035328	4.16696859297290
H	-5.20555145693029	1.68861783869109	5.50238686872712
H	-4.74110608724968	2.66104434684880	4.08943426288941
H	-3.62119832947472	4.34213775470235	5.70219066701206
H	-4.10062201662250	3.31724162472013	7.07767344455985
H	-2.39131007752403	3.77592783583715	6.85422317996516
C	0.26698596263932	-4.68923593066452	4.47002200692031

C	-1.81262455124624	-5.98881091914724	4.95258282026580
C	-1.18500856444565	-4.07315514695827	6.42574156776966
C	6.37318658184797	-1.08750343003471	1.89134418962905
H	7.61535394398746	0.42542715788453	2.80751968825659
C	4.00835430213353	-0.90913935821794	2.30419072489600
H	3.37526388528866	0.70091871667476	3.57827175263556
H	0.76883768860536	-3.71371965830641	4.44115146957419
H	0.84470595858734	-5.34965752998835	5.13410740881336
H	0.31021045196107	-5.10953480612874	3.45629462460199
H	-1.21213595062031	-6.66549298469634	5.57725655892250
H	-2.83702088411832	-5.98713034239107	5.35327716454350
H	-1.83924269255711	-6.41273423155494	3.93747002593087
H	-0.63448776288503	-4.77247214384656	7.07311944052441
H	-0.70186439792694	-3.08943188843566	6.49974886524358
H	-2.21071423942023	-3.97822176208023	6.81107995897197
C	5.07693675710411	-1.56734459179142	1.69752699260475
C	7.52590587369435	-1.86189829668013	1.30749678700687
H	4.89297830675519	-2.46091099208893	1.10153375392355
F	8.69194403971814	-1.16772497920586	1.33052922629433
F	7.29692489853070	-2.22199743007178	0.00904556872216
F	7.74045279997184	-3.02298822560303	1.99250758072090
C	-5.24825454652019	-2.15077514397471	-1.40646496217257
C	-4.33869770719839	-1.35594256615765	-2.10098054377797
H	-4.89855161507351	-3.00886769062394	-0.83426552329714
C	-6.60803492157796	-1.84621318005666	-1.47933943608299
C	-4.76128082918388	-0.25754968255823	-2.84400046691967
C	-7.05266312756321	-0.74434270038303	-2.21215654834594
C	-7.59084729141951	-2.77766097914992	-0.81609262082300
C	-6.12316526057546	0.04919056843948	-2.88423090400343
H	-4.03224576390365	0.34051297166044	-3.38860097701106
H	-8.11240560862714	-0.50242402834124	-2.25157950696184
F	-7.20839528909293	-3.10478443181573	0.45313628188380
F	-7.68783163781307	-3.95437325152616	-1.50153885664263
F	-8.84014516256112	-2.25754627476256	-0.74149382707924
C	-6.56779792656053	1.22733689981854	-3.71493456919643
F	-7.87157887623672	1.54598261031786	-3.51126645161816
F	-5.82850411487119	2.33920427531625	-3.44545685449446
F	-6.42024734477160	0.97477656329764	-5.04689839541259
H	-1.60773692563423	-4.07827910073834	-1.74179403200849
H	-1.74207741907292	-6.71102370147614	-0.70760824458253
H	-0.29775515197670	-6.28555755521214	1.14367715326480
H	-0.67699241276489	-3.26781418205363	0.66152520529030

228
B-E

C	0.87119387398588	4.45044892655031	3.44670894413367
C	0.77435116033057	3.60759833456671	4.70126718559296
C	1.88172104910211	2.53842672841513	4.79477232892443
C	1.74841561037046	1.42939616742226	3.75781281802632
O	2.13763109243222	1.87395433580411	2.43027898911474
H	0.83330411788380	4.25376212729785	5.59038262959448
H	1.83073690300584	2.06680026490340	5.78993158688345
H	0.71475269403040	1.05459190867251	3.73321282961521
C	1.91698696465419	5.50379081009392	3.39128911123529
C	2.21392839775930	6.30044357824817	4.51093738162110
C	2.63031825685330	5.74310844447771	2.20343279452173
C	3.16559691725358	7.31748288883937	4.43605531353772
H	1.67526601339683	6.14394571120976	5.44649727972439
C	3.58581814038818	6.75448179579529	2.12949875183753

H	2.45017665751579	5.10522789037240	1.33909399741391
C	3.85608562813170	7.55049674774473	3.24492802084062
H	3.36795475412762	7.93250871012263	5.31467744676309
H	4.12836562986503	6.91385529302296	1.19585015216749
H	4.60567008404324	8.34145607256751	3.18966210410738
C	0.05577394624501	4.21813909709190	2.39643261726492
H	-0.69959809483337	3.43111021536783	2.43840624403964
H	0.11093203905802	4.80800433336117	1.48146052860615
H	1.16866215340689	-7.90063053834545	0.59776488023730
C	0.99227416075877	-7.72543369400778	1.66833487834904
C	1.19068649701969	-6.23978227696064	2.03669139748719
H	1.69392816163430	-8.35441944948683	2.23727179168258
H	-0.03145798777673	-8.05254829793193	1.90086765222494
C	0.19527557406578	-5.40206201799782	1.22629570532936
C	2.63389809566527	-5.82395727519907	1.69092027321634
C	0.99266734284277	-6.08212813877168	3.55082473794334
C	3.88700125403193	-1.52312312724296	3.98514869766401
C	0.25160196728909	-5.40799818422874	-0.17795500327222
C	-0.81624954097323	-4.63497339766113	1.81936278832477
H	2.83848648840042	-5.93193148628725	0.61758657299090
H	2.82088746858566	-4.77904814987980	1.97103631015235
H	3.34977114639994	-6.45593225066102	2.23828304443550
H	1.14432586494713	-5.04285469036427	3.87552583441042
H	-0.01000249167492	-6.40479725230304	3.86718139603286
H	1.72561795905600	-6.70678928778070	4.08181819198719
C	3.15286744015704	-1.97630023588863	2.87501975384049
C	3.30080537786163	-1.40355469795948	5.24597392758732
H	4.93100714737821	-1.23214693817931	3.86336897239572
C	-0.67505228358576	-4.71192156777851	-0.94567805145366
H	1.03656612328333	-5.96554860923900	-0.69141304578652
C	-1.74629943746128	-3.92913097061363	1.05560470675267
H	-0.89757096659589	-4.58195314847155	2.90454561617253
C	3.82244166407871	-2.20607906448000	1.57210615563591
C	1.79612828368961	-2.28987119921211	3.09113102296324
C	1.94813355269589	-1.70714862072719	5.45774287830099
H	3.91537161191445	-1.03886460707353	6.06876299809124
C	-1.70712066203045	-3.97050906411819	-0.34509450536151
H	-0.58442224876615	-4.72273351005680	-2.03290219730393
H	-2.51340817662357	-3.34865839408286	1.56472790900137
C	3.12820479731387	-2.20894584128441	0.32460252276850
C	5.16999872858663	-2.53689250608878	1.52019232488676
C	1.21685372706319	-2.14934613736020	4.34551157004529
H	1.17615489847839	-2.66424535770033	2.28129682408767
C	1.25386508446894	-1.57316373153436	6.81445576374658
C	-2.72166848590612	-3.30749002382862	-1.19402101224473
O	1.78062896710168	-1.82735764717478	0.33615869511297
C	3.68020437223322	-2.66357239243091	-0.86797990136866
H	5.73945851481232	-2.59237946918399	2.44859753953960
C	5.83896241777014	-2.82900431993776	0.31070589194444
H	0.15458146543778	-2.37603312297542	4.44186395129378
C	0.77197336290242	-2.96967006309004	7.26150889135608
C	0.03608688597528	-0.63515726992746	6.67869041828451
C	2.18831942706268	-1.00954865140327	7.89308252811433
C	-3.20577216724948	-1.99613229676360	-0.93222254491181
C	-3.23260964529174	-3.94393161634900	-2.31379558426418
P	1.23691623793243	-0.59813311329702	-0.55102881311240
C	5.09025291721810	-2.90572163274319	-0.90902261753223
C	2.80962664232132	-2.96009645331395	-2.03789552814206

C	7.23744363188281	-3.07539165922879	0.27796304626942
H	0.26210547709978	-2.90128290588184	8.23429408567645
H	0.06578996843176	-3.40010994704810	6.53821705669718
H	1.61934686326528	-3.66339077827277	7.36298187849042
H	-0.49314622073999	-0.56683605472079	7.64102997158541
H	0.34813183863452	0.37783098946129	6.38885149210880
H	-0.67707612691555	-0.98629339094673	5.92133841388599
H	3.05658036241540	-1.66252767942313	8.06562932873778
H	2.55678922455137	-0.00727506229999	7.62803758451259
H	1.64283013202819	-0.92308309158201	8.84363766175488
O	-2.70348680447220	-1.32487288619688	0.18704020265831
C	-4.13706280082787	-1.33358927415230	-1.71750274951840
H	-2.90762785691265	-4.96170610689319	-2.53893460644415
C	-4.16130261278338	-3.32107719611966	-3.18026772923487
O	2.01322182085265	-0.72053120803106	-1.94790566916246
N	-0.29089257444633	-0.80475245316481	-0.65676068604985
N	1.82878643640269	0.84031562034234	0.02308706370895
C	5.78031106487380	-3.18542137970810	-2.11607863988911
C	2.73874793197002	-4.28207343363479	-2.59318992689363
C	1.96539266682940	-1.99289314483642	-2.55288957381979
H	7.79784258338677	-3.02159180367456	1.21295815929889
C	7.87865348501551	-3.35059763644335	-0.91032280668866
P	-1.61102481715484	-0.15431863898723	-0.06494134038845
C	-4.52533374961842	0.06467476897753	-1.39000703827492
C	-4.62516091409569	-1.99320585479520	-2.89331050538908
C	-4.62016164428322	-3.97745994719034	-4.35354437053634
S	2.64378048807305	2.01692281845982	-0.85983007137971
H	1.81586791948576	1.09237472043991	1.07367396575633
C	7.14350633670143	-3.39561419184761	-2.11731956746422
H	5.22201396918578	-3.21235108883327	-3.05130022850123
C	3.47802113850844	-5.38067101899386	-2.08256312246371
C	1.82922660106792	-4.52033438036457	-3.67809318647264
C	1.04846932863221	-2.19448983953147	-3.61911795360029
H	8.95525092600076	-3.52616943388327	-0.92424094337442
O	-2.20489203567892	0.63672610465563	-1.34703740098441
N	-1.53147136906582	0.77618390274846	1.21631827430278
C	-3.54242072189486	1.03937869705986	-1.27282739829878
C	-5.88988772622947	0.45306789733454	-1.19919659045650
C	-5.50276547431603	-1.37348225815488	-3.82135231618739
H	-4.26551316845256	-4.99094146163771	-4.55331218916268
C	-5.47918468734904	-3.34999192473821	-5.22902682637090
O	2.63514549786335	3.18960589088800	-0.00735313662862
O	2.14234274416519	2.07273980733524	-2.21616188077058
H	7.65988593499061	-3.58608133114670	-3.05882886143818
H	4.15168208339743	-5.22499520179169	-1.24233929347922
C	3.34490725874154	-6.64232614117456	-2.62540879649710
C	1.72910106010532	-5.82801477213248	-4.22390472744941
C	1.02136653916016	-3.46690515523854	-4.16684721918311
C	0.14380678376846	-1.13373030333234	-4.11178203852467
S	-2.06436442911022	0.39437220310358	2.68526206062868
C	-3.81040015976323	2.42817938866828	-1.11726301299686
C	-6.19170590901969	1.84414660666214	-1.02016553798396
C	-6.95094824895455	-0.48587686651029	-1.13251276165746
C	-5.91643813540548	-2.03288747147402	-4.96079869096392
H	-5.84642808464119	-0.35853210454909	-3.63000589096637
H	-5.81752133378925	-3.86299777125543	-6.13032906896128
H	3.91726631636970	-7.47163039343221	-2.20715889808500
C	2.46954117253504	-6.87033697630436	-3.71112367562258

H	1.03864536689257	-5.99203262426765	-5.05386428595528
H	0.34732823981640	-3.66663600881639	-5.00213819941648
C	-1.19662032011656	-1.43878125625632	-4.40350942480876
C	0.57829047919588	0.17902509877046	-4.33543503671862
O	-1.76876271622494	-0.98596540205907	3.07202598537207
O	-1.64894064898190	1.46783861667116	3.58587205404938
C	-5.14434387489548	2.79379180464891	-1.02099121537765
C	-2.74360542310016	3.45387069954886	-1.10444714659399
C	-7.54301182105045	2.23842059330398	-0.83336506338606
C	-8.25070057982471	-0.07137623810444	-0.92661274070317
H	-6.72503970206931	-1.54761644609383	-1.22417858434653
H	-6.58455957546795	-1.53025231832885	-5.66180412073619
H	2.37641219698086	-7.87136715374815	-4.13434800543469
C	-2.05375099988521	-0.47424386241141	-4.91998901881994
H	-1.58015634684811	-2.43846641938893	-4.19351534481608
C	-0.28858185567559	1.14172451423489	-4.85246601519801
H	1.60484516251350	0.46141787878176	-4.11018067840824
H	-5.40034453068197	3.85082120705660	-0.93616562668286
C	-2.87407519731384	4.59865267367147	-0.30239584103632
C	-1.61079777597212	3.37091928038419	-1.93282568765014
H	-7.76203452276240	3.29956921252805	-0.70049677635395
C	-8.55349503321514	1.30238126091760	-0.78704271915168
H	-9.04868378048140	-0.81180972817116	-0.85775141648870
F	8.02734619924942	-0.43743549910677	-3.29740793764603
C	-1.62006596220926	0.83969687523289	-5.16695563627334
H	-3.08776114909841	-0.75602281480562	-5.12379739451927
H	0.09824130420707	2.14749162113321	-5.01321532948814
C	-1.93774892818477	5.62956101366523	-0.35597313130736
H	-3.71638606762871	4.67911723260392	0.38703689974348
C	-0.68099703461120	4.40531582081422	-1.98032745427802
H	-1.45354117303386	2.49590676626395	-2.56117786419379
H	-9.58563848860976	1.61788337813745	-0.62800442404978
C	6.83826566784533	0.19915340735605	-3.43772103258866
C	-2.58003170253289	1.86030544424936	-5.78778254500023
C	-0.82422228842395	5.56878086928634	-1.20785275934633
H	-2.08320541006924	6.49356954306104	0.29201366754475
H	0.17804474620923	4.28860612851049	-2.64107011091075
F	6.03056724603364	-0.59717955644897	-4.19120169677407
C	6.20455867907320	0.54376858788960	-2.11219409573763
F	7.06973047065513	1.32481676109092	-4.17241896846381
C	-2.97783839707556	1.36146550882362	-7.19318612993400
C	-1.94627879344386	3.25204398969043	-5.92274892769874
C	-3.84886656764291	1.98873956133109	-4.92253676357553
C	0.15186557484358	6.74129929154081	-1.35682703171321
C	6.92707325344677	0.44678233890337	-0.92519484765809
C	4.88696245470546	1.01360570579810	-2.10663629416041
H	-2.09283408312760	1.25923545056864	-7.83787454139046
H	-3.47627172232706	0.38315905644094	-7.14361124566055
H	-3.67039996299112	2.07402833069660	-7.66662590178618
H	-2.67566784206504	3.94353601941646	-6.36872370784127
H	-1.65432542215286	3.66266799651977	-4.94523883349723
H	-1.06077258125232	3.23726902819523	-6.57487425368677
H	-4.55442767484985	2.69024485145523	-5.39281417506588
H	-4.35866446079685	1.02356509779050	-4.80658646605205
H	-3.61034061574467	2.36858559804159	-3.92045392050849
C	-0.31487316630994	7.58560746714664	-2.56327769780103
C	1.58566563526804	6.24621004638663	-1.62176353890787
C	0.17384436973373	7.63761165793935	-0.10717960844508

C	6.32948649639365	0.83259876244295	0.28035191250346
H	7.94584353550821	0.06341546638995	-0.93423197652707
C	4.31752369538687	1.39038110377983	-0.89702899299258
H	4.30759215079420	1.08890734166252	-3.02623827223202
H	-1.33031774363959	7.97497029404039	-2.40045606246183
H	0.36285934891505	8.43961501405318	-2.71743685312966
H	-0.32577461414175	6.98281423096143	-3.48287406215237
H	2.27267500576509	7.10491507455205	-1.64759425334495
H	1.92403473761931	5.55405309914134	-0.84016699626383
H	1.67330855706400	5.72847062955238	-2.58665754453833
H	0.44990749559099	7.07486674185189	0.79564652293225
H	0.91870674990883	8.43529514385501	-0.24139403461360
H	-0.79534206509017	8.12417808786227	0.07028362073466
C	5.02174724390081	1.31085475313562	0.30646632921710
C	7.13397118870000	0.80085867998521	1.55690770954343
H	4.54775956568456	1.61489770605731	1.24177481524144
F	8.05963884618941	-0.19594000949911	1.55599908241291
F	6.34778338338526	0.62377657836097	2.65175037691484
F	7.81192976016784	1.96851098501245	1.73929804202926
C	-4.45470200555159	1.78131066912032	2.61440272342924
C	-3.85821619762493	0.52142147066586	2.58412410662079
H	-3.83295462664593	2.67355354538826	2.68970670988822
C	-5.84680053507804	1.87522574543425	2.56946402229337
C	-4.63031527340890	-0.63386975802180	2.51140611533960
C	-6.63747298776590	0.72714107603781	2.48062076686674
C	-6.48741658497098	3.23417559601036	2.68855629194150
C	-6.02154244084400	-0.52346534522047	2.45028443048705
H	-4.14024670354834	-1.60613407608695	2.50933156638703
H	-7.72095193849841	0.80838330138544	2.42787341389893
F	-6.39634416773993	3.71478586981282	3.96168410047377
F	-7.80569151447605	3.22269335599451	2.36268971510721
F	-5.88259002576021	4.15809966899770	1.88483209035505
C	-6.83830215889297	-1.78860315927089	2.39392872339727
F	-6.76547898441272	-2.47908745438775	3.56897378105098
F	-8.15358613731980	-1.55369735848910	2.15206717809180
F	-6.39108892565520	-2.63468975775104	1.42169674710629
H	1.66436869061040	2.72361201680004	2.26607714259752
H	2.42086622413232	0.59832185804270	3.99030438557102
H	2.87910362219813	3.00124562628049	4.71414274093255
H	-0.20310124450467	3.10687983596013	4.72501274312807

228

B-TS1

C	0.84254749508035	4.36555222593391	3.25254646130008
C	0.53106619723980	3.67611207632331	4.52485504489376
C	1.60171545840620	2.56873348912594	4.82364621953247
C	1.67199390295123	1.41625074099544	3.82840877771208
O	2.44606953121083	1.83664896346008	2.68973527664897
H	0.55233987116445	4.37599949233569	5.37099951247415
H	1.32931522726755	2.16513345252105	5.80983206942439
H	0.65921596713428	1.10643268222033	3.52759683396981
C	1.75274991363119	5.48839933387871	3.24161523670107
C	1.86359762442179	6.36617124324198	4.34611395956284
C	2.58317583102548	5.69945388485830	2.11376340272757
C	2.74814511374173	7.43579686252865	4.30754760328000
H	1.21439200235315	6.23816464558175	5.21175534875957
C	3.49895047254352	6.74273894447558	2.10643237146303
H	2.55299096468561	4.99415466301762	1.28335099969593
C	3.57395734224810	7.62030229338381	3.19272028221249

H	2.80178988961899	8.12730940441441	5.14891314115349
H	4.15511757158625	6.87466617480517	1.24561523615276
H	4.28060935553735	8.45158186527208	3.17356460371138
C	0.38759807403213	3.76622477706698	2.03345269981893
H	-0.50665040768605	3.13909632579187	2.13748791552330
H	0.40524557967166	4.38202746155696	1.13165715499729
H	1.19226050054971	-7.86461103346943	0.67756303272349
C	1.00021617460326	-7.70023078953147	1.74706555384818
C	1.19806994280361	-6.21923195928584	2.13411903042938
H	1.69135456012345	-8.33759107853600	2.31968383846121
H	-0.02807850501608	-8.02634741518937	1.96056995190846
C	0.21717273080833	-5.36908699269126	1.31893859120230
C	2.64743057478267	-5.80390260988306	1.81302068682831
C	0.97869233530950	-6.07802927260382	3.64687805605996
C	3.90940530088653	-1.50141311746113	3.98744807521966
C	0.28935720638003	-5.37064368768662	-0.08410130289965
C	-0.79259821942546	-4.59298412900011	1.90379214682989
H	2.86522221210388	-5.89373592434374	0.74048871410077
H	2.83476395385298	-4.76461486486054	2.11267831147666
H	3.35385595321608	-6.44829403765544	2.35834491483863
H	1.13161051569968	-5.04336189400624	3.98518503433022
H	-0.03065193634165	-6.39838430028597	3.94435289145806
H	1.70036674326681	-6.71279453094692	4.18149795438728
C	3.17511498285571	-1.95705033756526	2.87909464440793
C	3.33679661854416	-1.42520938457170	5.25839712052671
H	4.94591823030966	-1.18704391540496	3.85754549555032
C	-0.62269670721300	-4.66500216340404	-0.85965635191526
H	1.07579823956629	-5.93175640873277	-0.59104334036902
C	-1.70666982552121	-3.87456033384931	1.13181371912101
H	-0.88496709179956	-4.54380082152485	2.98842895166572
C	3.83969262198352	-2.19059931965450	1.57395400734394
C	1.82469307579567	-2.29217083438619	3.10111388835469
C	1.99624594793508	-1.77290322900536	5.48100578056003
H	3.95411649774389	-1.06786716478244	6.08260270981625
C	-1.65443731091811	-3.91668399565703	-0.26829557307485
H	-0.52076815000405	-4.67311547413291	-1.94572967658208
H	-2.47523722918104	-3.28792893189557	1.63240222583430
C	3.13341191048477	-2.19812548270901	0.33282629317798
C	5.18266904457799	-2.53502558390563	1.52138876169612
C	1.25765157527942	-2.19324711194639	4.36526162188445
H	1.20409346364628	-2.65380317335160	2.28593689884955
C	1.31990539384089	-1.70151435848495	6.85199241799712
C	-2.66326206716877	-3.26123427946702	-1.12949628125070
O	1.80034074362628	-1.79746216083423	0.34431688191694
C	3.67653507495097	-2.67303269786263	-0.85652974202081
H	5.75739738453800	-2.58441653514042	2.44692857186349
C	5.84161729999141	-2.84665908966949	0.31078341895264
H	0.20108375080173	-2.44358686929381	4.46951036287970
C	0.85278214095676	-3.11802799639127	7.24856104029114
C	0.09421133671673	-0.76762557816812	6.77312029957431
C	2.26472936125144	-1.17711637350331	7.94126579941829
C	-3.14631124507568	-1.94481752587836	-0.89154009265116
C	-3.17280455927926	-3.92064740575423	-2.23645940033922
P	1.32026169282907	-0.49844148643278	-0.50664917433367
C	5.08348255425423	-2.93128521200146	-0.90291751948641
C	2.79170287798774	-2.96151257677257	-2.01736073188610
C	7.23812310098801	-3.10101309208767	0.27166988376298
H	0.35761715290556	-3.09429925017891	8.23116151785627

H	0.13909415587406	-3.52406963922400	6.51874468635921
H	1.70630362857164	-3.80890532699767	7.30746677509722
H	-0.43049563270632	-0.75045774999702	7.74034882902518
H	0.40069151667180	0.26089997775924	6.53406869908208
H	-0.61988794218508	-1.08230601139062	6.00063182522071
H	3.14165646993721	-1.82836879469091	8.07019018479222
H	2.62093165520954	-0.16076943367199	7.71546015938242
H	1.73349337895095	-1.13866785310244	8.90319083932033
O	-2.64508343021876	-1.25334950583361	0.21376911334012
C	-4.08193816883601	-1.30293525949953	-1.69095236066332
H	-2.84585105313192	-4.94228992757628	-2.43999673379859
C	-4.10037312330218	-3.31703409328987	-3.11679250213559
O	2.03829619577083	-0.71146888447774	-1.94296259471209
N	-0.22546310763056	-0.70593830111797	-0.61226565557776
N	1.93442489280610	0.85158106378650	0.11601812907044
C	5.76451559138590	-3.22997773452121	-2.11061718752988
C	2.69873709569998	-4.28328092656919	-2.56797302466290
C	1.95692350410555	-1.97928122038744	-2.52344953854746
H	7.80560203927562	-3.03737233699607	1.20206087717608
C	7.87052605638240	-3.39347752218318	-0.91749317770876
P	-1.55177339960644	-0.07805308326260	-0.05790760991444
C	-4.48198770093182	0.09720099956740	-1.38984189571586
C	-4.56575001626061	-1.98479951789957	-2.85601634292123
C	-4.55646937628374	-3.99738291508362	-4.27740676602253
S	2.68016377786361	1.98882562082541	-0.75582122798029
H	2.26509986435855	1.23283304510335	1.92258458336710
C	7.12664776179720	-3.44849440995475	-2.11859084253206
H	5.19994945515494	-3.26206576947677	-3.04187758923099
C	3.42503866673319	-5.39150578358634	-2.05858796332338
C	1.78127444637073	-4.51209627996958	-3.64821548291941
C	1.03030355184271	-2.17665967277049	-3.58491288266732
H	8.94645127129871	-3.57314143123948	-0.93645162497593
O	-2.16952837565687	0.68286096345060	-1.35301936542422
N	-1.55354208227732	0.89791898384828	1.20354371406172
C	-3.50391092695047	1.07729198612789	-1.27942016034976
C	-5.85049052596393	0.48160513756337	-1.22097986419648
C	-5.43989965524637	-1.38373463838444	-3.79970250746033
H	-4.19954969333884	-5.01397751683904	-4.45630141309388
C	-5.41440954185833	-3.38847882361329	-5.16691312206923
O	2.81047208594556	3.17026758803714	0.09970444715269
O	2.13454547216467	2.17800866058695	-2.09574185489000
H	7.63514834682077	-3.65242623438151	-3.06170377582390
H	4.10394480752750	-5.24246514058888	-1.22105569675965
C	3.27383365217963	-6.65248841586198	-2.59871935978715
C	1.66249534111407	-5.81916850309473	-4.19170700554809
C	0.98429885059358	-3.44845613958124	-4.13305207278164
C	0.13778459138535	-1.10472146223375	-4.07716818286803
S	-2.11871310074629	0.56871759346455	2.66007841564659
C	-3.78246819264702	2.46590522519107	-1.13332836864046
C	-6.16236069027525	1.87396829572105	-1.07242096942585
C	-6.90832667322303	-0.46103220921060	-1.15310059021238
C	-5.85151749083356	-2.06599540983433	-4.92633743762077
H	-5.78159615356082	-0.36396449513200	-3.63229138981332
H	-5.75061621097456	-3.91957024149059	-6.05852729581940
H	3.83738625955623	-7.48844275367779	-2.18143166795226
C	2.39149902754798	-6.87079444419980	-3.68090084577777
H	0.96620594597671	-5.97531258166106	-5.01852472491191
H	0.30582675199994	-3.64109575177109	-4.96651989245565

C	-1.19499031585187	-1.40408506679769	-4.40851101821137
C	0.57315022837059	0.21458679442879	-4.26230233260596
O	-1.77407834006988	-0.76502557613867	3.15223473317578
O	-1.78321973626991	1.71655768402437	3.51372616989005
C	-5.11896174532399	2.82793577380109	-1.06479785821463
C	-2.71819799602181	3.49378002351205	-1.09508627590544
C	-7.51879005816559	2.26498220285623	-0.91910591765622
C	-8.21377726393249	-0.04943694021682	-0.97970709879701
H	-6.67543136859588	-1.52295113844835	-1.22149416939290
H	-6.51669450049108	-1.57671868391898	-5.63957986483227
H	2.28382911023865	-7.87105260557184	-4.10274075826466
C	-2.04138120303721	-0.43190305617384	-4.92842485824745
H	-1.58270473284880	-2.40770171583074	-4.22715724985582
C	-0.28296030625309	1.18482662819719	-4.78336673447869
H	1.58863743702128	0.50000021988039	-3.99646594109347
H	-5.38242461763157	3.88428607411668	-0.99536427433416
C	-2.88090473106879	4.64719445395925	-0.30983724208227
C	-1.54881924202335	3.40000148604934	-1.86919597163857
H	-7.74522675209944	3.32742809949699	-0.81082323274088
C	-8.52592607639925	1.32522075552335	-0.87395525749141
H	-9.00920229177655	-0.79274454395475	-0.91130629866366
F	7.80697899059933	-0.54579279820956	-3.66368312473886
C	-1.60456549711468	0.88695637794540	-5.13962926365266
H	-3.06947527010536	-0.71225874467440	-5.16238289386380
H	0.10654171589269	2.19431520332224	-4.91169776671431
C	-1.94131005747180	5.67582089252612	-0.33313046196079
H	-3.75450866717779	4.73680226383142	0.33872349269710
C	-0.61026538998847	4.42816513108959	-1.88091727939805
H	-1.36323748095329	2.51867958428714	-2.47961285653348
H	-9.56235393060765	1.63879874024905	-0.74098037740620
C	6.60855514159227	0.09177801441419	-3.69513546410745
C	-2.55079844714145	1.91754567901632	-5.76531429781978
C	-0.78791301418942	5.60090378845919	-1.13123872044892
H	-2.12033974513941	6.55298322504776	0.28931485215013
H	0.28336085843240	4.29137695991691	-2.48893471730088
F	5.73162343070042	-0.72046987045448	-4.34927585085544
C	6.11086654208103	0.46399111020965	-2.32184598751587
F	6.76731654302064	1.19936344417770	-4.47795089440614
C	-2.91464336659049	1.44540497397985	-7.18891214392460
C	-1.91568671293539	3.31203093702814	-5.86027771727719
C	-3.84010918597458	2.02958669773759	-4.92870737993878
C	0.19068164177926	6.77442314359203	-1.24805336243898
C	6.93847331576714	0.35121610820002	-1.20633479976198
C	4.81126756265265	0.96589740141879	-2.19791467925371
H	-2.01414397376658	1.35605442037455	-7.81373424327467
H	-3.41177464985494	0.46543057147597	-7.16946321688394
H	-3.59723098653413	2.16551367632898	-7.66585473604795
H	-2.63456582162145	4.01103531305642	-6.31204842729673
H	-1.64695218985770	3.70489886115139	-4.86888363370974
H	-1.01408394827541	3.30825588117074	-6.49002748001138
H	-4.53456948419681	2.74150360882338	-5.40029280767522
H	-4.35240802255719	1.06220792487389	-4.84528402272653
H	-3.62495538284880	2.38668093713918	-3.91296479797560
C	-0.30179295044769	7.68365875504372	-2.39571844153270
C	1.61433398678946	6.28975806213023	-1.57781685462463
C	0.24110100321088	7.60495824913656	0.04631802689822
C	6.45726778931793	0.74781135650175	0.04529516255273
H	7.94363287493329	-0.05305792331773	-1.30603639797916

C	4.35086595073572	1.34963582556455	-0.94375294556244
H	4.15495733758332	1.06088062776423	-3.06237364089420
H	-1.31024010005031	8.06887659822250	-2.18656284767540
H	0.37706995813829	8.54100729842211	-2.52537362183849
H	-0.34057405810130	7.12739304429858	-3.34303678845096
H	2.31054800035192	7.14114099448271	-1.54748443565096
H	1.96426337766896	5.52455192006058	-0.87162004410695
H	1.67687065405715	5.85431687845817	-2.58405075351460
H	0.51061354371240	6.98651099804401	0.91396541612451
H	0.99849693408230	8.39665203742213	-0.05048446562961
H	-0.71809193593823	8.09591205568989	0.26087289219515
C	5.16272567553059	1.24635459803293	0.18704121121765
C	7.35951381791792	0.70986984742983	1.25168939849109
H	4.78275212798432	1.55260804399677	1.16314371573927
F	8.41622712332457	-0.12865818131547	1.08923356900387
F	6.69796361559221	0.31392788721079	2.37564915270181
F	7.87514692686958	1.94580769759637	1.52250470106292
C	-4.56780220850351	1.84332943879399	2.53968098600684
C	-3.91505178324776	0.61194635146262	2.52754493571879
H	-3.98609096380794	2.76183251812129	2.61345227047760
C	-5.96228209121883	1.87694977193619	2.48462870304833
C	-4.63572045182724	-0.57670968633939	2.45708094407848
C	-6.70109817526271	0.69517564824942	2.39827882169742
C	-6.65802354289136	3.20839403216073	2.59530715935439
C	-6.02962199140168	-0.52720521779290	2.38110138352911
H	-4.10469712734851	-1.52703569501608	2.46960525880629
H	-7.78663888293693	0.72775647198139	2.33730422664460
F	-6.58024609470062	3.70301407322159	3.86605988922172
F	-7.97554944560813	3.14299372998015	2.27728801428695
F	-6.09339508386758	4.15242001795315	1.78436130405486
C	-6.79017521543354	-1.82713429877250	2.32661152861002
F	-6.70307742682482	-2.50285649053634	3.50956692612146
F	-8.11163993653193	-1.65091722483343	2.06684757938151
F	-6.29541645167762	-2.66151281680840	1.36852705371793
H	1.24119001685831	2.99403642352447	1.93026954857246
H	2.15866248093267	0.55725446003356	4.30987289230058
H	2.59824980263872	3.02736201270252	4.91649721506183
H	-0.44770214158385	3.18346584924476	4.45952786371196

228

B-P

C	1.14714952298416	3.52490426761037	2.91777128196798
C	0.58410900255641	3.48482351001895	4.35566717542209
C	1.19684413641700	2.21765061804878	4.95072902515161
C	1.19111545915148	1.26771918624557	3.76082369316137
O	1.37404165861953	2.10984002709710	2.58993312471724
H	0.84284842378751	4.39574533779736	4.90990346124097
H	0.60665158812555	1.82575489901143	5.78797737271794
H	0.23480295884776	0.73574043779054	3.67296596256122
C	2.49433765427531	4.24292356605915	2.89330879150445
C	3.68781636760560	3.54058564933150	3.10945519624327
C	2.57006185633950	5.62842276468138	2.70016996039317
C	4.91947542425524	4.19702061446428	3.11930644042214
H	3.64948973665710	2.45974304764711	3.25147604778926
C	3.79987692702661	6.28857724312995	2.70377842986644
H	1.65928516044748	6.20438598310838	2.53172652634040
C	4.98192107569410	5.57584099900259	2.91070931164527
H	5.83585959192732	3.62674323536522	3.28409233238159
H	3.83288559272609	7.36666120037970	2.53638781330055

H	5.94413947059397	6.09007536068896	2.90789816438715
C	0.13166452821020	4.07785857932027	1.92573147729289
H	-0.75692839245633	3.43518186258631	1.92802702330526
H	-0.17978705894053	5.09121018651815	2.21367627879910
H	1.22782906539888	-7.75840479297854	0.81846224550531
C	1.03908247549614	-7.57512611796663	1.88542649927796
C	1.23032817452906	-6.08629223093537	2.24570278934407
H	1.73540045894941	-8.19837436494623	2.46703046809687
H	0.01338913294037	-7.90242319505883	2.10879729380624
C	0.24143361333188	-5.25706277141791	1.41816739336607
C	2.67693685790622	-5.67066962898830	1.91097030507550
C	1.01442006516721	-5.92336457432679	3.75662930054072
C	3.92620863458933	-1.11876896883543	4.08087491377908
C	0.30654300673019	-5.29150344134584	0.01507747592186
C	-0.77258874867540	-4.47497644268629	1.98797702838182
H	2.89021324252985	-5.78413154261489	0.83978101325536
H	2.86234735646457	-4.62392742202568	2.18651181830062
H	3.38840569590164	-6.29943567827462	2.46764015447562
H	1.17122250829442	-4.88551741033982	4.08179394225932
H	0.00500224088094	-6.23724972033239	4.06004181281343
H	1.73560617660279	-6.55213293477280	4.29874746126552
C	3.22323018730730	-1.70983659760900	3.01760400316392
C	3.37235684543455	-1.03947213697651	5.35937332196380
H	4.91717687025353	-0.69676480784581	3.90465196076595
C	-0.61996292988938	-4.61859807365743	-0.77148813082693
H	1.09605943877386	-5.85660594405496	-0.48257885601998
C	-1.70236209521805	-3.78829182115440	1.20411329915569
H	-0.86133679334646	-4.39946576727051	3.07158709468864
C	3.87164806571699	-1.92165132467632	1.70352752901032
C	1.92905745538846	-2.19204459558101	3.28832813335221
C	2.09141992444870	-1.54026735510611	5.63718842381200
H	3.95600802324463	-0.56388172866199	6.14701119021369
C	-1.66090635434805	-3.86981178733288	-0.19504346596542
H	-0.52537310331765	-4.65626927108808	-1.85739448403431
H	-2.47059563902376	-3.19498710169542	1.69701313029654
C	3.14206096256436	-1.96264976218539	0.47886836201501
C	5.22544146236301	-2.21336704209162	1.62876971337997
C	1.38628513259304	-2.10645146313640	4.56425897784530
H	1.33621044035702	-2.65416052278337	2.50192146123476
C	1.47920616308624	-1.53608298123623	7.04060486910220
C	-2.67790040407877	-3.25663929791088	-1.07915499154861
O	1.79043664854599	-1.60432891901615	0.52761711543978
C	3.67404920407745	-2.43035823977667	-0.71689875802569
H	5.81568026265506	-2.22925399318591	2.54609032798550
C	5.86870605984840	-2.52189999100462	0.40906274344223
H	0.37646171339156	-2.48898223335769	4.71640401214495
C	1.50706944434903	-2.98670219817802	7.57064010761807
C	0.01838819631641	-1.04601918084774	6.99928166418737
C	2.26242852867220	-0.63990647944411	8.01061609685269
C	-3.20068837636243	-1.94894552496114	-0.87976980785495
C	-3.14730555937654	-3.95024111867528	-2.18400156365154
P	1.19730213753486	-0.39617263830448	-0.36343774579607
C	5.08801025341334	-2.64927979363345	-0.78664139874185
C	2.78393275888589	-2.75800767453622	-1.86192588297852
C	7.26994497751822	-2.74329748847119	0.34647628980171
H	1.07160193389519	-3.03135105194904	8.58043521251851
H	0.93009012380726	-3.65792076993692	6.91865117391729
H	2.53787996567036	-3.36654544281523	7.62065317688795

H	-0.40892022148515	-1.07133601209033	8.01256206392848
H	-0.04683218143480	-0.01459381740933	6.62817330006918
H	-0.61610409570685	-1.66862566688667	6.35553734841300
H	3.29072481229418	-0.99786050512376	8.16411600179187
H	2.30670184311914	0.39932596981814	7.65281102049573
H	1.76575589109274	-0.63757040429962	8.99133252069064
O	-2.77096922649620	-1.23668760327685	0.24287711431758
C	-4.11346353702716	-1.33638184516631	-1.72735479481904
H	-2.79726905850480	-4.96990318325087	-2.35605441333987
C	-4.06096252669408	-3.38457792737380	-3.10271239202863
O	1.93562716086164	-0.53384007232314	-1.78110674776313
N	-0.32396389146052	-0.64099973592032	-0.38538974649620
N	1.80055590637373	1.06784299793924	0.13787764447909
C	5.75239423383641	-2.95494876275854	-2.00192657569902
C	2.73142229801310	-4.08689132277793	-2.40245595980064
C	1.91421749629912	-1.81147893660192	-2.37368968731108
H	7.85364101122632	-2.64991410828957	1.26404276324547
C	7.88471930372787	-3.04758589531728	-0.84883215821531
P	-1.71946670595741	-0.01908187058954	0.03249899107011
C	-4.54147541596180	0.06483646931227	-1.47319534156212
C	-4.54786199150104	-2.05131895069212	-2.89199907921820
C	-4.47708455174242	-4.10241637752190	-4.25606174368854
S	2.64353219648346	2.18637990588399	-0.81060361156259
H	1.54579135029196	1.46758373036438	1.09986609688442
C	7.11841172225208	-3.14479108614021	-2.03276329043735
H	5.17196583837433	-3.01995632006098	-2.92135764697611
C	3.49021381829517	-5.16784005239446	-1.88280635053052
C	1.82190265225080	-4.35153834087021	-3.48064808174784
C	1.00426201108263	-2.03764064554472	-3.44129062843673
H	8.96342724940185	-3.20597527295189	-0.88580528699356
O	-2.23395442484478	0.68011994731623	-1.33505458814432
N	-1.81072526944657	0.98059183270736	1.25482693427541
C	-3.58211700224988	1.06111736677467	-1.34331867831592
C	-5.92077964912177	0.43464953669579	-1.36628495763480
C	-5.39667775029439	-1.48452005625770	-3.87937113177653
H	-4.10639954470509	-5.12029045230113	-4.39524465159192
C	-5.31432172013513	-3.52750095091382	-5.18662845938840
O	2.73469950408131	3.36436393977256	0.02415803031975
O	2.08812128140156	2.25007478476559	-2.14692682290241
H	7.61215453109596	-3.35902653743045	-2.98124136779606
H	4.16249885797058	-4.99275124244574	-1.04522492050252
C	3.37596002231102	-6.43709465040282	-2.41202707748167
C	1.74101570662407	-5.66619341292469	-4.01276893116642
C	0.99851567860772	-3.31503024231600	-3.97855211615206
C	0.09876753732193	-0.99329724862374	-3.96589773419960
S	-2.41680364214911	0.64455456857410	2.70659254201034
C	-3.88202923907694	2.44730838504654	-1.23388407160226
C	-6.25492296712597	1.82493024052549	-1.25181903972363
C	-6.96910948951632	-0.51999808342235	-1.32361880840909
C	-5.76850406125274	-2.20227988823536	-4.99757152682625
H	-5.75294595413088	-0.46410772711451	-3.75070105035844
H	-5.62091311833291	-4.08725768767501	-6.07130820825529
H	3.96265825688556	-7.25274460179794	-1.98681354245670
C	2.50060868735203	-6.69073740798622	-3.49219432283830
H	1.05020468712697	-5.85004407789069	-4.83825992987434
H	0.33147424847673	-3.53081166571493	-4.81537269912663
C	-1.22809094937526	-1.31843408570596	-4.29438725663943
C	0.52806791112039	0.31845328955577	-4.20604028622588

O	-2.06225354394475	-0.69348045710093	3.18336409140639
O	-2.13340756263942	1.78796673714268	3.56894998684096
C	-5.22446020020411	2.79229779826185	-1.21901907501859
C	-2.83480842137918	3.49098230403478	-1.17798067063340
C	-7.62086040974780	2.20138293985299	-1.15847230837726
C	-8.28531408792750	-0.12203090602186	-1.21025544507713
H	-6.72206306482109	-1.58011730989505	-1.36231239723347
H	-6.41639325376052	-1.73975720314511	-5.74375744938119
H	2.42261845261163	-7.69749198136965	-3.90475432327163
C	-2.07501390234505	-0.37543991070327	-4.86441465091445
H	-1.60852776244678	-2.31745696267573	-4.07592592319295
C	-0.32899894171558	1.25949246512517	-4.77635995544869
H	1.54341346448893	0.61718671569227	-3.95390496956998
H	-5.50080530758675	3.84669487522564	-1.17308596549128
C	-3.01600664497857	4.62826578223413	-0.37740660261430
C	-1.66964574190272	3.42845205003115	-1.96293424840604
H	-7.86359983990352	3.26233058645325	-1.07479639895254
C	-8.61773124206798	1.24999964428107	-1.13989777326060
H	-9.07372335448085	-0.87415863046414	-1.16032325612674
F	7.85707894852603	-0.33253049953792	-3.54013319981701
C	-1.64565945266346	0.93595793445905	-5.12969486951117
H	-3.09750752514585	-0.67390065069562	-5.09866492950060
H	0.05413084706256	2.26472287732837	-4.94902220991685
C	-2.09213240722240	5.67282864943058	-0.38638419459012
H	-3.88860508870717	4.69170783911638	0.27569011869437
C	-0.75667864113073	4.47763734671445	-1.97010336429238
H	-1.47683195516298	2.55778650305908	-2.58753782972088
H	-9.66223874054756	1.55265756846294	-1.05314991941027
C	6.64457413273092	0.27135651120785	-3.61096643070758
C	-2.59255720627886	1.92946890147319	-5.81149615696526
C	-0.94676574734427	5.63254599631924	-1.19350780391808
H	-2.27665088154712	6.53145760449203	0.25855565457312
H	0.12901608589547	4.38259152747940	-2.59980430180088
F	5.80555638337694	-0.56636632452652	-4.28081735077552
C	6.10032167052063	0.64199220743619	-2.25303810865511
F	6.79386260851725	1.37904603825118	-4.39238271137752
C	-2.92521663180514	1.39377197875403	-7.22027040968506
C	-1.97122483854036	3.32629592897438	-5.95153607218122
C	-3.89792806773267	2.06023279044173	-5.00257322142138
C	0.03955262341305	6.80165136258857	-1.29097785877015
C	6.91605002364327	0.61662535484657	-1.12302589636469
C	4.77469570215697	1.07739492488863	-2.16420248648768
H	-2.01314801399267	1.28983130024746	-7.82576811665154
H	-3.41176739906444	0.40959063465302	-7.16832650713791
H	-3.60731385217068	2.08534695441082	-7.73793063129771
H	-2.69080569308728	3.99865802812315	-6.44060724058643
H	-1.72251006617705	3.76094852388893	-4.97242029536625
H	-1.06081894914283	3.30934545955648	-6.56832986840351
H	-4.59051698862529	2.74381281269601	-5.51641475285381
H	-4.40291504845595	1.09203945280098	-4.88836241339701
H	-3.70610554636033	2.46226034960628	-3.99922262278432
C	-0.14828194079049	7.46521095820725	-2.67274555613178
C	1.49354466895137	6.30911082182104	-1.16393002253630
C	-0.20464346257691	7.85780171959646	-0.20322550647062
C	6.40177103611123	1.03869932464675	0.10707665754816
H	7.94325789325752	0.26494595280039	-1.19683382808667
C	4.28874776037010	1.49557147912839	-0.93104161202225
H	4.12843700251790	1.10847646482547	-3.04057548054151

H	-1.17682853067361	7.83502673083040	-2.79459906099513
H	0.54202699999086	8.31542245996068	-2.78513487029726
H	0.05307858780863	6.75102476369335	-3.48370029649881
H	2.18283966308463	7.16080663840355	-1.26698457505687
H	1.67859835098836	5.83697139182107	-0.19067626099680
H	1.75288249005059	5.57628334025179	-1.93912426174584
H	-0.12688762491409	7.42340161620196	0.80457836127838
H	0.55079156919440	8.65226873372470	-0.28705975241798
H	-1.19305605431822	8.32996081228267	-0.29936469112913
C	5.08734861360147	1.48842612769570	0.21222393414802
C	7.29215555572156	1.09287065096308	1.32357173638216
H	4.69074927730626	1.85455799247198	1.15963709336837
F	8.33920981600061	0.23266756735858	1.23869108640543
F	6.61048758768925	0.79181809525738	2.46547799486280
F	7.81473975740991	2.33952312989121	1.49860051939601
C	-4.87467362176296	1.87340436032706	2.45181940406594
C	-4.20355078336855	0.65205357796087	2.48199988631230
H	-4.31060161236992	2.80089866501518	2.55015429239237
C	-6.26454802265435	1.88352673460943	2.32304100021026
C	-4.89992740773343	-0.54896924919322	2.38591527860829
C	-6.97922192794608	0.68890844174695	2.21174929845163
C	-6.98543494896543	3.20577033736783	2.37444607517191
C	-6.28917131551164	-0.52282444131024	2.24133340096905
H	-4.35498172050861	-1.49042442664785	2.43287203249906
H	-8.06070173429414	0.70364871894764	2.09652548166708
F	-6.96687822108782	3.73184364538829	3.63288859914829
F	-8.28841177894743	3.10891302590055	2.00481248180867
F	-6.40416609308632	4.13719730104558	1.56108793586643
C	-7.02561329468244	-1.83507180832311	2.16413978504639
F	-6.99259878571468	-2.49496184390355	3.35828221943582
F	-8.33353543175966	-1.68423169778866	1.83028763991795
F	-6.46659319800455	-2.67434309469358	1.24496594576837
H	0.54182593899303	4.11991167164487	0.91112234457054
H	2.01201743571158	0.54344155751540	3.77475684370852
H	2.22290469341560	2.39764544012159	5.30421211470327
H	-0.50801319365291	3.38520644035987	4.30783989961415

228

C-E

C	-1.17352706931966	4.19705386106581	-0.73799980957834
C	0.15617859306020	4.56722232043343	-0.13184307271453
C	0.81223246548997	5.75674711991026	-0.85209815314258
C	2.25528293519465	5.99310113947343	-0.41896976290904
O	3.10834468613417	4.87478046399243	-0.67007930514386
H	0.05194852980404	4.79395565680801	0.93903337183862
H	0.23320368594131	6.67974229734288	-0.68586422530789
H	2.29928379638619	6.19341138493510	0.66587051477398
C	-2.35846593890963	5.05032323732281	-0.48044979116193
C	-3.52327195028081	4.94177715266047	-1.26672612998643
C	-2.36885250917808	5.98530471299659	0.56934990659857
C	-4.65594170771405	5.70261088040146	-0.98965990062425
H	-3.53807603245731	4.25743142600444	-2.11480460015179
C	-3.50099186121560	6.75072188779784	0.84674044807506
H	-1.48749234027403	6.10099307792465	1.19769938763803
C	-4.65459195863783	6.60851617817086	0.07501545917601
H	-5.54659412333711	5.58949594164105	-1.60993812538832
H	-3.48128711882126	7.45854330778177	1.67717613187994
H	-5.54297238633078	7.20270248644483	0.29397952281569
C	-1.24608212428995	3.10340492710800	-1.53002412912428

H	-2.16449658952941	2.79635894405305	-2.02615149463298
H	-0.34069097502790	2.54622237817805	-1.76870459522894
H	-3.56315433465118	-2.32960187437761	-3.84247666932574
C	-3.56031977412501	-3.35626662517601	-4.23191723747109
C	-2.21684058749573	-3.69001661463074	-4.90861914596810
H	-4.38147392889571	-3.44552203340928	-4.95933138492153
H	-3.77152383384357	-4.03787327608187	-3.39761406876772
C	-1.08000923530897	-3.62278804833543	-3.88286991962843
C	-2.00955328122342	-2.71559851686619	-6.07653894303556
C	-2.28113088301780	-5.12550424569556	-5.47199890158565
C	-4.04679861598104	1.80923229142811	-3.49219826699360
C	0.05313009006314	-2.81702155799898	-4.05554753668376
C	-1.12756052082293	-4.41777813499433	-2.72465653416271
H	-1.97006466220371	-1.67114480218814	-5.73493322683684
H	-1.08675118601772	-2.93515829606231	-6.63280452728192
H	-2.85039400654511	-2.80407112685171	-6.77971481599630
H	-2.47365720362910	-5.85927488355321	-4.67668647064552
H	-3.08958645864770	-5.20646529024741	-6.21463761434728
H	-1.33437016588590	-5.39661075822466	-5.96102603848816
C	-3.43391402491310	0.62568905639643	-3.03620694289964
C	-3.39429006558518	2.66375637804296	-4.37423440762287
H	-5.03210029040444	2.08716851038364	-3.11627037951985
C	1.10215835644044	-2.81793379236827	-3.13604836065630
H	0.13840809824937	-2.17011571229917	-4.92805014178952
C	-0.08525015361098	-4.42486037400362	-1.80447086133145
H	-1.99819585052089	-5.04539788664916	-2.52733065847548
C	-4.14146969941719	-0.25841207897824	-2.08153996072265
C	-2.16032581938047	0.32498802804778	-3.55045740526121
C	-2.09106791788851	2.39981099943217	-4.83218639123028
H	-3.89995451402167	3.58169377435784	-4.67994777971988
C	1.06142847715474	-3.63491999186528	-1.99744683081703
H	1.95851037445950	-2.17184478140529	-3.31801711422263
H	-0.17171050590935	-5.03511668613844	-0.90411170855700
C	-3.45501426974083	-1.08267137157945	-1.14148478573360
C	-5.52557497884674	-0.35470981750808	-2.08284195230112
C	-1.50582659356042	1.19746906613521	-4.41814648196500
H	-1.65165802272658	-0.59355525214988	-3.26759046220571
C	-1.35680594349911	3.42034218359864	-5.70365492527082
C	2.17403389469029	-3.70857328204201	-1.02407222974595
O	-2.06082559406868	-0.97269930985125	-1.09428345389658
C	-4.07284845700617	-2.03965070213048	-0.34583228046208
H	-6.09892368633643	0.22898563164350	-2.80415499054122
C	-6.23045430636744	-1.18738609843647	-1.18478165369151
H	-0.49707591403463	0.93670178200569	-4.73497361870410
C	-1.24837800996555	4.74990402426383	-4.92622106757395
C	-2.15172965815722	3.64728959593609	-7.00503740261810
C	0.06239670331444	2.95717602627870	-6.06193498864913
C	2.86918190474921	-2.55363047468170	-0.57067792903531
C	2.56726421731258	-4.92027459443943	-0.47942649284036
P	-1.29151101558214	-0.52278147067902	0.24432267264195
C	-5.50401456315517	-2.05367244421809	-0.30213316243586
C	-3.25778492432892	-3.05310669076683	0.37799571617473
C	-7.65019556494158	-1.19442450373618	-1.14470140781322
H	-0.72180438163734	5.49902194174315	-5.53628073432167
H	-2.23710302952764	5.15653282799692	-4.67060745598688
H	-0.68860774535205	4.60847520602084	-3.99108630361292
H	-2.23736944962612	2.71339137289347	-7.57944923161943
H	-3.16744787042420	4.01600212570212	-6.80340108971296

H	-1.64140114520725	4.39234126553895	-7.63361303113455
H	0.55094582611273	3.72571002087329	-6.67803851194299
H	0.68030802326467	2.80213992385326	-5.16603640652941
H	0.05183192178728	2.02275389287562	-6.64218106450411
O	2.49731118584342	-1.31987210962782	-1.11224292232653
C	3.88662146955960	-2.56625951266679	0.37180092058767
H	2.07899153911703	-5.83688181550698	-0.81651362419474
C	3.57970561774056	-5.01282031930038	0.50458057168301
O	-2.05591073495753	-1.27979022003220	1.43027340799921
N	0.17602119885493	-0.93392265463698	0.01692071540011
N	-1.64889166219217	1.05630623457627	0.63572835061422
C	-6.23169168180886	-2.86502216463818	0.60528802732613
C	-3.41746049989830	-4.45647213444358	0.11868241500548
C	-2.24824312875796	-2.66565304284727	1.24218159696622
H	-8.19366449258147	-0.53299473847761	-1.82205015901798
C	-8.32836240166946	-1.99934138078021	-0.25509831493441
P	1.61881067143452	-0.31320598572334	-0.20127973573692
C	4.48929016751945	-1.28576096730368	0.83326122820277
C	4.25197943748302	-3.82574596040529	0.95280052140935
C	3.91932649377385	-6.26345131448216	1.08660158114481
S	-2.27057627209899	1.66353842374935	2.08737372924805
H	-1.41018967957287	1.81997156090937	-0.04615738141698
C	-7.61060840177262	-2.83477314878428	0.63129874140393
H	-5.68635289660153	-3.50270151268058	1.30006742000879
C	-4.34139488940211	-4.97088397967748	-0.82785929047867
C	-2.55746466016748	-5.38213594082724	0.80025047533842
C	-1.38019378683598	-3.55240998156911	1.93332300696723
H	-9.41882260177356	-1.98775176066127	-0.22678072827001
O	2.28112140925732	-0.46619046721317	1.26377347879752
N	1.75874468471010	1.15793681182011	-0.78409807648816
C	3.66833773646306	-0.28382692893453	1.33378289732322
C	5.89969005581669	-1.03936263533357	0.79559424986200
C	5.20863815860387	-3.94948512725136	1.99437753534696
H	3.40639476461246	-7.15857566645444	0.72816396865994
C	4.86055770104913	-6.34906394536242	2.08897317935775
O	-2.09552936582107	3.09629324064214	1.98062292474396
O	-1.74083408165666	0.91164891708432	3.20428058267290
H	-8.15056291611376	-3.45062727974540	1.35136753829288
H	-4.98260020947079	-4.28292912316404	-1.37512058249357
C	-4.42828525743329	-6.32575059070769	-1.07460962819520
C	-2.68506849543189	-6.77174908546756	0.53477486003551
C	-1.57552673310480	-4.90371504896246	1.69883951411924
C	-0.30769613672161	-3.08453133884018	2.83771582593156
S	2.28715917727594	1.50831431415805	-2.26332478863695
C	4.13833848379420	0.91423418262241	1.93714362216683
C	6.40537611023244	0.17215691843968	1.37644669883313
C	6.81273477423632	-1.92138771077226	0.16296192359256
C	5.50338875507236	-5.17776495379845	2.54995953285423
H	5.71117932430613	-3.05646178053386	2.36118721103325
H	5.10619581615508	-7.31525026464698	2.53183493445180
H	-5.13973952727041	-6.69350847604144	-1.81542621336140
C	-3.59988430817929	-7.23827517803698	-0.38329485309553
H	-2.02910832188955	-7.46367651224157	1.06706932836992
H	-0.94514935408101	-5.62564182473131	2.22161688573288
C	0.96020004041219	-3.68851991849209	2.79068583117927
C	-0.51539578480419	-2.07561032118836	3.78733053885142
O	1.78368719436861	0.60194932133056	-3.29423885970369
O	2.05836841749202	2.94270379607236	-2.47612512729130

C	5.51041327023116	1.10137688209177	1.95804937006634
C	3.21594121992470	1.88956982661028	2.55979875248255
C	7.80325386595137	0.41866439875120	1.34640555764929
C	8.16335046219582	-1.64082295099760	0.13274194849544
H	6.43172798632047	-2.81999707765665	-0.32088708189491
H	6.23732026653194	-5.24303182113036	3.35464625970822
H	-3.68109253212904	-8.30729961855360	-0.58426585443992
C	1.96386559476593	-3.31486505414906	3.67674621632984
H	1.17042105849100	-4.44259214186124	2.03055962441451
C	0.49691366083767	-1.70618130658045	4.67272246081526
H	-1.47849600510020	-1.57234389983369	3.84925857405935
H	5.91728472784780	1.99580206053136	2.43223991779850
C	2.13305359795810	1.47037419387294	3.34748187758020
C	3.43456143330548	3.27106325886292	2.43064236050342
H	8.17801541551612	1.34184802425873	1.79201121798439
C	8.66667635952711	-0.46785559066674	0.73960650184280
H	8.84499132140733	-2.32352525591199	-0.37626672721497
F	-8.24505725020855	1.97698837632581	-0.13703313618264
C	1.75558006201288	-2.32153735179879	4.64901407677206
H	2.93375611048854	-3.80781987067053	3.59803048151902
H	0.28433668168566	-0.92099891342141	5.39753959788256
C	1.32953648823967	2.39226665650668	4.01469451807740
H	1.91312301178556	0.40942447900374	3.45554427486927
C	2.62313806937662	4.18397620681320	3.09802206107657
H	4.22641828943928	3.63647219327118	1.77544789937700
H	9.73670555554711	-0.25731465122800	0.71131997772858
C	-7.04645125081174	2.57458633248150	0.09141681768400
C	2.86645769468975	-1.98020636959075	5.64808239132061
C	1.56242795319744	3.76995699133070	3.92154806522424
H	0.50266296708547	2.01299415145712	4.61325410900019
H	2.82622483284946	5.24857482060696	2.96778392689564
F	-7.30641297666775	3.80798744865497	0.60490523815303
C	-6.17247813650580	1.76638973717993	1.02015728158360
F	-6.45565099627407	2.76889091411856	-1.12072961332622
C	4.15283094401673	-1.58309469771699	4.89780806299709
C	3.15090415573258	-3.23333824119561	6.50340138049011
C	2.47707745353444	-0.82661471479127	6.58304506434036
C	0.71933968443678	4.80185863363147	4.67853382017777
C	-6.74818062006999	0.81150117904194	1.86376294058257
C	-4.80359959480797	2.03100748656637	1.06850712830908
H	4.49640181945889	-2.38654628488457	4.23353345279159
H	3.99917566255483	-0.68171665844183	4.28980105132544
H	4.95739580492531	-1.37357703600820	5.61873544193010
H	2.25136132832241	-3.54491166643531	7.05390822724834
H	3.47633749144530	-4.07758525302745	5.87935902398793
H	3.94699465027019	-3.02125070122656	7.23326569026288
H	3.30721325405360	-0.62141454714428	7.27420224782887
H	2.27305275128182	0.09865275484205	6.02503384020419
H	1.59306762781428	-1.07166703372831	7.18976718501026
C	1.64449886007690	5.65452797561919	5.57018496425698
C	-0.34176501210069	4.14066271165671	5.56996290751205
C	-0.00713256028981	5.71402308094190	3.67094960013383
C	-5.94415593503483	0.11382255351586	2.76307704701102
H	-7.81632598855471	0.61002151574892	1.81400173995485
C	-4.01898386823354	1.30725231221165	1.96795981652118
H	-4.35750130514502	2.79663787015843	0.43045432461340
H	2.39544969188328	6.19456460296417	4.97724354101635
H	1.05446160801367	6.39826424732074	6.12713885057267

H	2.17759097068406	5.02334970704020	6.29587837941286
H	0.11475640669554	3.48835676580459	6.32876782416257
H	-0.91398125603156	4.91820448313119	6.09668406466692
H	-1.05161406291523	3.54376891213607	4.97966506053970
H	-0.57264559171580	6.49463516406346	4.20293430540257
H	0.69999056664246	6.21089721506707	2.99110237544250
H	-0.71501308364553	5.12530699661875	3.07138303406619
C	-4.56882529015832	0.35219927675189	2.81659399037940
C	-6.54289848125531	-0.86524788630601	3.74349199504303
H	-3.92744374805419	-0.18572256129747	3.51338259967707
F	-5.84101900511603	-2.03124975094965	3.78480175062134
F	-7.83160753383333	-1.17114509277790	3.45316555171115
F	-6.52644921484753	-0.35539930155707	5.00813946527865
C	4.87924665887190	2.24531605778829	-1.59017076283677
C	4.07417187938628	1.28961758125621	-2.21181152947627
H	4.44225909741663	3.11675236058419	-1.09999295508721
C	6.26583143632604	2.07910649031632	-1.61963195509238
C	4.63105537076184	0.17678342990916	-2.83554950985857
C	6.84267362925734	0.96399849304422	-2.23050347231543
C	7.13594368878212	3.16678297700941	-1.03945161081587
C	6.01788156008324	0.01240017125573	-2.82897738692484
H	3.97978843144295	-0.54534204853347	-3.32525956210782
H	7.92266671325708	0.83480205327053	-2.23121765738975
F	6.71050799725667	3.56249693513169	0.19318994904947
F	7.12866583739007	4.27665194961566	-1.82921518867469
F	8.43448295867289	2.78172638818596	-0.91114211499202
C	6.59901914315475	-1.19386343320915	-3.51971836946621
F	7.92992653373515	-1.33810833585387	-3.29151735334846
F	5.99114756112761	-2.34711745185544	-3.11559503909894
F	6.43081914654357	-1.12357474697884	-4.87197798455020
H	2.69891859228641	4.33381673018088	-1.38108160592835
H	2.64714296441645	6.88891602166187	-0.93701758713906
H	0.79500212235290	5.57003581449410	-1.93959288997010
H	0.82232393457004	3.69736126101594	-0.19596426587007

228

C-TS1

C	-1.18386769018213	4.16702320656445	-0.62876822530100
C	0.13419675372626	4.55804032691640	-0.04827784033150
C	0.77401154921666	5.68000399511342	-0.89784963291988
C	2.22258440966959	5.95563317931329	-0.50121647995914
O	3.06658073627942	4.81949644586847	-0.63822485920349
H	0.03155191636129	4.89614202048932	0.99079626647727
H	0.19015912701549	6.60973349405494	-0.80758048823640
H	2.27708591639028	6.27108813078339	0.55472053637575
C	-2.35506707299650	5.02525756522401	-0.44218366206336
C	-3.51238190626491	4.85454058890667	-1.23214380538015
C	-2.36706541681180	6.03655027452778	0.54022030861208
C	-4.64126872790115	5.63998376683162	-1.02684823713099
H	-3.52261976280827	4.10477600552471	-2.02210926939438
C	-3.49625575429905	6.82260059151283	0.74368465962758
H	-1.49607314351849	6.18504842220796	1.17569675778703
C	-4.64023251829890	6.62346809740325	-0.03340339671344
H	-5.52906656864647	5.48240432690791	-1.64038336348651
H	-3.48895707225877	7.58750938880391	1.52125441194252
H	-5.52880864691681	7.23440305104255	0.13246698931182
C	-1.24647334453045	2.97487067357233	-1.34816533877219
H	-2.11438020237850	2.75679609989781	-1.97130144206747
H	-0.29109269776585	2.58396185759409	-1.70999500730607

H	-3.55917309784319	-2.33221113439139	-3.85052089781533
C	-3.55568484233540	-3.35838979592696	-4.24134740319671
C	-2.21008496959223	-3.69353160706825	-4.91311242781962
H	-4.37432407238138	-3.44658311539539	-4.97180727423596
H	-3.77065080253230	-4.03982969867679	-3.40799455362407
C	-1.07638888373077	-3.62462395559696	-3.88395780755979
C	-1.99884032576548	-2.72130888336134	-6.08218950623164
C	-2.27313698997343	-5.12989504935628	-5.47432529445819
C	-4.01397366036810	1.84358363723254	-3.44888680324203
C	0.05788004740419	-2.82012097951276	-4.05508527478175
C	-1.12869763931825	-4.41620959659378	-2.72373481916756
H	-1.95943353602716	-1.67632884299451	-5.74197729703248
H	-1.07449376921544	-2.94219419705976	-6.63541924932465
H	-2.83782842143800	-2.81028277998363	-6.78757766870386
H	-2.46757623712483	-5.86186880986512	-4.67788982412200
H	-3.07988883105223	-5.21234649027546	-6.21874163917248
H	-1.32509365486670	-5.40196538160258	-5.96040170822443
C	-3.42097427694550	0.63943363765551	-3.02276676036077
C	-3.34680996592334	2.70962639880501	-4.31062571242992
H	-4.99720073477435	2.12417482673197	-3.06838015633179
C	1.10260422560003	-2.81817012588512	-3.13084370093887
H	0.14726585876060	-2.17660422531604	-4.92975555560941
C	-0.09040280371798	-4.42037367700346	-1.79908854603637
H	-2.00054139411750	-5.04236809911895	-2.52729253528611
C	-4.13645541225258	-0.25518809547124	-2.08413153599681
C	-2.15300149826378	0.33045769858800	-3.54504266004073
C	-2.05087008239736	2.43184942473984	-4.78124487829437
H	-3.83804871684967	3.64234965681770	-4.59545958624207
C	1.05673967454420	-3.63100646380413	-1.98968706192531
H	1.96050579202519	-2.17359412974660	-3.31078057241088
H	-0.18163896650538	-5.02631268891633	-0.89638745273811
C	-3.44927380215854	-1.08883991957780	-1.15230525294383
C	-5.51999152887789	-0.34696315150595	-2.09328541201790
C	-1.48578507392796	1.21041669102979	-4.39548740608199
H	-1.65885917235268	-0.60102984230974	-3.27904015546472
C	-1.29729895688796	3.45908291287868	-5.62895025849017
C	2.16615170593454	-3.70059568186553	-1.01261261052472
O	-2.05995251462506	-0.98080534791389	-1.09806597483721
C	-4.07414681455664	-2.04966991225166	-0.36616259445871
H	-6.08994426922398	0.24587552892240	-2.81011349283339
C	-6.22906068711623	-1.18558363971167	-1.20358934660145
H	-0.48358625870344	0.93799638361610	-4.72241148011113
C	-1.06882533475166	4.72467997305574	-4.77432237738692
C	-2.13243397508893	3.82135362600613	-6.87262664838393
C	0.07151670627780	2.93617998796606	-6.08760886381128
C	2.85676628283421	-2.54271708463699	-0.55953253359268
C	2.56224438542196	-4.91060219569395	-0.46664777284540
P	-1.31482908057258	-0.48534071105044	0.25008592944832
C	-5.50544987573455	-2.05906831970495	-0.32554573415773
C	-3.26071294945740	-3.06287385650112	0.35962279025108
C	-7.64857132939418	-1.18673349619987	-1.16344352106993
H	-0.54622574727112	5.49131900164158	-5.36596221439353
H	-2.01738655435947	5.15478194659639	-4.42168599220005
H	-0.45096450193094	4.48904145207127	-3.89638410480354
H	-2.31377762611985	2.93278565248262	-7.49447438748813
H	-3.10730119399552	4.25086071198597	-6.60223137124181
H	-1.59661098396364	4.56402453246048	-7.48234032017050
H	0.57127960340916	3.70611603992863	-6.69293112408512

H	0.72681607695719	2.70123102069103	-5.23697006982390
H	-0.02719430633575	2.03412793232511	-6.70921776925603
O	2.47861682950098	-1.31005396137669	-1.09626173937289
C	3.87682438737087	-2.55284182393140	0.38027481888675
H	2.07582991231588	-5.82872058111502	-0.80237315714264
C	3.57505873995382	-4.99948279543344	0.51737406568540
O	-2.07685753708104	-1.28910940480341	1.42229373067205
N	0.15589932240418	-0.93820492809159	0.03997209117424
N	-1.65287752190101	1.08005918322145	0.56097680296233
C	-6.23688210172347	-2.87005523655306	0.57934051173837
C	-3.41744797385582	-4.46626283141681	0.10154100748867
C	-2.25488355502957	-2.66808878876470	1.22605011286302
H	-8.18938249304295	-0.51978798828210	-1.83768536607802
C	-8.33025237035597	-1.99104206066725	-0.27585126558851
P	1.58745090435677	-0.31519903283229	-0.17876354381317
C	4.47808632037967	-1.27004560585868	0.83720144964072
C	4.24619575058735	-3.81050894433715	0.96224050135756
C	3.91696160908045	-6.24821692299395	1.10197883002008
S	-2.21447853113889	1.68061531427222	1.97600324780775
H	-1.40451041976112	2.10319670362093	-0.38327032170049
C	-7.61562360872786	-2.83245252597335	0.60735965151416
H	-5.69357224552678	-3.51164506719077	1.27210691378984
C	-4.33978167864253	-4.98445577013584	-0.84467681434013
C	-2.55714567456791	-5.38950671893607	0.78610145683458
C	-1.38695202977977	-3.55519795208168	1.91924239593404
H	-9.42063268228728	-1.97371954632179	-0.24626008160038
O	2.27148808635704	-0.45625098012112	1.27968227770237
N	1.74672183109679	1.16115281326849	-0.76352747769350
C	3.65621640574800	-0.26920558907485	1.33872532730873
C	5.88759416333122	-1.02040406209081	0.79205256864136
C	5.20481170549885	-3.93067398961509	2.00235645263023
H	3.40420596788255	-7.14473209293909	0.74674532465177
C	4.85949324271997	-6.33032932741020	2.10350736419777
O	-2.08316693568160	3.12801440789986	1.86191055432834
O	-1.66399782013337	0.99982610444822	3.13593313797728
H	-8.15775165821748	-3.44750131860628	1.32658724054027
H	-4.98228238463247	-4.29832631386660	-1.39295003963575
C	-4.42424941643010	-6.33997756467581	-1.08951519766137
C	-2.68187191336291	-6.77965960448286	0.52236415066462
C	-1.57828869810107	-4.90730839939336	1.68624662402726
C	-0.31874596517522	-3.08441380743294	2.82756679533482
S	2.26014613782656	1.50334380077582	-2.24062136701059
C	4.12786219438469	0.93216950783816	1.93594847692995
C	6.39391803205863	0.19331956765619	1.36776104803661
C	6.79950757092779	-1.90242429023297	0.15759103198547
C	5.50188976056048	-5.15725127960536	2.56050895423468
H	5.70599635091322	-3.03596390203883	2.36698760959926
H	5.10636445058532	-7.29512568195256	2.54875819519548
H	-5.13489275843507	-6.71025844228259	-1.82995125896097
C	-3.59451950594134	-7.24981858777641	-0.39625979917675
H	-2.02524661106112	-7.46952606281126	1.05668997342785
H	-0.94885170664362	-5.62789441568391	2.21214244008176
C	0.94449435257999	-3.69963247862770	2.79908166222989
C	-0.52372652014278	-2.06050867388562	3.76199226917774
O	1.74234111342280	0.61326013466434	-3.27874785261319
O	2.03508362356108	2.94458677169525	-2.45043511333514
C	5.49946105253229	1.12273297866941	1.95017885069872
C	3.20490559113566	1.90699079233418	2.55931041742876

C	7.79133454720081	0.44148085414078	1.33164697432403
C	8.14966680157055	-1.62016738004873	0.12120967090141
H	6.41761264096728	-2.80322493246036	-0.32154591440385
H	6.23679331212197	-5.21967444074133	3.36456294723935
H	-3.67332842607705	-8.31939357895372	-0.59549069959428
C	1.94485031826729	-3.32429787180425	3.68812684813319
H	1.15462233248767	-4.46420492850098	2.04954260044940
C	0.48465867819030	-1.69049460160450	4.65170417047614
H	-1.47989022200511	-1.54275337762890	3.80644605327763
H	5.90681309784541	2.01907700366475	2.42051334500495
C	2.12424147826329	1.48587446727039	3.34885059068764
C	3.41666903697248	3.28911522041258	2.42543707707385
H	8.16671229014333	1.36593702016760	1.77417570118531
C	8.65378178915177	-0.44520537725041	0.72354933859897
H	8.83026733608780	-2.30348929454416	-0.38845673932576
F	-8.27882158739451	1.94560713927900	-0.03386622510288
C	1.73744408780201	-2.31781278037046	4.64695369686214
H	2.91125340601660	-3.82615544327440	3.62227976899990
H	0.27298419685349	-0.89381962326890	5.36419482952278
C	1.31565195069166	2.40576832723541	4.01185262809827
H	1.90704610858319	0.42474065867259	3.45847137876727
C	2.59950415985440	4.20093312224955	3.08818662437031
H	4.20768275245797	3.65589229724089	1.77011319363718
H	9.72344104515931	-0.23331862809339	0.69094289291141
C	-7.06799923452067	2.53145333266146	0.14388809176664
C	2.84404460230173	-1.97307502443753	5.64979158224815
C	1.54057746927724	3.78414913941019	3.91289866961986
H	0.48768753294644	2.02377422469773	4.60636981879385
H	2.79848363772710	5.26626898916195	2.95471041509307
F	-7.29820719336531	3.77856568308894	0.64741765352005
C	-6.16397636540383	1.73199316053337	1.04712888945247
F	-6.52249667623753	2.71215320056471	-1.09553868958322
C	4.13396824079113	-1.58173875209895	4.90252628998970
C	3.12359727856915	-3.22101476272628	6.51406204611178
C	2.45213485916599	-0.81397514427467	6.57689465049595
C	0.68938491859361	4.81203634170979	4.66712052226323
C	-6.70215742183781	0.76769870874918	1.90422035720872
C	-4.79804105906325	2.01485356107132	1.05913867070338
H	4.47770182551427	-2.38927189096981	4.24327666779225
H	3.98340362674876	-0.68344088415195	4.28907441918462
H	4.93673052004496	-1.36960452404552	5.62486307902480
H	2.22119000418430	-3.52797736618982	7.06247785949133
H	3.45046646979300	-4.06971491269319	5.89691465826988
H	3.91687581892944	-3.00550819059886	7.24611151002022
H	3.27813217617478	-0.60838795144804	7.27302542677104
H	2.25477387876179	0.10952066321336	6.01361875621605
H	1.56275008631257	-1.05364292392624	7.17787985395569
C	1.60765888862845	5.67944707399986	5.55127301499604
C	-0.36346635412286	4.14437298199828	5.56364324851132
C	-0.05087373202341	5.71213583986406	3.65817033346539
C	-5.85993490357669	0.08768251721765	2.78219774700790
H	-7.76677689072643	0.54452900584297	1.88101873269015
C	-3.97252319677116	1.31470156046403	1.93883455191537
H	-4.38021383134130	2.77954051617472	0.40325148485159
H	2.35132330055129	6.22449897699652	4.95356348654301
H	1.01169634797351	6.41927261619043	6.10726914311018
H	2.14976081685369	5.05717919764818	6.27782561877283
H	0.10223155243483	3.50117377991404	6.32445410056189

H	-0.94262619943470	4.91846442564618	6.08809082254204
H	-1.06698978953681	3.53596553378802	4.97765989514202
H	-0.62272797046980	6.48910889126371	4.18901861187704
H	0.65083086271674	6.21470233747833	2.97593707532586
H	-0.75506309166057	5.11029017356888	3.06639850593184
C	-4.48858522559378	0.35543552877083	2.80388201033574
C	-6.41227810129073	-0.91126395569461	3.76863879915156
H	-3.82016563769006	-0.16749443445572	3.48664276614788
F	-5.69471581403436	-2.06879666492333	3.77103287341826
F	-7.70639095748776	-1.23290986377732	3.51713880650827
F	-6.36210428102356	-0.41983235302604	5.04028353277487
C	4.87092933897358	2.22483564890762	-1.60808436201612
C	4.04782655474440	1.27760187666162	-2.21833362112642
H	4.45109477900278	3.09882692430009	-1.10922411108172
C	6.25555323491760	2.04764529889244	-1.65670507039935
C	4.58734297646965	0.15850419111131	-2.84669984713190
C	6.81419443951385	0.92559609558875	-2.27118224887663
C	7.14132258749903	3.13181706888520	-1.09477890914785
C	5.97232345572501	-0.01984629559093	-2.85552237905869
H	3.92366110239962	-0.55880336656263	-3.32653730100283
H	7.89278354860013	0.78590855967036	-2.28507067984772
F	6.73364696543975	3.54159161553312	0.13917331677943
F	7.13152024625028	4.23670594521322	-1.89372539517538
F	8.43812526706150	2.73946829562449	-0.98055115210456
C	6.53290388639444	-1.23653741493100	-3.54535684938420
F	7.86561482100152	-1.39063341429071	-3.33453116792655
F	5.92069611381647	-2.38066051089957	-3.12412192818205
F	6.34654717762399	-1.17418542085557	-4.89580671011481
H	2.69997212560274	4.25362024455545	-1.35730212692870
H	2.59791221844205	6.79552616366951	-1.11789511364372
H	0.74743554848511	5.39078333721361	-1.96119057359424
H	0.80109448181769	3.68613101315913	-0.04273947093581

228
C-I

C	-1.18866641017145	4.36512425456305	-0.63306580846945
C	0.10039283804229	4.71720956711739	-0.00190884876408
C	0.81615124921861	5.73939665186118	-0.93144219886317
C	2.26908811124625	5.96337017584794	-0.50924157457720
O	3.04142247100897	4.77767414078069	-0.51638319175958
H	-0.01299722724443	5.14750060929650	0.99768823109384
H	0.27815052399455	6.70028326026703	-0.92398524680339
H	2.31480348574832	6.37466262034010	0.51316684861482
C	-2.37180179871753	5.17492962655654	-0.44011597877466
C	-3.54235544817973	4.94308718826831	-1.20240253737391
C	-2.40142521841436	6.20048506391591	0.53448694352464
C	-4.69262196783770	5.68919932788509	-0.98396284646931
H	-3.54860075373947	4.16798216286875	-1.96749620569658
C	-3.55169218737419	6.94761590075644	0.74577381196496
H	-1.52420155414109	6.38936067489345	1.15038753767474
C	-4.70238197199758	6.69039542528213	-0.00727517058644
H	-5.59091193881801	5.48408727129510	-1.56658770104332
H	-3.56111792536322	7.72430321704152	1.51109266709154
H	-5.61034527891158	7.26901690933186	0.16963384072976
C	-1.16953961419832	3.22053800468007	-1.50700568752212
H	-1.96789611536310	3.13977667923273	-2.24615560046141
H	-0.17279999058437	3.04298297732090	-1.93460900597353
H	-3.54221632799716	-2.31140628303704	-3.83091975484299
C	-3.54056502050747	-3.33512453448350	-4.22793218480035

C	-2.19630337189583	-3.66707189106606	-4.90361814054189
H	-4.36033963412132	-3.41835403821910	-4.95776580290969
H	-3.75528074716422	-4.02100575921905	-3.39814289686222
C	-1.06067764829955	-3.60683526739007	-3.87608635654542
C	-1.98618373776445	-2.68623008710703	-6.06571155158466
C	-2.26148440044694	-5.09928796374471	-5.47504962248836
C	-4.01779761820080	1.83795939426304	-3.40857180059119
C	0.07710248331299	-2.80735365195806	-4.04688664240931
C	-1.11482134399024	-4.40204119678048	-2.71853860948337
H	-1.94349879187541	-1.64427057357448	-5.71651452009078
H	-1.06386308995919	-2.90452450414345	-6.62339468002072
H	-2.82746476594768	-2.76774135527020	-6.76934914576207
H	-2.45487369939657	-5.83641585066524	-4.68313008476850
H	-3.06974212937041	-5.17613246487817	-6.21852581309340
H	-1.31443732535905	-5.36869455611545	-5.96461062163908
C	-3.39784848021190	0.64995177174387	-2.97305309697980
C	-3.38616502521323	2.69829835339059	-4.30213974296747
H	-5.00266947347596	2.10424369683324	-3.02267975049310
C	1.12310272296100	-2.81358106973817	-3.12425503531936
H	0.16784504929693	-2.16116054661012	-4.91943203789876
C	-0.07541350222342	-4.41383416632655	-1.79527070348832
H	-1.98965186051827	-5.02407454286843	-2.52216504585470
C	-4.10575942017583	-0.26326050749559	-2.04477009713939
C	-2.12440158847320	0.36746071074637	-3.49758358989308
C	-2.09401789755105	2.43612654105174	-4.79385294772260
H	-3.90678962181281	3.60956341561105	-4.60498075715659
C	1.07501769942846	-3.62925831098674	-1.98545631850891
H	1.98469959227662	-2.17354000005027	-3.30313124448359
H	-0.16897104444537	-5.02049305443118	-0.89326614569592
C	-3.42083734061298	-1.13053842052795	-1.14119743762461
C	-5.49084961195963	-0.34253099302780	-2.04585385587903
C	-1.49025454606268	1.24405201460813	-4.37741960630904
H	-1.60304644697008	-0.54435202274517	-3.21743435032424
C	-1.39622820311412	3.44089470526927	-5.71414261827702
C	2.18537835273332	-3.70488455602034	-1.01012060113327
O	-2.03135788538506	-1.05981712905971	-1.09648027459161
C	-4.05531347536047	-2.08755158893909	-0.35755117693841
H	-6.06156294090262	0.26850752373874	-2.74640676041121
C	-6.20413068453642	-1.18921430147114	-1.16778938914443
H	-0.48500942494279	0.98805238394963	-4.70827003814152
C	-1.26003817554878	4.79153214377495	-4.97897469908979
C	-2.24213804357135	3.63641779837988	-6.98845255195257
C	0.00880945818597	2.97258183907880	-6.11882142392456
C	2.87712601878681	-2.54973393780720	-0.55127442795387
C	2.57986885183782	-4.91813817952716	-0.47041870848768
P	-1.30192817929769	-0.51708657436062	0.24920640618906
C	-5.48530584803534	-2.08049108004165	-0.30428930516496
C	-3.24473939624173	-3.10367947037596	0.36748202913429
C	-7.62337068789567	-1.17495963194545	-1.11940230875668
H	-0.79273366958773	5.53615027609629	-5.64062061638776
H	-2.23636864376762	5.18604571889301	-4.66264296542976
H	-0.62650034842598	4.68519561854416	-4.08680726741229
H	-2.35443685030106	2.68663889702885	-7.53075104736060
H	-3.24784552206393	4.01454428464668	-6.75641761571079
H	-1.75529952913951	4.36045224632498	-7.65893550393551
H	0.47352914781450	3.72932155574956	-6.76725926523015
H	0.66130667588887	2.83001223544023	-5.24543262683566
H	-0.02498815736567	2.02784829446180	-6.68050431969918

O	2.49941510152248	-1.31216603207764	-1.07580515057818
C	3.89639437828020	-2.56696327487091	0.38931057796160
H	2.09121997879611	-5.83365006087008	-0.81006879915025
C	3.59233778025678	-5.01357905128673	0.51334987585511
O	-2.06140564803752	-1.33104036601160	1.42730096666209
N	0.17434308915832	-0.99068955154952	0.06566197233505
N	-1.64102422523371	1.03394644632205	0.48140576026637
C	-6.22013201447280	-2.89045817231943	0.59882039656393
C	-3.40278032573214	-4.50611875817521	0.11062189561163
C	-2.23969672102031	-2.70541436952694	1.23451758834844
H	-8.16074760267916	-0.49503765956768	-1.78350221636653
C	-8.30870043757990	-1.97937241652359	-0.23474314012784
P	1.59007336625990	-0.34133449770721	-0.14509319048254
C	4.49500772804177	-1.28666207712752	0.85626381768827
C	4.26458463368277	-3.82774691446400	0.96468652118108
C	3.93245775930056	-6.26557187761484	1.09183122365077
S	-2.16026328433335	1.71185784565312	1.83585903659518
H	-1.29872169147890	2.33728066696676	-0.79525018544480
C	-7.59809085745953	-2.83647503612086	0.63672607795770
H	-5.67928652710238	-3.54418328884414	1.28215349426914
C	-4.32614547980253	-5.02339312219116	-0.83523561843457
C	-2.54362408974566	-5.42991965625424	0.79599976969268
C	-1.37373282782351	-3.59477717624581	1.92845887025815
H	-9.39862075372132	-1.94970106582566	-0.19757714819827
O	2.28748904182740	-0.48485325846749	1.30904929162862
N	1.74862627834041	1.14783279303870	-0.70990319922917
C	3.66978413774724	-0.29267434466141	1.36626874503826
C	5.90370800025251	-1.03382926023624	0.81327392195446
C	5.22230835130417	-3.95414577803881	2.00484721911091
H	3.41862496975520	-7.15964343047780	0.73197396149182
C	4.87421916319515	-6.35385066524014	2.09363840765902
O	-2.03321912286540	3.16219664880329	1.64644655827729
O	-1.61477934734329	1.12894573977122	3.05560171394665
H	-8.14284533934000	-3.45109009491856	1.35440502958865
H	-4.96830550108500	-4.33585969573417	-1.38261830086316
C	-4.41213279797314	-6.37889874083123	-1.07991283239461
C	-2.67013279249289	-6.81994082230796	0.53247863645254
C	-1.56517476152404	-4.94718185386906	1.69661817845148
C	-0.30586322194733	-3.12322111089128	2.83704580256506
S	2.25850232327736	1.50745946225264	-2.17669548693115
C	4.14032408920545	0.90484557369202	1.97360682896470
C	6.40800410005815	0.17567086394102	1.39945127026829
C	6.81708295680657	-1.90934256789032	0.17181389886009
C	5.51764412533176	-5.18385305533184	2.55706000226921
H	5.72352090679005	-3.06167381715398	2.37493686905948
H	5.11939268722608	-7.32116227455375	2.53440241644141
H	-5.12363341520724	-6.74886978316793	-1.81974410029966
C	-3.58316141197115	-7.28936426811763	-0.38636214657135
H	-2.01463248950995	-7.51065469697381	1.06721900335323
H	-0.93709952513422	-5.66813634734018	2.22375408447065
C	0.95370832190384	-3.74634147407268	2.81704736149448
C	-0.50597113024974	-2.09000645925125	3.76289163730548
O	1.72446326556851	0.65621203746863	-3.23838971348621
O	2.06107860111138	2.96184832252797	-2.36010768296411
C	5.51145488284677	1.09849029121725	1.98937572579912
C	3.21360336068964	1.87195354412812	2.60356393475249
C	7.80512769014135	0.42581073774840	1.36594793885898
C	8.16689254268587	-1.62502089600574	0.13810382743715

H	6.43620106811914	-2.80702933020189	-0.31407135902544
H	6.25187764409610	-5.25118881449399	3.36135745319960
H	-3.66304068876204	-8.35894610421179	-0.58533001061339
C	1.95373378817186	-3.37252748803980	3.70707376916589
H	1.16192266554936	-4.51591697872541	2.07210595177365
C	0.50117156043984	-1.72358252004346	4.65566826430538
H	-1.45754586526776	-1.56267745052752	3.79805521401438
H	5.91696171910837	1.99164798088910	2.46749449606240
C	2.13277271953111	1.43949069968550	3.38638824210552
C	3.41534889704617	3.25607920064921	2.47669693929314
H	8.17923017658353	1.34681018370896	1.81676267052201
C	8.66910906154755	-0.45442789428374	0.75056015850261
H	8.84869588498111	-2.30347722241233	-0.37652757166999
F	-8.27808373211444	1.98458136916380	-0.02427858600947
C	1.74905486897023	-2.36068795128140	4.66069138233585
H	2.91739951528382	-3.88050223536448	3.64657064256870
H	0.29237676233342	-0.92168124653599	5.36321998721327
C	1.31087686885669	2.35000810344264	4.04491367720659
H	1.92291732626826	0.37644262769305	3.48982077226678
C	2.58322646034507	4.15937084840296	3.13313421843711
H	4.20859429614527	3.63145039222412	1.82910699197325
H	9.73850489155395	-0.24078661199509	0.72031571252837
C	-7.06658095006138	2.57225887190366	0.13512922663582
C	2.85372458396849	-2.01976136858703	5.66708359841349
C	1.52055631438528	3.73069404080856	3.94696229937830
H	0.48014980496114	1.95907050418964	4.62896678065522
H	2.77302205591213	5.22693347595151	3.00213656119989
F	-7.29431213810289	3.82088306459464	0.64327456126346
C	-6.14315914164612	1.77704213415445	1.01916365040231
F	-6.54478695473120	2.76242700994282	-1.11541882971950
C	4.14739462412160	-1.63370202883477	4.92349754321792
C	3.12608694578983	-3.26803244639949	6.53312398887787
C	2.46328524479660	-0.85913022750805	6.59292474413894
C	0.64453112691535	4.74720776093370	4.68860931916790
C	-6.66224528298643	0.81989235487298	1.89557714229892
C	-4.77642618364131	2.05609585393791	0.99585932994998
H	4.48921771680202	-2.44279646646623	4.26511487004601
H	4.00198181973425	-0.73498009482806	4.30935676181074
H	4.94922495817408	-1.42468107019272	5.64787606723725
H	2.22072719316979	-3.57126039760695	7.07876551740197
H	3.45169845612042	-4.11847034594736	5.91775494750841
H	3.91783841141555	-3.05501166775956	7.26763487849380
H	3.28705035250981	-0.65804064547681	7.29309405017058
H	2.27296847149336	0.06569166949527	6.02947083798890
H	1.56978157038995	-1.09498690228228	7.18932431209482
C	1.53684834664298	5.62985542781435	5.58382214402469
C	-0.41180336820381	4.06496396761785	5.57005237680068
C	-0.09513272711097	5.63497250714259	3.66772990646908
C	-5.79811262703946	0.14860033227660	2.75915703625653
H	-7.72689749745315	0.59592952704760	1.89924792832342
C	-3.92753624924664	1.36830786663389	1.86176845838318
H	-4.37186145274715	2.80980944331957	0.32067031636448
H	2.28258161796168	6.18273124191204	4.99574271404620
H	0.92291370511795	6.36321223269288	6.12889156694086
H	2.07610758848476	5.01684780950867	6.32022508663515
H	0.05279995346507	3.43141283421116	6.33951477663460
H	-1.01106313168688	4.83088843399383	6.08401064174723
H	-1.09520705856276	3.44287912557672	4.97472348708293

H	-0.69489818829083	6.39674380379675	4.19013419322001
H	0.61289415873131	6.15687405315712	3.00616506635033
H	-0.77154702774401	5.01942867253702	3.05709337417268
C	-4.42707592747309	0.41931266303534	2.74866092225071
C	-6.32709797949967	-0.84403225505481	3.76396936919394
H	-3.74372014868613	-0.09451568627262	3.42347933382474
F	-5.60434008268678	-1.99800685843930	3.76478290714790
F	-7.62439477108827	-1.17499476502172	3.53851413830235
F	-6.25836289991305	-0.34152890397956	5.03112218176474
C	4.88357248564580	2.19816463250992	-1.56598533643929
C	4.04544639311463	1.26510323558778	-2.17709026574587
H	4.47621672682933	3.07012508160431	-1.05434491519509
C	6.26614631870398	2.01097519721641	-1.62993863169588
C	4.56978638635372	0.14749398567538	-2.82073029015114
C	6.80911186368422	0.88857891814074	-2.25747074358248
C	7.16508375226766	3.08643925348771	-1.07320856716236
C	5.95287277052379	-0.04425513985914	-2.84136470882127
H	3.89598491256927	-0.55933388830001	-3.30186674904452
H	7.88614859354234	0.73883066899142	-2.28185408763721
F	6.77236429288782	3.49673304194994	0.16542713420436
F	7.15356953204422	4.19474761830200	-1.86944224910070
F	8.46014305950756	2.68668151058723	-0.97416256806997
C	6.49569787841709	-1.26345252542696	-3.54105926204481
F	7.83041715158147	-1.42593019873837	-3.34975510632241
F	5.88281189739920	-2.40403464196453	-3.11218385417416
F	6.29001397728483	-1.19811149630965	-4.88868638536598
H	2.69170051230690	4.18989764809801	-1.23113948445959
H	2.69934899343290	6.72256790873433	-1.19297801917880
H	0.80248933197546	5.36712353616585	-1.96805746083231
H	0.73047762694225	3.82017062356110	0.07760504248180

228

C-TS2

C	-0.95153073487245	4.31938910520483	-0.66747169331128
C	0.36603602397297	4.52473419208399	-0.00575032884305
C	0.97405182270756	5.95230031718352	-0.04329823462844
C	2.42949457638411	5.94472067112743	-0.48449412634635
O	2.46076938188640	5.64659071334896	-1.88084852001359
H	0.22170220284703	4.21006037984426	1.04575348224125
H	0.91663381764551	6.42242662512774	0.94853992159207
H	2.99376990717822	5.19555094082539	0.10154486990506
C	-2.06215717916510	5.22220539444815	-0.45927312008763
C	-3.19085412412044	5.19021099322233	-1.31380930162019
C	-2.08046591210765	6.10961186307361	0.64209429555895
C	-4.29246401752818	5.99799046320576	-1.06515160915018
H	-3.19427993378387	4.53317906064546	-2.18254602813940
C	-3.19048747567375	6.90218181645499	0.89650017938119
H	-1.23944576242638	6.13003049166825	1.33026518165932
C	-4.29954043933261	6.84876613857370	0.04501977474468
H	-5.15655921401788	5.95641849615624	-1.72872743324074
H	-3.20054361590929	7.55701926709464	1.76848660345605
H	-5.17341511333139	7.46957093693608	0.24884661553722
C	-1.09028164823127	3.14369705657084	-1.47493952085561
H	-2.00870818157326	3.04448128082743	-2.05514644728284
H	-0.17461970161890	2.91958155472371	-2.04780170214071
H	-3.38457142250058	-2.35517596849984	-3.82144286613898
C	-3.50216659144429	-3.41866617281647	-4.06760402272193
C	-2.22961807407808	-3.97424919767606	-4.73501721241616
H	-4.36237339966076	-3.51684384909037	-4.74769498285129

H	-3.73568925252489	-3.95730327665268	-3.14057910054016
C	-1.06150021299621	-3.91174661023808	-3.74477191672520
C	-1.95918676706047	-3.15919521158761	-6.00741755967593
C	-2.47026161890741	-5.44426355896174	-5.13632382982316
C	-3.94504397077036	1.83474452971955	-3.38443355740085
C	0.08644040939413	-3.14352696888347	-3.97643604033572
C	-1.10890801316732	-4.64787272096852	-2.54801243366148
H	-1.82088240098779	-2.09098066546259	-5.78492117615323
H	-1.06891754975525	-3.52077117007701	-6.54250618588541
H	-2.81867623957740	-3.24732651538998	-6.68785232910573
H	-2.72720541634451	-6.06283016305066	-4.26516807869502
H	-3.30151151528307	-5.50921653248075	-5.85498304882199
H	-1.57353106956663	-5.87428323735947	-5.60560451335609
C	-3.36959158318696	0.61956888141849	-2.96305449367821
C	-3.28446723723212	2.67927987445964	-4.27196075176718
H	-4.91583639224680	2.13612278835819	-2.99029904153347
C	1.14535329836173	-3.11714496503838	-3.07009907504884
H	0.17307484987036	-2.54284585728932	-4.88109376641123
C	-0.05459990602291	-4.62933344483438	-1.64101772087841
H	-1.99237636661908	-5.24096498185578	-2.30576931219321
C	-4.11271803255658	-0.27417873867402	-2.04241102898431
C	-2.10806013438700	0.29586648470198	-3.49348140187603
C	-2.00698910957848	2.37279533290662	-4.77477377384304
H	-3.77306636693291	3.61141412205941	-4.56525886021366
C	1.10033269273835	-3.86800972516219	-1.88766880362325
H	2.00964842826630	-2.49444672394322	-3.29177421255357
H	-0.14271521654521	-5.18564894767716	-0.70634844407505
C	-3.46164222234903	-1.16318993828672	-1.13523752520647
C	-5.49968528745519	-0.30893998202449	-2.05032710014637
C	-1.44599135575251	1.15582858018344	-4.36965659422906
H	-1.61745399726480	-0.63624941598162	-3.22330397817465
C	-1.27879732657444	3.35649414295459	-5.69463715231498
C	2.21934479086563	-3.87704977690287	-0.91969994066791
O	-2.07131093873750	-1.13484270433918	-1.08783470742113
C	-4.12892557513534	-2.10074508304928	-0.35372556709777
H	-6.04672179647472	0.32127459062329	-2.75322715938019
C	-6.24374207957237	-1.13477295891769	-1.17756796553858
H	-0.45423636090025	0.86304670999978	-4.71077349102220
C	-1.03390998201050	4.67622180260487	-4.93151865836704
C	-2.15104282922691	3.63592682351170	-6.93503929494686
C	0.08059115474467	2.81392798276620	-6.15834716209390
C	2.88646520905128	-2.68038361756920	-0.53632780059228
C	2.64885251717858	-5.04573692927553	-0.31453722069871
P	-1.31851349156972	-0.62193804459194	0.25685486028529
C	-5.55848829473412	-2.04805484469211	-0.30936337562601
C	-3.35374961405944	-3.13899139952339	0.38117003592517
C	-7.66225169238130	-1.07779259989943	-1.13907830096246
H	-0.55110862009027	5.40998987941304	-5.59432475060760
H	-1.97476806530191	5.11770744658348	-4.57107714200017
H	-0.37067098627986	4.51282824128817	-4.07070928455706
H	-2.34324892217590	2.70879626733128	-7.49417457237400
H	-3.12163756157324	4.07482678174593	-6.66358146221280
H	-1.63820907564480	4.34208412698748	-7.60519470963574
H	0.56090547174098	3.55185748449451	-6.81724892515627
H	0.75527797554834	2.62991553854824	-5.31056967711696
H	-0.02907162204700	1.87912408105858	-6.72740219399563
O	2.46129170639095	-1.48685104250875	-1.11745608285250
C	3.92542400629138	-2.61537071817127	0.37902934524551

H	2.17588536778379	-5.99113107359203	-0.58878465917531
C	3.68208320741513	-5.05721539892565	0.65333032213208
O	-2.11830660766963	-1.39754061770082	1.43255008513089
N	0.13687289144688	-1.17017436132060	0.07640566892949
N	-1.57313161036784	0.94476055126817	0.50337368386203
C	-6.32440407693697	-2.83312658764079	0.59004445814106
C	-3.55190095146515	-4.53946161055901	0.13794643660970
C	-2.33787511232313	-2.76600586646779	1.24682829188279
H	-8.17375029444785	-0.38199746191599	-1.80703629214117
C	-8.37817246423907	-1.86077197864019	-0.25946351399708
P	1.52719790433126	-0.49863897424823	-0.23307568567547
C	4.48909801320420	-1.29185748069952	0.76565100229671
C	4.34021332864266	-3.83236115892678	1.01509977861958
C	4.06028378628421	-6.26327817782186	1.30095502979549
S	-2.12962415442314	1.63119643034772	1.84080269609381
H	-1.10446511524949	2.28168096174826	-0.72533910941477
C	-7.70026595732339	-2.73788076387759	0.61787844089955
H	-5.80880884051025	-3.50183225494690	1.27843968866454
C	-4.49429282378479	-5.03997838846389	-0.79803423793624
C	-2.71437732549006	-5.48159829989990	0.82642373783906
C	-1.49427967798224	-3.67332840976933	1.94519727892699
H	-9.46697447902939	-1.79837696985313	-0.23060122022459
O	2.26625192473906	-0.52687839952127	1.21370084785071
N	1.59435977273979	0.94735476379702	-0.90695968125987
C	3.64015986168019	-0.30328881139003	1.24909768318418
C	5.88601610315747	-0.98981800928303	0.67446831622782
C	5.32923268010242	-3.87726833646581	2.03207826638748
H	3.55519218244755	-7.18732163378573	1.01135148104917
C	5.02810821304159	-6.27182104599656	2.28188072259843
O	-1.92750074008769	3.07517846866046	1.68086872942519
O	-1.67100089657032	1.00978642570128	3.07840556034542
H	-8.26843830457420	-3.33417630455851	1.33283645407250
H	-5.12526620280922	-4.34068578628838	-1.34325876346655
C	-4.61458405659459	-6.39422296564154	-1.03554432493133
C	-2.87595278460616	-6.86916169747484	0.57016116220021
C	-1.71996161331916	-5.02097972432075	1.72182249149556
C	-0.40975851580746	-3.21381661742557	2.84086390572204
S	2.07762919333751	1.22774702202537	-2.40748234434215
C	4.08849437556478	0.91776090195580	1.82888716178167
C	6.36154823053596	0.25991027965169	1.19675877292976
C	6.81353955040808	-1.85520365192737	0.04003739905060
C	5.66250879121779	-5.06463966431907	2.65191129523943
H	5.82519131917619	-2.95544371432002	2.33057385467439
H	5.30155131597821	-7.20483352440857	2.77661400513352
H	-5.33932741253862	-6.75004230058905	-1.76938196351446
C	-3.80375107850730	-7.32107513213675	-0.34276086894305
H	-2.23509117382418	-7.57298746715261	1.10554748568463
H	-1.10777977735088	-5.75559531729413	2.24909838507362
C	0.84785546070427	-3.83996248911068	2.80113944465061
C	-0.59040512897489	-2.17380514449796	3.76269980459353
O	1.73723655547322	0.18371880794128	-3.37286867460411
O	1.69297818322198	2.60464013734645	-2.73898202837058
C	5.45192035501033	1.15824380748002	1.80206917942687
C	3.15316284713818	1.85414364449376	2.49195054288567
C	7.74310163104802	0.57081249179426	1.09314187553706
C	8.14718556405401	-1.51567374942427	-0.05863160793033
H	6.45429881070442	-2.78894915459716	-0.39154361476997
H	6.41937599328523	-5.06851347270958	3.43780040758117

H	-3.90947878057347	-8.38929971949240	-0.53688650858579
C	1.86836005108486	-3.45656904125868	3.66444415480313
H	1.03903208005802	-4.61637476649529	2.05853813277499
C	0.43602460889698	-1.79854637947616	4.62904784813497
H	-1.54028655348125	-1.64466271439245	3.81235436434176
H	5.84312488031926	2.06709220576743	2.26137175057154
C	2.07819774269178	1.38760799118942	3.26435689379649
C	3.35193436573472	3.24451260227161	2.43289278098379
H	8.09331295386822	1.52343437600235	1.49619747601683
C	8.62029394301337	-0.29676800891896	0.47803246424211
H	8.83856245254318	-2.18638739893192	-0.57012143295062
F	-8.07316692552213	2.21264639030196	-0.38371484725138
C	1.68476104370676	-2.43330719745658	4.61073772883664
H	2.83147216506969	-3.96405712212285	3.58784564496938
H	0.24210254942288	-0.98991042763956	5.33307745148518
C	1.26337054276595	2.26608381249273	3.97259750354986
H	1.86966911428230	0.32163235557739	3.32746954236123
C	2.52696795915011	4.11779295990130	3.13877578567811
H	4.16425020562776	3.64584667912992	1.82365329366030
H	9.67765967852372	-0.04105835974454	0.39488122381015
C	-6.90439984357469	2.81207979936043	-0.03842582003098
C	2.81341028278729	-2.07235181772868	5.58308947133146
C	1.47175091775427	3.64958323076281	3.94195073811571
H	0.43921732098225	1.84598959570752	4.54517837317208
H	2.72103761713651	5.19043517851695	3.06647452816009
F	-7.23055211945486	3.99847101902225	0.55686534837518
C	-6.06010735064239	1.95505669241945	0.86680795321091
F	-6.25908212138386	3.13203945916732	-1.19862056346527
C	4.07228028571795	-1.65634201956779	4.79768993319222
C	3.14287307878326	-3.31509954364807	6.43679565240833
C	2.42837593203489	-0.92219807925185	6.52412273899824
C	0.60395815898787	4.62443479693399	4.74477488514244
C	-6.67831166670420	1.04741335548398	1.73313681811592
C	-4.67714173041734	2.13072618497808	0.88673524977270
H	4.40472545064159	-2.45440924188008	4.12198543681258
H	3.88748729400430	-0.75677736753137	4.19536117121853
H	4.89555896138170	-1.43581220517346	5.49433659476860
H	2.26250448787765	-3.64170515668099	7.00915765762010
H	3.47110763743667	-4.15593541316024	5.80987438250314
H	3.95127015427630	-3.08370425275306	7.14722116641765
H	3.26840700804221	-0.70751207533233	7.20055995657715
H	2.20455760060802	0.00055420347503	5.96946849627012
H	1.55675329772342	-1.17616816687372	7.14496747481056
C	1.50464298201882	5.48108121260209	5.65641191243082
C	-0.42617264875115	3.89431069626555	5.61893401308394
C	-0.16506614451523	5.53960276331361	3.77244624090129
C	-5.89668468346253	0.31866408005449	2.62727777020087
H	-7.75711133266412	0.90676069070087	1.70558590911168
C	-3.91174647215650	1.38660103572326	1.78502927100122
H	-4.19439745837593	2.84640892325946	0.22166366881944
H	2.23155829799857	6.06789034261941	5.07759597044137
H	0.89271878784414	6.18402942202992	6.24185683817623
H	2.06537964006735	4.84651934298101	6.35764318215234
H	0.06079350950512	3.23166130660091	6.34909760058021
H	-1.01863674920974	4.63219396101652	6.17933606835583
H	-1.11932851273053	3.29349892339084	5.01322263893105
H	-0.77613312915722	6.26544872288404	4.33121262423923
H	0.52566161739620	6.10558523305922	3.12952186314247

H	-0.83344403337570	4.93650828011765	3.14157487270432
C	-4.50850767650160	0.48299393275817	2.65791579686005
C	-6.53500403741522	-0.60881626204032	3.63064467327328
H	-3.88763879486834	-0.07489406940920	3.35772511972881
F	-5.88221432760741	-1.80042919761323	3.70925628551297
F	-7.83593069803686	-0.87221890608016	3.34547608072704
F	-6.50510216284939	-0.06622913204447	4.88367999255405
C	4.47644083758819	2.38770546082088	-1.72684576983508
C	3.87389818028593	1.27790579328124	-2.31523963529258
H	3.84954628876627	3.17612684273246	-1.30977897757764
C	5.86734520062240	2.48554652517866	-1.71644420570657
C	4.64087133952475	0.26302103826933	-2.87793400092593
C	6.65616139087817	1.47530035092649	-2.27324159138244
C	6.49183989525809	3.72213542854955	-1.12914410890439
C	6.03492482497723	0.36809555948982	-2.85167835988449
H	4.14474004948490	-0.58572891417279	-3.34735082547205
H	7.74102501738755	1.55373903321759	-2.26290468828968
F	7.82940739684792	3.78419297272734	-1.32727025541597
F	6.28191782301699	3.82313299280393	0.21803794808605
F	5.94893544503855	4.85797823123307	-1.67595675154604
C	6.85153295146284	-0.69383398303685	-3.54413746207600
F	8.16987928678810	-0.63314079866598	-3.22230890712122
F	6.41426348524685	-1.94789773866826	-3.24707681021236
F	6.76966078461255	-0.56564896015060	-4.90152999579094
H	3.37170612752021	5.41574669352694	-2.13064283247754
H	2.87565488625239	6.93727899888703	-0.28217140010901
H	0.41513357182311	6.59910598099362	-0.73578383965465
H	1.07000064308890	3.80139403995027	-0.43703582588257

228
C-P

C	-0.48816940433592	4.54800353640017	-0.82300777767767
C	0.30651041630125	4.24169038693045	0.44125990015264
C	1.40799898155472	5.31031539130750	0.44927081033106
C	1.73340175607608	5.53516549800805	-1.02885137181888
O	0.67029514668492	4.82832002271685	-1.79702617694667
H	-0.32506010114260	4.26152854474042	1.33393241782899
H	2.28419580793434	4.97658146961408	1.01573569471659
H	2.67545469154575	5.08471154432986	-1.36130067036727
C	-1.30519074742387	5.82604469982548	-0.74554297315779
C	-2.17068971389700	6.00840539852897	0.34250353078176
C	-1.27592763569869	6.78929554688653	-1.76132617331001
C	-2.97585678173722	7.14503476391100	0.41754777117591
H	-2.23924927280421	5.24317621137458	1.11684790426637
C	-2.07821483600379	7.92932981593626	-1.67883571091416
H	-0.62929116086169	6.64566927459389	-2.62714888631980
C	-2.92799621887856	8.11318885532977	-0.58769107295778
H	-3.64708610588346	7.27061904516720	1.26849663254010
H	-2.04077054939997	8.67301061150216	-2.47652463674233
H	-3.55602193248136	9.00298975429163	-0.52426978754425
C	-1.28832923412650	3.41084073970780	-1.41083607286913
H	-1.71494417084819	3.68618328192346	-2.38099235520767
H	-0.69181377372235	2.49613375667124	-1.50092244884271
H	-3.58669631530235	-2.39471739162606	-3.89521037358457
C	-3.56246847279183	-3.41709986502240	-4.29481386131476
C	-2.20798770815969	-3.72342486776710	-4.96298241689445
H	-4.37540064443440	-3.51442246395921	-5.03034069129742
H	-3.76995909384412	-4.10917018025495	-3.46836929552257
C	-1.07939916706600	-3.63538438106023	-3.92996706919940

C	-2.01205277952169	-2.74299572738381	-6.12793584018165
C	-2.24013429243929	-5.15908756806027	-5.52851160287059
C	-4.10067812130279	1.79666270954176	-3.24601956431403
C	0.04109830832072	-2.81096738064731	-4.09605850745637
C	-1.12416148415463	-4.42636165168663	-2.76920086413031
H	-1.99502859070120	-1.69895337202603	-5.78310434509622
H	-1.08195144011070	-2.94382144026769	-6.67945549900354
H	-2.84714495824857	-2.84575875390696	-6.83600404575269
H	-2.42168941758058	-5.89693391657470	-4.73446191845526
H	-3.04358896120946	-5.25561383547566	-6.27476919739798
H	-1.28572670372867	-5.41067902539628	-6.01339764532664
C	-3.49657869189638	0.56909692552974	-2.91719225294292
C	-3.51242155914295	2.66688511835069	-4.15867825616354
H	-5.03872150093939	2.08052558535815	-2.76584646237502
C	1.07860129315497	-2.78791134518974	-3.16417085573666
H	0.12369398135780	-2.16704656488994	-4.97108244871219
C	-0.09431286781569	-4.40796974039453	-1.83587198866277
H	-1.98565870868185	-5.06755569546688	-2.57710646204390
C	-4.18361591266900	-0.38838260441686	-2.01928667942412
C	-2.26764852590188	0.27306937317531	-3.52933163445849
C	-2.28743732304697	2.36842449221133	-4.78104264638633
H	-4.01841859343291	3.60921593043835	-4.37844429011515
C	1.03822927179859	-3.59660203145738	-2.02031060433489
H	1.92681436132595	-2.12953517181793	-3.34010268896117
H	-0.18260006565596	-5.01054787528229	-0.93070007795371
C	-3.47423882384390	-1.26400029377571	-1.14688951861227
C	-5.56412145025998	-0.51141298068371	-2.03248413971507
C	-1.68065138694080	1.15488985244886	-4.43585397638998
H	-1.75475448112262	-0.66058369915298	-3.30829135163465
C	-1.66989560249440	3.34576044334922	-5.78735796077492
C	2.14123720642264	-3.64307033588786	-1.03549498401426
O	-2.08812367449888	-1.13707151725085	-1.09440121931672
C	-4.07454580642245	-2.26569152844736	-0.39302579310743
H	-6.14921900345560	0.11253330493067	-2.70963835240251
C	-6.25023082592532	-1.41924256322662	-1.19311456912058
H	-0.71872409131579	0.87695944660190	-4.86408340130031
C	-1.40311679832768	4.70218269147550	-5.10301443756259
C	-2.65574229524464	3.55146572772318	-6.95552964143387
C	-0.33991631963949	2.82812287516229	-6.35355786857426
C	2.81053217769755	-2.47476586473102	-0.57826230475954
C	2.55686483678726	-4.84489164506966	-0.48645097927333
P	-1.41899151575879	-0.55974065284228	0.26699085835421
C	-5.50544830563642	-2.31298120135482	-0.35328285270072
C	-3.23003539647778	-3.26102050051405	0.32343368575531
C	-7.66910102367513	-1.46163605836099	-1.15964399967847
H	-1.02715243623374	5.42892747179832	-5.83936170361404
H	-2.31263546407668	5.11993651405485	-4.64884170749975
H	-0.64498051144229	4.59662651832544	-4.31425358432525
H	-2.86655830733312	2.59725430788873	-7.45940536711799
H	-3.61168450122287	3.96951806043329	-6.61065458838227
H	-2.22912215470450	4.24576993781944	-7.69536532929878
H	0.06803717841012	3.56374578608625	-7.06200263647376
H	0.40738817766074	2.67155259011557	-5.56211762412836
H	-0.47115003137696	1.88018950365577	-6.89522417181185
O	2.41549856241336	-1.24378411367608	-1.11261480853092
C	3.82594379423836	-2.46515126707990	0.36488566377892
H	2.08756547611496	-5.77124416140655	-0.82362106055464
C	3.56565433946681	-4.91588595043802	0.50276507237065

O	-2.11540336239696	-1.46471385270142	1.41814970422170
N	0.09132126054519	-0.95839369029810	0.07748441410617
N	-1.82944419842712	0.95212748937275	0.53342865864235
C	-6.21818728313158	-3.17627269503288	0.51768424789558
C	-3.33987941361988	-4.66728948808433	0.06155799423714
C	-2.24523870101752	-2.83612251801568	1.20217032771359
H	-8.22551834145506	-0.77888700645508	-1.80466665012859
C	-8.33207832379909	-2.32092195500138	-0.31017097847267
P	1.47396396589315	-0.29777386647652	-0.19355770705877
C	4.41487888413876	-1.17741044933382	0.82385444823938
C	4.21390382294983	-3.71619127340191	0.95236457607546
C	3.92504997722737	-6.15965113818990	1.08734333031419
S	-2.38328291694833	1.55409409985042	1.90823009674401
H	-2.10652836021693	3.18942970878077	-0.71718373167549
C	-7.59767349779702	-3.17644916932057	0.54235280657956
H	-5.66051013869884	-3.82880643238240	1.18830922367801
C	-4.23640238920836	-5.20992237348884	-0.89600529312586
C	-2.46268833997099	-5.56777470086423	0.75585157103588
C	-1.36181748969442	-3.70229240997489	1.90501490670922
H	-9.42265476658277	-2.33283550588997	-0.28398688231733
O	2.21436439226031	-0.34191582579251	1.24498521167015
N	1.58099828928940	1.18044684615179	-0.84519377088023
C	3.59396984812291	-0.17470985922648	1.32948259897323
C	5.82783306731315	-0.94646591360117	0.80605241188602
C	5.16699976337731	-3.82239324476526	1.99881189155605
H	3.42804574484543	-7.06353468086757	0.72864008646977
C	4.86343398539430	-6.22788064751508	2.09363452096191
O	-2.41236053986388	3.01268499743006	1.76427201078043
O	-1.75023221325337	1.00332292292733	3.10621864729874
H	-8.12463949781101	-3.83300806534305	1.23588084167282
H	-4.89149394222971	-4.53931176314513	-1.44902477767762
C	-4.28171165418562	-6.56695958116036	-1.14296432263725
C	-2.54741671360014	-6.96057493765100	0.48967332142880
C	-1.50912133527287	-5.05931180066553	1.66949097322867
C	-0.32370207291083	-3.19133944933888	2.82732738700697
S	2.12490965571034	1.44646304015942	-2.30352590678895
C	4.07578895740956	0.98396619190055	2.00902515425713
C	6.34395671593640	0.22533043794304	1.45167793112226
C	6.73540794031497	-1.81229911441320	0.14366938191468
C	5.48220833174576	-5.04473767977491	2.55634204184263
H	5.64849614301506	-2.92117211806176	2.37281244303555
H	5.12376410589066	-7.18901548451735	2.53904769601955
H	-4.97423106103950	-6.95573877382380	-1.89114634172707
C	-3.43618976798263	-7.45470598399965	-0.43997010239102
H	-1.87965401078404	-7.63338443193164	1.03216003010082
H	-0.86892542166395	-5.76368334248023	2.20453871331062
C	0.95298679122187	-3.77859115595807	2.84275769207572
C	-0.56560157056421	-2.14168137640496	3.72488766605526
O	1.65267051630744	0.56342545313880	-3.35907119113280
O	1.94012071280006	2.90797825109967	-2.58988219788524
C	5.45391217725221	1.12968533037249	2.07438055622481
C	3.17653758176716	1.95996970241117	2.67029820334754
C	7.74596187759243	0.45236606099956	1.45091166349481
C	8.08979314852339	-1.54914233319167	0.13914611626915
H	6.34673245555478	-2.68436990169913	-0.38095500758701
H	6.21159316447789	-5.09522146162802	3.36622114470238
H	-3.48442228529046	-8.52606281021035	-0.63971368040398
C	1.93172709935308	-3.34797176293464	3.73176200874840

H	1.19197414140279	-4.56525635468511	2.12553118744038
C	0.42119636327436	-1.71387002455828	4.61201433962825
H	-1.53155321801670	-1.64199563720801	3.73756174361438
H	5.87676788173462	1.97132067117189	2.62420788349229
C	1.94021793922589	1.59722538564182	3.23228017233209
C	3.57619375874257	3.30255898739894	2.82017410659134
H	8.12940510655767	1.34360418703688	1.95143229495188
C	8.60333027441142	-0.41423798938161	0.80795621060838
H	8.76768756615527	-2.21734255815132	-0.39354400636989
F	-8.58429509364364	1.40840744626219	0.30032056512260
C	1.68884055667775	-2.30869184655202	4.64619189463796
H	2.90968559712378	-3.83053937091514	3.69871767527407
H	0.17977475369443	-0.89504514462016	5.28879777186911
C	1.14365998811990	2.52901843397905	3.89624101644774
H	1.58061287192989	0.57374091006493	3.16556032485944
C	2.78656887872349	4.21993129138662	3.50710995760430
H	4.51795519898431	3.63763021177094	2.38132559144248
H	9.67691230484662	-0.22047685579268	0.80361387659897
C	-7.38989546033968	2.04878518194220	0.38043348865163
C	2.77485048753509	-1.89018622839386	5.64323012083923
C	1.54344767684450	3.86057096468219	4.05694069515272
H	0.18171369500477	2.18821857654327	4.27504870691141
H	3.14685690510719	5.24642882596539	3.60350196085744
F	-7.63646686010339	3.28544538977164	0.90318306874387
C	-6.37951491873467	1.29690382863300	1.20726644887913
F	-6.95607159303418	2.25342693892123	-0.89848223544953
C	4.05716262942704	-1.49047999021188	4.88835423022744
C	3.08474834924508	-3.08940936476247	6.56361465962218
C	2.34084285264801	-0.70303175591911	6.51450193081528
C	0.69872614757281	4.88665091505604	4.81896545101852
C	-6.78929574789706	0.27318738975096	2.06518798722354
C	-5.04027389444943	1.68501747263413	1.14866123399055
H	4.42884594266950	-2.31224236109448	4.26254415676118
H	3.87939625933532	-0.62292743268443	4.23927429892675
H	4.84939745787294	-1.22385206967656	5.60447767871310
H	2.18739346077518	-3.39865809035588	7.11882742701830
H	3.44071400528548	-3.95473048918666	5.98697192033595
H	3.86562230252629	-2.81889482401363	7.29090434379693
H	3.15482916662317	-0.44002116695035	7.20569059295511
H	2.11789295514163	0.18644180453972	5.90742619485978
H	1.45453086332832	-0.94253449381537	7.11983768166027
C	1.47410854685428	5.31753865251748	6.08161870240014
C	-0.66043781197462	4.31150310166295	5.24349661694580
C	0.43985850336059	6.12627812312309	3.93966519987227
C	-5.84444436530181	-0.35373774415319	2.87636130826781
H	-7.83162651283698	-0.03678325550503	2.09339103276940
C	-4.11034706037620	1.04627379767359	1.96812109168391
H	-4.71858719830333	2.48586441142554	0.48277846072978
H	2.43939389274155	5.77682379894709	5.82484763893062
H	0.88884133030795	6.05205584480209	6.65546263050185
H	1.67312828843528	4.45229625641775	6.73013638933760
H	-0.54611297516008	3.46770677869812	5.93883696764856
H	-1.24129788950526	5.09005778838086	5.75913842202576
H	-1.24638311162182	3.96518316749364	4.37975001406114
H	-0.12148591720898	6.88063336197389	4.51092002468111
H	1.37516636109362	6.59217210660692	3.59914246396765
H	-0.15636959917678	5.86290757715831	3.05472557202942
C	-4.50234366108499	0.03336985349314	2.83774769057572

C	-6.25050370815553	-1.43306825383683	3.84674667635516
H	-3.75937108539462	-0.43838426792738	3.47918797353768
F	-5.45143827768940	-2.53291600867322	3.74244383306121
F	-7.53207299055219	-1.84492044234813	3.66717616029416
F	-6.14742932394182	-1.00205847439480	5.13773219051734
C	4.65763098427953	2.25943639176578	-1.55840525333712
C	3.91462750058343	1.29842931520683	-2.24165418223265
H	4.15620990949415	3.08274464313218	-1.05132562713602
C	6.04842393922696	2.15922895369636	-1.54154070203321
C	4.53680183612555	0.23108416798873	-2.88034232645176
C	6.69195746878215	1.08918019574504	-2.16766813095291
C	6.84578210551319	3.27415060453412	-0.91411012475126
C	5.92930748833229	0.12580619724264	-2.82643873086427
H	3.93434032230675	-0.50264538883179	-3.41412205470152
H	7.77586564363721	1.00278736261651	-2.13203781214397
F	6.30661864291656	3.68789549851551	0.26952540497134
F	6.86879537216932	4.37218641319021	-1.72730980386833
F	8.13426884864167	2.93076131923560	-0.67582027281772
C	6.58728509154715	-1.03257463328940	-3.53468492400320
F	7.91758511341447	-1.11211003258433	-3.27892636022729
F	6.02994284830390	-2.22214711154741	-3.17388852746862
F	6.44315433686048	-0.92890523147368	-4.88663353090472
H	1.10880031789957	3.88007990997137	-2.10523556601314
H	1.68832959667662	6.58329511982579	-1.34271388336709
H	1.03986578049383	6.24036064439090	0.90076326449706
H	0.72858908033240	3.22987808314206	0.36041551600043

228

TS1-1

C	0.954186	-4.171081	-0.174685
C	-0.229143	-4.264120	0.735844
C	-0.957058	-5.616705	0.718866
C	-1.756069	-5.814272	-0.565051
O	-0.927735	-5.660601	-1.720360
H	0.149875	-4.073884	1.754547
H	-1.643318	-5.661073	1.579062
H	-2.588634	-5.091851	-0.599696
C	2.023372	-5.174769	-0.082946
C	2.750157	-5.523315	-1.235735
C	2.352001	-5.800537	1.134090
C	3.779188	-6.458369	-1.174882
H	2.476709	-5.073270	-2.189029
C	3.394119	-6.720903	1.197482
H	1.823048	-5.521464	2.043884
C	4.109663	-7.055552	0.044045
H	4.322098	-6.727123	-2.082693
H	3.652953	-7.178678	2.153309
H	4.921011	-7.783323	0.095715
C	1.117247	-3.063159	-0.994723
H	1.958299	-3.035399	-1.689802
H	0.215593	-2.523445	-1.293692
H	2.784268	5.304498	-5.192393
C	2.612879	4.488197	-5.908068
C	2.519183	3.123694	-5.192966
H	3.446099	4.483804	-6.627371
H	1.684815	4.707380	-6.455551
C	1.350768	3.174119	-4.202585
C	3.838184	2.858455	-4.441033
C	2.340034	2.028414	-6.253692

C	2.408096	-0.730613	-3.400324
C	1.369900	4.092159	-3.138952
C	0.218357	2.357187	-4.316767
H	4.030929	3.626027	-3.679853
H	3.819403	1.881441	-3.940489
H	4.682151	2.862265	-5.147433
H	1.435404	2.189469	-6.857815
H	3.201892	2.036792	-6.936621
H	2.283807	1.027517	-5.801879
C	3.623415	-0.959372	-2.725251
C	1.853747	-1.702168	-4.224771
H	1.881250	0.213905	-3.282667
C	0.305472	4.200629	-2.251677
H	2.236967	4.736666	-2.987575
C	-0.854302	2.460983	-3.430915
H	0.151872	1.618866	-5.115322
C	4.266871	0.067925	-1.874758
C	4.250376	-2.198729	-2.929407
C	2.481685	-2.940646	-4.440137
H	0.892577	-1.487053	-4.694253
C	-0.839181	3.395879	-2.385758
H	0.372992	4.904887	-1.421367
H	-1.707827	1.800307	-3.565591
C	3.519227	0.998163	-1.097136
C	5.646948	0.195795	-1.834703
C	3.697203	-3.159393	-3.777351
H	5.176531	-2.428729	-2.400669
C	1.820982	-3.982494	-5.345845
C	-1.976391	3.583840	-1.457215
O	2.127872	0.871620	-1.110429
C	4.083851	2.051291	-0.388107
H	6.260965	-0.468787	-2.444071
C	6.294377	1.159606	-1.028804
H	4.225657	-4.104346	-3.901198
C	1.605767	-3.370258	-6.745530
C	0.456498	-4.379495	-4.744731
C	2.675542	-5.248307	-5.498523
C	-2.720593	2.495154	-0.923814
C	-2.355200	4.851295	-1.044042
P	1.349177	0.460578	0.243678
C	5.511786	2.104428	-0.285448
C	3.216001	3.100875	0.210787
C	7.710646	1.211490	-0.937544
H	2.563523	-3.068648	-7.194369
H	1.129711	-4.107322	-7.409311
H	0.955838	-2.485804	-6.704577
H	-0.198487	-3.509325	-4.603171
H	-0.057267	-5.088178	-5.411460
H	0.581065	-4.870388	-3.768961
H	2.826967	-5.759763	-4.536250
H	2.164541	-5.956001	-6.166593
H	3.661270	-5.027298	-5.934241
O	-2.374500	1.207099	-1.337670
C	-3.773146	2.625537	-0.029418
H	-1.831972	5.719475	-1.449567
C	-3.396884	5.063362	-0.110935
O	2.024695	1.392234	1.371541
N	-0.129392	0.816102	-0.054768

N	1.761509	-1.052754	0.707041
C	6.183800	3.029382	0.554049
C	3.359364	4.483378	-0.146248
C	2.185716	2.753968	1.069821
H	8.296105	0.490567	-1.511261
C	8.333755	2.131198	-0.122087
P	-1.573727	0.247791	-0.306532
C	-4.456382	1.414074	0.499647
C	-4.117895	3.942354	0.422392
C	-3.721427	6.372576	0.335420
S	2.299072	-1.483176	2.199772
H	1.470549	-2.119631	-0.081854
C	7.560861	3.039264	0.637119
H	5.597006	3.725173	1.152208
C	4.303423	4.944406	-1.100602
C	2.467726	5.440975	0.444229
C	1.282904	3.674927	1.667810
H	9.422177	2.152731	-0.051568
O	-2.319964	0.525870	1.102713
N	-1.786389	-1.258658	-0.781752
C	-3.712515	0.410129	1.107705
C	-5.875625	1.242118	0.410416
C	-5.103516	4.189268	1.413924
H	-3.171110	7.215794	-0.087599
C	-4.693949	6.577744	1.289298
O	2.291085	-2.939355	2.204185
O	1.624925	-0.760164	3.266427
H	8.055816	3.745714	1.304559
H	4.967742	4.227991	-1.580132
C	4.381918	6.280162	-1.437599
C	2.586850	6.810945	0.087377
C	1.465860	5.010047	1.344928
C	0.191591	3.252907	2.572820
S	-2.234912	-1.708122	-2.255402
C	-4.278027	-0.717304	1.767180
C	-6.476213	0.113994	1.062045
C	-6.708345	2.116946	-0.333436
C	-5.383052	5.472293	1.837914
H	-5.639930	3.348410	1.849938
H	-4.928106	7.587963	1.627830
H	5.110462	6.606390	-2.181355
C	3.523090	7.226379	-0.833785
H	1.907288	7.528683	0.551775
H	0.812291	5.757780	1.798337
C	0.367912	2.278172	3.564573
C	-1.069899	3.864802	2.477884
O	-1.614100	-0.934101	-3.328730
O	-2.110874	-3.168027	-2.313054
C	-5.661105	-0.813804	1.749945
C	-3.460660	-1.724044	2.482507
C	-7.884107	-0.056886	0.989600
C	-8.070585	1.909448	-0.403390
H	-6.256122	2.950379	-0.869229
H	-6.140335	5.632917	2.606810
H	3.598210	8.280415	-1.104474
C	-0.668597	1.945591	4.436391
H	1.321847	1.765829	3.667629
C	-2.096580	3.531013	3.353367

H	-1.257083	4.593241	1.687617
H	-6.144266	-1.636213	2.279165
C	-2.300385	-1.385350	3.204748
C	-3.876967	-3.063685	2.524587
H	-8.330510	-0.916808	1.492524
C	-8.667877	0.821632	0.273325
H	-8.688866	2.583822	-0.997294
F	5.439173	2.655943	3.789567
C	-1.921477	2.567774	4.361308
H	-0.477906	1.183516	5.191307
H	-3.058791	4.030795	3.235408
C	-1.627769	-2.336886	3.963557
H	-1.924255	-0.364306	3.192732
C	-3.204620	-4.008199	3.300983
H	-4.744911	-3.375670	1.940648
H	-9.746597	0.670269	0.213746
C	6.207477	1.545116	3.967616
C	-3.060436	2.265664	5.340961
C	-2.075553	-3.665273	4.057124
H	-0.732527	-2.021740	4.502584
H	-3.583589	-5.030021	3.315269
F	6.081451	1.191897	5.279190
C	5.779671	0.418189	3.060588
F	7.500758	1.908104	3.775487
C	-2.709073	1.130283	6.312734
C	-4.332601	1.864546	4.569139
C	-3.349813	3.542492	6.158401
C	-1.360582	-4.650870	4.987830
C	6.718032	-0.308228	2.329124
C	4.424357	0.081270	3.012629
H	-3.559087	0.949931	6.986614
H	-2.499862	0.190373	5.781560
H	-1.838418	1.380012	6.936459
H	-4.172042	0.948333	3.985775
H	-5.157020	1.679305	5.274216
H	-4.652380	2.655107	3.877475
H	-3.646316	4.375475	5.505486
H	-4.167524	3.359721	6.872274
H	-2.460308	3.855405	6.724160
C	0.109959	-4.817206	4.557255
C	-2.022937	-6.035319	4.990412
C	-1.405850	-4.088448	6.424819
C	6.293080	-1.382483	1.542528
H	7.770950	-0.036390	2.363832
C	4.022729	-0.988570	2.220043
H	3.683494	0.632972	3.589646
H	0.632415	-3.854862	4.475936
H	0.649571	-5.439536	5.287208
H	0.168997	-5.318494	3.580705
H	-1.479765	-6.701120	5.676460
H	-3.068270	-5.987477	5.328674
H	-2.002526	-6.499597	3.993285
H	-0.914977	-4.786129	7.120100
H	-0.890592	-3.120923	6.495471
H	-2.444596	-3.944042	6.755317
C	4.943786	-1.730078	1.480029
C	7.300056	-2.234053	0.810562
H	4.609408	-2.576288	0.878848

F	8.496145	-1.610573	0.657267
F	6.863795	-2.584480	-0.433388
F	7.539399	-3.397697	1.479153
C	-4.880812	-2.274145	-1.706850
C	-4.003911	-1.393929	-2.339036
H	-4.489316	-3.135812	-1.166555
C	-6.254803	-2.046926	-1.796886
C	-4.479656	-0.297987	-3.052105
C	-6.750824	-0.944451	-2.496400
C	-7.196387	-3.052156	-1.184589
C	-5.856900	-0.073656	-3.118635
H	-3.775490	0.364013	-3.553531
H	-7.822093	-0.763051	-2.548533
F	-6.817714	-3.402992	0.080560
F	-7.226101	-4.206886	-1.909496
F	-8.472914	-2.597529	-1.109384
C	-6.351700	1.106617	-3.915178
F	-7.682114	1.325978	-3.754872
F	-5.705093	2.255702	-3.566571
F	-6.133464	0.930006	-5.250128
H	-1.169615	-4.795344	-2.111482
H	-2.187532	-6.829526	-0.582516
H	-0.233482	-6.438018	0.832519
H	-0.928704	-3.445667	0.511028

228

TS1-2

C	-1.93191756467025	-3.66461720900963	-2.72970872642533
C	-1.55937882706122	-3.73633493975201	-4.18315788481961
C	-1.18688565405374	-5.11249376484887	-4.76447368935081
C	-0.04239941282019	-5.75753576273878	-4.00223229348784
O	-0.47259106621706	-6.06827741097993	-2.67703453357460
H	-2.41815230480997	-3.33130364806865	-4.74385278560456
H	-0.87985898012934	-4.95789516204988	-5.80887527363320
H	0.82261988776679	-5.07208616087625	-3.98074410812893
C	-3.24155496525666	-4.15562324919518	-2.26842323087614
C	-4.06138767627481	-3.30594383605323	-1.50307433294773
C	-3.69049420086677	-5.46118051462159	-2.53842194656149
C	-5.30254829589425	-3.73994585677955	-1.04548805040958
H	-3.73443052593925	-2.28940298511638	-1.29608314519639
C	-4.92019324368077	-5.89963961464379	-2.05155359122841
H	-3.03455703808634	-6.15778929900894	-3.05378142358581
C	-5.73666561947535	-5.03966596613992	-1.31387858736856
H	-5.93278134448405	-3.05646536385025	-0.47400268270232
H	-5.23814199947743	-6.92539347969601	-2.24411913925416
H	-6.70434889904949	-5.38327779295388	-0.94491033351324
C	-1.12154953422414	-2.94376124134456	-1.86553784862339
H	-1.35171169867315	-2.94697693634415	-0.79803600952000
H	-0.05514404281522	-2.90509777090495	-2.12803508985947
H	-0.32500779304170	2.21671567008305	4.79770292722332
C	0.20560807421875	2.09022039151023	3.84193899136632
C	1.57363657409311	1.42336663542583	4.08492572030041
H	0.31229203932717	3.08269853349614	3.38356101955194
H	-0.42352129138608	1.47894184871661	3.18181446701758
C	2.33834131203005	1.28561420968752	2.76417530460630
C	1.33479126517787	0.06755274663598	4.76429281169456
C	2.40535744169150	2.31175102472512	5.03452267858059
C	-0.51942929677930	-1.25204364862769	1.53639048214266
C	2.87667910026479	0.07136532482243	2.31788220665750

C	2.55988224542823	2.41494636579034	1.95887232889628
H	2.27962854837425	-0.43168730341618	5.02361029735879
H	0.77365238906538	0.21980182373240	5.69764971039156
H	0.74689731713471	-0.60899141093719	4.12707487644183
H	1.88299910612910	2.43438807640338	5.99569060666653
H	3.38911554516910	1.86129690601857	5.23065963405069
H	2.56987383782519	3.31001524143604	4.60473589145840
C	-1.77101152203231	-0.65426425351804	1.31523097227014
C	-0.40661154768837	-2.61872323089274	1.78867652513184
H	0.38785501115262	-0.65190067377341	1.50424854344687
C	3.61887364096163	-0.01397197928774	1.13994463863039
H	2.72665889482611	-0.84058741128449	2.89491297021577
C	3.29819892874059	2.33873452195001	0.78398112653786
H	2.14484859382528	3.38168280488494	2.24596710998159
C	-1.92421669026309	0.78954331125384	1.03130166937453
C	-2.90468793444035	-1.48395726260050	1.37843396286569
C	-1.53225240046300	-3.45213062006722	1.83784966566809
H	0.59225164368237	-3.03403878761630	1.91288311601603
C	3.85940041561422	1.12362648950584	0.35551278139537
H	4.01188582796283	-0.98280461789916	0.83994326584800
H	3.42469218412696	3.23444036883366	0.17432876265469
C	-0.99878387949167	1.49890800611871	0.21670740241224
C	-2.99534783840269	1.51536952947750	1.52464967839400
C	-2.78475590644397	-2.84524110674955	1.63939495978869
H	-3.88985046682329	-1.05863450258082	1.17743626145406
C	-1.43719516426058	-4.96202302664132	2.07463928008830
C	4.71816075684439	1.09892186585191	-0.84903629226525
O	0.06141003990453	0.76735164099261	-0.33406797722990
C	-1.07440139848782	2.85800773743812	-0.05370241880468
H	-3.71616256455839	1.02166108238591	2.17898826770797
C	-3.20935779785056	2.87098426678106	1.18394624997221
H	-3.68887200590694	-3.45569734587837	1.65100118832395
C	-2.06853001568610	-5.70984746568022	0.88204775088811
C	-2.20712310305657	-5.31525172920857	3.36366760619794
C	0.01652914081369	-5.43243906247495	2.22351124365535
C	4.69925227472958	0.03183388319278	-1.79003391318532
C	5.59983741570350	2.13663415278168	-1.10850145561886
P	0.06958460164142	0.50437309257899	-1.92679119811618
C	-2.24886043589684	3.56046596388091	0.37119064887450
C	0.04458849500992	3.54369302418563	-0.75259188596379
C	-4.37103060150580	3.56453074765511	1.61461051188991
H	-1.52508806775358	-5.50176779355342	-0.04958126134734
H	-2.03088280540225	-6.79532467115923	1.05857207548324
H	-3.11964737565057	-5.42903259710417	0.72862455476874
H	-2.15513587636400	-6.39871354408915	3.54946845878888
H	-1.77653541642067	-4.79548120983641	4.23203722147793
H	-3.26758129557399	-5.03505510485387	3.29206669757079
H	0.03220630609124	-6.52036681664402	2.38281692345548
H	0.61067980187685	-5.21680476299151	1.32422194057757
H	0.51061812287772	-4.96222804410444	3.08654975518901
O	3.83306479182026	-1.03479901876497	-1.54535686804297
C	5.50946419149332	-0.03379894727329	-2.91418579430632
H	5.66977789240211	2.96158281713014	-0.39696045970344
C	6.41822245274093	2.16557800764481	-2.26141271582327
O	-0.13367096260293	1.97577211914020	-2.55109356168980
N	1.45704858559626	-0.10838741829765	-2.22642394914968
N	-1.19896759808947	-0.32307756690571	-2.53643736192467
C	-2.52638020724092	4.89460165963714	-0.02424044190500

C	0.72134974310189	4.65899409680914	-0.15382532226658
C	0.51804099006996	3.07163055761676	-1.96712932462052
H	-5.08958386264253	3.03470958493501	2.24342325436740
C	-4.60382068432732	4.86663532345740	1.22800054492238
P	2.51706453762147	-1.23303085577206	-2.46989121441569
C	5.42346996713568	-1.19815153657060	-3.83493269507879
C	6.37617569647595	1.07437664258062	-3.19257801318340
C	7.26582034161039	3.27386951791917	-2.52942087926621
S	-2.73273496598308	0.20314783495269	-2.86779323239483
H	-1.24616217854593	-1.67347436005262	-2.26066454488254
C	-3.67988248820353	5.52943729077882	0.38841954922681
H	-1.82340612602908	5.41222328040771	-0.67637161885417
C	0.39575897026458	5.16128066600022	1.13337753796967
C	1.81346223332488	5.26460950786568	-0.86100144524624
C	1.60902085021550	3.63612101325599	-2.68271694864689
H	-5.50773184108806	5.38266321791174	1.55468427089271
O	3.05427344158480	-0.88667451956365	-3.95973500334921
N	2.09161107312629	-2.76831912817119	-2.36579872226958
C	4.20212848340981	-1.55867420576448	-4.38903693363598
C	6.57965881187760	-1.96883070215773	-4.18579621094853
C	7.15781666766596	1.16949603319768	-4.37399402123706
H	7.29479697366842	4.09151496160008	-1.80595613808777
C	8.02221933790557	3.32796608356972	-3.67958837952237
O	-3.38441201570825	0.79992632448327	-1.71257184635520
O	-3.35806013477182	-0.92293103702361	-3.54908137798594
H	-3.88280417401278	6.54869571697377	0.05682440982989
H	-0.40775076437548	4.69280137908751	1.69856493364305
C	1.08705567449033	6.22291748213214	1.67983254403161
C	2.49422131709644	6.36610445744767	-0.27656819216775
C	2.21982648938179	4.73952748318832	-2.10954593968721
C	2.10055696573545	3.08411915604361	-3.96479278899686
S	2.51592375580587	-3.76386572370593	-1.17928327290961
C	4.04945596608441	-2.54720948898506	-5.40190474105523
C	6.45228073824287	-2.98881948331819	-5.18605218830564
C	7.83746840250816	-1.79487245890627	-3.55360922629824
C	7.95799465785483	2.26807057650069	-4.61272242105722
H	7.11440827472105	0.36433027718306	-5.10553762789024
H	8.66371797967305	4.18810059533699	-3.87607325289768
H	0.82047966310941	6.58481622127322	2.67399818346656
C	2.14010748997667	6.84017734007015	0.96726777764062
H	3.31598422900564	6.82232371886798	-0.83264010355137
H	3.04421907180224	5.22191116625958	-2.63815715575533
C	1.24385987469292	2.67834902223697	-4.99749150361163
C	3.48307285044823	2.99544619916730	-4.19538281271525
O	2.58662660359897	-3.13523772994740	0.13665089502226
O	1.68919200230988	-4.96964854817919	-1.30646600404209
C	5.19618340629011	-3.22168545343239	-5.79089868434283
C	2.76099148991418	-2.80930144314647	-6.08163146291278
C	7.59479769810169	-3.75049849325163	-5.54743290222119
C	8.92351655521821	-2.56643475700827	-3.91261651209978
H	7.93477578493189	-1.05392170690892	-2.76154096011482
H	8.54260195591385	2.31973535559632	-5.53252394651993
H	2.67487326627117	7.68287426702637	1.40752905984671
C	1.75082138414008	2.22320808684589	-6.21514631180133
H	0.16649208787405	2.73866505596533	-4.86035732479143
C	3.98115155944895	2.54287395312620	-5.41138843886132
H	4.17838856124256	3.26342814224137	-3.39929162266347
H	5.13444560716695	-3.95849279018241	-6.59290392586859

C	2.44971216821765	-4.09894268484056	-6.53919315377824
C	1.85686666397527	-1.77724233934948	-6.39443131914510
H	7.48595543512095	-4.52140302095013	-6.31261093123599
C	8.80745999495304	-3.54487452901932	-4.92593781757672
H	9.87577296920402	-2.42869074784882	-3.39894918535646
F	-1.27756042115596	0.74133156536453	-7.97516556237315
C	3.12951485506492	2.15173840989233	-6.45713991288616
H	1.04721337668652	1.93261084457157	-6.99442255374863
H	5.06307155421647	2.49002761018600	-5.53778512685471
C	1.32154259348611	-4.33777431517954	-7.32530568872824
H	3.10363068218728	-4.93340421346673	-6.27904086266653
C	0.73330802408770	-2.02592885304317	-7.17450423862170
H	2.04639951558494	-0.76152630370107	-6.05139829463004
H	9.67452480607539	-4.14598890367506	-5.20333610904597
C	-1.99039382222060	1.89287975186290	-7.80719474633946
C	3.72020121310436	1.71856147882898	-7.80304473291776
C	0.44531298466725	-3.30340308338560	-7.68223447881415
H	1.13457751535315	-5.35600740748015	-7.66605777481977
H	0.07885482772662	-1.18798169261628	-7.41548556843665
F	-3.17231539794850	1.72058803755284	-8.45949165042293
C	-2.20286834686229	2.21629933360656	-6.34992190893153
F	-1.31634738838376	2.87819305840068	-8.45631016442990
C	4.46453020321279	2.92411161230436	-8.41581055668930
C	2.64202430375646	1.25680567265491	-8.79375293325553
C	4.72005210503647	0.56313064949025	-7.59672487060993
C	-0.74554369871699	-3.50015613742807	-8.62647007340609
C	-2.35264613231203	3.55056634825865	-5.96091311416516
C	-2.29513209565677	1.18748541333314	-5.41105276624749
H	4.90368638768807	2.64593828298152	-9.38598932137335
H	3.77777954572238	3.76775412841364	-8.57655477471624
H	5.27630127625726	3.26859626652868	-7.75987120863449
H	3.11793206051509	0.95868296549129	-9.73910749914265
H	2.08492196496070	0.38791886809995	-8.41543742444694
H	1.92428049321822	2.05818965099136	-9.02190996125279
H	5.52912026776045	0.84415353683869	-6.90919254946617
H	4.22231358575286	-0.32598959549350	-7.18792538805363
H	5.17575232192105	0.28470855343533	-8.55886817370922
C	-0.47622409893742	-2.69273764355336	-9.91514642321672
C	-2.04385039387676	-2.98241845776263	-7.97557021987909
C	-0.94897148021818	-4.97277948043273	-9.00870332644319
C	-2.61256610730806	3.85269347440428	-4.62423091952494
H	-2.26466364018376	4.34907619816545	-6.69571056830999
C	-2.55288119081659	1.51439596966149	-4.08066818736677
H	-2.17826945587798	0.14413724868149	-5.70281487453486
H	-1.30938536891721	-2.81855226926662	-10.62310755368171
H	-0.37116743037494	-1.62014334444854	-9.70112290956394
H	0.44784440363413	-3.03351062587551	-10.40390956758467
H	-2.88391352598930	-3.08967261244398	-8.67765418876001
H	-2.28725848118634	-3.55008296944338	-7.06711095863080
H	-1.97075879950310	-1.92058455063854	-7.70580886726674
H	-0.07657919577179	-5.38153625559718	-9.53864422805483
H	-1.14373699813764	-5.60188143539729	-8.12737801421883
H	-1.81597621507684	-5.06181252210267	-9.67871547009209
C	-2.72620906760465	2.83479942667405	-3.67483862516346
C	-2.72504783217912	5.28795058004588	-4.17046604265596
H	-2.94110993637199	3.05555431820646	-2.62896395312680
F	-3.77431738405032	5.46821571949200	-3.32705842499415
F	-2.88363264475555	6.14608273520479	-5.21052984608903

```
  F   -1.60320360699240      5.67838133609328     -3.49680459173066
  C   4.37218452105264      -5.22168795678063     -2.61497630884781
  C   4.18356383542249      -4.29571037843443     -1.59005424890230
  H   3.51320018191106      -5.61131737693180     -3.16115242777730
  C   5.66578609285333      -5.65289093905404     -2.91113150678744
  C   5.26234874282852      -3.79858060641339     -0.86573711749995
  C   6.76254426580558      -5.15980901337380     -2.20016855720240
  C   5.85151947807389      -6.71137317206608     -3.96794323696334
  C   6.55274239182447      -4.23183058153367     -1.18083220675081
  H   5.08625703223547      -3.08865222519570     -0.05893270116270
  H   7.76970717738243      -5.49009177396884     -2.44461566008154
  F   5.12763134318298      -6.43925306983451     -5.09498187767444
  F   5.43096990051379      -7.93093754292036     -3.52744823052655
  F   7.14694512075193      -6.85094795786368     -4.34848641270086
  C   7.70391152461247      -3.71012568596013     -0.35886484834030
  F   7.66697073446963      -4.20364234202014      0.91218681407002
  F   8.91275497506186      -4.04360873590327     -0.87997362876534
  F   7.66923703370348      -2.35160169879561     -0.24749347893402
  H   0.22272597753106      -5.73377941165680     -2.06872228927780
  H   0.27412268479897      -6.68252456712430     -4.52023866154174
  H   -2.05362663384310      -5.78801981390557     -4.78307896176341
  H   -0.70858185457604      -3.05862260699179     -4.35281806620628
228
TS1-3
  C   0.95754708495624       4.12760011182242      0.28838105568644
  C   -0.32377442755213      4.33943785765121     -0.45665879672842
  C   -0.98749591349929      5.70809663961093     -0.22146525111604
  C   -1.67437285742108      5.79786502938355      1.13811755109089
  O   -0.78204816908176      5.47657356900943      2.20796774967243
  H   -0.09058715413024      4.23350401966957     -1.52993920212651
  H   -1.73881084245885      5.87662107363227     -1.00899376405170
  H   -2.54558546495686      5.12215179899080      1.16103867634790
  C   2.08053608847046       5.05194581262873      0.08342995194210
  C   3.05359000297353       5.21549375557855      1.08823070848915
  C   2.23615341508929       5.76657565685835     -1.12022480920840
  C   4.15427033115942       6.04355922362672      0.88810248511063
  H   2.92684558582101       4.70498853558941      2.04174473670458
  C   3.34230374685077       6.58501940270005     -1.32336298391518
  H   1.51328303924044       5.63537072055395     -1.92367311432377
  C   4.30745360772930       6.72494200638499     -0.32163224363835
  H   4.89600691386069       6.15909036992390      1.67979966014856
  H   3.45848803056605       7.11046358435598     -2.27226380809809
  H   5.17545625693814       7.36588529929879     -0.48353144087545
  C   1.12867680657803       2.98509653307551      1.05869473494390
  H   2.02857591946140       2.88026047385558      1.66755345361923
  H   0.22687580643502       2.50039558643268      1.43777570054495
  H   1.28019999867137      -2.63405863432711      6.72347987323635
  C   2.17960236459307      -2.41270664609681      6.13070072694649
  C   2.38886754022636      -3.43699389270158      5.00665603021230
  H   3.04212852471346      -2.43724627982773      6.81241765460103
  H   2.09467510323598      -1.38888704514754      5.73832900635839
  C   1.22251066327243      -3.45908962212232      4.01294715406975
  C   2.52255865965804      -4.83896728414331      5.63773033761601
  C   3.69842842310314      -3.08863608519117      4.27330414577091
  C   4.11232105549185       2.07755312096333      3.13368617905342
  C   1.26020802519670      -4.31622958094513      2.89957830016034
  C   0.07550457225169      -2.67073750686664      4.17092835874510
  H   1.60144198066412      -5.11801980014140      6.16951474030402
```

H	2.71792818141517	-5.60496391784725	4.87414726448916
H	3.35546048307111	-4.85392911587019	6.35728545982132
H	3.65084668371679	-2.08305396779568	3.83485000322320
H	4.54368478743599	-3.11241622585085	4.97790747362317
H	3.90972815671595	-3.80177417229799	3.46606380616351
C	3.49910498977594	0.85190227327171	2.81129564034902
C	3.50219894119625	2.98530070723700	3.99342682096479
H	5.06934338098659	2.33798914506337	2.68076118945414
C	0.19985200198831	-4.39186337154421	2.00329812316454
H	2.13961974372954	-4.93542395325373	2.71560719640451
C	-0.99243681683257	-2.74282758489482	3.27712731189513
H	-0.00504540631752	-1.97886897440028	5.00859931236881
C	4.18888818012699	-0.10913093613752	1.91915410224004
C	2.25294392545532	0.58401036513025	3.40543845395741
C	2.23670040182280	2.73955934005013	4.55428720154587
H	4.02019358990365	3.92047964687339	4.21426341582371
C	-0.95694931343630	-3.61314321663389	2.17868454731978
H	0.28205315800159	-5.04466038248716	1.13303077821258
H	-1.85809617368533	-2.10609759615424	3.44546080078077
C	3.48580224292359	-1.02095341222533	1.07770165758160
C	5.57360197729836	-0.19158177694814	1.89557072130308
C	1.63654649732267	1.51221685482720	4.24373181915435
H	1.73902845473204	-0.35483760659061	3.21231862528633
C	1.58076786665766	3.77144049591918	5.47514796903916
C	-2.08444708540837	-3.74446087101228	1.22914240280105
O	2.09245260913790	-0.93284886759450	1.06255489831740
C	4.09727638624501	-2.03109088257313	0.34349578543122
H	6.15769658860264	0.45790269230231	2.54926251776734
C	6.26580813261686	-1.09262760815112	1.05544308717010
H	0.65052587414203	1.26504639699512	4.63474568432397
C	2.37896607587681	3.81739756353827	6.79562060542689
C	0.12082389672951	3.41284694920138	5.78983209554559
C	1.60648561153491	5.16686916870307	4.81916791179490
C	-2.80434625406604	-2.61709594801199	0.74527529659070
C	-2.47307424464086	-4.98087891369867	0.74025232903094
P	1.30318299077600	-0.53999107605389	-0.29173961278884
C	5.52717078233978	-2.03194445062957	0.26225555876162
C	3.27716763653032	-3.09684567741105	-0.29510549386752
C	7.68415240806854	-1.09111823656819	0.98218025571227
H	3.42651979953164	4.10226598890798	6.62056931988267
H	1.93462169258918	4.55454476744953	7.48146268068748
H	2.37253053116706	2.83609089793806	7.29192172197062
H	-0.32389227511133	4.20500389287803	6.40893312752772
H	-0.48478885871202	3.31684885039170	4.87737332412951
H	0.04227249584573	2.47080679971297	6.35149331606355
H	1.13864852835964	5.90049853098428	5.49222388770017
H	2.63252613890514	5.51004204295155	4.62407874775417
H	1.05035634822202	5.18357252594217	3.87170156130545
O	-2.43554714092607	-1.35870662170334	1.22483479175986
C	-3.84692513512889	-2.67901801409268	-0.16722572137264
H	-1.96467084218568	-5.87765128929561	1.10021909251693
C	-3.50678456364303	-5.12392101899548	-0.21554016379890
O	2.04518450369469	-1.40348860116832	-1.43031781559407
N	-0.15602948643995	-0.99478140640442	-0.01187956336673
N	1.60411887369269	1.00566049697550	-0.72908294403148
C	6.24195531762309	-2.89873464028553	-0.60351970904604
C	3.45950514353105	-4.48213526727749	0.03487266313841
C	2.24692486118329	-2.76636762000014	-1.15929007770463

H	8.23618772847346	-0.37521870207399	1.59394711563999
C	8.34949777717170	-1.95335351171205	0.13793995109973
P	-1.58421442484200	-0.38391924331597	0.25213106731806
C	-4.49082918040074	-1.42541205352708	-0.64778225807141
C	-4.20900671820882	-3.96462090150784	-0.69044200074172
C	-3.84138042006806	-6.40038444580570	-0.74156707240930
S	2.15432570497512	1.50424584446815	-2.19358985033672
H	1.35816909010361	2.05368899721222	0.10968616957213
C	7.61916748886713	-2.85652818672460	-0.66794777224870
H	5.68702229557006	-3.58956941328079	-1.23706678006826
C	4.41228701940112	-4.93553415581973	0.98402241675665
C	2.59687539753208	-5.45298036229574	-0.57779524073380
C	1.37739575349127	-3.70085714224520	-1.78494334219787
H	9.43879410393356	-1.93418164186545	0.08162404442312
O	-2.32256217922605	-0.56674292322676	-1.17681263587392
N	-1.73981141299612	1.10398722340223	0.80544483779616
C	-3.71087312183373	-0.41558254778526	-1.19886704729895
C	-5.90569149882150	-1.21640227548207	-0.57130158008016
C	-5.19120293279687	-4.14250806347815	-1.69979448229356
H	-3.30446244396921	-7.27339365398915	-0.36424486756724
C	-4.80676887992786	-6.53815193773036	-1.71478221420289
O	1.98719094436237	2.95095396200606	-2.19160857849454
O	1.61450172249396	0.72278627629975	-3.29330787317843
H	8.14767979421447	-3.51644886855500	-1.35686880567878
H	5.05882995093019	-4.21174752657920	1.47619749567232
C	4.52216281177893	-6.27419804199297	1.30074774928901
C	2.74773199833229	-6.82459436904742	-0.24075502610405
C	1.59131308060528	-5.03513935493274	-1.48131868702534
C	0.28699598955419	-3.28432649365247	-2.69334945041657
S	-2.19729698607122	1.48050426365200	2.29547305819909
C	-4.23479808602840	0.75255759086766	-1.82157862676701
C	-6.46449216407363	-0.04289247801339	-1.17928165623308
C	-6.77330561316214	-2.09884984080123	0.12193149703188
C	-5.48074846021189	-5.39515150494334	-2.20119677647044
H	-5.71758361335093	-3.27217658690508	-2.08713072362772
H	-5.04755244565064	-7.52391143822113	-2.11511249760767
H	5.25606950891379	-6.59381371570190	2.04206770188962
C	3.68860247599598	-7.23138450245964	0.67968933386605
H	2.08884657388546	-7.55162556089825	-0.72022311293325
H	0.96100448550543	-5.79235684787463	-1.95189852263948
C	0.46618905516953	-2.29883799159761	-3.67325430874883
C	-0.97340740715913	-3.89968800783624	-2.60889718044349
O	-1.65482005058946	0.60526420631652	3.33189568238713
O	-1.98570148414341	2.92451977062851	2.45376110955022
C	-5.61427243206083	0.88928447819857	-1.81764036873436
C	-3.37283107077173	1.75957763231042	-2.48255277473007
C	-7.86746545725115	0.16681961234894	-1.11417596163203
C	-8.12948041897064	-1.85353175240748	0.18665054034503
H	-6.35218079596276	-2.96884271064856	0.62415704938731
H	-6.23415107006694	-5.50168234400340	-2.98316102461558
H	3.78728008726560	-8.28721058593417	0.93539475960058
C	-0.56876094813569	-1.95415575077176	-4.54171364732065
H	1.42113219433987	-1.78634233708028	-3.76703024536798
C	-1.99929110199307	-3.55360126293108	-3.48114878553123
H	-1.16185418178531	-4.63785744783025	-1.82785794204539
H	-6.06632014569620	1.74779451693608	-2.31635393270801
C	-3.73282727762545	3.11867304571783	-2.46529567689754
C	-2.21713427556474	1.40926786127254	-3.20077677776691

H	-8.28236389080428	1.06079415904730	-1.58364798947941
C	-8.68561216921903	-0.71850668177159	-0.44619350228841
H	-8.77495476484683	-2.53484043952336	0.74243039152301
F	5.78892728279495	-2.18419635677918	-3.80021792324696
C	-1.82240505361690	-2.57536270673856	-4.47480490969233
H	-0.37707613340821	-1.18140248163599	-5.28530496422575
H	-2.96280123453978	-4.05317708037892	-3.37105677221701
C	-2.99651000487851	4.06931224861720	-3.16761950482163
H	-4.60151794384297	3.43741125613537	-1.88634022133870
C	-1.47856619776249	2.36910569076767	-3.88999622648939
H	-1.88588975347765	0.37327452236175	-3.23870901754208
H	-9.76005856907593	-0.53758010788820	-0.39169678648443
C	6.46659509501761	-1.00385975259921	-3.83368598663036
C	-2.96307770337692	-2.24874225365158	-5.44454520365353
C	-1.85545202985794	3.71930391800992	-3.90929681147069
H	-3.32984386583593	5.10781218075841	-3.13634812097241
H	-0.58556023092176	2.03853758368188	-4.41843617035079
F	6.42262691892014	-0.56688719023792	-5.12580388018978
C	5.86340497513698	0.01681523588363	-2.90069137402479
F	7.76629729373801	-1.26870628874087	-3.54821865900825
C	-2.59503102677730	-1.12223362637164	-6.42034719821066
C	-4.21563288536753	-1.81606324830274	-4.65820923675186
C	-3.29323325549330	-3.51770519544566	-6.25837914852142
C	-1.11842101339054	4.75491275338578	-4.76689929797085
C	6.67081623428982	0.79323468285100	-2.07236155508162
C	4.47909814644314	0.21272133785058	-2.93165831172509
H	-1.73287055353245	-1.38988658218453	-7.04846587774104
H	-3.44563390078526	-0.92590682778437	-7.08900219704226
H	-2.36410049960378	-0.18571056366102	-5.89184611465610
H	-5.04612851009598	-1.61678459531975	-5.35239082932992
H	-4.54027098727812	-2.59588498650746	-3.95730794669655
H	-4.02687849566332	-0.90017674777730	-4.08269029332111
H	-3.60635597950270	-4.34256625726936	-5.60298983519689
H	-4.11230585467889	-3.31327337386368	-6.96474691855894
H	-2.41760571999236	-3.85410535015296	-6.83227917928440
C	0.28410794877264	4.27353548935303	-5.17203948413230
C	-0.96695294695639	6.09541559281698	-4.02541771981370
C	-1.95524101161662	4.98616652219831	-6.04426284815056
C	6.08656722999058	1.78273793370948	-1.27403708611774
H	7.74602157853044	0.62680444695689	-2.04200796104953
C	3.91995207910696	1.19938974173869	-2.12824681230186
H	3.83691118593023	-0.38506045990446	-3.57728638427785
H	0.24236603402382	3.39130431080941	-5.82594806866963
H	0.79608640944401	5.06994011431835	-5.73182153741789
H	0.89713614147830	4.01750339709656	-4.29616107468274
H	-1.93592300506614	6.54164802737925	-3.76388312016828
H	-0.38955779631905	5.97526849129710	-3.09849028364052
H	-0.43581821626545	6.81577290547242	-4.66481735859881
H	-2.95794477949362	5.36418652740205	-5.79804090017877
H	-1.46032379565751	5.72042045226875	-6.69857659753486
H	-2.07522757110901	4.04846336180206	-6.60555886302577
C	4.70981883619815	1.99610837118617	-1.29733034028358
C	6.96384775351490	2.68357974850794	-0.44167236074431
H	4.25507534297188	2.77852639294387	-0.68878526369274
F	7.35367186019300	3.78394114046080	-1.14668139354206
F	8.09854759387458	2.05895946684791	-0.02752882278902
F	6.32403267084642	3.13884553774356	0.67071998506287
C	-4.53894536277432	0.17645312134672	2.97667992565037

C	-3.98301235366642	1.26811245207523	2.31728526682631
H	-3.88635568687603	-0.54050272580154	3.47254767470400
C	-5.92796758935592	0.02699289420304	2.99668858615364
C	-4.79072358962486	2.21566103958384	1.69001914151212
C	-6.75343950504069	0.96590984814052	2.37940311327262
C	-6.51145288002192	-1.14713026687887	3.74084146325056
C	-6.17694989939573	2.06151025469368	1.73225258805903
H	-4.33689496792059	3.06962944661391	1.18741545888486
H	-7.83411542587361	0.84238242588924	2.39408405273494
F	-6.33161461940718	-1.01803928585219	5.08704534949089
F	-5.91395884658043	-2.31884502951058	3.38149643896830
F	-7.84503115029539	-1.29048208677252	3.52964963261433
C	-7.04560000159292	3.13539200440397	1.12908795246301
F	-7.05789817176569	4.25599212569951	1.90640188353760
F	-6.59660192610067	3.52640651188310	-0.10067440611406
F	-8.33598095899799	2.74281674182531	0.97800661418850
H	-1.00453288612665	4.56056207730252	2.47307714821782
H	-2.04102027519406	6.82546689936220	1.29864630044650
H	-0.23918633078449	6.51031573285544	-0.30965208593184
H	-1.02328012485641	3.52546650054453	-0.21780339416728

228

TS1-4

C	-1.16890256413186	-4.32086384801995	-0.17961821815726
C	-0.15818741841689	-4.67826558422125	-1.22607217153017
C	1.31308044436808	-4.68247099840271	-0.80042143242237
C	1.61883743964209	-5.81842488530462	0.16834472829679
O	0.79249725760584	-5.78857561189073	1.33197644637599
H	-0.42537463826795	-5.64114868254694	-1.68438496588637
H	1.58772690614360	-3.71557116802823	-0.35547538343341
H	2.68887023968301	-5.79264412077227	0.44776738727386
C	-2.38304586553770	-5.13837623695986	-0.07753613736092
C	-3.62242714659468	-4.53691371867112	0.20498604704704
C	-2.33453743949760	-6.53578017406296	-0.23216450832492
C	-4.78097907970280	-5.29960403730549	0.30452386417728
H	-3.66652005134585	-3.45684949273122	0.33549283996086
C	-3.48868704604014	-7.30354748622783	-0.09506874442129
H	-1.37644220988913	-7.02518206025810	-0.40537750102092
C	-4.71705384980141	-6.68853236142358	0.15885425535181
H	-5.73805717555763	-4.81046664122920	0.49428638968094
H	-3.42995896951441	-8.38854873334718	-0.19131228696362
H	-5.62322386781003	-7.29017033088984	0.24421599202959
C	-1.03989139553121	-3.16865212152565	0.58507798295444
H	-1.68182005237659	-3.05875241316706	1.46162596230139
H	-0.03837467390657	-2.74048837429385	0.67979083278840
H	-3.42078624454970	4.02793999392143	6.88313864096032
C	-2.58074317931099	4.06827045469402	6.17292067139326
C	-2.51611897904686	2.76472462245335	5.34919398652157
H	-1.65258230700393	4.21695565896669	6.74342309876860
H	-2.72337140802466	4.94443302323055	5.52481374142015
C	-1.33876074198920	2.86537126866358	4.37341747239924
C	-3.83512859756075	2.59620357831916	4.57001500513493
C	-2.37399839611928	1.58126847833935	6.31657716704641
C	-2.40529914473764	-0.96969691778112	3.25208672018040
C	-0.22241617520234	2.02140378982966	4.43595658791507
C	-1.33232154317031	3.85827215810335	3.37928920695896
H	-3.83440679092654	1.66582620225744	3.98698776960580
H	-4.68410599242180	2.55902310621702	5.26936398706285
H	-4.00497368731171	3.43067849774440	3.87670478656806

H	-1.47257237244920	1.66936254532112	6.94012732729938
H	-3.24274210765468	1.55344498674135	6.99022309455537
H	-2.33553883534640	0.62063624426159	5.78280421928636
C	-3.64215359734971	-1.08834897832474	2.59385850217804
C	-1.84982311764281	-2.04569794126594	3.94386297774150
H	-1.85243045706867	-0.03327796300417	3.22166549397754
C	0.85838751558067	2.16792466185822	3.56629473170580
H	-0.17506373669607	1.22619859567487	5.17928761317938
C	-0.26072750321662	4.00861238088355	2.50686924174534
H	-2.18519545927748	4.52949878489076	3.27049396233478
C	-4.25787843523364	0.02084836727685	1.83039875094021
C	-4.31356832386864	-2.31941601611735	2.70351183625714
C	-2.49988817477770	-3.28529818684539	4.02233959231462
H	-0.86779209319583	-1.90795887738365	4.39494515714187
C	0.86699393993516	3.17347941663995	2.58862628628994
H	1.69775386856352	1.48278082962054	3.65933728433671
H	-0.30941981128350	4.77221001586158	1.72938132629504
C	-3.48310061564513	0.97112664739565	1.10471026467811
C	-5.63305495419873	0.19547669094147	1.80397138403853
C	-3.75763244454275	-3.38442720893717	3.40315368157780
H	-5.27051034449003	-2.45705712027013	2.19674524239190
C	-1.89575100215702	-4.49905021496615	4.73204056071725
C	2.01362771866822	3.40279590852142	1.68136037319544
O	-2.09723843848124	0.79695479702177	1.10322838470161
C	-4.01431003625508	2.08399006205447	0.46413228025085
H	-6.26785104258989	-0.48477703995996	2.37345494060945
C	-6.25098467733402	1.22577412655633	1.05963240613513
H	-4.30332479522456	-4.32849768227913	3.43347183095137
C	-1.85889724423587	-5.70046886252412	3.76441450535873
C	-2.77951260685627	-4.84987247475634	5.94717778004023
C	-0.46583729222518	-4.22717097553276	5.22036815509958
C	2.74924488010108	2.33890008550044	1.08851233647300
C	2.41461046684235	4.68780956165753	1.35233368992688
P	-1.32962186402248	0.44745797118108	-0.27304793426765
C	-5.44057666615892	2.18922815449728	0.37207067211936
C	-3.11480979593155	3.14207135457410	-0.06991712472878
C	-7.66518207194326	1.32611120276032	0.97566425634249
H	-1.22860691254964	-5.49444296537752	2.88913155486723
H	-1.44069369617498	-6.57871819746924	4.27832911161310
H	-2.86134660438427	-5.96974630436049	3.40330250376700
H	-2.82131988573710	-4.01112011917437	6.65731222784352
H	-3.80847350934451	-5.08721022224624	5.64125478027101
H	-2.37037573558375	-5.72636339327973	6.47201313438246
H	-0.43538123919691	-3.42133307707800	5.96828825617566
H	-0.06339791775951	-5.13442689819627	5.69325836333176
H	0.20570771416156	-3.95298285205754	4.39414787217346
O	2.38038091697678	1.03359415462189	1.41940839701548
C	3.81666664052773	2.50875513931708	0.21922425105552
H	1.89943395896348	5.53658523931029	1.80625949018146
C	3.46967511172341	4.94252752035917	0.44548540351372
O	-1.96435526004473	1.47777918207872	-1.33645932701354
N	0.15970187652036	0.72179067944307	0.04883004697970
N	-1.80655770613918	-1.01259041833271	-0.84788616523903
C	-6.08550818082418	3.18522330308857	-0.40511351774370
C	-3.22336204121446	4.50446081324253	0.36927881494520
C	-2.09032681232570	2.82109000383674	-0.94655907108985
H	-8.27158750728701	0.58909860905663	1.50569127305057
C	-8.26123814537354	2.31365388844496	0.22158428909734

P	1.59303765183004	0.13835161619141	0.32487077840768
C	4.49900526788683	1.32781746316192	-0.37691598778076
C	4.18322680493840	3.84689640924387	-0.14733973570448
C	3.81607413389709	6.27249313487303	0.08518548743872
S	-2.32333310677830	-1.31764631428335	-2.38377621575119
H	-1.47735548579194	-2.14599076345834	-0.19364981522272
C	-7.46182794755190	3.24361875061592	-0.48156615463375
H	-5.47868887864577	3.89869953010170	-0.96078156257413
C	-4.15603036518168	4.93222017445919	1.35014962765052
C	-2.30864256321608	5.47345809471856	-0.16418435820853
C	-1.16178803402837	3.75335379080900	-1.48493879268003
H	-9.34859530127419	2.37258013637604	0.15625262563745
O	2.37044923263382	0.48005085849841	-1.05242522515775
N	1.77076365391866	-1.38879593619586	0.74566928478669
C	3.76175125904076	0.37132506965605	-1.06506211045866
C	5.91770063445213	1.15129092735697	-0.28961325696740
C	5.18309186506531	4.14215143054790	-1.11105133694157
H	3.27109749881577	7.09492775280378	0.55360893028695
C	4.80274901236850	6.52366274339820	-0.84271560007991
O	-2.38641611034841	-2.76946474321634	-2.48147634749673
O	-1.58286218395935	-0.56116504187349	-3.38070965117504
H	-7.93545883104175	4.00633860624850	-1.10085051183847
H	-4.83591780488468	4.20529452491618	1.79017560390848
C	-4.20457909675362	6.24811687662438	1.76233306867127
C	-2.39667436883118	6.82347497406140	0.26965971100721
C	-1.31404897234295	5.07144360576437	-1.08558404220434
C	-0.07390153333871	3.36080108042704	-2.40720176223616
S	2.19090419732508	-1.90059626269925	2.21031202212232
C	4.33591246651012	-0.68326457821321	-1.83317314049153
C	6.52623698914356	0.08729569652551	-1.03414231886695
C	6.74130912719734	1.95918350390889	0.53534564789852
C	5.48388600539319	5.44516103133149	-1.45138347437934
H	5.71333148701585	3.32330932253375	-1.59374710687618
H	5.05384489588831	7.54973124877894	-1.11494469816537
H	-4.92560421008782	6.54768436361458	2.52441352847456
C	-3.32455713892275	7.20760605110267	1.21246286296162
H	-1.69979303490856	7.55022837803740	-0.15317445875785
H	-0.64065748389493	5.82797304173504	-1.49295680612916
C	1.20014971404513	3.93661088394481	-2.26617956156484
C	-0.26359512871430	2.45312537570372	-3.45822284873157
O	1.60782605842059	-1.13061301737723	3.30564936887731
O	1.99961438083785	-3.35470774209763	2.22850668071247
C	5.72026666873889	-0.77042658850488	-1.81633066479727
C	3.52793018905105	-1.61618469665416	-2.65384061457894
C	7.93372017847007	-0.08860625554715	-0.96280634261172
C	8.10298515166373	1.74684408653957	0.60089726654518
H	6.28217485750113	2.74206987097368	1.13753335171803
H	6.25186834860037	5.64273393706309	-2.20081208679239
H	-3.37630783438111	8.24612656428383	1.54214992131477
C	2.22622153542169	3.63361699520306	-3.15348286500564
H	1.39702560914610	4.61085851349734	-1.43147524133086
C	0.77265427551486	2.14932021419883	-4.34063172872867
H	-1.22794240931203	1.97075064835468	-3.59979734794124
H	6.21508156055409	-1.53015860056908	-2.42237862047043
C	2.31509676025731	-1.24633338151548	-3.26059316130615
C	3.99950123651859	-2.91755416216544	-2.90954948888134
H	8.38673362137528	-0.89951653090141	-1.53633402538288
C	8.70861817180959	0.72292447557808	-0.16280481087451

H	8.71433603960143	2.36618944206358	1.25842634892189
F	-5.83541935969740	1.88177003457977	-5.27862156080722
C	2.03852245433023	2.73688864472702	-4.21873077968634
H	3.19778891917686	4.10444768554986	-2.99911903981783
H	0.57209170078481	1.43717545977418	-5.14030980803427
C	1.61393791697630	-2.13280825204282	-4.07762557698855
H	1.90511228472854	-0.25043410050390	-3.11192604301047
C	3.30732107752575	-3.78624993576098	-3.74670338177525
H	4.91683246757523	-3.26300745199358	-2.43032173924486
H	9.78693442994031	0.56770689215457	-0.10568368832798
C	-5.98540714925520	2.10945562540230	-3.94260365988609
C	3.17852662670629	2.46880499943451	-5.20683287286518
C	2.09238436028549	-3.42016015546007	-4.35195941162101
H	0.67020362310273	-1.79120909271940	-4.50135151346164
H	3.72390047041944	-4.78024511910462	-3.91998132805187
F	-7.26003330527607	2.53310705333524	-3.75044081647979
C	-5.65735704156787	0.87307143417153	-3.14254032059767
F	-5.15763524391652	3.14571329968793	-3.62999276196554
C	4.44098670436974	2.01139557524698	-4.45091836301788
C	3.48825984339277	3.78146189480908	-5.95731851253029
C	2.81711896541253	1.38757657153229	-6.23508259626299
C	1.37149287073409	-4.38172169093727	-5.30201949688953
C	-6.65928322630897	0.14952688257359	-2.49623734258368
C	-4.32895577670705	0.44239579268382	-3.10424230495328
H	4.76567680719349	2.75705615486869	-3.71307662320804
H	4.26486954024904	1.06550406211086	-3.92252595096704
H	5.26793605141318	1.85637470986607	-5.16028913424808
H	2.60553587734803	4.13466745740012	-6.50984055884062
H	3.79251104576336	4.57610714904515	-5.26154460630175
H	4.30678105200015	3.62442843035098	-6.67639419762547
H	2.58859357283965	0.42705230091267	-5.75054129784972
H	1.95561358427869	1.68107594315441	-6.85238962269147
H	3.66953392310614	1.22651068806520	-6.91088753279122
C	-0.03003301418678	-3.87823149589463	-5.67859557903901
C	1.22371166999118	-5.77416123561621	-4.65790494593354
C	2.21639607070410	-4.51026934449283	-6.58757787849889
C	-6.32471532566364	-1.02230214466152	-1.81356166002416
H	-7.68979144634918	0.49751174733295	-2.51862317037244
C	-4.01602630921093	-0.72225183774021	-2.41051034648852
H	-3.54127176489502	0.99611688331102	-3.61290093149358
H	0.01095296822843	-2.92866654421172	-6.23085480899103
H	-0.52162706868087	-4.61660111964760	-6.32848044837501
H	-0.66541339851129	-3.73093701611708	-4.79294575966361
H	0.73104241866104	-6.46084435932255	-5.36220548042036
H	2.19488454191619	-6.21277690404310	-4.39097566843347
H	0.60943610723351	-5.72323640760398	-3.74878618309971
H	2.33483298832370	-3.53209892088834	-7.07553751701367
H	3.21968949509761	-4.90241445009940	-6.36802280772713
H	1.72839298632506	-5.19469606189571	-7.29828268588790
C	-5.00371223983759	-1.46802884315391	-1.76933707516653
C	-7.39197117710149	-1.86828786269066	-1.16619707520591
H	-4.74391882477763	-2.39613399522560	-1.26121406463838
F	-7.61750055464775	-3.00955927562656	-1.87635378111150
F	-8.57846791806176	-1.22222119035240	-1.05806565547293
F	-7.03068196563052	-2.26490157787038	0.09201707838407
C	4.80219571068082	-2.51182546831079	1.55739532476597
C	3.97126534853341	-1.66167682430754	2.28630347578197
H	4.36950692724816	-3.30628555187814	0.94924639049808

C	6.18447861478126	-2.33772688744844	1.63178659129339
C	4.49948186890678	-0.64692000192150	3.07750305238360
C	6.73408490312351	-1.31827304209539	2.41335096743067
C	7.07858975235486	-3.31409755387261	0.91125969679015
C	5.88548775508272	-0.47625645350326	3.13071220717213
H	3.82848597064941	-0.00543806208566	3.64715282224909
H	7.81196913091574	-1.17712073344250	2.45376873155983
F	8.36088674540352	-2.87932612603659	0.81648337074375
F	6.64274818085523	-3.56605981234180	-0.35844093498563
F	7.11217673344045	-4.51820716973616	1.55111158159702
C	6.43977112595738	0.61058764415319	4.01637559530223
F	6.24185822429673	0.32284281061546	5.33499162369592
F	7.77398303793870	0.79584754613362	3.84534244391088
F	5.82989504396466	1.80830736095270	3.78618307752876
H	1.03568840547221	-4.96410109683211	1.80575274618838
H	1.42853269838976	-6.79206947015166	-0.31297548159399
H	1.93838750125204	-4.79366457592284	-1.69900083681896
H	-0.29941057837203	-3.92700928015770	-2.02406019942364

228

TS1-1’

C	0.14941323787541	-4.41026946214439	-0.81157055844671
C	0.49324290974072	-5.66309673084176	-1.55506659404388
C	-0.77548814980113	-6.48046113283091	-1.90752828607213
C	-1.83832243109286	-5.68070524384267	-2.65262844583548
O	-2.45199743514897	-4.74601720975968	-1.76395875431854
H	1.02221663849773	-5.39428747358371	-2.48039898035230
H	-1.22838490093546	-6.87991952575085	-0.98682970233849
H	-2.60143216220595	-6.37789358230407	-3.04670877036953
C	-0.31085135603640	-4.50042119992982	0.57499594750443
C	-0.09543032687650	-5.66323793646572	1.34368734794442
C	-0.89666038645556	-3.39002124414029	1.20912923013183
C	-0.42462989850006	-5.69506946768045	2.69433038541335
H	0.37431136051337	-6.53715601048900	0.89471397391996
C	-1.22268523193125	-3.41840270649616	2.55744858428478
H	-1.11724835224546	-2.49687131307562	0.63252042418962
C	-0.98035335318883	-4.56899700174699	3.30842552376341
H	-0.23335520011371	-6.59774747341909	3.27609815703395
H	-1.66703082350877	-2.53679493644472	3.01619626155668
H	-1.22656172653154	-4.59253482430710	4.37148393011844
C	0.36416401900140	-3.18290910525563	-1.42704990366125
H	0.69704929360963	-3.18260829511599	-2.46645010187903
H	-0.29808454110911	-2.34872168826616	-1.16697160904508
H	5.20980109780950	2.03742245724744	-5.81132336085419
C	4.52621314475165	2.08742792353728	-4.95031867456363
C	3.29291835561707	2.93919775914696	-5.31251006733976
H	5.08429234312480	2.50822447023384	-4.10270299471144
H	4.22895271795443	1.06376111827397	-4.68615390141222
C	2.36649037589295	3.07373955955689	-4.09964753413444
C	2.59160557379832	2.27434097660452	-6.50557794052690
C	3.75484170111558	4.35057676527381	-5.73204799644134
C	4.08276619655316	-1.92381884514826	-3.26999776642414
C	1.00345470466508	2.75320332238612	-4.14476691135021
C	2.86334189348547	3.55872676688261	-2.87793945853738
H	1.73460946312566	2.86528198960207	-6.85982027596350
H	3.29958280622063	2.18295326670416	-7.34198662800572
H	2.23873800570706	1.26395150397985	-6.25197458704564
H	2.89439956952572	4.98053421117348	-6.00033874961801
H	4.29977345389548	4.85092366952464	-4.91906080579897

H	4.42395786619301	4.28882244868055	-6.60383297158800
C	3.48270163647995	-0.76174925055975	-2.75982687854833
C	3.47742607294476	-2.66682493040101	-4.28437306292539
H	5.03776570141514	-2.25998869761975	-2.86255208657838
C	0.17764302517051	2.89830926396299	-3.03048207471982
H	0.56175065295034	2.36371878057547	-5.06097394157520
C	2.04324868013039	3.71801101837316	-1.76720894232307
H	3.92063304353681	3.81426588781475	-2.78352590741999
C	4.19477590352611	0.04805609757089	-1.74703963918980
C	2.23198108530875	-0.39341961425490	-3.28739895113181
C	2.24612047343362	-2.28397663842913	-4.83352402837195
H	3.98640748155208	-3.55978008341943	-4.64663920892398
C	0.67885202541805	3.38812424312774	-1.81532259928872
H	-0.87187367472770	2.62733920481000	-3.11902564511525
H	2.47342799124229	4.08572414833686	-0.83654148295107
C	3.52793720732409	0.71389749887137	-0.68194855490660
C	5.57268466172494	0.18885135671197	-1.79576767689090
C	1.63679498220840	-1.13695320512578	-4.29922201274447
H	1.72630653722527	0.50038651647903	-2.93338883429378
C	1.56086711767665	-3.04372321264485	-5.97238885251626
C	-0.19559478369018	3.64606281287418	-0.64932246111745
O	2.13013944599050	0.62583095922543	-0.63797590122982
C	4.16727105207222	1.44993651867338	0.30782589284536
H	6.12370632590718	-0.25897812378968	-2.62373353114918
C	6.29852327934154	0.87506830769538	-0.79610922678320
H	0.66359377001156	-0.80736867963303	-4.66427524829435
C	2.38338134731315	-4.24881728753104	-6.44719488236925
C	1.36538935385715	-2.08381222379437	-7.16484555720085
C	0.18397827075860	-3.54727205162248	-5.49557053475332
C	-1.31585872090577	2.82654133013158	-0.32547654486661
C	-0.00113278957234	4.76520269518433	0.14484509781528
P	1.50049155929193	-0.35112963649979	0.48140822168345
C	5.60036845000168	1.48863133227491	0.29694858756190
C	3.36561965947128	2.16494205353533	1.33679810548463
C	7.71682754260286	0.93692655879114	-0.83384144756374
H	2.53283676678423	-4.98284957862442	-5.64142278116815
H	1.85309127846231	-4.75685533163674	-7.26543929468472
H	3.37069909053322	-3.94597067148738	-6.82568609588506
H	0.87885397037019	-2.61075047987851	-7.99945683508955
H	0.73404092920103	-1.22723362796669	-6.89253315230726
H	2.33215614987014	-1.69597994635291	-7.51774448113423
H	0.30315233381986	-4.27175279144133	-4.67649488683060
H	-0.45538297987457	-2.73133831430572	-5.13249845783201
H	-0.34255566960015	-4.05278617756059	-6.31929383387082
O	-1.47057606865833	1.62721694157208	-1.03215244028726
C	-2.28724280695715	3.16946982786787	0.60198633063363
H	0.83593788670028	5.43156892583353	-0.07019450224946
C	-0.86254408531353	5.08948932877044	1.21828500510245
O	2.23303874766532	0.11324216408918	1.83976982374042
N	-0.03181751257435	-0.10746589024858	0.49322371784865
N	2.03625331485001	-1.87594917771950	0.24356511175395
C	6.36269546677905	2.07489152143221	1.33974083131478
C	3.51403890915058	3.57564720048059	1.55932515932611
C	2.39395818492940	1.49047556475787	2.06574170928490
H	8.23534601622047	0.47204650181468	-1.67449299092643
C	8.42651797875146	1.53941109990112	0.18219683507213
P	-1.38507662291942	0.21400220374781	-0.24329287162203
C	-3.56414898591220	2.41348959065376	0.69348012407200

C	-2.03817067075094	4.30115356898050	1.44758028383357
C	-0.59617421125330	6.19791626405098	2.06498036989750
S	2.78282756271703	-2.84904791794198	1.35250412188977
H	1.32232556552217	-2.60834260583727	-0.64025035408965
C	7.74117204904434	2.09872656153314	1.28432772021725
H	5.84717971884846	2.49731887844090	2.20068379820036
C	4.37702976653450	4.39712974519466	0.78555415617617
C	2.71314556322491	4.20190873449963	2.57123003500079
C	1.54830985519184	2.09502169078702	3.03555212158852
H	9.51645017443481	1.56914608464755	0.14706546271974
O	-2.37614239996699	0.36380933415293	1.03219649901803
N	-1.89246198451745	-0.91132394831383	-1.24249580967814
C	-3.59162409087294	1.04396920813622	0.92601982322726
C	-4.81108539861357	3.10812958387822	0.53400024276888
C	-2.87558364236623	4.63850191771183	2.54286801469373
H	0.29648753075546	6.79488693716672	1.86933606687337
C	-1.43048944854928	6.50067137392857	3.11913559592935
O	2.86004322070678	-4.15084538297030	0.70522888018310
O	2.20218277579181	-2.71725030661130	2.67874861742950
H	8.30571573470409	2.54092106813755	2.10609625257253
H	4.95957077668001	3.94823431657812	-0.01726684935196
C	4.47693539469518	5.75199852225968	1.02596938412380
C	2.86155701332861	5.59399056541188	2.81297976331298
C	1.75913863905440	3.44210011709794	3.28372785880909
C	0.45039402391638	1.37245275112561	3.71726966529968
S	-2.36596637776206	-0.85882990387623	-2.76844131892012
C	-4.79406530874909	0.32123210305289	1.19028877184222
C	-6.03192215186679	2.40120647436493	0.78370408501250
C	-4.89166197834865	4.45464434719503	0.09321891458366
C	-2.57400690913042	5.70571706730120	3.36378525061687
H	-3.75558519567543	4.02868514205907	2.74388679193468
H	-1.21023093107285	7.34947287396222	3.76834843188055
H	5.14094939322743	6.36094184207538	0.41081364345640
C	3.72616481120021	6.35712355501109	2.05985241273261
H	2.25758753266408	6.04807775849686	3.60090990606200
H	1.15017894055937	3.94346025110637	4.03781693769758
C	0.56903696738558	0.06771692391216	4.22531693797036
C	-0.77741277725785	2.02758677883930	3.88847137394978
O	-1.45423991032081	-0.12174925731056	-3.63886874953583
O	-2.70709238265148	-2.23652130049261	-3.14462404597780
C	-5.98026367572072	1.03620910498022	1.14252963375995
C	-4.83983501490238	-1.12176041424241	1.52292759957259
C	-7.27226355083163	3.07403289489198	0.62544274181310
C	-6.11258370621097	5.07667314242731	-0.06609576285106
H	-3.97573214157494	4.99323731590316	-0.14421222333957
H	-3.22274749144509	5.93626788239912	4.21003880715343
H	3.82593694105313	7.42668647104866	2.25042779946102
C	-0.49362789246003	-0.52887451837600	4.89817031534066
H	1.49444433458116	-0.49237692899986	4.10194032236245
C	-1.84107890815190	1.41526487104260	4.54618857696283
H	-0.91524198845309	3.02596863376672	3.47448381431720
H	-6.91771806557488	0.50750446162288	1.32313419993303
C	-5.66555002039189	-1.57074853323268	2.56931390607041
C	-4.15652271254962	-2.09027489919638	0.77876387238098
H	-8.19249662512451	2.51894702419180	0.81731835826263
C	-7.31476558259631	4.38758511610775	0.21172904492340
H	-6.14967378759572	6.10548619844053	-0.42655237416915
F	6.55500268469040	-1.24253405913641	5.09663226822712

C	-1.72412499043938	0.12548090361946	5.08031226033592
H	-0.34810939478351	-1.53608008059189	5.29211324051225
H	-2.77740848115289	1.96552346263050	4.63420470022230
C	-5.78269423217896	-2.92693954286012	2.85664633346207
H	-6.20313408399107	-0.84285039177012	3.18015622772322
C	-4.28939951790007	-3.44911184929917	1.06115142624411
H	-3.51181280716458	-1.79029706100584	-0.04413084815682
H	-8.27314937464053	4.89255930654775	0.08288226364123
C	6.59735443291181	-0.42166061435448	4.00813363820627
C	-2.84856803413238	-0.53542744134740	5.88556270871242
C	-5.09431246629676	-3.90085345295403	2.11281772570144
H	-6.41905392067861	-3.22930546754157	3.69058472205464
H	-3.73690566522088	-4.15355909249715	0.43986181147372
F	7.85851307031875	0.07591959093158	3.93502160453489
C	6.19057440079306	-1.15836903537704	2.75649845124304
F	5.76144954885299	0.62058114214374	4.27570882531745
C	-3.10254330041769	-1.97243650049764	5.39440404600869
C	-4.16808496936914	0.24356518197743	5.77964705078595
C	-2.41617808542914	-0.57757603266848	7.36689296693766
C	-5.22960537404381	-5.38413152439290	2.46695127346276
C	7.14100342012216	-1.53578773646694	1.80790087905585
C	4.84990111231653	-1.51640434017820	2.60123613714242
H	-2.20497962815441	-2.59913434293664	5.48494067525887
H	-3.89342541573227	-2.44097260245654	5.99935479167684
H	-3.43335645234123	-1.98424598120130	4.34741150906560
H	-4.08615971027790	1.25128938997739	6.21168207116232
H	-4.49571889248935	0.33843790060124	4.73424562934532
H	-4.95457651011033	-0.28993490397880	6.33286470186865
H	-2.23400128727307	0.43664588257838	7.75066436570713
H	-3.20152185644018	-1.04482224689236	7.98080944763664
H	-1.49132601558465	-1.15786355210283	7.49384975623546
C	-4.79578414278094	-5.59794861269586	3.93184732153886
C	-4.35361015515502	-6.27182929544879	1.57051935955998
C	-6.70155927455867	-5.81237091204492	2.30068977089997
C	6.73982649931658	-2.27037130982190	0.69028789479906
H	8.18464574440382	-1.25666994789333	1.93522918488787
C	4.46992063672590	-2.24668076580065	1.47847589528634
H	4.10430320265470	-1.25349377012838	3.34990278101440
H	-5.41434047284459	-5.01640499485285	4.62959791823563
H	-4.88826649048421	-6.66062878207873	4.20295848717604
H	-3.74832393608833	-5.29611584051888	4.07257901710870
H	-4.48037049848078	-7.32438978395203	1.86373905657180
H	-4.62945710770489	-6.18183333561511	0.51014300614000
H	-3.28786103329753	-6.01999936947408	1.66753885614235
H	-7.36903476325282	-5.22618086804759	2.94801086055335
H	-7.03212025260628	-5.67430342502409	1.26115794166877
H	-6.82100410393997	-6.87470180421007	2.56287966790457
C	5.40170924816727	-2.62752346097023	0.51440686950298
C	7.76065306811194	-2.76126661350850	-0.30502553764471
H	5.08372390852186	-3.22194220264216	-0.34200247554997
F	7.34560211150341	-2.58103672848391	-1.59385495753882
F	7.99000529620004	-4.09685286845875	-0.15833227644021
F	8.95784790352006	-2.13512591960335	-0.17834503246781
C	-5.08238736168020	-0.55229121151567	-2.31816186259841
C	-3.90990293520503	0.06022855485256	-2.76242246737015
H	-5.07199788228081	-1.59517114132398	-2.00324656888960
C	-6.25958777687146	0.19425095489397	-2.27714554421903
C	-3.90719556109720	1.38556657871290	-3.18518953943324

C	-6.27302638299742	1.53250900562472	-2.68189926516594
C	-7.54438675157961	-0.46602623061124	-1.84275100855667
C	-5.09526270573297	2.11918195730558	-3.14079929842168
H	-2.98250099975424	1.83118400921748	-3.54812228321311
H	-7.19173182637436	2.11401044290011	-2.62955151214807
F	-8.20018074545036	-1.01971019812746	-2.90380554579434
F	-8.40696879722113	0.42177467501970	-1.27109086679046
F	-7.33317499607530	-1.46034622931473	-0.94456454126308
C	-5.08041019527228	3.54123401816154	-3.64159759029449
F	-4.03176603913484	4.24505756563559	-3.12928692377131
F	-4.94742100918278	3.58253950349021	-4.99981313697587
F	-6.21578241134871	4.21586940026496	-3.33263507736292
H	-2.54665088467431	-3.88479785918243	-2.22895215523996
H	-1.39253916825699	-5.15987914488876	-3.51711472727623
H	-0.47042232209341	-7.34256130648161	-2.52195944025223
H	1.17412269581512	-6.29354281553925	-0.96327226011490

228
TS1-2'

C	-1.241367	-1.829752	3.850322
C	-1.422670	-2.807840	4.966844
C	-2.915829	-3.206462	5.136299
C	-3.602267	-3.668036	3.856223
O	-3.901079	-2.535518	3.040398
H	-0.839987	-3.710494	4.735912
H	-3.488423	-2.356687	5.539667
H	-4.534102	-4.202513	4.121602
C	-1.495839	-0.407573	4.074932
C	-1.327818	0.529304	3.033871
C	-1.857119	0.081770	5.348032
C	-1.508405	1.888791	3.251195
H	-1.040232	0.195479	2.041158
C	-2.042319	1.443635	5.565097
H	-1.985168	-0.606548	6.182351
C	-1.868785	2.352679	4.517749
H	-1.361997	2.587604	2.428184
H	-2.318424	1.799805	6.558492
H	-2.005105	3.421063	4.689291
C	-0.777170	-2.307873	2.629251
H	-0.619515	-3.385026	2.535500
H	-1.129114	-1.828826	1.709430
H	1.523781	-6.660347	-4.370763
C	2.189413	-6.692142	-3.496146
C	3.276372	-5.609877	-3.561151
H	2.661534	-7.684958	-3.474790
H	1.577211	-6.590645	-2.587896
C	2.684081	-4.197426	-3.556674
C	4.087128	-5.795325	-4.860936
C	4.209332	-5.798853	-2.347768
C	1.602129	-4.268859	-0.075998
C	1.308887	-3.947799	-3.651011
C	3.527010	-3.076145	-3.472216
H	4.531012	-6.802052	-4.892802
H	3.444153	-5.672481	-5.744503
H	4.901967	-5.060929	-4.931051
H	3.651628	-5.700805	-1.406625
H	4.660962	-6.801915	-2.378366
H	5.025755	-5.064018	-2.334941
C	2.702521	-4.141022	0.786704

C	0.532555	-5.104485	0.240019
H	1.576036	-3.722135	-1.015466
C	0.794603	-2.650961	-3.645750
H	0.603873	-4.775691	-3.713715
C	3.022538	-1.782290	-3.469134
H	4.607031	-3.211902	-3.390479
C	3.855303	-3.258126	0.505241
C	2.679255	-4.878755	1.982533
C	0.510443	-5.854714	1.425426
H	-0.303635	-5.143519	-0.457322
C	1.640425	-1.535473	-3.549509
H	-0.282466	-2.518875	-3.716220
H	3.714489	-0.946834	-3.367913
C	3.693087	-1.953448	-0.031188
C	5.146374	-3.626191	0.841302
C	1.611918	-5.719432	2.286947
H	3.489551	-4.753807	2.702293
C	-0.630121	-6.827354	1.744634
C	1.140421	-0.143105	-3.586922
O	2.394327	-1.562148	-0.390359
C	4.712147	-1.029936	-0.195086
H	5.329947	-4.631460	1.224988
C	6.230954	-2.721645	0.766930
H	1.639898	-6.268069	3.229042
C	-0.947669	-6.837600	3.251773
C	-0.177517	-8.242008	1.322237
C	-1.917526	-6.468871	0.985014
C	-0.129714	0.249037	-3.071990
C	1.896234	0.860833	-4.176281
P	1.657081	-0.569898	0.635546
C	6.018442	-1.387606	0.281806
C	4.445759	0.291024	-0.821322
C	7.525028	-3.094414	1.217313
H	-0.106512	-7.205149	3.854962
H	-1.207094	-5.830386	3.609249
H	-1.805248	-7.497985	3.446770
H	0.041123	-8.274283	0.245167
H	0.731478	-8.543686	1.862602
H	-0.968215	-8.977641	1.536311
H	-2.248739	-5.441621	1.192114
H	-1.798481	-6.560552	-0.102769
H	-2.724196	-7.153566	1.285077
O	-0.884092	-0.704373	-2.361413
C	-0.679896	1.510962	-3.235818
H	2.866171	0.611026	-4.609043
C	1.463038	2.204308	-4.237278
O	2.620326	0.724517	0.698328
N	0.251540	-0.273356	0.053573
N	1.569528	-1.078406	2.172409
C	7.095790	-0.466658	0.344434
C	5.238042	0.740088	-1.933460
C	3.435994	1.131584	-0.364942
H	7.676616	-4.115922	1.572163
C	8.558441	-2.183402	1.239047
P	-1.070054	-0.461575	-0.771256
C	-2.094893	1.785427	-2.873393
C	0.154526	2.549274	-3.766221
C	2.303028	3.224589	-4.757231

S	2.636937	-1.002619	3.431724
H	0.416442	-1.734685	2.513104
C	8.333187	-0.853906	0.816734
H	6.935295	0.563608	0.030368
C	6.146729	-0.110372	-2.617299
C	5.074026	2.080316	-2.412922
C	3.252540	2.466946	-0.826173
H	9.543992	-2.479351	1.601226
O	-1.696075	1.035487	-0.638424
N	-2.071383	-1.535786	-0.186748
C	-2.566717	1.571352	-1.585763
C	-3.000957	2.302763	-3.858496
C	-0.234687	3.913180	-3.781991
H	3.291862	2.943957	-5.125492
C	1.891588	4.539246	-4.769223
O	3.860459	-1.737846	3.160237
O	1.836027	-1.332154	4.602001
H	9.141225	-0.123087	0.871668
H	6.257549	-1.142093	-2.286352
C	6.873004	0.345787	-3.698024
C	5.854642	2.527172	-3.511955
C	4.107290	2.919183	-1.815031
C	2.193644	3.366583	-0.312900
S	-2.881105	-2.753059	-0.832181
C	-3.845548	2.012963	-1.136282
C	-4.310552	2.710969	-3.443193
C	-2.671538	2.390746	-5.235685
C	0.617689	4.885274	-4.261924
H	-1.210279	4.191846	-3.386290
H	2.549957	5.317292	-5.159034
H	7.554450	-0.331695	-4.214523
C	6.738993	1.680505	-4.143945
H	5.726569	3.556315	-3.854212
H	3.977102	3.932437	-2.198152
C	2.485430	4.687252	0.055599
C	0.852232	2.963980	-0.287239
O	-2.040223	-3.664064	-1.600979
O	-3.707422	-3.311997	0.245299
C	-4.676101	2.591150	-2.083783
C	-4.304564	1.918578	0.268737
C	-5.223965	3.207253	-4.410407
C	-3.584026	2.870126	-6.152342
H	-1.692067	2.053021	-5.570913
H	0.306954	5.930971	-4.246184
H	7.327137	2.032239	-4.992712
C	1.465942	5.577248	0.404435
H	3.524601	5.023139	0.070246
C	-0.156653	3.855370	0.051913
H	0.587486	1.951886	-0.574684
H	-5.672638	2.916014	-1.780243
C	-4.176418	0.756883	1.040526
C	-4.978555	3.010271	0.846314
H	-6.219661	3.508481	-4.078602
C	-4.869322	3.288459	-5.739404
H	-3.315598	2.911238	-7.208791
F	2.276088	5.170623	5.022613
C	0.119263	5.188266	0.391927
H	1.738424	6.595011	0.682880

H	-1.188113	3.501881	0.022849
C	-4.740990	0.668171	2.313108
H	-3.644141	-0.106296	0.645920
C	-5.530922	2.919630	2.119564
H	-5.062856	3.944674	0.287623
H	-5.581274	3.663502	-6.475980
C	1.634463	4.037153	4.631169
C	-1.039812	6.149164	0.675157
C	-5.444048	1.739136	2.877361
H	-4.618317	-0.268251	2.856998
H	-6.049982	3.790215	2.524804
F	0.710917	4.411166	3.698857
C	2.587957	3.004613	4.084429
F	0.945265	3.571042	5.706313
C	-1.941704	5.585400	1.792112
C	-1.874844	6.293189	-0.616276
C	-0.549717	7.538680	1.102833
C	-6.137955	1.643823	4.239432
C	3.833570	3.405661	3.598306
C	2.206997	1.661240	4.048271
H	-2.778845	6.274387	1.981365
H	-2.373689	4.612145	1.521286
H	-1.378469	5.465015	2.727745
H	-2.290525	5.326021	-0.933450
H	-2.713682	6.986809	-0.452501
H	-1.258393	6.683636	-1.439244
H	0.053410	8.018588	0.318344
H	-1.413084	8.189431	1.302915
H	0.052976	7.488685	2.021588
C	-5.700160	2.812593	5.144028
C	-5.814175	0.326611	4.959419
C	-7.662981	1.720689	4.014875
C	4.721879	2.449683	3.104549
H	4.118496	4.455709	3.626767
C	3.103747	0.727356	3.532984
H	1.232288	1.342864	4.418329
H	-5.951903	3.788032	4.705527
H	-6.206108	2.744059	6.118937
H	-4.615393	2.785716	5.315263
H	-6.322516	0.307722	5.934722
H	-6.151461	-0.548503	4.386505
H	-4.734202	0.220909	5.134424
H	-7.949223	2.663723	3.527983
H	-8.004789	0.893652	3.376206
H	-8.193978	1.657579	4.977122
C	4.366259	1.100859	3.076174
C	6.060200	2.875049	2.554235
H	5.050095	0.341152	2.697547
F	6.454294	4.082489	3.038883
F	6.024009	2.990925	1.192011
F	7.037823	1.979156	2.845814
C	-3.638980	-1.930331	-3.345459
C	-4.004343	-1.982161	-2.003410
H	-2.722390	-2.418259	-3.672805
C	-4.453844	-1.243812	-4.248157
C	-5.185765	-1.391215	-1.554059
C	-5.624749	-0.623874	-3.815633
C	-4.053114	-1.215217	-5.701485

C	-5.988752	-0.709317	-2.468729
H	-5.464496	-1.453380	-0.502923
H	-6.243439	-0.069334	-4.518974
F	-4.227821	-2.436786	-6.285115
F	-2.736401	-0.898423	-5.853174
F	-4.774600	-0.321851	-6.422964
C	-7.287044	-0.094623	-2.008447
F	-7.630719	0.991705	-2.757811
F	-8.319422	-0.981592	-2.110184
F	-7.235401	0.306928	-0.714184
H	-3.833588	-2.800378	2.095401
H	-2.961350	-4.383669	3.311881
H	-2.961585	-4.013886	5.883877
H	-1.044108	-2.410751	5.919204

228

TS1-3'

C	-0.20760560643525	-3.35153875511674	-2.89580763102848
C	-0.00031610808882	-4.12841192505947	-4.15641012995329
C	-1.34298592932869	-4.39190380344053	-4.89400943937952
C	-2.21171292642461	-3.15571403356906	-5.09321542078389
O	-2.83434464522066	-2.80131079622505	-3.85780382542032
H	0.65861633108891	-3.54674692555536	-4.81547047495867
H	-1.93736241190664	-5.13506163035865	-4.33983205808451
H	-2.98343726423800	-3.37854870339141	-5.85384521351249
C	-0.61234265597763	-4.03914107512709	-1.66951768564295
C	-0.76943954318949	-3.32896189650073	-0.46114166116145
C	-0.80607974778874	-5.43682523485738	-1.64237968586125
C	-1.10251962680379	-3.98129948779715	0.71911478276729
H	-0.62244770204030	-2.25277066940276	-0.43510941594208
C	-1.14674422337127	-6.08925894241662	-0.46183465889548
H	-0.68590431437798	-6.02275666516861	-2.55257388639068
C	-1.29495802065425	-5.36409016825058	0.72364488500652
H	-1.20976916052936	-3.40030012731918	1.63524352399625
H	-1.29181863852988	-7.17024219498446	-0.46302993134033
H	-1.55232959105407	-5.87999075372892	1.64968253790964
C	0.05296003768513	-1.98622515835953	-2.91321742484749
H	0.34839041816841	-1.53856272568554	-3.86508459923047
H	-0.54744043836972	-1.32720284974178	-2.27423170768933
H	5.33236542099059	4.33950723643367	-2.92995423971369
C	4.68293604528600	4.40430887705419	-3.81345624412377
C	3.48949281131830	5.35421486626322	-3.58863492266009
H	4.33477872087480	3.39054581707565	-4.05228784079922
H	5.29385949800531	4.76657997892146	-4.65407602171349
C	2.66614177595906	4.89239087557585	-2.38234220171047
C	2.65802982534186	5.38410901742876	-4.87902181569841
C	4.01676419865588	6.77616277227185	-3.30356360232357
C	2.22253669032869	1.44292300093240	-3.58499798688411
C	3.28597276365569	4.68042936257319	-1.13921044548063
C	1.28213574582090	4.68360399583642	-2.44214967117947
H	2.25119909596071	4.39029238252650	-5.11750974917840
H	1.82302176227291	6.09662554964383	-4.81396660235688
H	3.29597553995780	5.69695617839282	-5.71823440053216
H	4.65159442090367	6.79419674499705	-2.40636169568883
H	4.61595928407541	7.13758168076931	-4.15323600897454
H	3.18510109422764	7.47756639548837	-3.14356461187113
C	3.45499872424470	0.78694841050837	-3.44635322799802
C	1.41136615637820	1.22202817003058	-4.69749819525076
H	1.88741611154110	2.14789384614231	-2.82769979463835

C	2.56349712745245	4.28143423251416	-0.02144951922351
H	4.36381890356896	4.82008953288422	-1.03720955147044
C	0.55200149798077	4.27537562761758	-1.32656902667849
H	0.74589073235105	4.82323569713563	-3.37982257491766
C	4.35452593560984	0.98896575522081	-2.28997996384175
C	3.83277700064164	-0.10974735076232	-4.46180517195393
C	1.78996658351420	0.34175638146847	-5.72034535082507
H	0.45685421844882	1.74394240656370	-4.73806148247420
C	1.17712723137140	4.06462051952131	-0.08840353259056
H	3.09048802115552	4.11023691582299	0.91613622589516
H	-0.51908195842282	4.12160569209997	-1.43384843877400
C	3.87111178220327	1.07466307150077	-0.95744594588636
C	5.72971868794730	1.01748141780577	-2.45023137574178
C	3.02137637125006	-0.31908415928491	-5.57239553123940
H	4.75573150601207	-0.68084559407044	-4.35244252322166
C	0.93528463277427	0.13089304567611	-6.97462867732834
C	0.41703293355726	3.71024052765271	1.13067477619508
O	2.47785620792570	1.05332448940448	-0.77069607617851
C	4.67967822332622	1.14704840586035	0.16776904145312
H	6.14635866022063	0.99074235853258	-3.45874301438372
C	6.61834128967591	1.01825690275106	-1.35107436895272
H	3.35676811385360	-1.02584108200823	-6.33311959837043
C	-0.48191102062658	0.70044748633512	-6.80899285182498
C	0.81559943124833	-1.36948432476364	-7.30426517814224
C	1.62722860221146	0.84836758787690	-8.15333550570558
C	-0.74982220354306	2.89228010991928	1.10459488274407
C	0.77844977670752	4.22317130850604	2.36685289226204
P	1.83773009439055	-0.31475767304268	-0.20888183744925
C	6.09678285461932	1.04374883459564	-0.01486496549887
C	4.07227687027006	1.29881262104469	1.51527017276267
C	8.02385069710080	0.94324712797836	-1.54086496631621
H	-1.07206454260442	0.48382079630881	-7.71141870134471
H	-1.00642133993942	0.26456517528938	-5.94629291414120
H	-0.47251668887047	1.79122163781657	-6.67613819204701
H	1.79135227147393	-1.83198580319099	-7.50499739682982
H	0.34603379123778	-1.91663648253534	-6.47449619124675
H	0.19024845668591	-1.50858770374851	-8.19861502063620
H	1.71855874607329	1.92550282729618	-7.95222199757143
H	2.63722813546502	0.44899667259926	-8.32454624582419
H	1.04357009603259	0.71786437848992	-9.07763658074996
O	-1.07810271756451	2.26027372084651	-0.10184601071857
C	-1.60658927804652	2.72136514065048	2.18029871595329
H	1.65851604570767	4.86399554144285	2.44124971087378
C	0.03927243037965	3.95659228338106	3.54233634421224
O	2.72728687339345	-0.64030379960698	1.09690720795135
N	0.34488746143500	-0.04258737987848	0.12949370980375
N	2.04742536437123	-1.62060693933248	-1.15383891722999
C	7.00295240538098	0.91419310313380	1.06969584190824
C	4.42302043493492	2.39090138696939	2.37809968182812
C	3.09506297300434	0.41481711243902	1.94893486263158
H	8.41074229535132	0.93829422625320	-2.56201529300557
C	8.88019162420728	0.84719381021888	-0.46574030882052
P	-1.03682490196405	0.64347357328196	-0.18298916613063
C	-2.93608072732953	2.07916316878568	2.00298173618524
C	-1.18651877138542	3.21661079962334	3.45872527132605
C	0.48148574795397	4.42188785719069	4.80923622198913
S	3.33588322693705	-2.62983781493638	-1.34767666855011
H	1.08893996583771	-1.87190913701298	-2.09614217341325

C	8.36113707105937	0.81365555773167	0.84884448951616
H	6.61296988749358	0.87997702460577	2.08670278648236
C	5.32303906032204	3.41991525560787	1.99292569437138
C	3.79423013491300	2.48627910540196	3.66378657623971
C	2.44429714021060	0.49288601907199	3.21010709430054
H	9.95690438879324	0.77646788157508	-0.62630536741890
O	-1.90891158198370	0.12254793634138	1.08526644977898
N	-1.71790011020646	0.22182975101533	-1.55253348625361
C	-3.06795245479978	0.79781886256588	1.47952069206059
C	-4.12210751035825	2.80243746176567	2.36579208730497
C	-1.89999297118484	2.95108224667540	4.65657269165351
H	1.41178547661707	4.99153718737541	4.85461216284901
C	-0.23334186888534	4.14546685603947	5.95451714135886
O	4.57741849575203	-1.91181759657697	-1.57850519335047
O	2.87741798858468	-3.62770662923419	-2.30376372632477
H	9.03898096758209	0.70039482609514	1.69608973765493
H	5.78109607257366	3.38114667021615	1.00587160187250
C	5.60933339665541	4.46950954499839	2.84128779658470
C	4.12835356289272	3.56770982816741	4.52215890610193
C	2.83049584158921	1.52653302236145	4.04664219639922
C	1.37435344699291	-0.44222918734963	3.62063570638030
S	-2.31422004782702	1.06507315111400	-2.77192211216328
C	-4.31144804839414	0.09670753397976	1.43652555909184
C	-5.38261955373528	2.12432541006735	2.32020585279047
C	-4.11022255469310	4.17622337411974	2.72202290085158
C	-1.43010837666643	3.39630538872631	5.87513970843966
H	-2.81957813585939	2.36908353070224	4.60642540066056
H	0.12215972248314	4.49956212368018	6.92331036043206
H	6.29696616262960	5.25223610400916	2.51779255929393
C	5.01761452518980	4.54133864943515	4.12340780708838
H	3.65221738024635	3.61470738195839	5.50364442389559
H	2.36425920000280	1.61464330253243	5.02945141849107
C	1.45974327892707	-1.82911964772758	3.44342142668542
C	0.22725254712904	0.06708834594734	4.25042085893366
O	-1.43594911010608	2.13788458597752	-3.22643331740159
O	-2.77274096538805	0.08378188182071	-3.76459537399535
C	-5.42951624884855	0.78125267833048	1.88916865998577
C	-4.48927532690927	-1.29323478319544	0.95238988436902
C	-6.56663435169401	2.82698322548975	2.66818301908283
C	-5.27866687828182	4.83534462718436	3.04312982307322
H	-3.16698334980231	4.71991383669667	2.71798524601863
H	-1.98548367729992	3.16572444358447	6.78539352546089
H	5.26118995469087	5.37061672049585	4.78911104131732
C	0.44253269471521	-2.67205414144753	3.89270494198171
H	2.33798981716626	-2.26257174908037	2.96876872838359
C	-0.78979322219227	-0.77642326660921	4.67803672361481
H	0.12075078320098	1.14322076222675	4.38238217567368
H	-6.39943533590633	0.28335947430671	1.84680961872991
C	-3.92466447316947	-1.76967354119365	-0.23673233947246
C	-5.35376476760096	-2.15863132269394	1.64862994222671
H	-7.51872968926943	2.29448493565970	2.62409450905432
C	-6.51799310934551	4.15609514036047	3.02741611070323
H	-5.24665088352507	5.89634205504002	3.29417679805759
F	2.91816732828724	-7.31382595710867	2.86097544740681
C	-0.70712972188976	-2.16908591139080	4.51690707816289
H	0.56201525290525	-3.74497835000156	3.74938569655853
H	-1.67060858811856	-0.33138569156016	5.14465089496170
C	-4.23834690251063	-3.03593007182371	-0.73274154214953

H	-3.24914011525560	-1.13948489843979	-0.81055420134857
C	-5.65160043595958	-3.42455157853089	1.15809383465719
H	-5.78476893270719	-1.83608254388178	2.59818996786772
H	-7.43395781950836	4.69033389361416	3.28367506748543
C	2.17185047025492	-6.41484788243242	2.16453047448367
C	-1.84779897490441	-3.06293708299532	5.01162370222679
C	-5.11489608567461	-3.88995891808902	-0.05508945194333
H	-3.78614704059326	-3.33736279909365	-1.67746145148939
H	-6.32489472114974	-4.06143826825597	1.73521736257238
F	1.11250764002366	-6.08747374664179	2.96789314463970
C	2.97788642169602	-5.19824950821386	1.78222324279116
F	1.66543225211910	-7.05697915160513	1.08374232579516
C	-3.13629288847062	-2.70035035783097	4.24740321029314
C	-2.06272308773461	-2.82804269638920	6.52071281105099
C	-1.55693025359633	-4.55394848674859	4.79368108139745
C	-5.50754622301894	-5.26861856102216	-0.59275327563814
C	3.94743499422372	-4.72510431929251	2.67233060911762
C	2.75208733266743	-4.55434075736045	0.56400554562314
H	-3.97746782682816	-3.31503034969905	4.60274721046476
H	-3.40571289160867	-1.64366009154255	4.38430202956802
H	-3.02429492282046	-2.87467377688280	3.16882986740224
H	-2.88431524271927	-3.46089327710127	6.88901978804237
H	-1.15458644388731	-3.07614294038550	7.08895525727379
H	-2.32076376620435	-1.78201575358490	6.73550362649956
H	-2.40112752247896	-5.15128441562398	5.16703201105826
H	-1.42666633208018	-4.79266078227346	3.72896929203425
H	-0.65255699414377	-4.87784819880799	5.32876315504510
C	-5.13220327722224	-6.35109495773440	0.43952213081753
C	-4.80144132768470	-5.59272978118498	-1.91710420376572
C	-7.03145687908091	-5.29675346521850	-0.83085166535113
C	4.71668554092711	-3.61524156951009	2.32516898248549
H	4.11537653588638	-5.23221827516125	3.62122147228426
C	3.53647470969875	-3.44763082437545	0.23797021804485
H	2.00023899164240	-4.91965902505817	-0.13503024536868
H	-5.64065280816282	-6.19095236757879	1.40051404663475
H	-5.41947721629663	-7.34639759959828	0.06750835962026
H	-4.04869199265359	-6.35056075621120	0.62392269582877
H	-5.04775917976019	-4.86327836929931	-2.70161923083422
H	-3.70898916349946	-5.60519057827765	-1.79873144565886
H	-5.11734719836918	-6.58687772126699	-2.26624623523402
H	-7.58975561011245	-5.10538101373288	0.09625042390362
H	-7.32450752255895	-4.53269754825047	-1.56513996557122
H	-7.33828608517813	-6.28146750528199	-1.21526986410299
C	4.52681557735700	-2.97899145708761	1.09698681520636
C	5.74424642797927	-3.05376299608661	3.27683390214483
H	5.14100295833451	-2.12934925549499	0.79967822242257
F	5.31894305634551	-1.87301902002372	3.81488354495096
F	6.92288465656961	-2.79649916190413	2.65041000369757
F	6.00506616668258	-3.89357818479989	4.31099111888479
C	-3.72738621271127	3.21116259003259	-1.78622881772796
C	-3.78935521238573	1.86384473995480	-2.12828884517987
H	-2.80363401712553	3.76444555115160	-1.94755790368566
C	-4.85668299399925	3.83135449758294	-1.24636180013158
C	-4.96446182833948	1.13026755613711	-1.95986966824294
C	-6.03448937605844	3.11232119393865	-1.05258497659932
C	-4.78594094018565	5.30277447759871	-0.92471006756999
C	-6.08177699019594	1.76290242458772	-1.41552156634657
H	-5.00164651535965	0.07898497429047	-2.24271759477862

H	-6.90543042126715	3.59489510354884	-0.61259727351516
F	-5.83307935807425	5.72268049550941	-0.17140328113097
F	-4.78630475016952	6.05512965441811	-2.06415764092013
F	-3.64347042956087	5.61839523682542	-0.25321521489985
C	-7.37246364650151	0.99769632478814	-1.26233013468809
F	-7.16537762274093	-0.32968402018802	-1.07813284153515
F	-8.10704889338550	1.44519767191308	-0.20326037123441
F	-8.16229123837756	1.12947570830377	-2.36741511073624
H	-2.84620714013321	-1.82015208872577	-3.79054992331479
H	-1.60882006370160	-2.31525297446784	-5.47857201961706
H	-1.10440122297129	-4.83223959736508	-5.87498178493199
H	0.50041953683885	-5.08730206539019	-3.96191946818667

228
TS1-4'

C	-2.89984679279618	-3.26936275022447	-0.10106099218057
C	-2.83103304066087	-3.05934062890925	1.38076278639125
C	-1.51407105009976	-3.46947570538706	2.04384032474766
C	-1.32978244711739	-4.98469288800443	2.03656205947164
O	-1.40666703574306	-5.54212430082104	0.72584525562789
H	-2.99835211334740	-1.98020476820532	1.56094341900761
H	-1.51358385701041	-3.11233235704965	3.08446394745465
H	-2.13669527552709	-5.46079958623052	2.61888117501197
C	-4.13425427534634	-3.81623836064375	-0.66861958934271
C	-4.08319536732878	-4.60841404903238	-1.82940606909150
C	-5.38590609250381	-3.55676750319101	-0.07886100052229
C	-5.24776588635365	-5.12552213686798	-2.38801914030050
H	-3.11166125324135	-4.85411029846030	-2.25780504370698
C	-6.55150053922858	-4.05269014354448	-0.65471485819484
H	-5.44716162644112	-2.92128543431557	0.80521115348399
C	-6.48639096233078	-4.84183026081287	-1.80653858707629
H	-5.18937187226298	-5.75565111071017	-3.27676044195941
H	-7.51802206336595	-3.82153761099280	-0.20460757233515
H	-7.40143068018639	-5.23997523931067	-2.24796223558843
C	-1.91031511969424	-2.76441085307626	-0.93333019313532
H	-0.88249645633345	-2.67990793706403	-0.56562455048872
H	-2.00013542924448	-2.91366637767263	-2.01102398295171
H	-2.06487667486690	1.19864681731145	7.83849276059536
C	-1.51312279001547	1.31684149337586	6.89387154685143
C	-0.22103273651428	0.47645486394371	6.93621236113762
H	-1.30255526714046	2.38721319417982	6.76565954020677
H	-2.17272831680207	1.00009389271757	6.07494529216302
C	0.58869684440248	0.65156903171014	5.64672059352618
C	-0.60143133459279	-0.98892235725806	7.19085788140021
C	0.65253546187408	0.96435731911097	8.11293846393522
C	-3.06230214610713	-0.60007569078087	4.04816897549322
C	1.13308371929371	-0.42283122482268	4.92926228360900
C	0.88120119821259	1.94026209551468	5.17145350406815
H	0.28522422077140	-1.62491348402286	7.32352893011634
H	-1.20099668466636	-1.05932839964774	8.10989772713544
H	-1.20231049154516	-1.40044876188077	6.36811016473822
H	0.10820505993368	0.85405232712173	9.06342692729306
H	1.58300965454165	0.38207601442846	8.17677968565417
H	0.92370597701779	2.02278035076078	7.99364341016684
C	-3.98889464085116	0.25062422371141	3.43075763305230
C	-3.45031154823233	-1.85534926820764	4.51985616389694
H	-2.02899184009363	-0.27616535077522	4.17742467463870
C	1.95843936948403	-0.23155982812890	3.81831513895882
H	0.93124378589661	-1.44772287264867	5.24014723667443

C	1.69884076593772	2.14044565336144	4.06814156906128
H	0.46794013679052	2.81710340923391	5.67243944484474
C	-3.65723067844851	1.64351989020751	3.05696227941908
C	-5.29816303045732	-0.22829034812438	3.25147424145567
C	-4.75736281520498	-2.33245363374207	4.36499470471170
H	-2.70487084005818	-2.47238372902770	5.01992236009170
C	2.27657885509739	1.06180451364138	3.37252559385056
H	2.35801531999772	-1.10809983556040	3.31236346454041
H	1.87847296201532	3.15935352252755	3.72781007239521
C	-2.47258796782029	1.99695690541307	2.35262176635868
C	-4.51580060396479	2.67096347482549	3.40154622255728
C	-5.66783912460967	-1.49054597943704	3.70338493632604
H	-6.02191148291827	0.39413420110435	2.72402403970742
C	-5.21480791547905	-3.69141314054440	4.90592866579196
C	3.20050574582448	1.36431020726188	2.25298073540178
O	-1.63041730907288	0.95156495710353	1.97184244591139
C	-2.10191700374434	3.30547261823017	2.08154577284132
H	-5.42407334385659	2.43547455385760	3.95953227943349
C	-4.26563883299503	4.01624450772859	3.04495578200473
H	-6.69512378158389	-1.82246941132746	3.54097451082895
C	-5.86255954491868	-4.52481922472287	3.78184469196118
C	-6.25699370498308	-3.44870045472685	6.01820271103885
C	-4.05200091494250	-4.50238573822913	5.49481355179073
C	3.32554477072031	0.56348483261578	1.08039760180914
C	3.98165428284694	2.50974837807753	2.29939447284327
P	-1.22406615463474	0.63940078719031	0.43975974503965
C	-3.04612894638408	4.35081251257435	2.36919798203959
C	-0.77825969170938	3.64339450132099	1.49647959059173
C	-5.20163471236582	5.04208890147096	3.33900396596212
H	-5.13977420934008	-4.75327965476474	2.98560954590092
H	-6.22958167558733	-5.47980553099037	4.18630084685697
H	-6.71616276656526	-4.00602863457508	3.32452592022087
H	-6.60647891894704	-4.40805226265782	6.42899329715433
H	-5.82225457345570	-2.85918813911796	6.83823666760056
H	-7.13102435058233	-2.90289998679886	5.63617122712553
H	-4.42423541450591	-5.47297378016419	5.85240382914767
H	-3.27261764324498	-4.70036342671790	4.74424536629772
H	-3.58660210392326	-3.99042928127741	6.34952530523490
O	2.62905176058175	-0.64482888302236	1.04847648007189
C	4.13209056898652	0.88816202511658	-0.00670077109539
H	3.95743926039379	3.13356843526178	3.19371561979892
C	4.78999442360072	2.92557355354104	1.22012909736443
O	-1.14557266310098	2.08557227692350	-0.28516818217781
N	0.07058035782901	-0.19597096591758	0.61170918221612
N	-2.35555271900785	-0.12584391597271	-0.45601627885520
C	-2.84526796127578	5.69884859327588	1.97325369175439
C	0.10560537523332	4.55861805479982	2.15892696830562
C	-0.36988425417418	3.10825935895864	0.28596530165703
H	-6.12078326326858	4.77216573594190	3.86320650899557
C	-4.96710810296802	6.34511221981332	2.95716699315107
P	1.37453306080919	-0.86418321481571	0.04522695546972
C	4.18959732651010	0.00014261777356	-1.19937891294981
C	4.85503126098290	2.12761660580387	0.03120351143403
C	5.50789750105125	4.15065990620497	1.27786814545222
S	-3.87740631478545	0.39975296793721	-0.81568981084991
H	-2.19319194079258	-1.44059753664404	-0.72831305469049
C	-3.78387560971295	6.66934765122745	2.25686992386458
H	-1.94662276970233	5.96318623952139	1.41861429905158

C	-0.13429750663722	5.04146115202690	3.47154997834952
C	1.31266857686964	4.95464965650710	1.49453723396344
C	0.75998148922869	3.56708482927285	-0.45082255129467
H	-5.69829103550602	7.12337526890946	3.18003580357541
O	1.79735645240717	-0.00280774876759	-1.25738558996611
N	1.29231464859050	-2.40736242662765	-0.31195789133566
C	3.01633678271689	-0.39670742330234	-1.82778236826915
C	5.43197723315331	-0.47733533934756	-1.73483657510529
C	5.59176565074349	2.63350874568517	-1.07342938764170
H	5.46061511163679	4.73417241210677	2.19975021303085
C	6.22853218799649	4.60270444758382	0.19459798014419
O	-4.52065033516894	0.99957794388371	0.34348815969583
O	-4.50274579796576	-0.70942002233916	-1.51796957328604
H	-3.61375144088342	7.69505040444351	1.92674537875607
H	-1.03504756963368	4.72519969920182	3.99566922533594
C	0.77049843449508	5.87497398323369	4.09602829086923
C	2.21449060275461	5.82842074461086	2.15802754056228
C	1.57817871224982	4.48536603283527	0.18778237048564
C	1.03383701820692	3.16518900841846	-1.84857351845470
S	1.89949050708782	-3.64442224702130	0.49982848070862
C	2.96401281624855	-1.20015104630893	-2.99972462085350
C	5.41310700626896	-1.23927876798991	-2.95075802771811
C	6.67922258479090	-0.26642301606608	-1.09268448242430
C	6.25611458851722	3.84017721996791	-0.99584102650171
H	5.61899062849972	2.06182633081915	-1.99963155996207
H	6.76876357448545	5.54898799288198	0.24774937500179
H	0.57432196683516	6.22096199299716	5.11195013484117
C	1.95373121250896	6.27795406071919	3.43439260370469
H	3.12763606399436	6.12298624603039	1.63703337641636
H	2.45297710258357	4.87308480179384	-0.33673609622518
C	0.01130994044316	2.98413101564463	-2.79149032168126
C	2.35683897987030	3.04412659403525	-2.30203607674654
O	1.88691112815283	-3.45694572806486	1.95018082527276
O	1.27660099598518	-4.85421216746273	-0.04911565407887
C	4.17791791679586	-1.56682734848728	-3.55571767608459
C	1.68817571463146	-1.66068369691598	-3.59052203394769
C	6.64026018013683	-1.68899605512794	-3.50600632812206
C	7.85247166376878	-0.73489415867803	-1.64755647795820
H	6.70311304678024	0.26020167340925	-0.14016027426357
H	6.80603375847975	4.20928250864784	-1.86277010829762
H	2.65852898691664	6.94105835596341	3.93819666488098
C	0.30504520858613	2.69449116623182	-4.12411763125893
H	-1.02848752739256	3.08986878514282	-2.49080390046833
C	2.64129406600503	2.74010908143233	-3.62745912219955
H	3.17839574650133	3.16585500958663	-1.59835197481110
H	4.18683470683755	-2.14864316153475	-4.47866831673676
C	1.58281291506649	-2.97031102958512	-4.08389342706730
C	0.55938268522281	-0.83356552387484	-3.70265682672190
H	6.61120464652131	-2.26323578603918	-4.43392354755177
C	7.83824945067963	-1.43837787020912	-2.87285991556529
H	8.79660224008667	-0.57370635368235	-1.12581678468509
F	-2.68089282246848	1.04372711707675	-6.01234964782335
C	1.62346715759458	2.55414760581045	-4.57718569841987
H	-0.52188101831035	2.56919927723040	-4.82109322069874
H	3.68709718334458	2.63837712898725	-3.92333186903997
C	0.40349551820337	-3.43119390058597	-4.66469518070893
H	2.43210883529968	-3.64905848924908	-3.98902694986094
C	-0.61125518209328	-1.29870739284053	-4.29577248512306

H	0.59795239834327	0.19274289145921	-3.34535127638013
H	8.77266700759964	-1.80077520328762	-3.30368184642834
C	-3.41785342078512	2.16140536823649	-5.75625251729460
C	1.97383599063501	2.23765061731595	-6.03396502793720
C	-0.72569891778170	-2.60724394075718	-4.78997007412053
H	0.37130965514140	-4.46124734023799	-5.01906451032993
H	-1.45121060577166	-0.61016671295212	-4.37806133335943
F	-4.66035298035098	1.93817462660901	-6.26525991150856
C	-3.47289521973248	2.47158043303420	-4.28078903281671
F	-2.86856139890983	3.17744400124807	-6.47265143018751
C	2.70739927833879	3.45390206461572	-6.63678912553079
C	0.72635731965339	1.94480428496152	-6.87916319528365
C	2.89505803224626	1.00258961010458	-6.09803729399660
C	-1.99510066716871	-3.08359428430107	-5.50524231540255
C	-3.49092136730175	3.80095956542178	-3.85304767872983
C	-3.56482629436558	1.42738557925368	-3.35808339684858
H	2.97706111844904	3.25393902009664	-7.68492740353545
H	2.06934373070933	4.34912049757826	-6.60893185361300
H	3.63071496326172	3.67931011363406	-6.08470678644015
H	1.02995577195935	1.70472913258231	-7.90828338659005
H	0.16569487175919	1.08445292895120	-6.48579993844572
H	0.04759556869154	2.80847230631061	-6.92761146735104
H	3.81980939679430	1.14616878394198	-5.52277911540771
H	2.38483749669011	0.11436322954623	-5.70169863500388
H	3.17643179973162	0.80152221477600	-7.14267544506052
C	-1.86120285670476	-2.71277081800165	-6.99885101030735
C	-3.25718896908249	-2.40488424375901	-4.94164764718392
C	-2.17383583523922	-4.60721561958690	-5.38672460641173
C	-3.61734340131311	4.08408291668849	-2.49257679287260
H	-3.41108672143856	4.61104532762582	-4.57563323198099
C	-3.68313724212323	1.73255100151208	-2.00392286613488
H	-3.57083551476444	0.38594333853342	-3.67927894881659
H	-2.75324292780458	-3.03781073879053	-7.55634354437035
H	-1.75461744522116	-1.62563090281687	-7.12365659782714
H	-0.97930771369746	-3.19470117466935	-7.44483102169556
H	-4.15113527563543	-2.83110015901799	-5.41943354664430
H	-3.35886688590145	-2.54753763301627	-3.85685678583426
H	-3.26584031423635	-1.32770860908756	-5.15291954718582
H	-1.38027425359313	-5.16040813869962	-5.90728594810726
H	-2.17995963108209	-4.93041338662194	-4.33612871166290
H	-3.13009401121011	-4.90328082544085	-5.84176886036477
C	-3.72419524969348	3.05207775167064	-1.55784952014353
C	-3.59466278269286	5.51201514702710	-2.00399640298193
H	-3.84266508573732	3.26402818499353	-0.49539495429464
F	-4.54788911035492	5.73711175872655	-1.06480699019662
F	-3.79210763955483	6.40316009266380	-3.00984695337979
F	-2.39131860185041	5.81422325669275	-1.43343257435882
C	3.97987315150370	-4.37565407803772	-1.16708327090543
C	3.62932093003734	-3.74546100331800	0.02512069983765
H	3.20089747655513	-4.78913692318318	-1.80733645580150
C	5.33036773132266	-4.49137192668925	-1.50196198850015
C	4.60203029893063	-3.23540913866184	0.88120984045669
C	6.32052809419683	-3.98388863569630	-0.65860188406423
C	5.70245608685694	-5.22771764328846	-2.76292621885586
C	5.94845090268669	-3.35626234970256	0.53043332227271
H	4.30201390773504	-2.76566278453268	1.81688485408539
H	7.37046992112715	-4.07179801459675	-0.92868201629321
F	4.95933533346613	-4.81169352290036	-3.83285112092338

```
  F   5.48227263628959     -6.56712904388417     -2.64144810162695
  F   7.00833587200003     -5.06738517849061     -3.09809617926782
  C   6.99070448185101     -2.85367013896795      1.49666418313221
  F   7.07887289726663     -3.66174225578029      2.59237577302313
  F   8.23154608779861     -2.79721119377889      0.94604303518195
  F   6.69370891398089     -1.60619388474261      1.95915186980393
  H  -0.54469200832881     -5.33868012789256      0.29688437913980
  H  -0.36877024924623     -5.23777386162379      2.51752851465072
  H  -0.66352036819361     -2.97466523812833      1.55832858940378
  H  -3.66306020941396     -3.57944491004520      1.87111568522219
228
TS1-5'
  H  -4.39739197088266     -1.99533435497703     -5.54013215167051
  C  -3.59684727492250     -2.08533846693109     -4.79015470255820
  C  -2.25168857750894     -2.37666909071916     -5.48504695035174
  H  -3.87397519507678     -2.88842973210884     -4.09415904575250
  H  -3.55301942473629     -1.14567586668308     -4.22418500029060
  C  -1.15126855409882     -2.55595459306040     -4.43350368715657
  C  -1.95125174970561     -1.22742250720052     -6.45714205157274
  C  -2.37491306765041     -3.68434014101748     -6.29517457191776
  C  -1.98044989921662      1.05101487252772     -3.25879772921047
  C  -1.27926545412782     -3.55345585777011     -3.45316068952415
  C   0.01761302903689     -1.78332241564761     -4.40592252813799
  H  -1.87980312497959     -0.26283904203975     -5.93514128944988
  H  -1.01659414382363     -1.39592383250393     -7.01196196157198
  H  -2.76474496842610     -1.14776890667818     -7.19290104489665
  H  -2.63397425872805     -4.53435686799629     -5.64855528999147
  H  -3.16127980012758     -3.58469588860646     -7.05891862435728
  H  -1.42850140234342     -3.92118580317328     -6.80248907364161
  C  -3.24595501120439      1.33137148387061     -2.70849215578273
  C  -1.39441554137025      1.90417862364601     -4.18576707256842
  H  -1.45232542296075      0.13891638089540     -2.99419972597972
  C  -0.28039077970792     -3.78521826468734     -2.51520124684210
  H  -2.17964361047580     -4.16748498326005     -3.41204240604661
  C   1.02223647928800     -2.00612060916679     -3.46331864219041
  H   0.16589890615204     -0.98945706193145     -5.13745442560929
  C  -3.97788312137360      0.34160304052252     -1.88189506409544
  C  -3.85742221391880      2.53601293624961     -3.09931881250192
  C  -2.02900250574455      3.07405535083674     -4.62868446169942
  H  -0.41271305628847      1.63218577243142     -4.57443334273571
  C   0.90209644751753     -3.02723052973622     -2.50984604068050
  H  -0.42583809127789     -4.55621290948188     -1.75769989035668
  H   1.91542742358290     -1.38444651797104     -3.48790771025057
  C  -3.32126185544684     -0.64289083039902     -1.08314815367192
  C  -5.35835610587256      0.24403705550843     -1.97325426134694
  C  -3.26584317687111      3.38142141926130     -4.04084462792062
  H  -4.82104105719812      2.81623240139898     -2.66928877755507
  C  -1.40438183211477      3.89938087886582     -5.75769998761197
  C   1.98537806942747     -3.37413703830471     -1.56322540766052
  O  -1.94103335433114     -0.52176863283768     -0.92901708336985
  C  -3.96741651648042     -1.75465754663555     -0.55018156573185
  H  -5.90153109942282      0.94437266568300     -2.60863441739271
  C  -6.09628874732465     -0.75208165189904     -1.29580936154893
  H  -3.79170815672490      4.29484690900903     -4.31907188813534
  C  -1.49229226068557      3.05991696627817     -7.05217092145384
  C   0.07765917063291      4.20314452170781     -5.46234574764119
  C  -2.14109472295077      5.22615866474237     -5.98816124241000
  C   2.74566682202809     -2.39507268326145     -0.86618151017583
```

C	2.30347487165664	-4.70089075125499	-1.32140739394218
P	-1.26560871075547	-0.34956822364916	0.53566326345505
C	-5.39972499956283	-1.77957811719535	-0.57932892549780
C	-3.18311324087995	-2.91069170358993	-0.03802303708341
C	-7.51542491909286	-0.77716593095218	-1.33678898621424
H	-1.06160844266862	3.61848684182751	-7.89700919176450
H	-0.94109588895110	2.11458243474210	-6.95073566951360
H	-2.53754781906413	2.81777758305579	-7.29322932046761
H	0.66644372787055	3.29115371542049	-5.29937244354056
H	0.52176654357958	4.74350802034841	-6.31141554569741
H	0.18707340689989	4.83000198552074	-4.56772610705485
H	-2.13839130498525	5.85799315470435	-5.08742186147644
H	-1.64319668456940	5.78848958730898	-6.79085397701234
H	-3.18526681660042	5.06781803158155	-6.29464964298178
O	2.42953695487433	-1.05248063297001	-1.09257953382951
C	3.78745602392769	-2.69355934752245	0.00313667059831
H	1.75981585985552	-5.48148131439761	-1.85718595100879
C	3.31106239187666	-5.08190184590402	-0.40555783629807
O	-2.04124688139093	-1.43537956569964	1.43698286742401
N	0.22399719953688	-0.70765530086210	0.29912451745044
N	-1.68026332309239	1.07592672365145	1.21935134233495
C	-6.15928897186295	-2.76574572850128	0.10040019803699
C	-3.35051625392346	-4.22605688946046	-0.59056252550036
C	-2.20068799890533	-2.72764802349487	0.92023376417602
H	-8.03483872883208	0.00760302922532	-1.89000370313899
C	-8.22454958018617	-1.75568473671582	-0.67417011271110
P	1.69605432630513	-0.20374378074372	0.08382270789153
C	4.53679626386376	-1.60025800635257	0.67656712616718
C	4.06882359865662	-4.07268346444649	0.27699916758158
C	3.57030958962662	-6.45107681414128	-0.12941038488056
S	-2.39592007811078	1.22340329431862	2.70381678861500
H	-1.00155464143120	2.29851401204699	0.93048002416134
C	-7.53816505357286	-2.75172208321830	0.05727775572962
H	-5.63978802251452	-3.53022687805476	0.67657443291262
C	-4.24663693275999	-4.51875483877974	-1.65185658274793
C	-2.53091849654141	-5.29070032038195	-0.08424813352046
C	-1.35310949559684	-3.75328477808857	1.42217300900263
H	-9.31504864641019	-1.75816483285454	-0.70427939165031
O	2.44907412668607	-0.72005880923499	1.41951522442330
N	1.99225870352068	1.33698448102986	-0.15964266592460
C	3.84465578752564	-0.63931997928027	1.39961203270357
C	5.96234930350220	-1.48674070623108	0.58996035622680
C	5.02665381587999	-4.48929565174538	1.23894499238824
H	2.99203769363466	-7.20698457397871	-0.66515095242003
C	4.51555080710861	-6.82086103153188	0.80198749058955
O	-2.37527036170438	2.64810698134995	3.00548778683428
O	-1.87310961600347	0.28686321773060	3.68609384667841
H	-8.10256807200468	-3.50767180873835	0.60439382949416
H	-4.85067858130742	-3.71853952423447	-2.07465000889486
C	-4.35451196763349	-5.79562500182628	-2.16403473813852
C	-2.67865273300086	-6.59693202248488	-0.62322177631906
C	-1.56262240041220	-5.02428555263158	0.91192179421320
C	-0.28077824825753	-3.49355976551976	2.40726834853681
S	2.46486844391036	2.06368323114109	-1.49858306993359
C	4.46269919447777	0.41669944189987	2.12571274221655
C	6.61786602785898	-0.46196351367096	1.35100481638420
C	6.75008321097026	-2.31242115387663	-0.25266075885811
C	5.24228794702399	-5.82741966552468	1.49711135619379

H	5.59147715586179	-3.73623453997793	1.78589597884667
H	4.69933224474361	-7.87573683152194	1.01041339230685
H	-5.04609172000409	-5.98961615165389	-2.98535079462209
C	-3.57218224994958	-6.84961675835479	-1.64070283612761
H	-2.05449072051649	-7.39714764777988	-0.21986558281690
H	-0.95552086644616	-5.85069194531836	1.28653464107663
C	-0.45587324621776	-2.65102669655679	3.51347722714025
C	0.96683734824836	-4.12284298929228	2.25616590601649
O	1.77315500112247	1.59817107299468	-2.70073179301218
O	2.45423265907678	3.50550678240563	-1.22027776750318
C	5.84845529266372	0.44600933412334	2.11443092693562
C	3.68121350561204	1.47228844465530	2.80764280549323
C	8.03205337466880	-0.35021316756405	1.28491128432313
C	8.12030311284767	-2.16126396644291	-0.31290849671026
H	6.25772615427750	-3.06111540244083	-0.87168161529172
H	5.97770700091569	-6.11981522308944	2.24816686727152
H	-3.67036448536959	-7.85613768133390	-2.04957764272876
C	0.57273899149680	-2.45615631440523	4.43507012366296
H	-1.39872271427324	-2.13104843955613	3.66615957457020
C	1.98434274927991	-3.92769022951961	3.18224211872773
H	1.15188584384205	-4.74882160731282	1.38201371904370
H	6.36924054229191	1.21419782863014	2.68789566831024
C	2.47738673942663	1.23000684130437	3.48833175340021
C	4.14397272983137	2.79911696139288	2.76129199632237
H	8.52000271810540	0.43108203537781	1.87074687430763
C	8.77098461518042	-1.18224576016271	0.47196494407638
H	8.70371616397317	-2.79527969690671	-0.98191210361419
F	-6.62570605933183	-1.54226413754034	4.96857183583471
C	1.81310415985068	-3.09223412273833	4.29877135373076
H	0.38479328689429	-1.79067198085781	5.27697922582622
H	2.93762441671994	-4.43136579439207	3.01692367550728
C	1.76303564040377	2.27415071384312	4.07791448847691
H	2.07998519931172	0.21840515894834	3.55635574623546
C	3.43047083613117	3.83199245985728	3.35643884330270
H	5.06409235736451	3.02753092819238	2.22126518198550
H	9.85530976572832	-1.07489446444171	0.41875913780325
C	-6.62035100256780	-1.75182136096274	3.62093770266066
C	2.94885022853554	-2.93004744663476	5.31440807161888
C	2.21594920036227	3.59967320099485	4.02258047992250
H	0.82472448388215	2.03400808114792	4.57822335006127
H	3.82462324347257	4.84664477280476	3.28199527962378
F	-7.90228976879999	-2.00531302779532	3.25298901706063
C	-6.02988914707000	-0.57017700140357	2.89248590921714
F	-5.90113182654440	-2.88902208999738	3.40814070758438
C	4.20864545701085	-2.38850482140265	4.61134684751077
C	3.26508778444498	-4.31162888963979	5.92475235927738
C	2.58137837542641	-1.96523920244439	6.45017013581134
C	1.46376182190369	4.76691753703320	4.66872005571553
C	-6.83778101656108	0.28724434319817	2.14725816274496
C	-4.66226914916043	-0.31772840884090	3.03255950281644
H	5.04381546888998	-2.32874693534800	5.32564034620070
H	4.51873911038749	-3.03457312125663	3.77933409391346
H	4.03471761708784	-1.38202009722664	4.20879826629645
H	2.38198961319949	-4.72768729651053	6.43094685076306
H	3.58161538165471	-5.02751178081337	5.15325726537825
H	4.07756930928950	-4.22400139288736	6.66216356693917
H	1.71067799502816	-2.31873906652072	7.02137521515380
H	3.42705967674462	-1.88114562418715	7.14796683202986

H	2.36172735508300	-0.95633814461050	6.07112668686856
C	1.31964887654904	5.92307193756741	3.65700172605401
C	2.27880279992629	5.26413405074194	5.88163555200159
C	0.06645653512647	4.35717597267736	5.15681304455737
C	-6.26862079397453	1.40819054597134	1.53642760743538
H	-7.90006594588430	0.08117421435734	2.03557822797033
C	-4.11826154485749	0.80614913439110	2.42067201891151
H	-4.02197938856679	-0.97263473870904	3.62251117694694
H	0.84629567978548	5.59060811132501	2.72253982771776
H	0.70902781417989	6.72954783011915	4.09031354000472
H	2.29218980425984	6.35636310045423	3.38758772376085
H	1.77288411590263	6.11865311672619	6.35686592436957
H	2.38816399134236	4.46727569086005	6.63136109396170
H	3.28534175028304	5.58693317175023	5.58024356294848
H	-0.44718918883469	5.23527837427802	5.57528702014443
H	-0.56180277848793	3.94985084382229	4.35188782077180
H	0.11970375915003	3.59945151386272	5.95174628066021
C	-4.90672137789216	1.67823216530551	1.66965505195497
C	-7.13564515405375	2.39007809005303	0.78981281487387
H	-4.46557392042784	2.56716944755988	1.21781458466479
F	-8.35081373053039	1.87773738406332	0.47034809454290
F	-6.55428042631675	2.80543253506376	-0.37393870194248
F	-7.36119950036301	3.51496471156566	1.52960654904798
C	5.17228737495081	2.37811207678207	-1.03665390862722
C	4.20060550958936	1.64189154904802	-1.71190558110478
H	4.87108065730874	3.20287726503098	-0.39110951118411
C	6.51885037805015	2.05718062508625	-1.22208871755626
C	4.55609436837956	0.60218537182338	-2.56684842016909
C	6.89291630698624	1.00352649436709	-2.05811820370675
C	7.56618701038813	2.90995477838202	-0.55289715288004
C	5.90511747533580	0.27999878921456	-2.72628169703137
H	3.78095654063121	0.05861350059552	-3.10365452265110
H	7.94244734135974	0.74617172228847	-2.18253370570284
F	7.63768936206467	4.14547665199628	-1.12541462097553
F	8.80785836866117	2.36658361984757	-0.62258266923637
F	7.29048397432661	3.11532379193643	0.76968036438433
C	6.26713380305914	-0.83798364811636	-3.67007130919624
F	6.01784531447284	-0.49262160256987	-4.96679939101501
F	7.57793577514888	-1.18309358384739	-3.59535121338541
F	5.53553783417516	-1.96228149400085	-3.42302431799625
O	1.11445993715146	5.99275361842534	-0.52809722290730
C	-2.33014432104772	4.87381023028730	0.68385275424339
C	-3.54757013719036	4.94114229464923	-0.02544506126720
C	-3.37416088995615	6.09323341050321	2.51104887421418
C	-4.66603058355865	5.54439681210326	0.54144309602665
H	-3.62801556642583	4.47968398952199	-1.00987774106933
C	-4.58257552347379	6.12303391657299	1.81130573427162
H	-3.30031786392795	6.55023930992979	3.49857276744466
H	-5.61080563783313	5.55338669646825	-0.00289696398321
H	-5.46033196306139	6.59715962862780	2.25294100009508
H	1.63667359409056	5.16658513550770	-0.48070413221915
H	1.24272110566198	5.45628930230860	-2.53532575291976
C	-0.83871124183052	5.57734798953766	-1.94992007558837
H	-1.49812598498412	6.28347583401397	-1.42359157307748
H	-1.15689484201470	5.56909000115941	-3.00365570278390
C	0.60872823254290	6.05823853950335	-1.86337015051047
C	-2.26262158814227	5.46662978340951	1.95801397711734
H	0.66559543045364	7.10873095127153	-2.19414064220160

H	-0.36365595831629	3.58820918594383	2.01340771632492
C	-0.32307055747841	3.43081488437082	0.93391319425615
C	-1.01545407840515	4.15806461295085	-1.36351365231262
C	-1.17109936665617	4.17322652723253	0.11910705144980
H	-1.92189684771949	3.71310825863138	-1.80457202372522
H	-0.17417363202902	3.51724829539063	-1.65468387802717
H	0.63487771408064	3.09828741948971	0.52913691869699
H	-1.32241108900365	5.45039452964582	2.50595823517038

178
3b_1 (cat)

H	0.83585999887289	-5.83991000981387	1.29774000863283
H	0.90537999998729	-1.88468990503682	3.36430991440632
H	3.14038002057474	-1.20769964863136	4.03126968370083
C	4.18346009363254	-2.51115981799999	2.65123988277742
C	3.04295995654414	-1.95603055016030	3.24535043827380
C	1.75719000286760	-2.34314024673242	2.86734024475711
C	1.55677999737958	-3.30942970494473	1.87144972999658
C	2.69792999427391	-3.84503988375081	1.24852988451434
C	3.97580993187412	-3.45696007016577	1.63289005666054
H	4.82808001611494	-3.90210000522482	1.11756999813187
H	2.57997999715397	-4.56029004578806	0.43310003073423
C	0.21411000621077	-3.80058002937446	1.49078002131569
C	0.00385999847049	-5.13986001793003	1.19958002004066
C	-1.25098000416396	-5.63645000483450	0.77498001416288
C	-1.41871999958010	-7.00242999861802	0.42254999899408
C	-2.62642999993837	-7.47151000132816	-0.04642000251922
C	-3.72117000108780	-6.58703999800736	-0.18108000164733
C	-3.59717000207372	-5.25799999882003	0.16882999937432
C	-2.37046000058147	-4.74485000349775	0.66550000424132
C	-2.18319999926754	-3.36682999869127	1.00669999021681
C	-0.91758000050020	-2.94597001763358	1.37971002432886
O	-0.75089999639420	-1.58188000362111	1.64662998994289
P	-0.68401999775923	-0.59113000079708	0.36668000140546
N	-0.93474000225810	0.90381000143569	0.82917000066079
S	-0.65496000059767	1.46620000134449	2.31421999788164
O	-0.73286999908143	2.92170999861241	2.24804000107838
O	0.51071999934689	0.85799999845789	2.95701000239593
C	-2.09426999953968	0.90075999857034	3.24050000150514
C	-1.97207000141611	-0.19689000095123	4.08716000219277
C	-3.10503000095561	-0.68644000155258	4.74160999783331
C	-4.34557999817218	-0.07403000235519	4.56952999956007
C	-4.44295000163526	1.04567999799362	3.73976000182966
C	-3.32273000149217	1.53683999901344	3.06691000139610
H	-3.39683999954409	2.40640000069706	2.41580999924466
C	-5.75377000124562	1.77555000155223	3.59659000216789
F	-6.02273000074725	2.09389999904574	2.29624000121277
F	-6.80789000006500	1.05245000020962	4.05673000040002
F	-5.74567999834469	2.95142000204756	4.28670000092484
H	-5.22807000039223	-0.46736000212831	5.06947999928348
C	-2.94619000069493	-1.87879999990455	5.64912999732968
F	-2.32245000019682	-2.91385000264859	5.01484999856214
F	-4.13309000165747	-2.34638000200486	6.11355000206294
F	-2.18072999890537	-1.57694000023040	6.73761000146427
H	-0.99795999955130	-0.66432000190750	4.22043999775025
N	0.59804000172123	-0.97145999972885	-0.49359999997546
P	1.81167999869161	-0.24308000047971	-1.10150000192416
N	1.75215999842521	1.41528999997400	-1.30400000077469
S	0.95366999881537	2.16173999854198	-2.59887999744994

O	-0.13264999916376	1.26476999874312	-2.93608000143433
O	1.94282999747805	2.56891999998975	-3.57843000031111
C	0.33269000130026	3.65563999777739	-1.83731999981507
C	-0.82239000249285	3.63976999974981	-1.04691000164300
C	-1.28556000066941	4.85707999970679	-0.54988999798462
C	-0.61206999746616	6.05265999908062	-0.82377999754938
C	0.53838999822239	6.03750999958714	-1.60442999948026
C	1.01762000071690	4.83292000155444	-2.12357000206955
H	1.90292999856881	4.80440000132227	-2.75767000146980
C	1.30149999822849	7.30409999747885	-1.89817000233111
F	0.69568999783384	8.40451000010555	-1.39012999969523
F	2.56096000002897	7.25491000197246	-1.36740000237399
F	1.45234999968661	7.49898999946384	-3.23601999794413
H	-0.98633000122086	6.99271999936673	-0.42033999832491
C	-2.54070000082213	4.95645999802048	0.28420999761032
F	-2.30560999918331	5.58442999787920	1.46547000131194
F	-3.09591999866821	3.75817999893666	0.55029999923239
F	-3.48715000110710	5.70547000248169	-0.36816999962343
H	-1.33501000208798	2.70473000200724	-0.81917999767481
H	2.56958000033863	1.93409000081011	-0.94894000138969
O	2.20455000092044	-0.86058000189617	-2.53797999831762
C	2.71401000804876	-2.18009000142933	-2.46266004185341
C	4.04334000158507	-2.32257000143535	-2.08784000404728
C	4.57380999996014	-3.64340000189392	-1.93364999863965
C	5.88490000078165	-3.89940999797134	-1.45339999454054
C	6.34145999977606	-5.19102999844482	-1.29079000029687
C	5.51136000230542	-6.29221999769284	-1.60361000207972
C	4.22883999769391	-6.07785999984275	-2.05821000182573
C	3.72150000116365	-4.76054000022431	-2.22047000019842
C	2.38507999593190	-4.53627000031076	-2.62186997922866
C	1.83752998255681	-3.26588999281570	-2.73055995124409
C	0.40791001208731	-3.10508002440095	-3.07293005924321
C	-0.06047012744577	-2.14470964196150	-3.97946961490966
C	-1.40744971577765	-2.10687043395825	-4.34481038964537
C	-2.34065016115907	-3.01112999281197	-3.81932002796306
C	-1.86976992916399	-3.93845964583022	-2.87473963684160
C	-0.53112994509182	-3.98603034872615	-2.50875036519765
H	-0.20879000526465	-4.70337994974800	-1.75257994446999
H	-2.55932002713453	-4.64240006125913	-2.40653005185115
H	-4.67200000094689	-6.95709000037051	-0.56761000040493
H	-3.86202999940497	-4.15828000095510	2.86885000031901
H	-7.84941999989145	-2.71999000151314	3.63982999798595
H	-7.58139999906255	-0.98772999768818	1.88250999781247
H	-0.56222999814523	-7.67307999770123	0.52027000280579
H	-2.73901000039503	-8.52142999987500	-0.32035999944649
H	-5.96823999914387	-4.28470999820337	4.14412000212743
H	5.17883807822709	-2.21117731845927	2.94736775073769
H	-4.44671000168075	-4.58554999793370	0.05819999840802
H	-6.23703000059179	0.07337999806788	0.14973999937356
H	-5.76228999775977	4.84271999827249	-3.89926999882690
H	-2.57613000245310	1.44568999758833	-4.25899000152788
H	-2.67631005852049	-0.32322992152489	-2.60375992138203
H	-1.72382004542424	-1.35446993811079	-5.06681996297808
H	0.62996001833035	-1.42761004965184	-4.42205005792747
H	1.75479999919296	-5.39903000113506	-2.84527999351165
H	3.57260999846410	-6.91953999857597	-2.28938000251376
H	5.88614000233804	-7.30862000164184	-1.47566000219055
H	7.35029999849976	-5.36348999892716	-0.91294000082438

H	6.53259000172360	-3.06034999794870	-1.20374999601896
C	4.84322000163842	-1.10947999900451	-1.78278999775828
C	4.36894000305807	-0.19738999891793	-0.85309000149841
C	5.02716000023744	1.01551999671414	-0.51570000131872
C	6.24496000184022	1.25895000242013	-1.12899000162514
C	6.78779999893832	0.38108000302951	-2.09557999971440
C	8.01576000241321	0.67436999858528	-2.74621999844299
C	8.49931999766975	-0.14132999770693	-3.74512000060336
C	7.76275000213623	-1.28032999954203	-4.14386000128606
C	6.57636000102056	-1.60423000227337	-3.51933999875449
C	6.06735000057200	-0.80503000005876	-2.46268000248183
H	6.01408000133345	-2.48043999916973	-3.83953999983228
H	8.13314000067957	-1.90620000193269	-4.95699000063925
H	9.43318580944408	0.09568413988305	-4.23744193187144
H	8.56146000184878	1.57172000037285	-2.44752000139372
H	6.79534000178492	2.16644000299293	-0.87298000071015
C	4.39842999887643	2.01494998468125	0.37810000467888
C	3.78358998989949	1.66779996335562	1.59158003263320
C	3.18859000004897	2.63856999923313	2.39446000153651
C	3.16945000313552	3.99032000063984	2.02518999800660
C	3.76858999963976	4.33097999995200	0.80008999776253
C	4.37343000136138	3.37069999853280	-0.00671999946450
H	4.81604000075315	3.66402000232685	-0.96133999985982
H	3.75809000163112	5.36763000266770	0.45896999782067
C	2.52661000150522	5.07371999980042	2.89489000050841
C	1.92416000119686	4.49951000172117	4.18461999747363
H	1.12889000242371	3.77332000240045	3.96824999952119
H	1.48180999868171	5.31670999813709	4.77237000027844
H	2.68826999785651	4.01531999998714	4.81062999909910
C	3.60036999955967	6.11633999911462	3.26808999945078
H	4.03366999735001	6.59202000061402	2.37694999801112
H	4.41849000249882	5.65146999778584	3.83745999994386
H	3.15472000050519	6.90726000233492	3.88971000000911
C	1.39861000089390	5.75801999825824	2.09723000002381
H	1.78180000242613	6.22557000160566	1.17979000246695
H	0.93234999923035	6.54613999767550	2.70753000039429
H	0.62354999963765	5.02841000049866	1.82893000096419
H	2.70195000157184	2.31523000409552	3.31196000112617
H	3.75503998881884	0.62660999723969	1.91117002853448
O	3.13487000263839	-0.46195999665461	-0.21738000173632
O	-1.88954000032930	-1.10326999884195	-0.58293999909809
C	-3.13794999891617	-1.28838999787820	0.02787000093935
C	-3.28483999674846	-2.37625000183495	0.87928999598116
C	-4.50320000163619	-2.50726999729083	1.61734000275400
C	-4.68084000178240	-3.47850999789736	2.63580999885453
C	-5.85993999936419	-3.54590000099992	3.34896999940735
C	-6.92161999839666	-2.65479999989381	3.06965000155089
C	-6.77716000233817	-1.69605000051117	2.09030999714381
C	-5.56817000155283	-1.58425999730587	1.35374999766692
C	-5.39177000166417	-0.57661999823582	0.37926999938509
C	-4.18919999993614	-0.37805999864472	-0.28381999960054
C	-4.05546999782197	0.70639999959422	-1.28257000136050
C	-3.26420000195416	0.57658999923792	-2.43855999936525
C	-3.21494999774351	1.59119000157911	-3.38689999841246
C	-3.93565000053565	2.78637000118280	-3.23810999919438
C	-4.72109999953055	2.91275000183555	-2.08504000089845
C	-4.77976999838882	1.89882999747165	-1.13001000074535
H	-5.37322999760371	2.05755999783639	-0.22900999897346

H	-5.28610999799197	3.82625000004837	-1.90383999851573
C	-3.83478999941811	3.87792000039227	-4.30792000167097
C	-2.36741999790031	4.33714999908042	-4.43634000160866
H	-2.02133000196683	4.79850999953391	-3.50070999773511
H	-2.27214000109702	5.08282000104918	-5.24002999813074
H	-1.69662999968439	3.49859999886980	-4.66889000031592
C	-4.30711000070216	3.30215999979280	-5.65895000235310
H	-5.35117999824411	2.96319999997955	-5.59471000068195
H	-3.69272000039391	2.44639999787027	-5.97093999890247
H	-4.24092999834347	4.07101000081785	-6.44361999943940
C	-4.69626999949720	5.10302000215619	-3.97288999989800
H	-4.59044999922835	5.85510999789026	-4.76807999755179
H	-4.38848999923209	5.57072000065674	-3.02653999822580
H	-3.38236499952091	-2.97548246671822	-4.10454042273669

178

3b_1 (TS1-1)

C	-4.00390999712461	-1.39392999994943	-2.33904000198956
F	6.86378999959662	-2.58448000074430	-0.43339000046048
F	7.53939999784456	-3.39770000253121	1.47914999948030
F	8.49614999857050	-1.61057000025432	0.65727000209471
H	4.60941000057161	-2.57628999920015	0.87884999855348
C	1.35076989636650	3.17412001502987	-4.20258992577151
C	-6.25479999872846	-2.04693000251425	-1.79688999856430
C	7.30005999938426	-2.23405000028294	0.81056000259785
C	2.40810000216670	-0.73061000323221	-3.40031999870106
C	1.36989994988677	4.09216031583102	-3.13895032152058
C	0.21836027401115	2.35718966734238	-4.31676984433466
H	-2.72422617167005	2.30418997135886	5.03480681673432
C	-4.88080999911161	-2.27413999988235	-1.70684999913735
H	-4.48932000132836	-3.13581000107450	-1.16656000065983
H	-2.44460000159546	-3.94403999935323	6.75532000158842
C	4.94378999808254	-1.73008000008594	1.48003000002737
H	-0.89059000126896	-3.12092000217433	6.49546999941751
C	3.62342000180992	-0.95937000098658	-2.72525000187360
C	1.85374999919982	-1.70216999868728	-4.22477000003485
H	1.88124999903093	0.21391000401955	-3.28267000091559
C	0.30547015857369	4.20062967035391	-2.25167972392979
H	2.23696999001205	4.73666994016985	-2.98757993931835
C	-0.85430020784029	2.46098033411595	-3.43092024414573
H	0.15186995212857	1.61887006463738	-5.11532000745702
C	4.26687000017849	0.06792000041178	-1.87476000174828
C	4.25038000267338	-2.19872999857173	-2.92940999826555
C	2.48169000014875	-2.94065000200516	-4.44014000239441
H	0.89257999809159	-1.48705000114346	-4.69424999796702
C	-0.83918001975987	3.39588000758632	-2.38575998992795
H	0.37298997145025	4.90489005207335	-1.42137003157614
H	-1.70782997112242	1.80030995079992	-3.56558996322507
C	3.51922999878385	0.99816000135814	-1.09713999969048
C	5.64694999881907	0.19579000026458	-1.83470000120277
C	3.69719999873412	-3.15938999961527	-3.77734999911723
H	5.17652999768815	-2.42872999798593	-2.40067000114522
C	1.82098000257948	-3.98248999921152	-5.34584000250531
C	-1.97638999995114	3.58383999938157	-1.45721000856015
O	2.12787000074982	0.87161999878646	-1.11042999931859
C	4.08384999929216	2.05128999920021	-0.38810999946995
H	6.26096000239947	-0.46878999765170	-2.44406999895552
C	6.29437999853445	1.15960999884738	-1.02880000185913
H	4.22566000257908	-4.10435000116680	-3.90119999790778

C	1.60576999784498	-3.37025999994450	-6.74553000098703
C	0.45649999998308	-4.37949999813373	-4.74473000027322
C	2.67553999981585	-5.24831000215196	-5.49851999901475
C	-2.72058999231299	2.49514999609392	-0.92380999634709
C	-2.35520000556400	4.85129999614595	-1.04404000889920
P	1.34918000264516	0.46057999853457	0.24367999787794
C	5.51179000144397	2.10443000100614	-0.28544999732556
C	3.21599999955308	3.10087000156393	0.21078999656616
C	7.71064999761667	1.21148999786889	-0.93754000020644
H	2.56352000062860	-3.06864999956700	-7.19437000205032
H	1.12970999960372	-4.10731999768190	-7.40930999926349
H	0.95584000115845	-2.48579999805617	-6.70457999840944
H	-0.19848999985216	-3.50932999811449	-4.60317000114077
H	-0.05727000226534	-5.08817999996420	-5.41145999750695
H	0.58106999751650	-4.87039000244902	-3.76896000013051
H	2.82697000066966	-5.75976000153471	-4.53625000263648
H	2.16454000212480	-5.95599999810143	-6.16658999986866
H	3.66126999874894	-5.02729999838281	-5.93423999842221
O	-2.37449999113792	1.20710000174086	-1.33766999135183
C	-3.77314998868926	2.62553999717897	-0.02941998698597
H	-1.83197000572611	5.71948000159161	-1.44957000273403
C	-3.39688000466744	5.06336000158574	-0.11094000734807
O	2.02469000135392	1.39223000227454	1.37153999816335
N	-0.12938999821016	0.81610000098281	-0.05477000087328
N	1.76150999895605	-1.05275000134883	0.70704000228241
C	6.18380000165838	3.02938000232325	0.55404999759595
C	3.35936000100440	4.48337999752828	-0.14625000324982
C	2.18572000430301	2.75397000042466	1.06982000355942
H	8.29611000164748	0.49057000024470	-1.51126000148270
C	8.33376000220198	2.13120000178259	-0.12208999803052
P	-1.57373000072488	0.24779000062782	-0.30653000077343
C	-4.45637999969899	1.41406999798016	0.49965000058387
C	-4.11789000614048	3.94234999894627	0.42238999364914
C	-3.72143000189206	6.37258000200277	0.33542000032294
S	2.29906999856100	-1.48318000143139	2.19977000121990
H	1.47055000192077	-2.11963000251891	-0.08184999783283
C	7.56086000162451	3.03926000012661	0.63711999763743
H	5.59701000060516	3.72516999876363	1.15221000065628
C	4.30342000301166	4.94440999824400	-1.10059999616638
C	2.46772999820993	5.44096999804967	0.44422999945839
C	1.28289999434922	3.67492999403461	1.66780999443894
H	9.42217999760885	2.15272999985518	-0.05157000173400
O	-2.31995999929639	0.52586999965275	1.10271000206744
N	-1.78639000168020	-1.25866000233618	-0.78175000257958
C	-3.71251000170061	0.41013000102510	1.10770000058099
C	-5.87562999882803	1.24212000245690	0.41041999852051
C	-5.10351999771148	4.18926999905984	1.41392000547447
H	-3.17110999978320	7.21579000005460	-0.08760000033843
C	-4.69394999955364	6.57773999970740	1.28930000023347
O	2.29107999952939	-2.93934999907732	2.20418000008176
O	1.62492000023292	-0.76016000109172	3.26643000233285
H	8.05581999778758	3.74571000154201	1.30456000249945
H	4.96773999603796	4.22799000151997	-1.58013000347469
C	4.38191999885226	6.28016000149114	-1.43760000059735
C	2.58685000001785	6.81094999896614	0.08737999978799
C	1.46586000060305	5.01005000181196	1.34493000436746
C	0.19159000568349	3.25291000444315	2.57281999876119
S	-2.23490999759085	-1.70811999804089	-2.25539999862329

C	-4.27802999834574	-0.71730000233794	1.76717999967304
C	-6.47621000219732	0.11398999833484	1.06203999746236
C	-6.70833999853980	2.11695000010530	-0.33343999930642
C	-5.38305000734324	5.47229000286775	1.83790999609685
H	-5.63992999947130	3.34840999789302	1.84993999619542
H	-4.92811000067317	7.58796000253766	1.62782999912407
H	5.11046000063865	6.60638999785744	-2.18134999903595
C	3.52309000186377	7.22638000015863	-0.83378000296040
H	1.90729000123189	7.52868000007661	0.55178000215389
H	0.81229000169711	5.75778000072355	1.79834000463871
C	0.36791003239549	2.27817006043376	3.56456995323674
C	-1.06989993046259	3.86479997829534	2.47787996802007
O	-1.61409999846565	-0.93409999905122	-3.32872999807188
O	-2.11086999770142	-3.16802999786265	-2.31304999785548
C	-5.66110999776973	-0.81380000132563	1.74995000102679
C	-3.46065999855012	-1.72403999751190	2.48250999852842
C	-7.88411000009263	-0.05689000020091	0.98959999890080
C	-8.07057999899825	1.90945000197490	-0.40339000238611
H	-6.25611999943686	2.95037999945357	-0.86923000121970
H	-6.14034000058130	5.63291999779432	2.60680999809504
H	3.59820999918045	8.28041000191640	-1.10446999792440
C	-0.66860012950197	1.94558989326738	4.43639014874319
H	1.32184999856500	1.76582998924420	3.66763000409940
C	-2.09658019844252	3.53100996837360	3.35337009127569
H	-1.25708000660866	4.59324001448746	1.68762000340856
H	-6.14426999852152	-1.63621000114636	2.27916000262825
C	-2.30037999967051	-1.38535000439574	3.20474999964400
C	-3.87697000017203	-3.06367999879841	2.52458999988328
H	-8.33051000219746	-0.91681000151843	1.49252000050220
C	-8.66787999907548	0.82162999824780	0.27332000170533
H	-8.68886999824097	2.58382000039283	-0.99729000198144
F	5.43917000221947	2.65594000099650	3.78957000245924
C	-1.92147985755987	2.56777005389274	4.36130989611620
H	-0.47790997798141	1.18352002835176	5.19130994937694
H	-3.05878993217139	4.03080000164323	3.23540998785807
C	-1.62776999986778	-2.33689000182161	3.96356000237825
H	-1.92425999180101	-0.36430998573178	3.19273000765543
C	-3.20461999829815	-4.00820000006264	3.30097999808835
H	-4.74490999995829	-3.37567000084992	1.94064999903835
H	-9.74660000165384	0.67027000224627	0.21374999937641
C	6.20747999902628	1.54511999788369	3.96761999818773
F	-7.22610000194319	-4.20689000236727	-1.90949999855198
C	-2.07555000176527	-3.66527000143434	4.05711999894628
H	-0.73252999741437	-2.02173999801495	4.50258000217266
H	-3.58359000195627	-5.03002000102687	3.31526999996163
F	6.08145000152593	1.19190000051903	5.27919000056033
C	5.77966999746036	0.41818999906164	3.06059000099537
F	7.50076000198039	1.90810000204606	3.77548999927646
H	-7.82208999744108	-0.76305000189621	-2.54852999786258
F	-6.81770999975633	-3.40299000186469	0.08056000139555
F	-5.70509000035027	2.25569999982661	-3.56657000137041
C	-1.36057999827788	-4.65086999767532	4.98782999860183
C	6.71803000022762	-0.30823000197039	2.32911999842452
C	4.42436000086828	0.08126999858118	3.01262999852727
C	-5.85689999776732	-0.07366000136325	-3.11862999840802
C	-4.47965999790699	-0.29798999932117	-3.05210999969766
C	-7.19638999848212	-3.05216000165754	-1.18458999949119
C	-6.75082000027007	-0.94445000148147	-2.49639999964360

H	-3.77549000049291	0.36400999904640	-3.55353000053626
F	-8.47291000139003	-2.59752999757196	-1.10938000082777
C	-6.35169999743309	1.10661999774874	-3.91518000265975
F	-6.13345999822658	0.93000999903161	-5.25013000133017
F	-7.68210999966871	1.32598000050208	-3.75487000080618
C	0.10996000229729	-4.81721000022752	4.55724999797780
C	-2.02294000244517	-6.03531999972229	4.99041000210863
C	-1.40585000243954	-4.08844999882906	6.42482000011596
C	6.29308000061118	-1.38248000231704	1.54252999823815
H	7.77094999980297	-0.03638999789465	2.36383000151416
C	4.02272999950727	-0.98856999767549	2.22004000194018
H	3.68349000238488	0.63296999901878	3.58965000163836
H	0.63240999769283	-3.85485999756082	4.47593999900980
H	0.64956999746330	-5.43953999760527	5.28720999867332
H	0.16900000178366	-5.31849000211699	3.58070999736262
H	-1.47975999792223	-6.70111999971413	5.67645999992553
H	-3.06826999738701	-5.98747999875962	5.32867000103072
H	-2.00252999737172	-6.49959999998217	3.99328999824195
H	-0.91498000105110	-4.78612999928466	7.12010000205165
H	2.18019975854093	3.09103884850721	-4.89052215376743

178

3b_1 (TS1-1′)

C	-3.90652000156903	1.38616000124258	-3.18522000205027
C	-6.25928000239687	0.19539000030002	-2.27747000086921
H	3.00773874258799	2.94116982366041	-4.95908308126213
H	-5.07235000147132	-1.59459000227057	-2.00435999704620
H	-6.82006000143435	-6.87530000126407	2.56350999935427
C	2.36630995168446	3.07379000969058	-4.09989993591021
H	5.08231999737139	-3.22116999952028	-0.34347000252725
C	-5.08236999894661	-0.55159000105236	-2.31888000072360
C	4.08321999750129	-1.92355999895655	-3.26975000176258
C	1.00327003754130	2.75326998002060	-4.14507009843290
C	2.86313008265751	3.55871999809130	-2.87815006335491
C	5.40083999827457	-2.62742000064002	0.51319999897687
F	7.34327999751551	-2.58014999765564	-1.59612000164905
C	7.75920000087892	-2.76097999933274	-0.30767000018642
F	8.95648999936783	-2.13490999968477	-0.18149999824649
C	-3.90969000071669	0.06065000013485	-2.76297999792643
F	7.98870000209013	-4.09664000039520	-0.16180000081101
C	3.48303000202279	-0.76144999735176	-2.75979999766238
C	3.47795000132861	-2.66679999794192	-4.28400000118817
H	5.03824000160882	-2.25956999979455	-2.86222000262821
C	0.17743999352791	2.89834000989387	-3.03079995810905
H	0.56157999373048	2.36383000956490	-5.06129997087957
C	2.04300996049724	3.71796000092046	-1.76743997897266
H	3.92041997391635	3.81425000030333	-2.78369998672075
C	4.19491000195816	0.04850999763906	-1.74699000085568
C	2.23231999802007	-0.39328999870543	-3.28750000267975
C	2.24662000156505	-2.28415000069801	-4.83325000162738
H	3.98699000069979	-3.55979999919005	-4.64607000052292
C	0.67861000264625	3.38809000055701	-1.81560000542484
H	-0.87208000262632	2.62739999594748	-3.11938000808961
H	2.47317000580071	4.08562000238690	-0.83674000369071
C	3.52787999823308	0.71427000251151	-0.68197000010725
C	5.57281000195578	0.18945999822590	-1.79557999911027
C	1.63721999955897	-1.13703999810066	-4.29920999893425
H	1.72658000247929	0.50054999697182	-2.93365999754364
C	1.56144999904045	-3.04417999877125	-5.97198000144381

C	-0.19588000058702	3.64597999902782	-0.64962000096177
O	2.13007000161926	0.62618000063869	-0.63817999757214
C	4.16705999760280	1.45026999803749	0.30793000088780
H	6.12395000112789	-0.25828999842128	-2.62352000160405
C	6.29849000150852	0.87567999982243	-0.79580000154178
H	0.66402000137614	-0.80757999936536	-4.66437000146310
C	2.38396999921212	-4.24942000106323	-6.44640000014774
C	1.36609999917579	-2.08457999775440	-7.16469999909162
C	0.18449999909186	-3.54756999951488	-5.49517000034031
C	-1.31615000021680	2.82643000292724	-0.32582000176387
C	-0.00144000210621	4.76508000211454	0.14458999850879
P	1.50030000145900	-0.35086000025290	0.48107000050922
C	5.60015000050825	1.48906000165244	0.29724999803147
C	3.36524999558332	2.16518000059243	1.33682999912814
C	7.71678999842674	0.93778999949577	-0.83341000148788
H	2.53340000122771	-4.98322999875989	-5.64041999984774
H	1.85369999764879	-4.75767999849643	-7.26451999767862
H	3.37130999865253	-3.94669999854395	-6.82495000121593
H	0.87952000036807	-2.61169999790196	-7.99917000070118
H	0.73482999735784	-1.22787000102575	-6.89262000254674
H	2.33290000115055	-1.69693000082750	-7.51770000159871
H	0.30356000253776	-4.27188000159404	-4.67592999925905
H	-0.45484999893343	-2.73153000180074	-5.13234000202566
H	-0.34198999971324	-4.05324000261863	-6.31883000168576
O	-1.47088999825429	1.62716000192741	-1.03257999851244
C	-2.28752999887729	3.16926999817791	0.60167999926492
H	0.83561999903445	5.43146999730878	-0.07041000106228
C	-0.86288000271924	5.08933000345175	1.21802999715106
O	2.23271000176147	0.11340999915764	1.83955000027956
N	-0.03200000220916	-0.10732000110839	0.49277000171855
N	2.03608999749789	-1.87564000214645	0.24308000051853
C	6.36228999756797	2.07526000211307	1.34020999881294
C	3.51360999581254	3.57588000054940	1.55943999423938
C	2.39355999047952	1.49063001776863	2.06564999322017
H	8.23546000165794	0.47306999876384	-1.67405999903187
C	8.42629999881018	1.54030999921546	0.18273999807509
P	-1.38528000063313	0.21390000006413	-0.24379999924986
C	-3.56438999793419	2.41321999928654	0.69318999750752
C	-2.03849000061413	4.30095000105507	1.44731000191261
C	-0.59654000161964	6.19775000248767	2.06472999921136
S	2.78232000153621	-2.84892000129246	1.35202999741478
H	1.32231999932726	-2.60768999887816	-0.64105000106572
C	7.74077000231239	2.09929999842860	1.28491999791941
H	5.84664999954851	2.49751000171799	2.20116000165366
C	4.37658999889783	4.39742999846384	0.78573000175014
C	2.71270000254434	4.20203999714005	2.57138000655356
C	1.54784001439819	2.09508999713248	3.03546000277175
H	9.51623000136747	1.57034000148713	0.14768000239916
O	-2.37639999753249	0.36348999774912	1.03169000049094
N	-1.89254999741909	-0.91141000208348	-1.24306000090143
C	-3.59184999865293	1.04367999910722	0.92565000088720
C	-4.81134999756107	3.10788999766215	0.53388000059271
C	-2.87589000029737	4.63826000226621	2.54261000071256
H	0.29611000259926	6.79473999907767	1.86909000050057
C	-1.43086000219412	6.50046999916007	3.11890000046582
O	2.85941999903169	-4.15070999927383	0.70470999798696
O	2.20164000234757	-2.71711999843029	2.67825000101069
H	8.30517999846987	2.54144000215994	2.10681000204443

H	4.95914000062643	3.94859999860893	-0.01711999868993
C	4.47647999789367	5.75228000232480	1.02625999902673
C	2.86108999649465	5.59410000232636	2.81324999959276
C	1.75868002685565	3.44213999718531	3.28378003669114
C	0.44987992408501	1.37250993777519	3.71707985636462
S	-2.36599999852254	-0.85883999847202	-2.76903999787228
C	-4.79430000851316	0.32097000022092	1.19003999528962
C	-6.03216999788928	2.40101999909764	0.78379000217930
C	-4.89193000193628	4.45439999965300	0.09311000040027
C	-2.57434000264817	5.70547000287781	3.36355000069458
H	-3.75587999870622	4.02841999841945	2.74362999882353
H	-1.21061999864947	7.34926000141702	3.76813000175951
H	5.14048999863775	6.36127999917133	0.41116000218961
C	3.72569999857510	6.35731000156341	2.06018999544903
H	2.25710999641147	6.04812000275533	3.60120999860342
H	1.14972000169107	3.94343000114550	4.03790999851859
C	0.56837018845672	0.06769021659493	4.22494053200794
C	-0.77783018602110	2.02777988420308	3.88847972957911
O	-1.45409999929995	-0.12194999891959	-3.63945000100915
O	-2.70739999736575	-2.23646000030639	-3.14521999952978
C	-5.98048000108734	1.03599000066980	1.14254000165960
C	-4.84004995838553	-1.12203999200884	1.52263002844545
C	-7.27250000048760	3.07392000163508	0.62583000298869
C	-6.11285000118425	5.07650000060703	-0.06595000075470
H	-3.97601999840983	4.99293000214485	-0.14453000114794
H	-3.22308000096957	5.93598000160952	4.20981000249478
H	3.82546000300907	7.42685000094211	2.25086000319910
C	-0.49431000514866	-0.52883017304236	4.89783955217832
H	1.49366995328608	-0.49254003778023	4.10138991588884
C	-1.84150964852930	1.41554013881325	4.54624030006405
H	-0.91557997039919	3.02623001148305	3.47462003564855
H	-6.91792000150553	0.50730999784840	1.32329999900575
C	-5.66540007242246	-1.57104001130976	2.56928995187139
C	-4.15701998432676	-2.09054999934813	0.77819000959297
H	-8.19273000046773	2.51891999830518	0.81793000174981
C	-7.31501000169366	4.38749000242290	0.21215000019455
H	-6.14995999758570	6.10531999998210	-0.42639999825408
F	6.55660000017767	-1.24716000255189	5.09594999887804
C	-1.72468023891522	0.12568999815766	5.08022998633896
H	-0.34889000026385	-1.53610997060083	5.29163007620474
H	-2.77775008774860	1.96592998257709	4.63442996848397
C	-5.78232001812365	-2.92723000113988	2.85673998369287
H	-6.20282992893849	-0.84316998929275	3.18029005041621
C	-4.28968000125698	-3.44937999961548	1.06069999812879
H	-3.51264000840939	-1.79052000189966	-0.04495000552260
H	-8.27339000046384	4.89252999889354	0.08353999918379
C	6.59883999912468	-0.42512999932259	4.00832999768377
H	-2.54799272886839	-0.35467110512550	5.58900758116212
C	-5.09409997486391	-3.90112999603351	2.11275002161473
H	-6.41834999077117	-3.22960999918479	3.69092000622525
H	-3.73735000796762	-4.15380999914894	0.43923999657515
F	7.86018999966972	0.07195999860447	3.93518000121694
C	6.19109000000549	-1.16013000018315	2.75599000094859
F	5.76353999898667	0.61724000061588	4.27739000109571
F	-8.20007000054775	-1.01836000224596	-2.90388999838384
H	-7.19068999794552	2.11568000159300	-2.62885000151870
F	-4.94538000096564	3.58475000074760	-4.99834999920050
C	-5.22899999684151	-5.38436999750120	2.46720999935983

C	7.14099000259480	-1.53709999968177	1.80670000200690
C	4.85019999842448	-1.51757000078753	2.60100999873374
C	-6.27224000206443	1.53382000061332	-2.68162999890483
F	-7.33308000096557	-1.45878000022363	-0.94457999912741
H	-2.98168000040474	1.83159000020389	-3.54799000215955
C	-7.54418000265511	-0.46458000139531	-1.84292999786095
C	-5.09429000164731	2.12021999808559	-3.14039999953984
F	-8.40663000042974	0.42340999869094	-1.27140999798052
C	-5.07880000056256	3.54261000038699	-3.64021000000001
F	-6.21404000251921	4.21741999909693	-3.33116000090022
F	-4.03006999971878	4.24575999981584	-3.12713999867418
C	-4.79483000137969	-5.59785000154634	3.93204999526197
C	-4.35302000455071	-6.27206000306524	1.57076999989586
C	-6.70090000230556	-5.81293999811677	2.30130999970171
C	6.73910000042168	-2.27073999926451	0.68870000085577
H	8.18478000184593	-1.25841999924004	1.93380000121396
C	4.46954999911459	-2.24695000191668	1.47792000110293
H	4.10501999825648	-1.25498999967094	3.35019999810486
H	-5.41358999904828	-5.01659000184764	4.62986000101002
H	-4.88678000138239	-6.66056000031553	4.20320000198959
H	-3.74748999999953	-5.29552000139679	4.07263999874842
H	-4.47955999961955	-7.32461000078187	1.86413000091226
H	-4.62904999993537	-6.18226000108354	0.51042999857833
H	-3.28731000206344	-6.02001999918099	1.66762999840031
H	-7.36840000211937	-5.22688999912727	2.94872000224927
H	-7.03166000172890	-5.67497000124388	1.26183000127755

178

3b_1 (TS1-2')

C	6.06020000169310	2.87504999930436	2.55422999838886
F	6.02401000105706	2.99092000080527	1.19201000060770
H	3.07735423394003	-5.20350017576578	-3.54056647609182
C	-3.63898000168433	-1.93033000094335	-3.34545999894997
F	6.45429000058934	4.08248999763768	3.03887999797976
C	2.68407994893277	-4.19742986119626	-3.55666998171864
H	5.05008999913552	0.34114999968820	2.69754999866985
H	-2.72238999806709	-2.41825999981535	-3.67279999760546
C	1.60212999728106	-4.26885999446855	-0.07600002098782
C	1.30889001748081	-3.94780018218218	-3.65101055041806
C	3.52701007102591	-3.07614013629842	-3.47221963701100
F	7.03782000009680	1.97916000252984	2.84581000044230
H	-8.19398000167819	1.65758000261057	4.97712000229431
C	4.36626000024886	1.10085999905424	3.07616999923519
C	-5.18576000205479	-1.39121999893874	-1.55406000227581
C	-4.00434000165381	-1.98215999813073	-2.00340999792748
C	-4.45383999785150	-1.24380999863716	-4.24815999838497
C	2.70251999935700	-4.14102000102130	0.78669999868765
C	0.53256000220287	-5.10449000058773	0.24002000088561
H	1.57604001731228	-3.72213999012302	-1.01546984167039
C	0.79460002693129	-2.65095991842677	-3.64575019299740
H	0.60386999486316	-4.77568995410473	-3.71371979888220
C	3.02253994302635	-1.78228996166148	-3.46912984250611
H	4.60702998165798	-3.21189996778880	-3.39048018693103
C	3.85529999829326	-3.25813000094231	0.50524000011216
C	2.67924999728155	-4.87875000139702	1.98253000000883
C	0.51044000036763	-5.85470999638152	1.42543000013254
H	-0.30363000180485	-5.14351999745273	-0.45732000161450
C	1.64043000310893	-1.53547001088135	-3.54950991626358
H	-0.28247001120491	-2.51887001584726	-3.71621989611227

H	3.71449001412749	-0.94683000613804	-3.36791009858091
C	3.69309000106792	-1.95345000249116	-0.03119000139404
C	5.14636999805810	-3.62619000037515	0.84130000196278
C	1.61192000017184	-5.71942999916169	2.28694999914177
H	3.48954999980300	-4.75380999877996	2.70228999824829
C	-0.63011999977112	-6.82734999916909	1.74463000288935
C	1.14041998724060	-0.14311001000996	-3.58692006065035
O	2.39432999770640	-1.56214999982627	-0.39035999742568
C	4.71214999938164	-1.02993999770752	-0.19508999899939
H	5.32995000125932	-4.63145999971954	1.22499000205297
C	6.23094999783794	-2.72164999876564	0.76692999770845
H	1.63990000173503	-6.26807000150871	3.22904000244956
C	-0.94767000233851	-6.83759999851924	3.25176999978339
C	-0.17751999817171	-8.24200999849227	1.32223999827941
C	-1.91752999804868	-6.46887000232688	0.98501000036039
C	-0.12970998008485	0.24904001127612	-3.07198995410554
C	1.89622998886877	0.86082999653675	-4.17628002394803
P	1.65707999919644	-0.56989999801185	0.63554999781394
C	6.01843999810596	-1.38760999986813	0.28180999875593
C	4.44576000283892	0.29102000096019	-0.82131999930028
C	7.52503000023340	-3.09441000205499	1.21730999999308
H	-0.10650999968684	-7.20514999797162	3.85496000148236
H	-1.20709000056074	-5.83038999794481	3.60925000052323
H	-1.80524999829247	-7.49798000131785	3.44677000183263
H	0.04112000069601	-8.27428000246660	0.24517000191499
H	0.73147999866388	-8.54368999836588	1.86260000151700
H	-0.96822000138789	-8.97764000063505	1.53631000166137
H	-2.24874000190168	-5.44161999747395	1.19210999925976
H	-1.79848000139081	-6.56055000105275	-0.10276999747528
H	-2.72419999793357	-7.15357000008710	1.28507999989498
O	-0.88409000330480	-0.70437000342759	-2.36141000474257
C	-0.67990000046104	1.51096000141807	-3.23581999917344
H	2.86616999907633	0.61103000051140	-4.60903999777962
C	1.46304000135884	2.20430999929198	-4.23727999176747
O	2.62033000181088	0.72452000145298	0.69833000153767
N	0.25153999930131	-0.27336000175226	0.05357000075052
N	1.56953000043750	-1.07840999979273	2.17240999970735
C	7.09578999798842	-0.46666000125835	0.34442999863623
C	5.23804000258101	0.74008999822570	-1.93345999799345
C	3.43598999922468	1.13158000117701	-0.36494000257464
H	7.67661999777762	-4.11592000048622	1.57216000060334
C	8.55843999809475	-2.18339999958236	1.23905000197493
P	-1.07005000099855	-0.46158000029776	-0.77126000177377
C	-2.09489000071925	1.78542999834067	-2.87339000525230
C	0.15452999944931	2.54926999872239	-3.76622000195999
C	2.30303000198893	3.22458999810928	-4.75722999993740
S	2.63693999917383	-1.00262000191127	3.43172000090084
H	0.41643999936175	-1.73469000143999	2.51310000146269
C	8.33318999898182	-0.85390999839583	0.81673000246163
H	6.93529000038216	0.56361000194824	0.03036999858291
C	6.14673000278173	-0.11036999749311	-2.61729999963780
C	5.07403000156432	2.08031999865680	-2.41291999952074
C	3.25253999795026	2.46695000394469	-0.82616999806194
H	9.54398999741010	-2.47934999994065	1.60123000133982
O	-1.69606999803313	1.03548999733473	-0.63842000086580
N	-2.07137999999515	-1.53579000054020	-0.18675000212836
C	-2.56671999859949	1.57134999965810	-1.58575999723079
C	-3.00096000076896	2.30275999855506	-3.85850000072549

C	-0.23469000222550	3.91317999843695	-3.78198999985354
H	3.29185999928856	2.94396000045063	-5.12548999893280
C	1.89159000020989	4.53924999761422	-4.76922000123538
O	3.86045999950679	-1.73784999903131	3.16024000130634
O	1.83602999805515	-1.33215000040070	4.60200000087147
H	9.14123000148950	-0.12309000008522	0.87167000048804
H	6.25754999965051	-1.14209000005341	-2.28635000212042
C	6.87300000073564	0.34579000007025	-3.69802000111734
C	5.85463999847449	2.52716999782145	-3.51195000235459
C	4.10728999350009	2.91918000077606	-1.81503000126307
C	2.19364001881515	3.36657998753570	-0.31289999894038
S	-2.88109999778473	-2.75305999975627	-0.83217999790225
C	-3.84554999780242	2.01296000065003	-1.13627999929105
C	-4.31055000159866	2.71097000193600	-3.44319000143656
C	-2.67154000207219	2.39074999772991	-5.23568999877927
C	0.61769000054281	4.88527000189081	-4.26192000039922
H	-1.21028000273399	4.19184999762308	-3.38629000064682
H	2.54995999861229	5.31728999759899	-5.15903000192429
H	7.55444999816761	-0.33170000086929	-4.21452000233768
C	6.73899000061829	1.68050000114296	-4.14394999834639
H	5.72657000187076	3.55631999767502	-3.85420999851536
H	3.97710000075991	3.93244000004320	-2.19815000065468
C	2.48543001794428	4.68724991944895	0.05560015801195
C	0.85223001218825	2.96398005389716	-0.28724017041708
O	-2.04021999851242	-3.66406000093772	-1.60098000222380
O	-3.70741999819444	-3.31200000235930	0.24529999964991
C	-4.67609999748667	2.59114999893101	-2.08377999826661
C	-4.30456000162394	1.91858000201329	0.26873999931028
C	-5.22395999755942	3.20724999840840	-4.41041000090226
C	-3.58403000225805	2.87013000058693	-6.15233999853218
H	-1.69207000030642	2.05302000125858	-5.57091000176387
H	0.30694999354308	5.93097000475724	-4.24618001911368
H	7.32713999743932	2.03224000082330	-4.99271000201179
C	1.46593992165321	5.57725017081984	0.40442988639550
H	3.52460000171845	5.02314000490019	0.07024998564206
C	-0.15665011623027	3.85537000679814	0.05191019286244
H	0.58749000288065	1.95188999153525	-0.57467997851595
H	-5.67264000195326	2.91600999799996	-1.78023999861818
C	-4.17641999992323	0.75688000195943	1.04052999779912
C	-4.97855999931071	3.01027000043461	0.84630999766217
H	-6.21966000207259	3.50848000187284	-4.07860000011613
C	-4.86932000081564	3.28845999879356	-5.73939999838519
H	-3.31560000022196	2.91123999990205	-7.20879000160964
F	2.27609000204768	5.17061999864082	5.02261000135884
C	0.11926011292656	5.18826989952377	0.39192995858058
H	1.73842001656134	6.59500995837293	0.68288001150924
H	-1.18810996759005	3.50187999520035	0.02284997283303
C	-4.74098999805470	0.66816999952852	2.31311000125073
H	-3.64413999805759	-0.10630000103938	0.64591999765709
C	-5.53092000070902	2.91962999874664	2.11955999766354
H	-5.06285999768264	3.94467000068798	0.28761999965085
H	-5.58127000028915	3.66349999914907	-6.47598000067003
C	1.63446000169066	4.03714999918988	4.63117000012776
H	-0.66984623479791	5.87891166432416	0.65232869869812
C	-5.44405000032489	1.73914000076956	2.87736000068414
H	-4.61832000062127	-0.26825000241616	2.85699999755708
H	-6.04998000225911	3.79020999997523	2.52479999845295
F	0.71091999779941	4.41117000057849	3.69885999843140

C	2.58796000243106	3.00461000034315	4.08442999840363
F	0.94526999859588	3.57103999825531	5.70631000253076
F	-8.31941999835751	-0.98159000221384	-2.11017999826908
C	-4.05310999966100	-1.21521999867909	-5.70148000103372
F	-4.22781999817309	-2.43679000218250	-6.28512000062785
C	-6.13794999804306	1.64381999816755	4.23942999791780
C	3.83357000253033	3.40566000251619	3.59830999737118
C	2.20700000131388	1.66123999854763	4.04827000207701
F	-7.63071999753538	0.99170000159299	-2.75781000032441
C	-7.28704000163371	-0.09462000168810	-2.00844999805064
F	-7.23540000032401	0.30692999879522	-0.71417999982534
C	-5.62474999912659	-0.62386999831594	-3.81563000086901
H	-5.46450000064395	-1.45338000076106	-0.50292000142203
C	-5.98874999765554	-0.70931999928268	-2.46873000073626
H	-6.24343999812555	-0.06932999843085	-4.51897000137585
F	-2.73639999849275	-0.89841999829918	-5.85317000246521
F	-4.77460000051231	-0.32185000201366	-6.42296000047109
C	-5.70016000024583	2.81259000158471	5.14402999752614
C	-5.81417000208185	0.32660999862499	4.95941999836377
C	-7.66298000014901	1.72068999927183	4.01486999825560
C	4.72188000141789	2.44968000226707	3.10455000040895
H	4.11849999864739	4.45570999885612	3.62676999961645
C	3.10375000095135	0.72735999958494	3.53298000173761
H	1.23229000126644	1.34286000202227	4.41833000034496
H	-5.95190000185912	3.78803000154627	4.70552999738047
H	-6.20611000159283	2.74406000262020	6.11894000005497
H	-4.61538999934435	2.78572000222959	5.31525999969045
H	-6.32252000199023	0.30772000215362	5.93471999920305
H	-6.15145999800489	-0.54849999764199	4.38650000188026
H	-4.73419999830177	0.22090999987039	5.13442000183794
H	-7.94922000090497	2.66372000134999	3.52798000214177
H	-8.00479000023677	0.89365000022392	3.37620999967973

158

3b_2 (cat)

H	0.83586000679889	-5.83991003433506	1.29774000282563
H	-5.37322999349435	2.05755999721857	-0.22900999964629
C	-3.83478999294965	3.87792000088098	-4.30791999767644
C	-2.36741999816402	4.33714999886984	-4.43634000114878
H	-5.28611000359554	3.82624999766379	-1.90384000176365
C	-4.77976998102137	1.89882999610064	-1.13000999431507
H	1.17063269562912	-3.39937002067521	1.78821672276092
C	-3.26420013820579	0.57659011285153	-2.43855998956466
C	-4.72109999127303	2.91275000100417	-2.08503999715241
C	-3.93564998303981	2.78637000037042	-3.23810999189347
C	-3.21495000391037	1.59119000086460	-3.38690000311759
C	0.21410988871268	-3.80058022406447	1.49078012008225
C	0.00386006774987	-5.13985983189224	1.19957980467459
C	-1.25097998733935	-5.63645001833070	0.77498000971174
C	-1.41872002450582	-7.00243001349314	0.42255005320658
C	-2.62642999419112	-7.47150999800136	-0.04642002131309
C	-3.72117000162705	-6.58703999914102	-0.18107999690740
C	-3.59716999851470	-5.25799999371927	0.16882998685392
C	-2.37046001421994	-4.74485000254013	0.66550002396500
C	-2.18320003138302	-3.36683006110494	1.00670006039647
C	-0.91757992364262	-2.94596984851212	1.37970993898938
O	-0.75090002440230	-1.58188003439794	1.64662997293190
P	-0.68402000093389	-0.59112999986219	0.36668000722059
N	-0.93473999740708	0.90381000291796	0.82916999860601

S	-0.65496000096176	1.46620000101376	2.31421999804210
O	-0.73286999945068	2.92170999852615	2.24804000111647
O	0.51071999971752	0.85799999874657	2.95701000238083
C	-2.09426999972221	0.90075999842887	3.24050000173838
C	-1.97207000161178	-0.19689000084250	4.08716000241501
C	-3.10503000125171	-0.68644000137489	4.74160999796871
C	-4.34557999837240	-0.07403000226688	4.56952999963857
C	-4.44295000173602	1.04567999804419	3.73976000184860
C	-3.32273000157096	1.53683999907351	3.06691000144668
H	-3.39683999961033	2.40640000072082	2.41580999920628
C	-5.75377000133027	1.77555000157240	3.59659000206841
F	-6.02273000058178	2.09389999906862	2.29624000019429
F	-6.80789000009731	1.05245000012759	4.05673000055064
F	-5.74567999833597	2.95142000200247	4.28670000107427
H	-5.22807000053779	-0.46736000213886	5.06947999927831
C	-2.94619000079946	-1.87879999978646	5.64912999738346
F	-2.32245000014082	-2.91385000247531	5.01484999857772
F	-4.13309000166816	-2.34638000206204	6.11355000209461
F	-2.18072999892961	-1.57694000019892	6.73761000148848
H	-0.99795999862329	-0.66432000247760	4.22043999754097
N	0.59804000736038	-0.97146000158502	-0.49359998788742
P	1.81167998188186	-0.24308000163880	-1.10149996142587
N	1.75215999504959	1.41529000064966	-1.30399999425532
S	0.95367000086667	2.16173999867949	-2.59887999825290
O	-0.13264999782617	1.26476999856221	-2.93608000130377
O	1.94282999759787	2.56892000053474	-3.57843000109498
C	0.33269000206631	3.65563999797768	-1.83732000010531
C	-0.82239000260178	3.63976999959167	-1.04691000158290
C	-1.28556000076672	4.85707999965919	-0.54988999797001
C	-0.61206999746029	6.05265999906645	-0.82377999752349
C	0.53838999826932	6.03750999950859	-1.60442999939560
C	1.01762000065004	4.83292000142417	-2.12357000206415
H	1.90292999754171	4.80440000148134	-2.75767000194400
C	1.30149999826232	7.30409999745541	-1.89817000229839
F	0.69568999784513	8.40451000010137	-1.39012999969761
F	2.56096000008247	7.25491000201459	-1.36740000237172
F	1.45234999958225	7.49898999936428	-3.23601999793933
H	-0.98633000120603	6.99271999938803	-0.42033999834772
C	-2.54070000088585	4.95645999798764	0.28420999761804
F	-2.30560999917715	5.58442999785871	1.46547000132634
F	-3.09591999873437	3.75817999889126	0.55029999916704
F	-3.48715000112872	5.70547000249000	-0.36816999967465
H	-1.33501000230726	2.70473000195449	-0.81917999761618
H	2.56958000245643	1.93409000087610	-0.94894000410872
O	2.20455000824694	-0.86057998492754	-2.53798015690352
C	2.71401007204487	-2.18009015721417	-2.46265987474602
C	4.04333994744278	-2.32256997425334	-2.08783986148678
C	4.57380998129936	-3.64339999335872	-1.93364995061473
C	5.88489999030822	-3.89940999831760	-1.45339997669342
C	6.34145999335463	-5.19102999840323	-1.29078998770964
C	5.51135999025466	-6.29221999660122	-1.60360997989940
C	4.22883995999696	-6.07785999894299	-2.05820989298824
C	3.72150011379737	-4.76053999794747	-2.22047018908263
C	2.38508011603998	-4.53627017576390	-2.62187039413806
C	1.83752974338638	-3.26588969080622	-2.73055983800397
H	0.80878339869015	-3.08367939569727	-2.99968435232182
H	-4.38848999927476	5.57072000048322	-3.02653999866341
C	-4.69627000040809	5.10302000113046	-3.97289000018309

H	-3.69272000027126	2.44639999807110	-5.97093999917643
C	-4.30711000061548	3.30215999893935	-5.65895000224645
H	-2.27214000142487	5.08282000098916	-5.24002999804796
H	-1.69663000012061	3.49859999891768	-4.66889000023135
H	-5.35117999854394	2.96319999939995	-5.59471000106918
H	-4.67200000235362	-6.95709000138512	-0.56760999618991
H	-3.86202999943979	-4.15828000090517	2.86885000053991
H	-7.84941999988095	-2.71999000150055	3.63982999791580
H	-7.58139999913435	-0.98772999779902	1.88250999768001
H	-0.56222999653055	-7.67307999657858	0.52027000108836
H	-2.73900999916866	-8.52142999900851	-0.32036000285009
H	-5.96823999921227	-4.28470999827416	4.14412000211599
H	-2.02133000239905	4.79850999965449	-3.50070999787537
H	-4.44670999867123	-4.58554999654392	0.05819999053651
H	-6.23703000065499	0.07337999827901	0.14973999851397
H	-5.76228999860420	4.84271999865924	-3.89926999878522
H	-2.57613000336756	1.44568999113639	-4.25899001019178
H	-2.67630979520687	-0.32323016668961	-2.60376001477674
H	-4.59045000020101	5.85510999753815	-4.76807999822223
H	-4.24092999892372	4.07101000099724	-6.44361999942510
H	1.75479996735851	-5.39902997226874	-2.84527977469114
H	3.57260998829948	-6.91953999861286	-2.28937998475365
H	5.88614001550507	-7.30861999996309	-1.47566004247570
H	7.35030000038595	-5.36348999929369	-0.91294000396459
H	6.53259001359136	-3.06034999912582	-1.20375003144034
C	4.84322001478985	-1.10947999321720	-1.78279005021583
C	4.36893999553098	-0.19738999720253	-0.85309001628235
C	5.02715998770378	1.01551999484198	-0.51570000426675
C	6.24496000268372	1.25895000067999	-1.12898999937611
C	6.78780000190090	0.38107999898558	-2.09557999252523
C	8.01576000309362	0.67436999708621	-2.74621999644674
C	8.49931999773067	-0.14132999808081	-3.74512000022107
C	7.76275000199034	-1.28032999994321	-4.14386000022612
C	6.57636000191764	-1.60423000262018	-3.51933999621086
C	6.06735000494969	-0.80502999788740	-2.46268000496946
H	6.01408000024083	-2.48043999865612	-3.83954000065978
H	8.13314000014730	-1.90620000141411	-4.95699000107094
H	9.43318999979115	0.09568000264522	-4.23744000163067
H	8.56146000121323	1.57172000081928	-2.44752000236099
H	6.79534000130959	2.16644000302229	-0.87298000099731
C	4.39843003631730	2.01495001563984	0.37810001420366
C	3.78358987589817	1.66779990468303	1.59157995997548
C	3.18859003008044	2.63857002432951	2.39446001554866
C	3.16945002177276	3.99032000768469	2.02519001313439
C	3.76858999643633	4.33097999795425	0.80008999699024
C	4.37343002555527	3.37070000516580	-0.00671999071204
H	4.81603999326143	3.66401999845523	-0.96134000274417
H	3.75808999793174	5.36763000025030	0.45896999610079
C	2.52660999694083	5.07371999889508	2.89488999905844
C	1.92415999997511	4.49951000131395	4.18461999700240
H	1.12889000211864	3.77332000273749	3.96824999978569
H	1.48180999968809	5.31670999832944	4.77237000065719
H	2.68826999793934	4.01531999950057	4.81062999874919
C	3.60036999783875	6.11633999883012	3.26808999798962
H	4.03366999762930	6.59202000050268	2.37694999794930
H	4.41849000262475	5.65146999765646	3.83745999986384
H	3.15472000066275	6.90726000203309	3.88971000023552
C	1.39860999888741	5.75801999720710	2.09722999949985

H	1.78180000256774	6.22557000107336	1.17979000229701
H	0.93235000011441	6.54613999818549	2.70753000015877
H	0.62354999932632	5.02841000065285	1.82893000137718
H	2.70194998981156	2.31522999697085	3.31195999322153
H	3.75504008737783	0.62661010416189	1.91117001900659
O	3.13487000046991	-0.46195999309916	-0.21737999862385
O	-1.88954000109652	-1.10326999786885	-0.58293999781617
C	-3.13795000050842	-1.28838999945706	0.02787000039045
C	-3.28483999537004	-2.37624999754621	0.87928998908138
C	-4.50319999980367	-2.50726999524605	1.61734000570819
C	-4.68084000188365	-3.47850999833855	2.63580999879426
C	-5.85993999966455	-3.54590000148727	3.34896999913554
C	-6.92161999850014	-2.65480000001981	3.06965000141045
C	-6.77716000258500	-1.69605000062947	2.09030999699624
C	-5.56817000137405	-1.58425999623513	1.35374999894971
C	-5.39177000159996	-0.57661999855070	0.37926999897120
C	-4.18919999642813	-0.37806000323751	-0.28381999893434
C	-4.05547001128318	0.70639999700770	-1.28257001080186

158
3b_2 (TS1-1)

C	-4.00390999746516	-1.39393000034737	-2.33904000187969
F	6.86378999980812	-2.58448000068834	-0.43339000014811
F	7.53939999776564	-3.39770000247093	1.47914999935909
F	8.49614999856374	-1.61056999981979	0.65727000192986
H	4.60941000053520	-2.57628999921011	0.87884999852091
C	-2.02294000444408	-6.03531999902275	4.99041000305948
C	-6.25479999873253	-2.04693000243884	-1.79688999860239
C	7.30005999933274	-2.23405000012492	0.81056000242174
C	2.40809992347119	-0.73060992606646	-3.40031996287246
F	-6.13345999821167	0.93000999903296	-5.25013000134815
C	-1.40585000314524	-4.08844999764726	6.42482000056887
H	-1.47975999946529	-6.70111999959457	5.67646000233383
C	-4.88080999911668	-2.27413999995009	-1.70684999902011
H	-4.48932000132057	-3.13581000110172	-1.16656000069403
H	-2.44460000208930	-3.94403999893786	6.75532000284346
C	4.94378999820950	-1.73008000067906	1.48003000023009
H	-0.89059000105226	-3.12092000259199	6.49546999943252
C	3.62342000471562	-0.95936999630793	-2.72525000604905
C	1.85375000245670	-1.70216999923834	-4.22477000298188
H	1.88125009309863	0.21390990275085	-3.28267004237347
C	-6.35169999744500	1.10661999775065	-3.91518000259387
F	-7.68210999965134	1.32598000048014	-3.75487000083557
C	6.29308000055149	-1.38248000236160	1.54252999824760
H	-3.77548999924355	0.36401000007466	-3.55353000058683
C	4.26686999944230	0.06791999967658	-1.87476000326540
C	4.25038000951641	-2.19873000284249	-2.92941000181195
C	2.48169000634291	-2.94065000544915	-4.44014001012868
H	0.89257999994353	-1.48705000462206	-4.69424999211014
H	-1.17135550627595	3.39906832414403	-2.15176450323463
F	-8.47291000148820	-2.59752999764442	-1.10938000111175
H	0.50306594539001	3.32399965706123	2.32592328335897
C	3.51923000670734	0.99815999654672	-1.09713998776897
C	5.64694999901646	0.19579000535818	-1.83470000682861
C	3.69720000110231	-3.15939000143354	-3.77735000171630
H	5.17652999942916	-2.42872999901915	-2.40067000053505
C	1.82098000434606	-3.98249000006033	-5.34584000539779
C	-1.97639009372792	3.58384019080864	-1.45721006511635
O	2.12787000596886	0.87162000262644	-1.11043001063994

C	4.08384996340705	2.05128996521888	-0.38811001393096
H	6.26096000234694	-0.46879000205992	-2.44406999315222
C	6.29437999838519	1.15961000850773	-1.02880001418139
H	4.22566000043659	-4.10435000131756	-3.90119999541486
C	1.60576999782593	-3.37026000025534	-6.74553000116418
C	0.45649999960318	-4.37949999813181	-4.74473000052149
C	2.67553999962392	-5.24831000232306	-5.49851999917546
C	-2.72058994259507	2.49514992256859	-0.92380992535802
C	-2.35520003728448	4.85129985871685	-1.04403992277440
P	1.34917996222492	0.46057998183460	0.24368000396250
C	5.51179000440837	2.10443000774321	-0.28544999578459
C	3.21600009750611	3.10086997100278	0.21079013242366
C	7.71064999761028	1.21148999990351	-0.93754000241606
H	2.56352000049428	-3.06864999964486	-7.19437000163217
H	1.12970999943256	-4.10731999756023	-7.40930999905461
H	0.95584000109401	-2.48579999790984	-6.70457999841634
H	-0.19849000019731	-3.50932999799485	-4.60317000094936
H	-0.05727000232187	-5.08817999996747	-5.41145999737309
H	0.58106999753240	-4.87039000173867	-3.76895999907115
H	2.82697000066478	-5.75976000168220	-4.53625000233630
H	2.16454000184055	-5.95599999813701	-6.16658999924894
H	3.66126999835466	-5.02729999808284	-5.93423999797333
O	-2.37450001862187	1.20710000264051	-1.33767001795536
C	-3.77314995944335	2.62554000061377	-0.02941995675382
H	-1.83196994465866	5.71948003570296	-1.44956999126963
C	-3.39687997917765	5.06336001630211	-0.11094005010595
O	2.02469002781132	1.39222996460261	1.37154011475425
N	-0.12938999440804	0.81610000330169	-0.05477000791323
N	1.76150998718542	-1.05275000193935	0.70703999494561
C	6.18380000273263	3.02938000877649	0.55404998876267
C	3.35935994769597	4.48337993691107	-0.14625002348079
C	2.18571999860574	2.75397030729043	1.06981971306282
H	8.29611000156477	0.49056999714596	-1.51125999796191
C	8.33376000220592	2.13120000002000	-0.12208999603330
P	-1.57372999261615	0.24778999841465	-0.30652999510783
C	-4.45638002221729	1.41406999908890	0.49964996451087
C	-4.11789001592652	3.94235000598829	0.42238997649335
C	-3.72143000306409	6.37258000504416	0.33542000313576
S	2.29907000263957	-1.48318000004612	2.19977000101207
H	1.47055001235546	-2.11963000094334	-0.08185000030498
C	7.56086000172418	3.03925999945009	0.63711999869396
H	5.59701000065835	3.72516999804837	1.15221000194380
C	4.30341992794799	4.94440998841856	-1.10060007151695
C	2.46773020745007	5.44097008457943	0.44423015107658
C	1.28289977211898	3.67492939802962	1.66781015122560
H	9.42217999753583	2.15272999989041	-0.05157000225870
O	-2.31995999996093	0.52586999285753	1.10271000228636
N	-1.78639000255938	-1.25865999906293	-0.78175000332517
C	-3.71251000550152	0.41013001196187	1.10770000535746
C	-5.87563000057781	1.24212000354255	0.41042000338917
C	-5.10352000351107	4.18926999937434	1.41392000471007
H	-3.17111000249760	7.21578999829224	-0.08759999968588
C	-4.69395000645959	6.57774000061028	1.28929999785428
O	2.29107999697346	-2.93934999923774	2.20418000105292
O	1.62492000059037	-0.76016000032425	3.26643000275603
H	8.05581999778270	3.74571000009034	1.30456000416538
H	4.96774001816810	4.22799000849088	-1.58012997846457
C	4.38192001730571	6.28016000797563	-1.43759998174177

C	2.58685000465469	6.81094997951518	0.08738001791858
C	1.46585999031116	5.01005028910271	1.34492992606654
H	7.77094999977835	-0.03638999781484	2.36383000143235
S	-2.23490999812371	-1.70811999739526	-2.25539999926988
C	-4.27802998277636	-0.71729998447614	1.76717999244186
C	-6.47621000098845	0.11398999466749	1.06203999545030
C	-6.70833999702404	2.11694999724194	-0.33344000144239
C	-5.38305000101542	5.47229000176938	1.83791000439730
H	-5.63992999983333	3.34840999691664	1.84993999749363
H	-4.92811000127575	7.58796000320800	1.62782999852750
H	5.11046001379481	6.60639000140278	-2.18134998788442
C	3.52308991113686	7.22637997511285	-0.83378009395283
H	1.90728997528193	7.52867999519919	0.55177997984390
H	0.81228999566305	5.75777995473667	1.79833996116179
H	-2.00252999698898	-6.49959999931105	3.99328999953495
C	4.02273000037107	-0.98856999844225	2.22004000208160
O	-1.61409999781357	-0.93409999935678	-3.32872999818026
O	-2.11086999746751	-3.16802999818222	-2.31304999774246
C	-5.66110999799547	-0.81380000653794	1.74994999798884
C	-3.46066004040722	-1.72404000672501	2.48251005314218
C	-7.88411000003543	-0.05688999940344	0.98959999979154
C	-8.07057999868092	1.90945000277974	-0.40339000085610
H	-6.25611999924342	2.95038000005573	-0.86923000079151
H	-6.14033999737881	5.63291999746908	2.60681000033360
H	3.59821003559981	8.28041000943100	-1.10446995718026
H	-3.06826999882031	-5.98747999993096	5.32867000207689
C	0.10996000049046	-4.81721000044912	4.55724999751371
H	0.64956999756843	-5.43953999742828	5.28720999921814
H	3.68349000157443	0.63296999929509	3.58965000201824
H	-6.14426999961507	-1.63621000082668	2.27916000508958
C	-2.30038009362835	-1.38535022808244	3.20475000060357
C	-3.87696998593129	-3.06367997843932	2.52458998804067
H	-8.33051000229118	-0.91681000102761	1.49252000082418
C	-8.66787999920079	0.82162999895292	0.27332000244107
H	-8.68886999826642	2.58382000048539	-0.99729000191416
F	5.43917000223774	2.65594000128646	3.78957000176398
H	0.16900000099895	-5.31849000298715	3.58070999869869
H	-0.91498000145626	-4.78612999969895	7.12010000276513
H	0.63240999667787	-3.85485999801229	4.47593999922049
C	-1.62777003372147	-2.33689001352801	3.96356003911213
H	-1.92425979970650	-0.36430966567484	3.19272993077215
C	-3.20461997789057	-4.00820000099377	3.30097997140507
H	-4.74490998953283	-3.37567000168215	1.94064999312608
H	-9.74660000169724	0.67027000187088	0.21374999870489
C	6.20747999904975	1.54511999794779	3.96761999807383
F	-7.22610000207969	-4.20689000232351	-1.90949999874492
C	-2.07554998564237	-3.66526998074762	4.05711998272179
H	-0.73252998532843	-2.02173998422212	4.50258000769731
H	-3.58359001392139	-5.03002000009359	3.31527001494786
F	6.08145000152816	1.19190000048599	5.27919000057885
C	5.77966999762390	0.41818999894603	3.06059000097400
F	7.50076000195619	1.90810000204546	3.77548999928557
H	-7.82208999740651	-0.76305000189472	-2.54852999788277
F	-6.81770999950120	-3.40299000203011	0.08056000191600
F	-5.70509000037739	2.25569999989111	-3.56657000118129
C	-1.36057998533424	-4.65086999775546	4.98782998005855
C	6.71803000025968	-0.30823000198421	2.32911999839288
C	4.42436000117555	0.08126999802484	3.01262999857574

C	-5.85689999796567	-0.07366000155701	-3.11862999837043
C	-4.47965999832441	-0.29798999974960	-3.05210999962553
C	-7.19638999860712	-3.05216000160595	-1.18458999968444
C	-6.75082000029010	-0.94445000147304	-2.49639999966639

158
3b_2 (TS1-1′)

C	-3.90652000175618	1.38616000101102	-3.18522000205713
C	-6.25928000229378	0.19539000036243	-2.27747000087781
F	-4.03006999970638	4.24575999991357	-3.12713999836285
H	-5.07235000254065	-1.59459000224319	-2.00435999798327
H	-6.82006000163494	-6.87530000076802	2.56350999915987
C	-4.79483000064584	-5.59785000064402	3.93204999745498
H	5.08231999739615	-3.22116999955159	-0.34347000256289
C	-5.08236999927680	-0.55159000104286	-2.31888000136166
C	4.08321998878670	-1.92355999137354	-3.26974999692563
C	-4.35302000234566	-6.27206000235856	1.57077000037064
C	-5.07880000053279	3.54261000037828	-3.64020999996234
C	5.40083999828935	-2.62742000053541	0.51319999906458
F	7.34327999788461	-2.58014999773156	-1.59612000170654
C	7.75920000104610	-2.76097999944047	-0.30767000001652
F	8.95648999949682	-2.13490999975237	-0.18149999815917
C	-3.90969000094343	0.06064999993752	-2.76297999847459
F	7.98870000211352	-4.09664000042145	-0.16180000074798
C	3.48302999638045	-0.76144998161104	-2.75979999008561
C	3.47794999987969	-2.66679999657320	-4.28399999986669
H	5.03823999965493	-2.25956999969625	-2.86222000479749
C	6.73910000054002	-2.27073999936749	0.68870000090794
C	-6.70089999954074	-5.81293999845176	2.30131000042905
F	-8.40663000042321	0.42340999859049	-1.27140999809442
F	-6.21404000251730	4.21741999908996	-3.33116000093332
C	4.19491000107824	0.04851000338938	-1.74698999526739
C	2.23232008267435	-0.39329017150526	-3.28750008423358
C	2.24661999899357	-2.28414998925111	-4.83324999256767
H	3.98699000222689	-3.55980000076825	-4.64607000232659
H	0.47568937832622	3.35665745967665	-1.44333825706061
H	8.18478000189905	-1.25841999924867	1.93380000117911
C	-5.09429000166881	2.12021999795901	-3.14039999951144
C	3.52788000059605	0.71427000564481	-0.68197000937747
C	5.57281000335063	0.18945999732197	-1.79557999939134
C	1.63722000058433	-1.13703998911834	-4.29920999077991
H	1.72657988512914	0.50055023976013	-2.93365988273830
C	1.56144999874874	-3.04417999681911	-5.97197999926820
C	-0.19587979710135	3.64597949853738	-0.64962021277913
O	2.13007000812685	0.62617999899299	-0.63817999388219
C	4.16705995941975	1.45026999580677	0.30792996286801
H	6.12395000121329	-0.25828999687829	-2.62352000331548
C	6.29849000358513	0.87567999321483	-0.79579999635214
H	0.66401999524352	-0.80757999537011	-4.66437000431782
C	2.38396999946509	-4.24942000095379	-6.44640000009552
C	1.36609999935777	-2.08457999757754	-7.16469999888810
C	0.18449999931496	-3.54756999936606	-5.49516999998585
C	-1.31615007761488	2.82643025695016	-0.32581971386982
C	-0.00144014736055	4.76508030652956	0.14459008089840
P	1.50029999629262	-0.35086002160890	0.48107002582271
C	5.60014999737592	1.48906000341296	0.29724999944918
C	3.36525013128410	2.16518006901696	1.33683006998669
C	7.71678999873774	0.93778999818147	-0.83341000093833
H	2.53340000116242	-4.98322999910828	-5.64041999999774

H	1.85369999761641	-4.75767999894689	-7.26451999797873
H	3.37130999866538	-3.94669999875582	-6.82495000156960
H	0.87952000023724	-2.61169999797438	-7.99917000098723
H	0.73482999754657	-1.22787000144668	-6.89262000242396
H	2.33290000117167	-1.69693000126361	-7.51770000201806
H	0.30356000253093	-4.27188000201066	-4.67592999959844
H	-0.45484999853280	-2.73153000188881	-5.13234000218393
H	-0.34198999986007	-4.05324000284034	-6.31883000174335
O	-1.47088998322398	1.62715996475256	-1.03258012307929
C	-2.28753003807026	3.16926997672220	0.60167984686511
H	0.83562002976676	5.43146993503438	-0.07040999729434
C	-0.86288009110690	5.08933012097409	1.21802987816098
O	2.23271003555509	0.11341009518095	1.83954991085921
N	-0.03200000130246	-0.10731998929340	0.49277000156151
N	2.03608999014368	-1.87564000654343	0.24308000374698
C	6.36228999873271	2.07526000079560	1.34021000170700
C	3.51361000344428	3.57588000151464	1.55943999706623
C	2.39355986454524	1.49062978189072	2.06565015222322
H	8.23546000142731	0.47306999992905	-1.67405999983607
C	8.42629999876770	1.54030999854626	0.18273999832992
P	-1.38527999540977	0.21389998763606	-0.24379999398237
C	-3.56438997932627	2.41321997683092	0.69319003247645
C	-2.03848991245320	4.30094990246409	1.44731008374274
C	-0.59653995132637	6.19774993955983	2.06473004451023
S	2.78232000027558	-2.84892000113618	1.35202999661252
H	1.32232000002801	-2.60768999834058	-0.64105000382194
C	7.74077000292329	2.09929999820802	1.28491999840500
H	5.84664999988832	2.49751000176729	2.20116000131796
C	4.37658999666516	4.39742999476781	0.78573000597126
C	2.71270002756897	4.20204001613478	2.57137988329565
C	1.54784019406119	2.09509011499453	3.03545990634493
H	9.51623000117342	1.57034000257313	0.14768000182295
O	-2.37639999634388	0.36348999940930	1.03169001326845
N	-1.89255000551230	-0.91140999830711	-1.24305999602893
C	-3.59185000129609	1.04368000472600	0.92564999892022
C	-4.81134999902790	3.10789000677620	0.53388001062280
C	-2.87588998039438	4.63825998489114	2.54261002260914
H	0.29611000607733	6.79473999618931	1.86909000253231
C	-1.43085997546603	6.50046996795925	3.11890002632621
O	2.85941999966931	-4.15070999876582	0.70470999786590
O	2.20164000176912	-2.71711999884251	2.67825000115115
H	8.30517999839045	2.54144000303415	2.10681000149220
H	4.95913998683749	3.94860000126984	-0.01712000946748
C	4.47648000793377	5.75227999868802	1.02626000855805
C	2.86109002759692	5.59409999451435	2.81325004772276
C	1.75867964978147	3.44213995855283	3.28378004040211
H	0.80116041099009	1.50675501096177	3.54619517238450
S	-2.36599999793069	-0.85883999723792	-2.76903999895192
C	-4.79429999725154	0.32097000186168	1.19003999998969
C	-6.03216999812731	2.40101999774106	0.78378999968881
C	-4.89193000257231	4.45439999886537	0.09310999803138
C	-2.57434002204685	5.70547002506244	3.36354998100945
H	-3.75588001383383	4.02842001591020	2.74362998286295
H	-1.21062001858394	7.34926002368903	3.76812998007273
H	5.14048999909665	6.36127999905905	0.41116000221823
C	3.72570000122444	6.35731000021155	2.06018999883635
H	2.25711000335941	6.04812000461583	3.60121000508524
H	1.14972012413596	3.94342998943093	4.03791000263319

H	4.10501999780524	-1.25498999959501	3.35019999791135
H	-5.41358999929602	-5.01659000203082	4.62986000084524
O	-1.45409999877014	-0.12194999901179	-3.63945000135092
O	-2.70739999651056	-2.23646000039643	-3.14521999963729
C	-5.98048000026735	1.03599000097897	1.14254000104708
C	-4.84004999768227	-1.12203999925346	1.52262999868088
C	-7.27250000053152	3.07392000072332	0.62583000039870
C	-6.11285000126399	5.07650000023605	-0.06595000166721
H	-3.97601999841635	4.99293000236633	-0.14453000019253
H	-3.22307999807043	5.93597999811774	4.20981000741828
H	3.82545998863555	7.42685000402815	2.25085998970627
H	-3.28731000141904	-6.02001999962379	1.66762999855959
C	4.46954999838731	-2.24695000131471	1.47792000139940
H	-3.74749000019977	-5.29552000174346	4.07263999892190
H	-4.88678000172791	-6.66056000076918	4.20320000205858
H	-6.91792000205319	0.50730999719753	1.32329999789797
C	-5.66540000037268	-1.57104000250477	2.56929000070640
C	-4.15701999812615	-2.09055000039491	0.77818999993246
H	-8.19273000049699	2.51891999841216	0.81793000179451
C	-7.31501000167212	4.38749000261068	0.21215000078918
H	-6.14995999754226	6.10532000015429	-0.42639999781681
F	6.55660000017035	-1.24716000250712	5.09594999883477
H	-7.36840000255069	-5.22688999887785	2.94872000232477
H	-4.62904999988340	-6.18226000067983	0.51042999972356
H	-4.47955999975503	-7.32461000117395	1.86413000104158
C	-5.78232000070196	-2.92722999953565	2.85674000205170
H	-6.20283000262014	-0.84316999774361	3.18029000146940
C	-4.28967999889236	-3.44937999909766	1.06069999967685
H	-3.51264000145063	-1.79052000154414	-0.04495000028850
H	-8.27339000047715	4.89252999886285	0.08353999905032
C	6.59883999919074	-0.42512999936044	4.00832999770614
H	-7.03166000166079	-5.67497000093234	1.26183000129651
C	-5.09410000080293	-3.90112999853493	2.11275000016068
H	-6.41834999915121	-3.22961000028583	3.69091999885219
H	-3.73735000180568	-4.15380999880264	0.43924000158544
F	7.86018999968903	0.07195999866727	3.93518000120008
C	6.19108999983721	-1.16013000015997	2.75599000117806
F	5.76353999897075	0.61724000031815	4.27739000134174
F	-8.20007000054344	-1.01836000220681	-2.90388999835779
H	-7.19068999792437	2.11568000161272	-2.62885000145609
F	-4.94538000098139	3.58475000073822	-4.99834999923757
C	-5.22899999777067	-5.38436999803985	2.46720999742924
C	7.14099000263807	-1.53709999980180	1.80670000204657
C	4.85019999750588	-1.51757000038486	2.60100999925520
C	-6.27224000199381	1.53382000064753	-2.68162999867398
F	-7.33308000099669	-1.45878000016268	-0.94457999913306
H	-2.98168000056002	1.83159000011360	-3.54799000228947
C	-7.54418000261860	-0.46458000141888	-1.84292999786460

158

3b_2 (TS1-2')

C	6.06019999894810	2.87505000115963	2.55422999155941
F	6.02401000006571	2.99092000190782	1.19201000240159
H	-6.32252000196920	0.30772000216465	5.93471999921449
C	-3.63898000169679	-1.93033000088456	-3.34545999914363
F	6.45429000271960	4.08248999642201	3.03888000083302
H	-4.61538999933990	2.78572000223638	5.31525999968578
H	5.05008999725560	0.34114999997391	2.69754999435163
H	-2.72239000010899	-2.41825999967756	-3.67279999752652

C	1.60212996768540	-4.26885984608892	-0.07600010174136
H	-6.15145999800704	-0.54849999763496	4.38650000188790
H	-5.95190000186085	3.78803000155010	4.70552999738393
F	7.03782000080104	1.97916000053447	2.84580999842301
H	-8.19398000167603	1.65758000262129	4.97712000229865
C	4.36626000967158	1.10085999811010	3.07617002229445
C	-5.18576000205224	-1.39121999877934	-1.55406000244102
C	-4.00434000169836	-1.98215999764148	-2.00340999795396
C	-4.45383999772291	-1.24380999863505	-4.24815999845827
C	2.70252000875507	-4.14102003419911	0.78670001430380
C	0.53256000254646	-5.10449002989307	0.24002001098616
H	1.57604002181541	-3.72214016435141	-1.01546985540441
H	-7.94922000090145	2.66372000134813	3.52798000213978
H	-4.73419999829314	0.22090999986846	5.13442000183281
H	1.23228999954181	1.34286000237421	4.41832999592355
H	-6.20611000158532	2.74406000261947	6.11894000005908
C	3.85529999328486	-3.25812999513241	0.50524000473740
C	2.67925000832003	-4.87875001487269	1.98252999789448
C	0.51044000363022	-5.85471001709926	1.42542999641131
H	-0.30363000153470	-5.14351998789789	-0.45731999785782
H	1.48207752872803	-1.16102578803036	-3.47970983095009
H	-8.00479000023399	0.89365000021942	3.37620999968918
C	3.10375000403583	0.72735999817779	3.53298000960570
C	3.69308999797595	-1.95345000022088	-0.03118998999781
C	5.14636999875566	-3.62618999987976	0.84130000066074
C	1.61191999990123	-5.71942999800690	2.28694999926069
H	3.48954999828332	-4.75380999442494	2.70228999875841
C	-0.63012000057907	-6.82734999939191	1.74463000407919
C	1.14041990224555	-0.14310977917984	-3.58692053395116
O	2.39432999815312	-1.56215000084551	-0.39035999891484
C	4.71214998531532	-1.02994001628306	-0.19509003154313
H	5.32995000071917	-4.63145999930555	1.22499000231273
C	6.23094999933409	-2.72164999893887	0.76692999534534
H	1.63990000000073	-6.26806999708892	3.22904000221868
C	-0.94767000307476	-6.83759999737733	3.25176999981889
C	-0.17751999861776	-8.24200999743865	1.32223999867641
C	-1.91752999864082	-6.46887000165721	0.98501000036283
C	-0.12970995900237	0.24903982436754	-3.07198968967710
C	1.89623016247622	0.86082992305976	-4.17627944319277
P	1.65707998579537	-0.56989998720377	0.63554998952047
C	6.01843999473999	-1.38760999643137	0.28181000812665
C	4.44576002078157	0.29102000377876	-0.82132002423273
C	7.52503000036276	-3.09441000211488	1.21731000023185
H	-0.10650999939178	-7.20514999713243	3.85496000119954
H	-1.20709000113877	-5.83038999801038	3.60925000093349
H	-1.80524999779231	-7.49798000139412	3.44677000159600
H	0.04112000069372	-8.27428000246814	0.24517000189752
H	0.73147999890137	-8.54368999794411	1.86260000119511
H	-0.96822000113627	-8.97764000024616	1.53631000188665
H	-2.24874000231390	-5.44161999712557	1.19210999912106
H	-1.79848000122770	-6.56055000060764	-0.10276999759084
H	-2.72419999749640	-7.15357000015554	1.28507999973149
O	-0.88408998228650	-0.70436991868963	-2.36141000521720
C	-0.67990001609329	1.51096004166054	-3.23582014187860
H	2.86616996013717	0.61103000294582	-4.60904013633161
C	1.46303999326181	2.20431000413156	-4.23728000616195
O	2.62033004869726	0.72451996936533	0.69832999068932
N	0.25153999633953	-0.27336000126743	0.05356999934679

N	1.56952999431754	-1.07840999539501	2.17240999931150
C	7.09579000004543	-0.46666000183763	0.34442999480289
C	5.23803995944986	0.74008997889550	-1.93346004263675
C	3.43599002102345	1.13158009028643	-0.36493982977144
H	7.67661999760789	-4.11592000040935	1.57216000114142
C	8.55843999749726	-2.18339999920183	1.23905000380785
P	-1.07005001085297	-0.46158000689697	-0.77126002026846
C	-2.09489000285644	1.78542999003549	-2.87338999950912
C	0.15452995923377	2.54927000032799	-3.76622007247137
C	2.30302994973662	3.22458998924921	-4.75723012245745
S	2.63693999710375	-1.00262000188536	3.43171999649488
H	0.41644000055126	-1.73469000233306	2.51310000184873
C	8.33318999898990	-0.85390999787328	0.81673000397095
H	6.93528999950959	0.56361000331079	0.03037000109901
C	6.14672999985364	-0.11036999729471	-2.61729999736382
C	5.07403004256932	2.08031998774768	-2.41291997986196
C	3.25253984424538	2.46694987050156	-0.82617032005105
H	9.54398999768834	-2.47935000023194	1.60123000013557
O	-1.69606999905599	1.03548999111469	-0.63842000272938
N	-2.07137999584595	-1.53579000467954	-0.18674999786559
C	-2.56671999917612	1.57135000257748	-1.58575999508682
C	-3.00095999936886	2.30276000181140	-3.85850000019042
C	-0.23468998405506	3.91317999831593	-3.78198996309158
H	3.29186000137684	2.94396000052014	-5.12548999324707
C	1.89159001197772	4.53925000370515	-4.76921997765920
O	3.86045999994826	-1.73784999846520	3.16024000122531
O	1.83602999861363	-1.33215000056508	4.60200000079420
H	9.14123000154975	-0.12309000029690	0.87166999991331
H	6.25755000901131	-1.14208999637146	-2.28634999470003
C	6.87300000097420	0.34579000068361	-3.69801999946611
C	5.85463995906460	2.52716998212009	-3.51195004470686
C	4.10729012353229	2.91918013467265	-1.81502976426354
H	2.46852891685362	3.07748464941809	-0.40416759329426
S	-2.88109999672931	-2.75305999994801	-0.83217999676080
C	-3.84554999929749	2.01295999695878	-1.13628000007406
C	-4.31055000275726	2.71096999918323	-3.44319000213835
C	-2.67154000252510	2.39074999611990	-5.23568999893712
C	0.61768999664949	4.88526999735629	-4.26192000659711
H	-1.21027998648182	4.19184999579497	-3.38628997653561
H	2.54996000697925	5.31728999934404	-5.15902998134721
H	7.55444999917699	-0.33170000049060	-4.21452000191709
C	6.73898999598992	1.68049999964915	-4.14395000194455
H	5.72657000057831	3.55631999732389	-3.85420999931873
H	3.97709996887199	3.93243996221375	-2.19815005225821
C	4.72188000215060	2.44968000561721	3.10455000497089
H	-5.46450000051504	-1.45338000080197	-0.50292000144839
O	-2.04021999861344	-3.66406000076103	-1.60098000215947
O	-3.70741999865714	-3.31200000180614	0.24529999933034
C	-4.67609999778369	2.59114999790790	-2.08377999833265
C	-4.30456000227845	1.91858000170086	0.26873999912909
C	-5.22395999744742	3.20724999847925	-4.41041000085143
C	-3.58403000198462	2.87013000118496	-6.15233999829996
H	-1.69207000018207	2.05302000151786	-5.57091000175720
H	0.30695000041731	5.93097000010748	-4.24618000653324
H	7.32714000925451	2.03224000572564	-4.99270999095945
C	-5.81417000209512	0.32660999861870	4.95941999836013
H	4.11850004924153	4.45571003140977	3.62677011445411
H	-6.24343999810000	-0.06932999849012	-4.51897000135578

C	-5.98874999766694	-0.70931999933503	-2.46873000076256
H	-5.67264000118754	2.91600999990967	-1.78023999823863
C	-4.17641999977638	0.75688000252805	1.04052999784615
C	-4.97855999866360	3.01027000120229	0.84630999790447
H	-6.21966000163606	3.50848000296362	-4.07860000002317
C	-4.86932000038130	3.28845999980693	-5.73939999825225
H	-3.31560000015395	2.91124000010562	-7.20879000160679
F	2.27609000243528	5.17061999758683	5.02261000489050
C	-5.70016000025607	2.81259000157935	5.14402999752077
C	-7.66298000016105	1.72068999926774	4.01486999825369
F	-2.73639999857570	-0.89841999851699	-5.85317000250621
C	-4.74098999792324	0.66816999960029	2.31311000127739
H	-3.64413999801489	-0.10630000103767	0.64591999759638
C	-5.53092000040279	2.91962999886180	2.11955999780642
H	-5.06285999753568	3.94467000078059	0.28761999981316
H	-5.58127000056442	3.66349999847752	-6.47598000077605
C	1.63445999856043	4.03715000100424	4.63116999343474
F	-4.77460000050731	-0.32185000198377	-6.42296000044569
C	-5.44405000032057	1.73914000072466	2.87736000069409
H	-4.61832000079544	-0.26825000251905	2.85699999748830
H	-6.04998000253844	3.79020999983489	2.52479999832277
F	0.71092000158706	4.41117000056601	3.69886000142376
C	2.58796000416522	3.00461000544481	4.08443000216600
F	0.94526999599140	3.57103999712819	5.70631000215282
F	-8.31941999837395	-0.98159000223118	-2.11017999825931
C	-4.05310999967204	-1.21521999872262	-5.70148000105225
F	-4.22781999812368	-2.43679000215244	-6.28512000061254
C	-6.13794999812640	1.64381999811898	4.23942999787709
C	3.83356995944869	3.40565998449536	3.59830990164858
C	2.20700001192853	1.66123999644485	4.04827002435791
F	-7.63071999762145	0.99170000150441	-2.75781000026090
C	-7.28704000165039	-0.09462000180268	-2.00844999803245
F	-7.23540000033178	0.30692999878454	-0.71417999982150
C	-5.62474999907293	-0.62386999836495	-3.81563000085972

142

3b_3 (cat)

H	0.83586000238212	-5.83991000206209	1.29773999851010
H	-5.37322999755856	2.05755999781377	-0.22900999891625
C	-3.83478999946120	3.87792000035870	-4.30792000182628
C	-2.36741999793564	4.33714999905846	-4.43634000166718
H	-5.28610999796971	3.82625000002599	-1.90383999849177
C	-4.77976999831995	1.89882999740375	-1.13001000077668
H	1.17062999763714	-3.39937000143240	1.78822000294882
C	-3.26420000176072	0.57658999918237	-2.43855999927845
C	-4.72109999950110	2.91275000178443	-2.08504000088907
C	-3.93565000049872	2.78637000118879	-3.23810999918243
C	-3.21494999771065	1.59119000155829	-3.38689999836841
C	0.21410999840857	-3.80057999947133	1.49077999189209
C	0.00385999809966	-5.13986000220092	1.19958000339127
C	-1.25097999986604	-5.63645000156822	0.77498000093158
C	-1.41871999939660	-7.00242999884724	0.42255000051203
C	-2.62642999997451	-7.47151000154142	-0.04642000188851
C	-3.72117000105195	-6.58703999816880	-0.18108000120518
C	-3.59717000104520	-5.25799999892351	0.16882999789113
C	-2.37045999994433	-4.74485000253410	0.66550000035732
C	-2.18320000728067	-3.36683000114652	1.00670000769152
C	-0.91757998732923	-2.94597000215749	1.37970999722010
O	-0.75090007905516	-1.58188001594331	1.64663000844926

P	-0.68401969035867	-0.59112993211060	0.36667994855252
N	-0.93474039684167	0.90380993229863	0.82917004497678
S	-0.65495956696570	1.46619991523555	2.31422002761849
O	-0.73287003761643	2.92171007096277	2.24803999065169
O	0.51071982495103	0.85800001476232	2.95700999097739
H	0.62355001090274	5.02841001419696	1.82892999956492
H	3.75503999908567	0.62660999869675	1.91117000244686
C	-4.68084000171046	-3.47850999810856	2.63580999935998
C	-6.92161999831353	-2.65479999976468	3.06965000160416
C	-5.85993999927609	-3.54590000085607	3.34896999966194
O	3.13487000117738	-0.46195999614629	-0.21738000095583
H	2.70195000136234	2.31523000150746	3.31196000192436
C	-4.05546999777297	0.70639999961591	-1.28257000091684
O	-1.88954001954769	-1.10327000677584	-0.58294000690144
C	-4.18919999966334	-0.37805999876371	-0.28381999707350
C	-5.56817000142246	-1.58425999762478	1.35374999791119
C	-5.39177000192639	-0.57661999829774	0.37926999944331
C	-4.50320000218054	-2.50726999865866	1.61734000175667
C	-3.13795000064378	-1.28838998703659	0.02786999335511
C	-6.77716000206116	-1.69605000022565	2.09030999749346
C	-3.28483999851892	-2.37625000347127	0.87928999943954
H	0.93234999658988	6.54613999452933	2.70753000104488
N	0.59803995910659	-0.97146001592974	-0.49359998774524
P	1.81168001112678	-0.24307999905507	-1.10149998606720
N	1.75215999733785	1.41528999959843	-1.30400000292593
S	0.95366999796456	2.16173999889022	-2.59887999756060
O	-0.13264999895458	1.26476999931535	-2.93608000198545
O	1.94282999838032	2.56891999976669	-3.57842999993912
C	0.33269000294931	3.65563999752871	-1.83731999731744
C	-0.82239000166870	3.63977000122885	-1.04691000047928
C	-1.28556000086220	4.85708000474989	-0.54988999961057
C	-0.61207000846867	6.05266000336399	-0.82378001670856
C	0.53838999724137	6.03751000004927	-1.60443000193725
C	1.01762000172091	4.83292000150888	-2.12357000100446
H	1.90292999939696	4.80440000108376	-2.75767000066516
C	1.30150000064544	7.30409999700895	-1.89816999898764
F	0.69568999835612	8.40450999997099	-1.39012999889035
F	2.56096000087654	7.25491000199014	-1.36740000159221
F	1.45234999969302	7.49898999926055	-3.23601999726832
H	-0.98632999990685	6.99271999960161	-0.42033999810359
C	-2.54069999023745	4.95645992740258	0.28421006192643
F	-2.30560999918462	5.58443001092159	1.46546999086124
F	-3.09592000584577	3.75818006749294	0.55029994716211
F	-3.48715000901547	5.70547000533321	-0.36817000358548
H	-1.33501000472988	2.70473000302176	-0.81918000031610
H	2.56957999995624	1.93409000131549	-0.94894000069978
O	2.20455000018593	-0.86058000290688	-2.53797999748221
C	2.71400999685499	-2.18009000105700	-2.46265999954382
C	4.04334000204213	-2.32257000141203	-2.08783999941063
C	4.57381000066574	-3.64340000193340	-1.93365000064023
C	5.88490000233636	-3.89940999794120	-1.45339999839513
C	6.34146000041113	-5.19102999842868	-1.29079000189226
C	5.51136000240757	-6.29221999769132	-1.60361000229024
C	4.22883999778178	-6.07785999983445	-2.05821000199808
C	3.72150000134214	-4.76054000021224	-2.22047000076027
C	2.38508000135847	-4.53627000018251	-2.62187000084605
C	1.83752999860680	-3.26588999799842	-2.73055999866832
H	0.80877999860674	-3.08367999754520	-2.99968000050904

H	-4.38849000026949	5.57072000073170	-3.02654000083099
C	-4.69626999965917	5.10302000209392	-3.97289000052444
H	-3.69272000041988	2.44639999783194	-5.97093999889107
C	-4.30711000073258	3.30215999973619	-5.65895000239406
H	-2.27214000107359	5.08282000097705	-5.24002999820313
H	-1.69662999967953	3.49859999884683	-4.66889000026141
H	-5.35117999826716	2.96319999997213	-5.59471000068463
H	-4.67200000129442	-6.95709000051501	-0.56760999932414
H	-3.86202999944509	-4.15828000105500	2.86885000041046
H	-7.84941999994493	-2.71999000164262	3.63982999775249
H	-7.58139999905774	-0.98772999781338	1.88250999755112
H	-0.56222999778892	-7.67307999806380	0.52027000228404
H	-2.73901000112959	-8.52143000039818	-0.32035999740267
H	-5.96823999920041	-4.28470999826272	4.14412000198347
H	-2.02133000210594	4.79850999956599	-3.50070999776009
H	-4.44671000145369	-4.58554999802067	0.05819999937845
H	-6.23703000045383	0.07337999789377	0.14973999921063
H	-5.76228999784445	4.84271999827327	-3.89926999931658
H	-2.57613000251254	1.44568999763238	-4.25899000152819
H	-2.67631000022471	-0.32323000089319	-2.60376000078139
H	-4.59044999944309	5.85510999774483	-4.76807999804679
H	-4.24092999834104	4.07101000080139	-6.44361999944798
H	1.75480000021392	-5.39903000054823	-2.84527999819249
H	3.57260999822655	-6.91953999857081	-2.28938000179003
H	5.88614000217182	-7.30862000164869	-1.47566000172911
H	7.35029999886772	-5.36348999893700	-0.91294000174783
H	6.53259000255370	-3.06034999791520	-1.20374999804162
C	4.84322000191513	-1.10947999940336	-1.78278999783275
C	4.36894000206020	-0.19738999921243	-0.85309000141820
C	5.02715999932950	1.01551999814534	-0.51570000148301
C	6.24496000148652	1.25895000193728	-1.12899000113187
C	6.78779999904750	0.38108000255996	-2.09557999912689
C	8.01576000253172	0.67436999844453	-2.74621999814083
C	8.49931999759858	-0.14132999761862	-3.74512000069365
C	7.76275000208117	-1.28032999945405	-4.14386000142238
C	6.57636000105158	-1.60423000223336	-3.51933999888647
C	6.06735000073301	-0.80503000027867	-2.46268000244997
H	6.01408000134646	-2.48043999914503	-3.83953999986234
H	8.13314000061956	-1.90620000186906	-4.95699000074088
H	9.43319000038435	0.09568000187055	-4.23744000059188
H	8.56146000186815	1.57172000040147	-2.44752000140146
H	6.79534000169046	2.16644000227665	-0.87297999970165
C	4.39843000046743	2.01494999809581	0.37809999930602
C	3.78358999853527	1.66780000205497	1.59157999993522
C	3.18858999843896	2.63857000249809	2.39446000084328
C	3.16945000224333	3.99032000143961	2.02519000040284
C	3.76858999917773	4.33097999912430	0.80008999860126
C	4.37343000219941	3.37070000012735	-0.00672000017106
H	4.81604000018153	3.66402000029410	-0.96133999904620
H	3.75809000153103	5.36763000087174	0.45896999799432
C	2.52661000012540	5.07371999842102	2.89489000148145
C	1.92416000104453	4.49951000152758	4.18461999780860
H	1.12889000277917	3.77332000206287	3.96824999973484
H	1.48180999839254	5.31670999827748	4.77237000005832
H	2.68826999804008	4.01532000062366	4.81062999954441
C	3.60036999929092	6.11633999876761	3.26808999924231
H	4.03366999740177	6.59202000040413	2.37694999766554
H	4.41849000251877	5.65146999776659	3.83745999960570

```
  H   3.15472000046660      6.90726000210766      3.88970999965876
  C   1.39860999951556      5.75801999611441      2.09723000012477
  H   1.78179999945850      6.22556999773723      1.17979000164113
  H   -1.73750191251085      1.04910649245866      3.01213249515000
142
3b_3 (TS1-1)
  C   -4.00390999748598     -1.39392999988061     -2.33904000202004
  C   -6.75082000026197     -0.94445000154589     -2.49639999966577
  C   -5.85689999799743     -0.07366000157793     -3.11862999842278
  C   -7.19638999851149     -3.05216000165714     -1.18458999952417
  H   -0.73252991553918     -2.02173994814966      4.50257995686790
  C   -2.02294000284768     -6.03531999981806      4.99041000242051
  C   -6.25479999869711     -2.04693000253021     -1.79688999860552
  C   -4.47965999835009     -0.29798999961334     -3.05210999968325
  C   2.40809999834243     -0.73060999988145     -3.40032000096212
  F   -6.13345999818042      0.93000999904247     -5.25013000130587
  C   -1.40585000224214     -4.08844999879387      6.42482000013742
  H   -1.47975999779403     -6.70111999979861      5.67645999983034
  C   -4.88080999909418     -2.27413999995042     -1.70684999923242
  H   -4.48932000126985     -3.13581000111675     -1.16656000069039
  H   -2.44460000180057     -3.94403999953822      6.75532000165131
  H   -7.82208999738846     -0.76305000185132     -2.54852999784145
  H   -0.89059000158045     -3.12092000209050      6.49546999950485
  C   3.62342000051868     -0.95936999996777     -2.72525000145177
  C   1.85374999967741     -1.70216999834080     -4.22477000065400
  H   1.88125000014278      0.21391000126354     -3.28267000088899
  C   -6.35169999745051      1.10661999771612     -3.91518000261547
  F   -7.68210999963154      1.32598000046835     -3.75487000081580
  F   -5.70509000034244      2.25569999987124     -3.56657000132493
  H   -3.77548999924612      0.36401000000039     -3.55353000066445
  C   4.26686999715465      0.06792000038182     -1.87476000167773
  C   4.25038000268406     -2.19872999868858     -2.92940999779513
  C   2.48169000032365     -2.94065000230391     -4.44014000210191
  H   0.89257999874361     -1.48705000143911     -4.69424999783602
  H   -1.17135999774215      3.39906999994717     -2.15176000191221
  F   -8.47291000139368     -2.59752999761239     -1.10938000094815
  H   0.50307000123250      3.32400000126538      2.32592000042777
  C   3.51922999554688      0.99816000509895     -1.09713999141552
  C   5.64694999775280      0.19579000104231     -1.83470000143412
  C   3.69719999886735     -3.15938999987766     -3.77734999900272
  H   5.17652999779941     -2.42872999834575     -2.40067000091146
  C   1.82098000244275     -3.98248999951389     -5.34584000232127
  C   -1.97638999785664      3.58384000115345     -1.45720999998426
  O   2.12786999730979      0.87161999655616     -1.11042996946194
  C   4.08384999597831      2.05128999763285     -0.38811000013220
  H   6.26096000185167     -0.46878999746364     -2.44406999870513
  C   6.29437999790648      1.15960999938629     -1.02880000196626
  H   4.22566000272449     -4.10435000120861     -3.90119999808385
  C   1.60576999785604     -3.37025999996224     -6.74553000101876
  C   0.45649999978503     -4.37949999805963     -4.74473000027638
  C   2.67553999980502     -5.24831000221744     -5.49851999909142
  C   -2.72058999888883      2.49514999827454     -0.92380999970312
  C   -2.35519999958250      4.85129999816939     -1.04403999833885
  P   1.34918042488241      0.46058007164813      0.24367985445399
  C   5.51178999941506      2.10443000085517     -0.28544999824887
  C   3.21599999750374      3.10086999938187      0.21078999754675
  C   7.71064999753236      1.21148999795415     -0.93754000015350
  H   2.56352000058591     -3.06864999931547     -7.19437000197046
```

H	1.12970999964314	-4.10731999762474	-7.40930999927371
H	0.95584000105548	-2.48579999798613	-6.70457999835639
H	-0.19848999990268	-3.50932999862882	-4.60317000174599
H	-0.05727000237373	-5.08818000034396	-5.41145999827576
H	0.58106999723800	-4.87039000323865	-3.76896000092815
H	2.82697000039692	-5.75976000157928	-4.53625000275671
H	2.16454000222056	-5.95599999810530	-6.16659000019122
H	3.66126999879317	-5.02729999839869	-5.93423999843799
O	-2.37449999785005	1.20710000062053	-1.33767000420438
C	-3.77314999893944	2.62553999821136	-0.02941999822247
H	-1.83197000218311	5.71948000081124	-1.44956999879461
C	-3.39688000010981	5.06336000135711	-0.11094000139989
O	2.02468993944049	1.39222999521957	1.37154003193487
N	-0.12939003146625	0.81609998139651	-0.05476998035758
N	1.76150962502469	-1.05275010751635	0.70704006259710
C	6.18380000104409	3.02938000246759	0.55404999721044
C	3.35936000184523	4.48337999732015	-0.14625000219015
C	2.18571999383740	2.75396999853992	1.06982000271711
H	8.29611000160492	0.49057000012013	-1.51126000148637
C	8.33376000214525	2.13120000163272	-0.12208999801869
P	-1.57373000308580	0.24778999728418	-0.30653001697520
C	-4.45638000121106	1.41406999788256	0.49964999847427
C	-4.11789000030701	3.94234999797265	0.42238999923681
C	-3.72143000176490	6.37258000185201	0.33542000011122
S	2.29907024074650	-1.48317977200074	2.19976995587449
H	1.47054989415172	-2.11962999501319	-0.08184994993115
C	7.56086000149516	3.03926000006703	0.63711999756164
H	5.59701000063522	3.72516999873524	1.15221000071908
C	4.30342000022530	4.94440999797242	-1.10059999834364
C	2.46772999845202	5.44096999820863	0.44423000004647
C	1.28290000147426	3.67493000070423	1.66781000007311
H	9.42217999760347	2.15272999997802	-0.05157000192156
O	-2.31996000178951	0.52586999945540	1.10271000054386
N	-1.78639000321720	-1.25866000381514	-0.78175000153552
C	-3.71251000135745	0.41013000107113	1.10770000114950
C	-5.87562999797090	1.24212000227124	0.41041999984604
C	-5.10352000058989	4.18926999951464	1.41392000244544
H	-3.17110999966003	7.21579000007909	-0.08760000027039
C	-4.69395000196840	6.57773999997216	1.28929999765436
O	2.29108009058855	-2.93935006532835	2.20417998805226
O	1.62491976700549	-0.76016015728999	3.26643007383800
H	8.05581999772621	3.74571000149421	1.30456000239322
H	4.96773999731143	4.22799000145978	-1.58013000239710
C	4.38191999955597	6.28016000141109	-1.43759999988580
C	2.58685000039700	6.81094999891519	0.08738000064831
C	1.46585999961675	5.01005000124678	1.34492999998818
H	-3.58359000291552	-5.03002000055998	3.31527000110557
S	-2.23490999852835	-1.70811999819716	-2.25539999798845
C	-4.27802999986192	-0.71730000145712	1.76718000211454
C	-6.47621000216585	0.11398999869244	1.06203999797741
C	-6.70833999835106	2.11695000048262	-0.33343999887793
C	-5.38305000255727	5.47229000243052	1.83791000060947
H	-5.63992999764344	3.34840999786021	1.84993999777562
H	-4.92811000120705	7.58796000258323	1.62782999857892
H	5.11046000092001	6.60638999799705	-2.18134999880488
C	3.52309000259512	7.22638000014127	-0.83378000207046
H	1.90729000130746	7.52868000004164	0.55178000204472
H	0.81228999978013	5.75778000039990	1.79834000295344

H	-2.00252999738293	-6.49960000001810	3.99328999828237
H	3.57183974367837	-1.03166278417744	2.14164448569612
O	-1.61409999820179	-0.93409999904260	-3.32872999776880
O	-2.11086999751089	-3.16802999778334	-2.31304999786219
C	-5.66110999781380	-0.81380000104811	1.74995000116400
C	-3.46066000134147	-1.72403999740271	2.48251000021677
C	-7.88411000015493	-0.05689000023427	0.98959999877939
C	-8.07057999897521	1.90945000191257	-0.40339000251675
H	-6.25611999947245	2.95037999954852	-0.86923000129259
H	-6.14034000047950	5.63291999775826	2.60680999832106
H	3.59820999883690	8.28041000191054	-1.10446999836880
H	-3.06826999731148	-5.98747999897381	5.32867000112449
C	0.10996000172008	-4.81721000072441	4.55724999765031
H	0.64956999747063	-5.43953999740607	5.28720999878746
C	-2.07554999720748	-3.66527000226981	4.05711999748009
H	-6.14426999859734	-1.63621000150633	2.27916000230247
C	-2.30037999728303	-1.38535000155790	3.20474999940307
C	-3.87697000190430	-3.06367999828289	2.52459000163377
H	-8.33051000216754	-0.91681000161802	1.49252000035044
C	-8.66787999912269	0.82162999829073	0.27332000165421
H	-8.68886999824508	2.58382000029331	-0.99729000215415
C	-1.36057999741654	-4.65086999865949	4.98782999729662
H	0.16900000172217	-5.31849000188309	3.58070999743577
H	-0.91498000107648	-4.78612999926905	7.12010000208199
H	0.63240999762069	-3.85485999735272	4.47593999919642
C	-1.62776998228857	-2.33688999610623	3.96355999480355
H	-1.92425999821747	-0.36431000259152	3.19272999763028
C	-3.20461999982925	-4.00819999966017	3.30097999970998
H	-4.74490999909241	-3.37567000094062	1.94064999821822
H	-9.74660000163639	0.67027000223678	0.21374999930371
F	-6.81770999980204	-3.40299000190130	0.08056000143323
F	-7.22610000195442	-4.20689000233701	-1.90949999861856

142
3b_3 (TS1-1′)

C	-3.90652000175041	1.38616000135118	-3.18522000215061
C	-6.25928000235236	0.19539000025262	-2.27747000112363
F	-4.03006999972050	4.24575999986133	-3.12713999826639
H	-5.07235000240471	-1.59459000229814	-2.00435999802825
H	-6.82006000163409	-6.87530000076644	2.56350999918266
C	-4.79483000066817	-5.59785000064744	3.93204999744025
H	-6.41834999938861	-3.22961000028482	3.69091999859293
C	-5.08236999933856	-0.55159000087746	-2.31888000173162
C	4.08321999960808	-1.92355998856768	-3.26974999392278
C	-4.35302000235986	-6.27206000236675	1.57077000036127
C	-5.07880000053832	3.54261000039944	-3.64020999990321
C	-5.09410000078646	-3.90112999856097	2.11275000011493
H	-3.73735000190215	-4.15380999889626	0.43924000147561
F	-4.94538000097521	3.58475000076806	-4.99834999917931
C	-5.22899999777123	-5.38436999805285	2.46720999740316
C	-3.90969000109285	0.06065000146744	-2.76297999837536
F	-7.33308000099778	-1.45878000016830	-0.94457999910726
C	3.48303002210250	-0.76145002473806	-2.75980001268179
C	3.47794999668470	-2.66679998976432	-4.28399999551550
H	5.03823999485349	-2.25956999589702	-2.86222000297359
F	-8.20007000052236	-1.01836000218619	-2.90388999833314
C	-6.70089999955882	-5.81293999846240	2.30131000041302
F	-8.40663000046450	0.42340999830196	-1.27140999832882
F	-6.21404000250225	4.21741999908986	-3.33116000085361

C	4.19490999520958	0.04851000169291	-1.74698999182056
C	2.23232006379270	-0.39329013808632	-3.28750006899975
C	2.24661999438791	-2.28414999528084	-4.83325000075124
H	3.98698999939778	-3.55979999795360	-4.64607000020896
H	0.47585142960412	3.35641484053141	-1.44312162015330
H	-7.19068999788107	2.11568000159941	-2.62885000140471
C	-5.09429000169361	2.12021999799287	-3.14039999959378
C	3.52788000110865	0.71427000469759	-0.68196999392382
C	5.57281000087799	0.18946000354602	-1.79558000086173
C	1.63722001849180	-1.13704004109001	-4.29921002078045
H	1.72657985729260	0.50055030241955	-2.93365985214025
C	1.56144999681542	-3.04417999417603	-5.97197999859451
C	-0.19587982732770	3.64597949158632	-0.64962028698200
O	2.13006999781172	0.62617999406877	-0.63818000164717
C	4.16706000166439	1.45026999663572	0.30793000500761
H	6.12395000108085	-0.25828999664044	-2.62352000414208
C	6.29849000234158	0.87567999574857	-0.79580000083189
H	0.66401999827011	-0.80757998505872	-4.66436999514571
C	2.38396999914004	-4.24941999950500	-6.44639999893730
C	1.36609999917205	-2.08457999636772	-7.16469999815314
C	0.18449999891753	-3.54756999850156	-5.49516999943262
C	-1.31615005229427	2.82643018069871	-0.32581975260488
C	-0.00144000229472	4.76508012185517	0.14459012575504
P	1.50029997843962	-0.35086000911689	0.48107000492728
C	5.60015000581061	1.48905999892216	0.29725000154885
C	3.36525000859522	2.16518006926351	1.33682997336663
C	7.71678999835777	0.93778999992269	-0.83341000200450
H	2.53340000146279	-4.98322999905708	-5.64042000000195
H	1.85369999768855	-4.75767999840101	-7.26451999764936
H	3.37130999889910	-3.94669999891217	-6.82495000128168
H	0.87952000176686	-2.61169999849998	-7.99917000081198
H	0.73482999665783	-1.22787000148154	-6.89262000298235
H	2.33290000124496	-1.69693000003944	-7.51770000105587
H	0.30356000220030	-4.27188000157072	-4.67592999997272
H	-0.45484999930189	-2.73153000182134	-5.13234000268049
H	-0.34198999872816	-4.05324000354983	-6.31883000126210
O	-1.47088998967246	1.62716000805916	-1.03258005790761
C	-2.28753006029003	3.16927003888336	0.60167990384659
H	0.83561998854845	5.43147000965023	-0.07041001435116
C	-0.86288008409748	5.08933013614959	1.21802996367709
O	2.23271007482438	0.11341005297287	1.83954999479200
N	-0.03200000779839	-0.10731999748481	0.49277001238292
N	2.03609005334123	-1.87563997896706	0.24307999059412
C	6.36228999806933	2.07525999873010	1.34020999975566
C	3.51361000319424	3.57588000586269	1.55943999429978
C	2.39355983542758	1.49062984861576	2.06565005116000
H	8.23546000158617	0.47306999903874	-1.67405999891856
C	8.42629999858416	1.54030999902507	0.18273999825874
P	-1.38527999213820	0.21389999447392	-0.24380002341842
C	-3.56438998183672	2.41321996472631	0.69319001217918
C	-2.03848998163919	4.30094996124001	1.44730999916283
C	-0.59654000735488	6.19775000596170	2.06472999839747
S	2.78231993317110	-2.84892004321736	1.35203000722286
H	1.32231999189325	-2.60769000306668	-0.64104999525985
C	7.74077000188421	2.09929999954261	1.28491999716581
H	5.84664999905880	2.49751000103067	2.20116000183675
C	4.37658999417147	4.39742999890778	0.78572999876220
C	2.71270007605223	4.20203998872032	2.57137997489829

C	1.54784014448573	2.09509010400877	3.03545989977516
H	9.51623000128259	1.57034000143308	0.14768000248090
O	-2.37639999601459	0.36349000410446	1.03169000505730
N	-1.89254999163097	-0.91141000974579	-1.24305999626538
C	-3.59185000215307	1.04367999463018	0.92564999377803
C	-4.81134999416603	3.10788999816411	0.53387999828654
C	-2.87588997334669	4.63825997089420	2.54261001930806
H	0.29611001315932	6.79473998337014	1.86909000630710
C	-1.43085997157860	6.50046996306474	3.11890002744592
O	2.85942001391572	-4.15070998594039	0.70470999573935
O	2.20164002178749	-2.71711998349773	2.67824999635479
H	8.30517999821069	2.54144000281659	2.10681000162813
H	4.95914000382332	3.94859999711005	-0.01711999491628
C	4.47647999753467	5.75228000197079	1.02625999914994
C	2.86108999601653	5.59410000394738	2.81325000900193
C	1.75867984939601	3.44213992759738	3.28378016435827
H	0.80209192354271	1.50610111108616	3.54672814840253
S	-2.36600000222843	-0.85883999737550	-2.76903999569108
C	-4.79429999817253	0.32097000313657	1.19004000361321
C	-6.03216999778477	2.40102000102886	0.78379000668060
C	-4.89193000149884	4.45440000118921	0.09311000324999
C	-2.57433999613141	5.70546999645521	3.36355000526136
H	-3.75588000039680	4.02842000377726	2.74362999732406
H	-1.21062000618837	7.34926000854618	3.76812998917238
H	5.14048999838719	6.36127999845321	0.41116000262159
C	3.72569998940115	6.35731000445238	2.06018998735115
H	2.25710999252474	6.04812000577523	3.60120999525434
H	1.14972003432534	3.94343001243402	4.03790993613090
C	-7.54418000258219	-0.46458000146719	-1.84292999791904
H	-5.41358999929157	-5.01659000203375	4.62986000085507
O	-1.45410000200664	-0.12194999858584	-3.63944999922667
O	-2.70739999788455	-2.23645999952347	-3.14521999904070
C	-5.98048000020189	1.03599000263590	1.14254000465267
C	-4.84004999732329	-1.12203999804486	1.52263000120902
C	-7.27250000051462	3.07392000220593	0.62583000462369
C	-6.11285000140907	5.07650000118783	-0.06594999966373
H	-3.97601999886223	4.99293000222122	-0.14453000192576
H	-3.22308001348985	5.93598001640585	4.20980999102928
H	3.82546000726815	7.42684999997926	2.25086000659251
H	-3.28731000141147	-6.02001999961352	1.66762999855668
H	3.99991598283204	-2.26140956841129	1.36171211044009
H	-3.74749000019494	-5.29552000173659	4.07263999892984
H	-4.88678000171500	-6.66056000077152	4.20320000206541
H	-6.91792000201511	0.50730999664376	1.32329999624818
C	-5.66540000011842	-1.57104000244088	2.56929000092709
C	-4.15701999782188	-2.09055000005761	0.77819000019238
H	-8.19273000062709	2.51891999809067	0.81793000035569
C	-7.31501000181514	4.38749000260854	0.21215000059943
H	-6.14995999777924	6.10531999974089	-0.42639999942190
H	-2.98168000056263	1.83159000010261	-3.54799000237144
H	-7.36840000254453	-5.22688999887275	2.94872000232649
H	-4.62904999988787	-6.18226000068665	0.51042999973704
H	-4.47955999972675	-7.32461000117182	1.86413000106522
C	-5.78232000086431	-2.92722999955827	2.85674000182449
H	-6.20283000255946	-0.84316999782891	3.18029000138219
C	-4.28967999893680	-3.44937999907346	1.06069999963119
H	-3.51264000102785	-1.79052000157243	-0.04495000017494
H	-8.27339000058954	4.89252999854532	0.08353999813326

C	-6.27224000197864	1.53382000059920	-2.68162999875303
H	-7.03166000164771	-5.67497000094484	1.26183000130220

142
3b_3 (TS1-2')

C	-6.13794999802205	1.64381999818774	4.23942999793021
C	-4.05310999963789	-1.21521999867901	-5.70148000102835
H	-6.32252000199961	0.30772000214860	5.93471999919727
C	-3.63898000170229	-1.93033000110804	-3.34545999875631
F	-8.31941999837062	-0.98159000221573	-2.11017999826930
H	-4.61538999934424	2.78572000222750	5.31525999969200
H	-6.04998000214290	3.79021000005836	2.52479999852235
H	-2.72239000022104	-2.41825999973913	-3.67279999753880
C	1.60213002201370	-4.26886015236330	-0.07599986867012
H	-6.15145999800281	-0.54849999764421	4.38650000187628
H	-5.95190000185908	3.78803000154371	4.70552999737838
F	-7.63071999745531	0.99170000171833	-2.75781000041479
H	-8.19398000167906	1.65758000260525	4.97712000229263
H	-4.61832000057567	-0.26825000238574	2.85699999758519
C	-5.18576000196339	-1.39121999912861	-1.55406000210936
C	-4.00434000116355	-1.98215999873358	-2.00340999786301
C	-4.45383999772759	-1.24380999859243	-4.24815999828844
C	2.70252000051628	-4.14101999026093	0.78669997519396
C	0.53256000546777	-5.10448998069934	0.24001998780314
H	1.57603998204031	-3.72213980408472	-1.01547018434060
H	-7.94922000090783	2.66372000135204	3.52798000214450
H	-4.73419999830433	0.22090999986912	5.13442000184000
C	-5.62474999904958	-0.62386999828351	-3.81563000085366
H	-6.20611000159426	2.74406000261821	6.11894000005301
C	3.85529999994224	-3.25812999811144	0.50523999581163
C	2.67924998415499	-4.87874998199268	1.98253000058383
C	0.51043999710263	-5.85470997356296	1.42542999970016
H	-0.30363000442271	-5.14352000487115	-0.45732000762365
H	1.48190005333077	-1.16110942881478	-3.47989941413119
H	-8.00479000023685	0.89365000022690	3.37620999967291
H	2.92206228247112	0.31876722180506	3.40202209137018
C	3.69309000637825	-1.95345000052466	-0.03119000686265
C	5.14636999745008	-3.62619000156231	0.84130000276966
C	1.61191999827658	-5.71942999640440	2.28695000035619
H	3.48955000013829	-4.75381000247871	2.70228999650490
C	-0.63011999966159	-6.82734999702404	1.74463000278288
C	1.14042006290814	-0.14311032122830	-3.58691948009260
O	2.39433000379625	-1.56215001685836	-0.39036000177281
C	4.71214998909488	-1.02994000156099	-0.19509000245492
H	5.32995000185430	-4.63146000051519	1.22499000111032
C	6.23094999667786	-2.72164999777879	0.76693000204880
H	1.63990000476834	-6.26807000764522	3.22904000113022
C	-0.94767000150697	-6.83760000112027	3.25176999946461
C	-0.17751999752330	-8.24200999953339	1.32223999702752
C	-1.91752999732362	-6.46887000314357	0.98501000026407
C	-0.12971000337575	0.24904027107744	-3.07199025634404
C	1.89622990761082	0.86083015903657	-4.17628042930709
P	1.65707997004495	-0.56989994022552	0.63555002157663
C	6.01844000042115	-1.38760999909451	0.28180999827280
C	4.44576000833770	0.29102000687412	-0.82131999019591
C	7.52503000009006	-3.09441000209546	1.21731000002084
H	-0.10651000026724	-7.20514999905178	3.85496000179583
H	-1.20708999746405	-5.83038999136945	3.60924999908388
H	-1.80524999867298	-7.49798000167874	3.44677000192104

H	0.04112000081461	-8.27428000247368	0.24517000207826
H	0.73147999850328	-8.54368999886894	1.86260000200048
H	-0.96822000165892	-8.97764000089587	1.53631000149701
H	-2.24874000147150	-5.44161999782932	1.19210999948105
H	-1.79848000160164	-6.56055000148051	-0.10276999735420
H	-2.72419999821764	-7.15357000006571	1.28508000005389
O	-0.88409002081585	-0.70437010565406	-2.36140998935739
C	-0.67990004747138	1.51095990254598	-3.23581999448252
H	2.86617001946000	0.61102998072348	-4.60903990169170
C	1.46303996869244	2.20430997205948	-4.23728009001613
O	2.62032999682851	0.72451997605551	0.69832998748248
N	0.25154000919843	-0.27336001688575	0.05356999404126
N	1.56953002084148	-1.07841002737911	2.17240998640462
C	7.09578999771258	-0.46666000079078	0.34443000061580
C	5.23803998996963	0.74008999122041	-1.93346000719097
C	3.43599002787847	1.13158001685710	-0.36493998012970
H	7.67661999796287	-4.11592000061228	1.57215999994309
C	8.55843999827693	-2.18339999975172	1.23905000123005
P	-1.07004999136309	-0.46157999711717	-0.77125998779345
C	-2.09488998210891	1.78543001355376	-2.87338996310452
C	0.15453006483904	2.54927000852954	-3.76621986396566
C	2.30303004496592	3.22459000269221	-4.75722989127263
S	2.63693999732484	-1.00262000185899	3.43172000367404
H	0.41643999156663	-1.73469000589191	2.51310000532978
C	8.33318999910346	-0.85390999863719	0.81673000176991
H	6.93529000050111	0.56361000168832	0.03036999781310
C	6.14673000692528	-0.11036999586168	-2.61729999487926
C	5.07402998724991	2.08031999781451	-2.41292001338763
C	3.25253998530250	2.46694999902745	-0.82617000932558
H	9.54398999732777	-2.47934999983126	1.60123000172366
O	-1.69606999606659	1.03549000750431	-0.63841999868093
N	-2.07138000610234	-1.53578999356685	-0.18675000567804
C	-2.56671999881040	1.57135000234153	-1.58576000157599
C	-3.00095999616319	2.30276000875460	-3.85850000349270
C	-0.23469000014234	3.91318000113868	-3.78199000422656
H	3.29186000079274	2.94396000086087	-5.12548999969850
C	1.89159000348997	4.53924999719229	-4.76921998783443
O	3.86045999632358	-1.73784999766759	3.16024000161671
O	1.83602999758087	-1.33214999409717	4.60200000077106
H	9.14123000149313	-0.12309000010565	0.87167000035158
H	6.25754999803002	-1.14209000057804	-2.28635000393018
C	6.87300000669019	0.34579000237143	-3.69801999675013
C	5.85463999651778	2.52716999653967	-3.51195000244343
C	4.10729000589069	2.91917999482967	-1.81502999624266
H	2.46735514769331	3.07727894642063	-0.40622556711096
S	-2.88109999875766	-2.75305999937585	-0.83217999900443
C	-3.84554999706283	2.01296000160082	-1.13628000028599
C	-4.31055000273087	2.71096999974250	-3.44319000243599
C	-2.67154000331572	2.39074999666894	-5.23569000041012
C	0.61768999825533	4.88526999955605	-4.26192001089144
H	-1.21028001301096	4.19184999590989	-3.38629002113446
H	2.54995998722733	5.31728999447072	-5.15903002959057
H	7.55444999554355	-0.33170000197746	-4.21452000472511
C	6.73899000615093	1.68050000333166	-4.14394999352931
H	5.72657000303722	3.55631999844425	-3.85420999692770
H	3.97710000403822	3.93244000603287	-2.19814999573235
C	-5.44405000036230	1.73914000078153	2.87736000068664
H	-5.46450000074378	-1.45338000075310	-0.50292000143033

O	-2.04021999804166	-3.66406000135999	-1.60098000237862
O	-3.70741999743379	-3.31200000308425	0.24529999987768
C	-4.67609999793412	2.59114999762331	-2.08377999829640
C	-4.30456000165987	1.91858000106954	0.26873999922717
C	-5.22395999876299	3.20724999586866	-4.41041000112770
C	-3.58403000305720	2.87012999883339	-6.15233999903298
H	-1.69207000024834	2.05302000187123	-5.57091000135588
H	0.30695000321139	5.93097000223074	-4.24617999757756
H	7.32713999392804	2.03223999942039	-4.99271000526962
C	-5.81417000207480	0.32660999862994	4.95941999836703
F	-7.23540000033084	0.30692999880781	-0.71417999983516
H	-6.24343999811384	-0.06932999846284	-4.51897000138790
C	-5.98874999754079	-0.70931999927278	-2.46873000072044
H	-5.67264000213254	2.91600999756323	-1.78023999857915
C	-4.17641999985609	0.75688000177197	1.04052999792858
C	-4.97855999971208	3.01027000007185	0.84630999753044
H	-6.21966000211934	3.50848000194623	-4.07859999997533
C	-4.86932000108545	3.28845999812834	-5.73939999841518
H	-3.31559999987254	2.91124000059068	-7.20879000143290
C	-7.28704000159408	-0.09462000160617	-2.00844999806804
C	-5.70016000023606	2.81259000159023	5.14402999753227
C	-7.66298000013885	1.72068999927819	4.01486999825888
F	-2.73639999841614	-0.89841999812373	-5.85317000249211
C	-4.74098999807019	0.66816999949835	2.31311000129926
H	-3.64413999815592	-0.10630000105222	0.64591999768194
C	-5.53092000077441	2.91962999873123	2.11955999766174
H	-5.06285999775508	3.94467000062312	0.28761999957340
H	-5.58126999992971	3.66349999990925	-6.47598000051600
F	-4.22781999815483	-2.43679000219815	-6.28512000061844
F	-4.77460000052134	-0.32185000202460	-6.42296000047223

126
3b_4 (cat)

H	0.83586000205982	-5.83991000158813	1.29773999796600
H	-5.37322999642427	2.05755999832441	-0.22900999830064
C	-3.83478999872539	3.87792000105662	-4.30792000116123
C	-2.36741999826033	4.33714999925507	-4.43634000125379
H	-5.28610999865416	3.82624999985184	-1.90383999863348
C	-4.77977000006314	1.89882999650986	-1.13001000177550
H	1.17062999788576	-3.39937000081245	1.78822000156203
C	-3.26420000195208	0.57659000036354	-2.43855999845453
C	-4.72110000237550	2.91275000058920	-2.08504000248541
C	-3.93564999790142	2.78637000292610	-3.23810999911359
C	-3.21494998279081	1.59119000353372	-3.38689999085477
C	0.21410999860924	-3.80057999974305	1.49078000198106
C	0.00385999856754	-5.13986000155356	1.19958000176201
C	-1.25098000045699	-5.63645000120697	0.77498000018564
C	-1.41871999961438	-7.00242999896555	0.42255000111777
C	-2.62643000007118	-7.47151000165869	-0.04642000141536
C	-3.72117000119640	-6.58703999834469	-0.18108000087127
C	-3.59717000146397	-5.25799999883905	0.16882999817453
C	-2.37045999919971	-4.74485000171716	0.66549999859962
C	-2.18320000237062	-3.36683000001659	1.00670000076051
C	-0.91758000177033	-2.94597000036258	1.37970999895508
O	-0.75089999833857	-1.58188000184857	1.64663000173169
P	-0.68401999698214	-0.59113000217619	0.36668001021758
N	-0.93474000188054	0.90381000187728	0.82917000330405
S	-0.65496000072604	1.46619999724840	2.31421999816338
O	-0.73287001854595	2.92170997012838	2.24804000636995

O	0.51071999971057	0.85799999944324	2.95701000193575
H	0.62354999387986	5.02840994028883	1.82892994209106
H	3.75504000158871	0.62660999947992	1.91117000302842
C	-4.68084000178470	-3.47850999793181	2.63580999929500
C	-6.92161999839474	-2.65479999986764	3.06965000152491
C	-5.85993999932372	-3.54590000085795	3.34896999964525
O	3.13487001087560	-0.46195999735352	-0.21738001196132
H	2.70194999956321	2.31522999753013	3.31196000579233
C	-4.05547000118149	0.70639999789134	-1.28257000341021
O	-1.88953999951562	-1.10326999922423	-0.58293999534363
C	-4.18920000055377	-0.37805999958984	-0.28382000038779
C	-5.56817000147287	-1.58425999722534	1.35374999810174
C	-5.39177000133880	-0.57661999823783	0.37926999943750
C	-4.50320000210803	-2.50726999808394	1.61734000213521
C	-3.13794999845340	-1.28838999777078	0.02787000225470
C	-6.77716000216713	-1.69605000018508	2.09030999745340
C	-3.28483999810087	-2.37625000207751	0.87928999919734
H	0.93234998647590	6.54613992503951	2.70752993166686
N	0.59803999690283	-0.97145999800904	-0.49360000280046
P	1.81168003840415	-0.24307996769040	-1.10149999651104
N	1.75216000806815	1.41528995555062	-1.30400009271037
S	0.95366999126015	2.16173974707594	-2.59888026906380
O	-0.13265008295583	1.26477002920608	-2.93607999610850
O	1.94282999862792	2.56892003747926	-3.57842996827440
H	0.47236579549226	3.25080170372426	-1.95860593324610
C	4.37343000030512	3.37069999945117	-0.00671999865530
C	1.92415999730324	4.49950998907320	4.18461998204284
C	3.60036998724617	6.11633998774023	3.26808999232133
H	4.41849000297235	5.65146999842279	3.83745999593990
H	3.75808999195386	5.36762999745769	0.45896999996071
C	2.52660998789828	5.07371997040396	2.89488997834870
H	1.78180008727552	6.22557024465267	1.17979025934890
H	-1.73750000045200	1.04910999981937	3.01212999900730
H	4.03367002317862	6.59201998994645	2.37694998471953
C	1.39861001562634	5.75802007712106	2.09723005872957
H	3.15471999891989	6.90726000659329	3.88970996556682
H	1.48180999073785	5.31671001142635	4.77236999883605
H	2.68827000659639	4.01532003941310	4.81063001941497
H	4.81604000128078	3.66402000235001	-0.96133999789091
H	1.12889001619628	3.77332000585487	3.96825000930820
C	3.76858998846541	4.33097998764748	0.80008999914331
H	2.56957994817805	1.93409008628545	-0.94893982704568
O	2.20454999184876	-0.86057999330879	-2.53797999964251
C	2.71400998149077	-2.18009000574029	-2.46266000297673
C	4.04333999646063	-2.32257000053428	-2.08784000317248
C	4.57381000102997	-3.64340000127163	-1.93365000011610
C	5.88490000273521	-3.89940999799500	-1.45339999814850
C	6.34146000055153	-5.19102999859693	-1.29079000179028
C	5.51136000250095	-6.29221999780116	-1.60361000247388
C	4.22883999797564	-6.07785999987789	-2.05821000240470
C	3.72150000179054	-4.76054000012848	-2.22046999997231
C	2.38508000169271	-4.53627000059384	-2.62186999949254
C	1.83752999443905	-3.26589000177172	-2.73055999828833
H	0.80877999914157	-3.08367999787804	-2.99967999890942
H	-4.38848999920214	5.57072000063356	-3.02653999815688
C	-4.69626999984733	5.10302000208928	-3.97289000002589
H	-3.69272000084250	2.44639999778907	-5.97093999878803
C	-4.30711000056583	3.30215999972032	-5.65895000253565

```
  H   -2.27214000117130      5.08282000082164     -5.24002999827836
  H   -1.69662999986866      3.49859999861944     -4.66889000056090
  H   -5.35117999866619      2.96320000021928     -5.59471000085132
  H   -4.67200000125399     -6.95709000053506     -0.56760999946630
  H   -3.86202999945741     -4.15828000094267      2.86885000034581
  H   -7.84941999987742     -2.71999000150928      3.63982999794642
  H   -7.58139999905805     -0.98772999769630      1.88250999774976
  H   -0.56222999775227     -7.67307999805929      0.52027000233585
  H   -2.73901000103255     -8.52143000035441     -0.32035999769240
  H   -5.96823999915717     -4.28470999822379      4.14412000208801
  H   -2.02133000206182      4.79850999918236     -3.50070999785171
  H   -4.44671000162096     -4.58554999820891      0.05819999944032
  H   -6.23703000052933      0.07337999813507      0.14973999936400
  H   -5.76228999780160      4.84271999851875     -3.89926999889735
  H   -2.57612995123019      1.44568998975771     -4.25898997320068
  H   -2.67630999826598     -0.32322999983759     -2.60376000014373
  H   -4.59044999903032      5.85510999807389     -4.76807999743789
  H   -4.24092999849856      4.07101000082497     -6.44361999949998
  H   1.75480000044488     -5.39903000027313     -2.84527999801576
  H   3.57260999833283     -6.91953999858494     -2.28938000203596
  H   5.88614000215935     -7.30862000172810     -1.47566000158292
  H   7.35029999896187     -5.36348999908907     -0.91294000161477
  H   6.53259000272858     -3.06034999810816     -1.20374999774294
  C   4.84322000216542     -1.10947999795251     -1.78279000120367
  C   4.36894000647517     -0.19739000506983     -0.85309001184275
  C   5.02715999860683      1.01551999761030     -0.51570000194510
  C   6.24496000129203      1.25895000219016     -1.12898999992648
  C   6.78779999918543      0.38108000235381     -2.09557999860892
  C   8.01576000267579      0.67436999825609     -2.74621999781465
  C   8.49931999769485     -0.14132999772147     -3.74512000049454
  C   7.76275000231436     -1.28032999982338     -4.14386000084049
  C   6.57636000117194     -1.60423000250349     -3.51933999814341
  C   6.06735000036222     -0.80502999910168     -2.46268000262920
  H   6.01408000153221     -2.48043999937452     -3.83953999938543
  H   8.13314000046398     -1.90620000175396     -4.95699000077997
  H   9.43319000015135      0.09568000205712     -4.23744000075887
  H   8.56146000172724      1.57172000056955     -2.44752000151601
  H   6.79534000168931      2.16644000236600     -0.87297999969305
  C   4.39842999983833      2.01494999955895      0.37809999989292
  C   3.78358999746574      1.66780000091259      1.59158000260457
  C   3.18858998997670      2.63856998987595      2.39446000212764
  C   3.16945001179118      3.99031998903222      2.02518998504307
126
3b_4 (TS1-1)
  C   -2.30037999801528     -1.38535000000019      3.20475000148716
  H   -1.92425999997510     -0.36431000109305      3.19273000043088
  C   -1.62777000035786     -2.33689000262934      3.96356000229681
  H   0.63240999768910     -3.85485999756045      4.47593999900587
  H   -0.73252999726357     -2.02173999788886      4.50258000188742
  C   -2.02294000246769     -6.03531999969203      4.99041000213017
  H   -0.91498000106172     -4.78612999928306      7.12010000204313
  C   -8.66787999895249      0.82162999824349      0.27332000164666
  C   2.40809999545348     -0.73060999998011     -3.40031999907950
  H   -4.74491000002606     -3.37567000076955      1.94064999919927
  C   -1.40585000245678     -4.08844999881324      6.42482000013178
  H   -1.47975999793818     -6.70111999969997      5.67645999993484
  H   -8.33051000201408     -0.91681000157334      1.49252000034072
  H   -3.55657585090036     -1.40956714114968     -2.28320256216272
```

H	-2.44460000160277	-3.94403999933401	6.75532000158638
C	-1.36057999824208	-4.65086999765441	4.98782999860925
H	-0.89059000127030	-3.12092000217477	6.49546999941559
C	3.62341999970352	-0.95937000039389	-2.72525000086235
C	1.85374999945389	-1.70216999658054	-4.22477000170068
H	1.88124999958194	0.21391000086348	-3.28266999850464
H	-9.74660000153357	0.67027000228719	0.21374999947218
H	0.16900000177779	-5.31849000212644	3.58070999736523
H	-8.68886999821365	2.58382000039491	-0.99729000203233
C	-3.87697000020294	-3.06367999903322	2.52459000014014
C	4.26687000013278	0.06791999998271	-1.87476000192236
C	4.25038000243618	-2.19872999870773	-2.92940999821160
C	2.48168999749381	-2.94065000162773	-4.44014000089112
H	0.89258005750070	-1.48705001338648	-4.69425002856839
H	-1.17136000031445	3.39906999885451	-2.15176000255192
C	-3.20461999811836	-4.00820000013958	3.30097999800089
H	0.50307000212134	3.32400000041687	2.32592000047092
C	3.51922999637620	0.99815999998172	-1.09713999949097
C	5.64694999906748	0.19579000047401	-1.83470000166146
C	3.69719999700561	-3.15939000001525	-3.77734999795915
H	5.17652999867529	-2.42872999777698	-2.40067000237678
C	1.82098000119338	-3.98248999940735	-5.34584000173705
C	-1.97639000485445	3.58384000078591	-1.45721000091410
O	2.12786999430547	0.87162000050948	-1.11042999574973
C	4.08384999831027	2.05128999879297	-0.38811000035146
H	6.26096000260480	-0.46878999745434	-2.44406999911986
C	6.29437999849272	1.15960999904090	-1.02880000206301
H	4.22566000290649	-4.10435000051673	-3.90119999923031
C	1.60576999727585	-3.37025999995797	-6.74553000058301
C	0.45649999951440	-4.37949999873215	-4.74473000008431
C	2.67553999960528	-5.24831000202804	-5.49851999900502
C	-2.72059002433627	2.49514999360415	-0.92380998968993
C	-2.35520000000011	4.85129999857295	-1.04403999840928
P	1.34917999213011	0.46058000546968	0.24368000172773
C	5.51179000098684	2.10443000104359	-0.28544999777750
C	3.21600000148196	3.10087000103413	0.21078999876993
C	7.71064999760802	1.21148999792907	-0.93754000020501
H	2.56352000030247	-3.06864999917205	-7.19437000151034
H	1.12970999969462	-4.10731999775106	-7.40930999932145
H	0.95584000047331	-2.48579999827806	-6.70457999845124
H	-0.19848999987886	-3.50932999844999	-4.60317000102982
H	-0.05727000218297	-5.08818000039399	-5.41145999742747
H	0.58106999731614	-4.87039000304491	-3.76896000027900
H	2.82697000057386	-5.75976000168271	-4.53625000274500
H	2.16454000234077	-5.95599999794157	-6.16659000020469
H	3.66126999872813	-5.02729999812037	-5.93423999828084
O	-2.37450002128469	1.20710000590401	-1.33766997944293
C	-3.77315000640354	2.62553999774652	-0.02941999621005
H	-1.83197000093329	5.71948000069219	-1.44956999805058
C	-3.39688000027919	5.06336000175131	-0.11094000174060
O	2.02468999659483	1.39223000215234	1.37154000317958
N	-0.12939000119123	0.81610005167799	-0.05477003947611
N	1.76150999344383	-1.05275000155405	0.70704000312068
C	6.18380000151427	3.02938000239161	0.55404999748463
C	3.35936000228004	4.48337999757201	-0.14625000180208
C	2.18572000200530	2.75396999887298	1.06981999905217
H	8.29611000165758	0.49057000025315	-1.51126000146615
C	8.33376000220300	2.13120000177434	-0.12208999800228

P	-1.57372947118225	0.24778997505143	-0.30653011478384
C	-4.45638001061401	1.41406999580833	0.49964999807066
C	-4.11789000205905	3.94234999843604	0.42238999876210
C	-3.72143000123540	6.37258000192005	0.33542000065227
S	2.29906999867731	-1.48318000180873	2.19977000123847
H	1.47054999803457	-2.11963000369879	-0.08184999659551
C	7.56086000159794	3.03926000009850	0.63711999765743
H	5.59701000059719	3.72516999879011	1.15221000065893
C	4.30342000022802	4.94440999794063	-1.10059999859339
C	2.46772999860442	5.44096999841938	0.44422999967424
C	1.28290000166945	3.67492999929225	1.66780999815298
H	9.42217999761845	2.15272999983841	-0.05157000169067
O	-2.31995998490948	0.52587001087785	1.10271002086008
N	-1.78639058201518	-1.25866002888418	-0.78174980492725
C	-3.71251001550810	0.41012999063773	1.10770000468646
C	-5.87563000080848	1.24212000213090	0.41041999869880
C	-5.10352000067042	4.18926999984315	1.41392000252905
H	-3.17110999930414	7.21579000009960	-0.08760000001989
C	-4.69395000165201	6.57773999991678	1.28929999791164
O	2.29108000229864	-2.93934999919684	2.20417999932966
O	1.62492000041013	-0.76016000208717	3.26643000152729
H	8.05581999778315	3.74571000151190	1.30456000253962
H	4.96773999750583	4.22799000159101	-1.58013000221561
C	4.38191999960568	6.28016000147745	-1.43759999991492
C	2.58685000026648	6.81094999901981	0.08738000027133
C	1.46585999979244	5.01005000139250	1.34493000046643
H	-3.58359000189145	-5.03002000106585	3.31526999982801
S	-2.23490999625798	-1.70812001598219	-2.25540010066108
C	-4.27803000398394	-0.71730000510420	1.76718000185707
C	-6.47621000280080	0.11398999853038	1.06203999810276
C	-6.70833999886852	2.11695000061184	-0.33343999914467
C	-5.38305000209696	5.47229000243399	1.83791000109756
H	-5.63992999766751	3.34840999788692	1.84993999756387
H	-4.92811000119728	7.58796000254929	1.62782999852253
H	5.11046000095426	6.60638999796617	-2.18134999871287
C	3.52309000258740	7.22638000018652	-0.83378000210011
H	1.90729000118017	7.52868000005005	0.55178000216355
H	0.81228999984484	5.75778000026140	1.79834000298492
H	-2.00252999737657	-6.49959999998548	3.99328999825032
H	3.57183999832611	-1.03165999678467	2.14164000088489
O	-1.61410006071477	-0.93410000132650	-3.32872995739472
O	-2.11086980907772	-3.16802996164781	-2.31305002275417
C	-5.66110999934392	-0.81380000321692	1.74995000106822
C	-3.46065999925303	-1.72403999783227	2.48250999854840
C	-7.88411000005561	-0.05689000014097	0.98959999889991
C	-8.07057999899100	1.90945000204682	-0.40339000259860
H	-6.25611999935041	2.95037999953477	-0.86923000135931
H	-6.14034000019094	5.63291999766229	2.60680999850605
H	3.59820999883256	8.28041000181971	-1.10446999825202
H	-3.06826999740792	-5.98747999876003	5.32867000103127
C	0.10996000227642	-4.81721000021958	4.55724999798377
H	0.64956999744786	-5.43953999760192	5.28720999867142
C	-2.07555000136052	-3.66527000172708	4.05711999858658
H	-6.14426999905615	-1.63621000188837	2.27916000242696

126

3b_4 (TS1-1')

C	-5.66540000056467	-1.57104000253432	2.56929000018497
C	-5.78232000079431	-2.92722999951843	2.85674000210048

H	-8.19273000055075	2.51891999797100	0.81793000087137
H	-6.20283000261766	-0.84316999768318	3.18029000162473
H	-6.82006000163423	-6.87530000076818	2.56350999915788
C	-4.79483000066449	-5.59785000064840	3.93204999743356
H	-6.41834999900656	-3.22961000031424	3.69091999900227
C	-4.28967999889870	-3.44937999913239	1.06069999977925
C	4.08321999062278	-1.92355998892124	-3.26974999621812
C	-4.35302000237269	-6.27206000236436	1.57077000034758
H	-6.14995999747779	6.10531999991980	-0.42639999832362
C	-5.09410000071143	-3.90112999853269	2.11275000028371
H	-3.73735000173851	-4.15380999877081	0.43924000162892
C	-4.15701999808542	-2.09055000058722	0.77818999963972
C	-5.22899999770382	-5.38436999803486	2.46720999747488
H	-3.52015671472549	-0.14843287472646	-2.73909567393208
H	-7.03166000167042	-5.67497000093026	1.26183000128969
C	3.48302999901320	-0.76144998580133	-2.75979999591494
C	3.47794999913893	-2.66679999585483	-4.28399999843465
H	5.03823999901036	-2.25957000015746	-2.86222000440248
H	-4.47955999976507	-7.32461000117424	1.86413000103301
C	-6.70089999956299	-5.81293999845248	2.30131000041208
H	-3.51264000169472	-1.79052000155867	-0.04495000030816
C	-7.31501000168595	4.38749000216873	0.21214999966148
C	4.19490999700220	0.04851000896415	-1.74698999769868
C	2.23232008243968	-0.39329016960595	-3.28750008432098
C	2.24661999762605	-2.28414998810447	-4.83324999157513
H	3.98699000221665	-3.55980000144119	-4.64607000246958
H	0.47494071576566	3.35584820268969	-1.44367726071073
H	-4.62904999987927	-6.18226000068303	0.51042999971204
H	-7.36840000255074	-5.22688999887642	2.94872000232346
C	3.52788000225724	0.71426999889823	-0.68196999205529
C	5.57281000238472	0.18946000102387	-1.79558000237846
C	1.63722000329987	-1.13703999773846	-4.29920999382023
H	1.72657988202225	0.50055024698355	-2.93365987919941
C	1.56144999825624	-3.04417999548961	-5.97197999895423
C	-0.19587979295870	3.64597948799028	-0.64962020965711
O	2.13007000432139	0.62617999397351	-0.63818000073926
C	4.16706001364288	1.45026998773944	0.30793001907459
H	6.12395000220084	-0.25828999896475	-2.62352000201319
C	6.29849000089794	0.87567999926509	-0.79580000280714
H	0.66401999513832	-0.80757999327639	-4.66437000361978
C	2.38396999960079	-4.24942000095124	-6.44639999995082
C	1.36609999952388	-2.08457999748089	-7.16469999881746
C	0.18449999940588	-3.54756999929467	-5.49516999988948
C	-1.31615013529845	2.82643030360430	-0.32581978296216
C	-0.00144009562817	4.76508025994899	0.14459012161708
P	1.50029996709216	-0.35086000921417	0.48106999624880
C	5.60015000257314	1.48905999639800	0.29725000076613
C	3.36524998856585	2.16518004386776	1.33682996145097
C	7.71678999809006	0.93779000118562	-0.83341000218370
H	2.53340000112479	-4.98322999915775	-5.64042000005083
H	1.85369999762546	-4.75767999901096	-7.26451999808880
H	3.37130999868723	-3.94669999880522	-6.82495000168260
H	0.87952000031335	-2.61169999802779	-7.99917000110065
H	0.73482999750967	-1.22787000159687	-6.89262000242981
H	2.33290000118126	-1.69693000127693	-7.51770000208239
H	0.30356000250127	-4.27188000210201	-4.67592999970341
H	-0.45484999846620	-2.73153000195880	-5.13234000237621
H	-0.34198999986960	-4.05324000297701	-6.31883000182238

O	-1.47088994898565	1.62715994033848	-1.03258008368147
C	-2.28753004251090	3.16926998393751	0.60167985573094
H	0.83561999973700	5.43146996443732	-0.07041002629336
C	-0.86288004185671	5.08933006901082	1.21802995555246
O	2.23271007050582	0.11341001968516	1.83955001152702
N	-0.03200000211378	-0.10731999401489	0.49277000113969
N	2.03609002214640	-1.87563999094237	0.24307999520749
C	6.36228999658471	2.07526000154054	1.34020999794281
C	3.51360999141399	3.57588000062774	1.55943999701475
C	2.39355989448677	1.49062994503607	2.06565000140882
H	8.23546000160663	0.47306999829424	-1.67405999856282
C	8.42629999875622	1.54030999952919	0.18273999805729
P	-1.38527998853233	0.21389999528239	-0.24380001518315
C	-3.56438996820177	2.41321996323563	0.69319003972964
C	-2.03848994576601	4.30094993667817	1.44731003674207
C	-0.59653997937698	6.19774997031086	2.06473000812825
S	2.78231998015526	-2.84892001561223	1.35203000289775
H	1.32231999501561	-2.60769000056469	-0.64104999922090
C	7.74077000181105	2.09929999990643	1.28491999691164
H	5.84664999944163	2.49751000166537	2.20116000194794
C	4.37658999462031	4.39742999797764	0.78573000061535
C	2.71270006588414	4.20203999405769	2.57138000397875
C	1.54784005017466	2.09509004470562	3.03545995296310
H	9.51623000148746	1.57034000108001	0.14768000260897
O	-2.37639999527771	0.36349000407544	1.03169001842384
N	-1.89255002424613	-0.91140999786361	-1.24305998751504
C	-3.59185000319496	1.04368000094422	0.92565000433472
C	-4.81134999616049	3.10789000496967	0.53388000445799
C	-2.87588998445137	4.63825998922410	2.54261001692341
H	0.29611000956445	6.79473999388906	1.86909000621232
C	-1.43085998249667	6.50046997429408	3.11890001733657
O	2.85942000235159	-4.15070999536999	0.70470999762119
O	2.20164000804952	-2.71711999427391	2.67824999916520
H	8.30517999853627	2.54144000178993	2.10681000225096
H	4.95914000693655	3.94859999726933	-0.01711999350491
C	4.47647999610351	5.75228000258209	1.02625999706652
C	2.86108997675621	5.59410000835590	2.81324999279043
C	1.75867997778604	3.44213995080137	3.28378011609355
H	0.80186059333219	1.50592046227726	3.54617520888219
S	-2.36599998837643	-0.85884000113280	-2.76904000227595
C	-4.79429999815745	0.32097000343998	1.19004000063467
C	-6.03216999903437	2.40101999964617	0.78379000180528
C	-4.89193000327548	4.45440000147834	0.09311000105853
C	-2.57434000822661	5.70547001005183	3.36354999735875
H	-3.75588000549401	4.02842000699108	2.74362999252742
H	-1.21062000924034	7.34926001367737	3.76812999061204
H	5.14048999717493	6.36127999966134	0.41116000090057
C	3.72569999362274	6.35731000194442	2.06018999046179
H	2.25710999160626	6.04812000561000	3.60120999319401
H	1.14971999432759	3.94343001238236	4.03790994943429
H	-8.27339000033386	4.89252999911360	0.08353999996678
H	-5.41358999929451	-5.01659000202780	4.62986000084420
O	-1.45410000215292	-0.12194999699069	-3.63945000036557
O	-2.70739999519371	-2.23646000031359	-3.14521999954130
C	-5.98048000029237	1.03599000118327	1.14254000132625
C	-4.84004999719532	-1.12203999920975	1.52262999754588
C	-7.27250000089912	3.07392000061464	0.62583000083627
C	-6.11285000158166	5.07650000055137	-0.06595000198446

H	-3.97601999860535	4.99293000202441	-0.14453000000247
H	-3.22308000649373	5.93598000742410	4.20980999857584
H	3.82546001112192	7.42684999859638	2.25086001036782
H	-3.28731000142907	-6.02001999962047	1.66762999856338
H	4.00013773781635	-2.26182605390236	1.36222548668138
H	-3.74749000020513	-5.29552000174636	4.07263999891333
H	-4.88678000173416	-6.66056000076691	4.20320000205979
H	-6.91792000192657	0.50730999672141	1.32329999696892

126

3b_4 (TS1-2')

C	-6.13794999801448	1.64381999818290	4.23942999793042
H	-6.21966000176910	3.50848000265154	-4.07860000000057
H	-6.32252000200267	0.30772000214675	5.93471999919723
C	-5.70016000024481	2.81259000158140	5.14402999752568
C	-4.17641999992521	0.75688000183525	1.04052999792785
H	-4.61538999935020	2.78572000222299	5.31525999969100
H	-6.04998000214563	3.79021000003344	2.52479999853449
H	-5.58126999998160	3.66349999984571	-6.47598000054856
C	1.60213003243036	-4.26886014933219	-0.07599990565743
H	-6.15145999800497	-0.54849999765004	4.38650000187896
H	-5.95190000185887	3.78803000154383	4.70552999737800
H	-5.06285999767576	3.94467000078889	0.28761999962866
H	-8.19398000168059	1.65758000260759	4.97712000229478
H	-4.61832000055170	-0.26825000237733	2.85699999755814
H	-3.64413999821306	-0.10630000098787	0.64591999760019
H	-3.72107237450367	-2.16684932543409	-1.71992089002055
H	-3.31559999980568	2.91124000078868	-7.20879000138129
C	2.70251999067110	-4.14101996762403	0.78669997547876
C	0.53255999806787	-5.10448995929462	0.24001998253430
H	1.57603998256741	-3.72213984596760	-1.01547013324691
H	-7.94922000090657	2.66372000134978	3.52798000214150
H	-4.73419999830800	0.22090999987365	5.13442000183675
C	-4.97855999965143	3.01027000019531	0.84630999752744
H	-6.20611000159895	2.74406000262292	6.11894000005168
C	3.85530000255627	-3.25813000521197	0.50523999773624
C	2.67924998252121	-4.87874998684543	1.98253000401195
C	0.51043999761193	-5.85470997785012	1.42543000360435
H	-0.30363000112410	-5.14352001059417	-0.45732000234863
H	1.48090294254384	-1.16142938476549	-3.47984485279670
H	-8.00479000024337	0.89365000022179	3.37620999967466
H	2.92251869092317	0.31862934101880	3.40246448420290
C	3.69309000313773	-1.95345000123704	-0.03119000013649
C	5.14636999757776	-3.62619000157301	0.84130000228015
C	1.61192000113438	-5.71943000316860	2.28694999940027
H	3.48955000333363	-4.75381000472933	2.70228999612890
C	-0.63011999719571	-6.82735000234126	1.74463000177531
C	1.14042010686351	-0.14311021146168	-3.58691947287493
O	2.39432999780548	-1.56215000387248	-0.39035999844549
C	4.71214999427610	-1.02994000031200	-0.19509000247733
H	5.32995000168292	-4.63145999991492	1.22499000151648
C	6.23094999699869	-2.72164999809834	0.76693000012711
H	1.63990000335420	-6.26807000426963	3.22904000200458
C	-0.94767000161360	-6.83760000062667	3.25176999961234
C	-0.17751999784103	-8.24200999985981	1.32223999736565
C	-1.91752999746547	-6.46887000339839	0.98501000047415
C	-0.12971005993611	0.24904018163923	-3.07199034078702
C	1.89622984153226	0.86083009378737	-4.17628054419869
P	1.65707999259779	-0.56989997930150	0.63555000715960

C	6.01843999920393	-1.38760999887901	0.28180999921367
C	4.44576000602640	0.29102000411364	-0.82131999332282
C	7.52503000038902	-3.09441000229416	1.21730999915120
H	-0.10651000017570	-7.20514999855968	3.85496000156323
H	-1.20708999960936	-5.83038999680269	3.60925000039579
H	-1.80524999911427	-7.49798000046932	3.44677000181900
H	0.04112000084431	-8.27428000225352	0.24517000219482
H	0.73147999836411	-8.54368999857149	1.86260000203558
H	-0.96822000163750	-8.97764000062380	1.53631000145476
H	-2.24874000153390	-5.44161999738225	1.19210999905821
H	-1.79848000166014	-6.56055000150799	-0.10276999739440
H	-2.72419999832669	-7.15356999966947	1.28508000032706
O	-0.88409001126281	-0.70437008754915	-2.36140997004783
C	-0.67990000462360	1.51095993364832	-3.23581989988187
H	2.86617003412667	0.61102999142435	-4.60903986973821
C	1.46304000094049	2.20430997637770	-4.23727998866228
O	2.62032999250857	0.72451999561294	0.69832999380633
N	0.25154000191227	-0.27336001064435	0.05356999399563
N	1.56953000929909	-1.07841002223120	2.17240999295768
C	7.09578999787889	-0.46666000120846	0.34442999922498
C	5.23803999665908	0.74008999472122	-1.93346000339229
C	3.43599001262123	1.13158000420801	-0.36493999714062
H	7.67661999784422	-4.11592000053030	1.57216000040122
C	8.55843999823411	-2.18339999969213	1.23905000152632
P	-1.07004998111602	-0.46157999972834	-0.77125998259125
C	-2.09488998589116	1.78543001889253	-2.87338998355433
C	0.15453006330540	2.54927001533779	-3.76621988125184
C	2.30303005701614	3.22459000687975	-4.75722987094454
S	2.63693999701903	-1.00261999233510	3.43172000163736
H	0.41643999704191	-1.73468999804824	2.51310000290356
C	8.33318999918364	-0.85390999862779	0.81673000172146
H	6.93529000042725	0.56361000184702	0.03036999830428
C	6.14673000451471	-0.11036999658124	-2.61729999677729
C	5.07402999523590	2.08031999866902	-2.41292000430911
C	3.25254000265831	2.46695000238868	-0.82616999369660
H	9.54398999731505	-2.47934999982113	1.60123000176437
O	-1.69606999454642	1.03549000482995	-0.63841999736143
N	-2.07138002701058	-1.53578997960524	-0.18675001528287
C	-2.56671999779814	1.57135000338828	-1.58576000153644
C	-3.00095999988206	2.30276000266698	-3.85850000477011
C	-0.23469001509756	3.91317999738989	-3.78199004042228
H	3.29185999693300	2.94396000086018	-5.12549000964825
C	1.89158998394459	4.53924999204324	-4.76922003527221
O	3.86045999896774	-1.73785000108675	3.16024000176914
O	1.83602999783258	-1.33215000045690	4.60200000152290
H	9.14123000140305	-0.12309000001262	0.87167000072839
H	6.25754999829694	-1.14209000057957	-2.28635000358102
C	6.87300000213361	0.34579000054621	-3.69802000019409
C	5.85464000181949	2.52716999899051	-3.51194999853726
C	4.10728999232145	2.91917999576754	-1.81503000765323
H	2.46723429396866	3.07710962082426	-0.40622343390981
S	-2.88109998682143	-2.75306001275520	-0.83217999009667
C	-3.84554999741056	2.01296000114600	-1.13627999953028
C	-4.31055000340362	2.71096999770645	-3.44319000234138
C	-2.67154000383579	2.39074999507352	-5.23569000010147
C	0.61768999950847	4.88527000085694	-4.26192000390145
H	-1.21028001310954	4.19184999650046	-3.38629001726274
H	2.54995999332918	5.31728999695503	-5.15903001565412

H	7.55444999796884	-0.33170000100598	-4.21452000270336
C	6.73899000203968	1.68050000171740	-4.14394999732337
H	5.72657000025428	3.55631999730926	-3.85420999935545
H	3.97710000551442	3.93244000428898	-2.19814999456939
C	-5.44405000031407	1.73914000080745	2.87736000068716
C	-5.53092000070691	2.91962999880306	2.11955999766780
O	-2.04022000249252	-3.66405999608165	-1.60098000637884
O	-3.70741999599050	-3.31200000246788	0.24529999906872
C	-4.67609999805735	2.59114999724892	-2.08377999825235
C	-4.30456000191684	1.91858000084629	0.26873999929501
C	-5.22395999846018	3.20724999634137	-4.41041000108588
C	-3.58403000273573	2.87012999940505	-6.15233999873949
H	-1.69207000005767	2.05302000195977	-5.57091000136329
H	0.30695000728427	5.93097000209414	-4.24617998893146
H	7.32713999570165	2.03224000011498	-4.99271000359242
C	-5.81417000208479	0.32660999862181	4.95941999836278
C	-4.86932000096839	3.28845999826066	-5.73939999839288
C	-7.66298000014965	1.72068999927214	4.01486999825425
C	-4.74098999803586	0.66816999956427	2.31311000129402
H	-5.67264000178857	2.91600999836576	-1.78023999853085

102

3b_5 (cat)

H	0.83586000199111	-5.83991000173270	1.29773999825967
H	-5.37322994287511	2.05756004166397	-0.22900995998877
H	-3.88429807313255	3.57301712250496	-3.97762351136187
H	5.88614000217293	-7.30862000163624	-1.47566000174185
H	-5.28610982287517	3.82625000336274	-1.90383987359691
C	-4.77977005220027	1.89882986279005	-1.13001002319170
H	1.17062999761299	-3.39937000111421	1.78822000138320
C	-3.26419993270446	0.57659002294265	-2.43855985262479
C	-4.72110035791889	2.91275007683380	-2.08504034270111
C	-3.93565000013086	2.78636983055249	-3.23810983445819
C	-3.21494963837623	1.59119031104259	-3.38690002546308
C	0.21410999890661	-3.80057999970630	1.49078000230246
C	0.00385999856955	-5.13986000173042	1.19958000231787
C	-1.25098000056445	-5.63645000131689	0.77498000060805
C	-1.41871999965619	-7.00242999899889	0.42255000112582
C	-2.62643000004625	-7.47151000168032	-0.04642000156477
C	-3.72117000118377	-6.58703999831845	-0.18108000090080
C	-3.59717000151994	-5.25799999880271	0.16882999848584
C	-2.37045999911029	-4.74485000156596	0.66549999914012
C	-2.18320000233527	-3.36683000008334	1.00670000053912
C	-0.91758000184597	-2.94597000042494	1.37970999798610
O	-0.75089999766151	-1.58188000162358	1.64662999839576
P	-0.68401999951542	-0.59113000011628	0.36668000130997
N	-0.93474000213369	0.90381000170383	0.82917000086083
S	-0.65496000032577	1.46620000130045	2.31421999809618
O	-0.73286999907488	2.92170999843228	2.24804000102969
O	0.51072000429065	0.85800000161618	2.95701000327464
C	4.39843000031299	2.01494999821511	0.37809999911903
H	3.75503998993512	0.62660999727028	1.91116999755028
C	-4.68084000163272	-3.47850999769608	2.63580999977241
C	-6.92161999835352	-2.65479999979451	3.06965000169450
C	-5.85993999942922	-3.54590000103770	3.34896999959264
O	3.13487000232815	-0.46195999712740	-0.21738000208826
H	2.70194998288446	2.31522999616784	3.31195999317896
C	-4.05547001039803	0.70639999183300	-1.28257000196520
O	-1.88954000030223	-1.10326999861255	-0.58293999862532

C	-4.18919999482345	-0.37805999655889	-0.28381999944254
C	-5.56817000158387	-1.58425999806769	1.35374999658186
C	-5.39176999366615	-0.57661998059710	0.37927001685896
C	-4.50320000196136	-2.50726999799002	1.61734000246296
C	-3.13795000768321	-1.28839001232081	0.02786998188915
C	-6.77716000215415	-1.69605000032870	2.09030999731665
C	-3.28483999840106	-2.37625000179737	0.87928999846716
C	3.18859002364302	2.63857000646110	2.39446001564561
N	0.59804000169409	-0.97145999998225	-0.49359999946581
P	1.81167999832609	-0.24308000105436	-1.10150000163750
N	1.75215999690242	1.41528999942079	-1.30400000169529
S	0.95366999659216	2.16173999888188	-2.59887999863074
O	-0.13264993558475	1.26476999773340	-2.93607996869054
O	1.94282999593467	2.56891999846654	-3.57843000001135
H	0.47236999715381	3.25080000123333	-1.95860999825754
C	4.37342998588472	3.37069999761118	-0.00672000884123
H	9.43319000042390	0.09568000183181	-4.23744000053795
H	6.01408000134374	-2.48043999915697	-3.83953999985798
C	7.76275000206312	-1.28032999944313	-4.14386000142932
H	3.75809001549303	5.36763000358368	0.45897000547245
H	2.67994024496636	4.73165080521294	2.63937574818293
H	6.79534000182581	2.16644000217923	-0.87297999954908
H	-1.73750000158868	1.04910999771770	3.01213000009248
C	6.57636000101762	-1.60423000222687	-3.51933999885550
C	3.78359001526082	1.66780000434285	1.59158000888194
H	8.13314000061743	-1.90620000186418	-4.95699000074403
C	3.16945000323125	3.99032000227239	2.02519000041117
C	6.06735000071266	-0.80503000024401	-2.46268000237146
H	4.81604000890548	3.66402000192264	-0.96133999438796
H	8.56146000181571	1.57172000044349	-2.44752000146187
C	3.76858997309596	4.33097999418017	0.80008998432056
H	2.56957999996751	1.93409000045665	-0.94894000190535
O	2.20455000045016	-0.86058000190790	-2.53797999825619
C	2.71400999773217	-2.18009000134813	-2.46265999957702
C	4.04334000198609	-2.32257000161104	-2.08783999910943
C	4.57381000061825	-3.64340000194787	-1.93365000043352
C	5.88490000233068	-3.89940999796678	-1.45339999835645
C	6.34146000041514	-5.19102999842953	-1.29079000190768
C	5.51136000238131	-6.29221999765528	-1.60361000230826
C	4.22883999770362	-6.07785999979019	-2.05821000201344
C	3.72150000128905	-4.76054000022870	-2.22047000066550
C	2.38508000173380	-4.53627000034442	-2.62187000066987
C	1.83752999986839	-3.26588999888858	-2.73055999890294
H	0.80878000088866	-3.08367999987636	-2.99968000062844
C	8.49931999751837	-0.14132999753345	-3.74512000081326
C	6.78779999947561	0.38108000214141	-2.09557999839303
H	7.35029999888117	-5.36348999893050	-0.91294000177863
C	4.36894000204742	-0.19738999880847	-0.85309000226602
H	6.53259000254795	-3.06034999791791	-1.20374999806107
H	1.75479999998052	-5.39903000048909	-2.84527999786181
C	5.02715999888077	1.01551999806292	-0.51570000166629
H	-4.67200000122265	-6.95709000051260	-0.56760999961575
H	-3.86202999939738	-4.15828000088855	2.86885000054922
H	-7.84941999996057	-2.71999000156706	3.63982999793980
H	-7.58139999935868	-0.98772999822620	1.88250999721229
H	-0.56222999770798	-7.67307999804953	0.52027000218719
H	-2.73901000101906	-8.52143000033674	-0.32035999774536
H	-5.96823999920087	-4.28470999826281	4.14412000206794

H	3.57260999824363	-6.91953999856241	-2.28938000186199
H	-4.44671000164095	-4.58554999822779	0.05819999949423
H	-6.23703000164197	0.07337999644610	0.14973999789247
C	8.01576000242630	0.67436999853395	-2.74621999830770
H	-2.57613023865033	1.44568989159824	-4.25899006155222
H	-2.67631006186975	-0.32323003543018	-2.60376005128241
C	6.24496000201728	1.25895000152772	-1.12898999998908
C	4.84322000145764	-1.10947999900377	-1.78278999835789

102

3b_5 (TS1-1)

C	-2.30038003909945	-1.38534998290798	3.20475006105602
H	-1.92425997693424	-0.36431001262571	3.19272996773912
C	-1.62777007881329	-2.33688996784289	3.96356010609144
C	-5.66110999824971	-0.81379999841564	1.74995000532266
H	-0.73252994100054	-2.02174000680986	4.50257993947881
C	-2.07555000250054	-3.66527000346298	4.05712000138578
C	-3.46065999854893	-1.72403999909031	2.48250999816625
C	-8.66787999906861	0.82162999795357	0.27332000127136
C	2.40810000039807	-0.73061002220613	-3.40031985927539
H	-4.74491002357614	-3.37566999244169	1.94065003101619
H	3.59820999872923	8.28041000182106	-1.10446999839091
H	-6.14426999852396	-1.63621000035721	2.27916000383627
H	-8.33051000215680	-0.91681000181817	1.49252000009716
H	-3.55657999654591	-1.40957000096270	-2.28320000196701
H	-6.14034000050080	5.63291999773208	2.60680999826298
H	-1.55175310714614	-4.39561533855804	4.65706954641806
H	-6.25611999950415	2.95037999949150	-0.86923000134514
C	3.62341999488128	-0.95937000814619	-2.72524998408568
C	1.85374964964304	-1.70217028496727	-4.22476982559500
H	1.88125003660406	0.21391003467559	-3.28267007352521
H	-9.74660000165055	0.67027000219965	0.21374999935584
C	-7.88411000014670	-0.05689000010364	0.98959999889637
H	-8.68886999829564	2.58382000039482	-0.99729000199701
C	-3.87696995882485	-3.06368001232833	2.52458994058857
C	4.26686999962083	0.06792000502700	-1.87476000540429
C	4.25038007528287	-2.19872985841675	-2.92941008364877
C	2.48169009205318	-2.94064984259474	-4.44013988296142
H	0.89258019860860	-1.48704991121966	-4.69425013926681
H	-1.17135999948041	3.39907000231772	-2.15176000152603
C	-3.20461991751937	-4.00820003271992	3.30097988978155
H	0.50307000273053	3.32400000121156	2.32591999999030
C	3.51922999806156	0.99816001602178	-1.09714001990557
C	5.64694999915466	0.19578998176375	-1.83469998112159
C	3.69720014448772	-3.15939006698109	-3.77735043091132
H	5.17652995709835	-2.42873004305927	-2.40066993301453
H	2.03878538760045	-3.69262603385015	-5.07672883578432
C	-1.97638999882502	3.58384000182310	-1.45720999984223
O	2.12787000050777	0.87161999812686	-1.11042999981395
C	4.08384999908808	2.05128999909574	-0.38811000099770
H	6.26096000276896	-0.46878999643329	-2.44407000066184
C	6.29437999798287	1.15960999724077	-1.02880000055388
H	4.22565990449568	-4.10435000849554	-3.90119981140453
H	1.90729000106423	7.52868000005207	0.55178000199399
H	-5.63992999760364	3.34840999784403	1.84993999776725
H	3.57183999903492	-1.03165999821301	2.14164000072617
C	-2.72058999891523	2.49514999853255	-0.92381000004152
C	-2.35519999993298	4.85129999825816	-1.04403999829374
P	1.34918000245966	0.46057999809840	0.24367999822968

C	5.51179000115979	2.10443000130927	-0.28544999782681
C	3.21600000170900	3.10087000105409	0.21078999961030
C	7.71064999746297	1.21148999886966	-0.93754000143573
H	0.81228999922298	5.75778000039433	1.79834000220911
C	3.52309000261036	7.22638000021499	-0.83378000212611
H	5.11046000084644	6.60638999795871	-2.18134999887076
C	-6.70833999829420	2.11695000046662	-0.33343999881705
H	-4.92811000115835	7.58796000255301	1.62782999860411
C	-5.38305000252297	5.47229000243513	1.83791000064025
O	-2.11086999758465	-3.16802999815921	-2.31304999765163
C	-8.07057999900030	1.90945000191027	-0.40339000253678
O	-1.61410005105122	-0.93409998867037	-3.32872996850547
O	-2.37449999724166	1.20710000208932	-1.33767000126057
C	-3.77314999892876	2.62553999799314	-0.02941999866989
H	-1.83197000242137	5.71948000069633	-1.44956999919171
C	-3.39687999998521	5.06336000140054	-0.11094000123833
O	2.02469000222631	1.39223000231654	1.37153999941887
N	-0.12938999736891	0.81610000124639	-0.05476999933662
N	1.76150999897292	-1.05275000101968	0.70704000230158
C	6.18380000165975	3.02938000241049	0.55404999764595
C	3.35936000262591	4.48337999761426	-0.14625000135143
C	2.18572000243007	2.75396999926552	1.06981999882927
H	8.29611000166467	0.49057000061201	-1.51126000186401
C	8.33376000222969	2.13120000209157	-0.12208999832757
P	-1.57373000050091	0.24779000087525	-0.30652999880075
C	-4.45638000043462	1.41406999667497	0.49964999655842
C	-4.11789000006528	3.94234999793866	0.42238999940537
C	-3.72143000174667	6.37258000182296	0.33542000009527
S	2.29906999714359	-1.48318000191416	2.19977000230844
H	1.47055000202582	-2.11963000263237	-0.08184999806496
C	7.56086000166600	3.03926000002115	0.63711999784784
H	5.59701000063400	3.72516999858519	1.15221000099467
C	4.30342000041214	4.94440999792508	-1.10059999833978
C	2.46772999869044	5.44096999840339	0.44422999984963
C	1.28290000120282	3.67492999890901	1.66780999845304
H	9.42217999765364	2.15272999963113	-0.05157000144755
O	-2.31995999914161	0.52586999750968	1.10271000079907
N	-1.78639000054010	-1.25866000198307	-0.78175000303995
C	-3.71250999991800	0.41012999764607	1.10769999634979
C	-5.87562999773339	1.24212000236394	0.41041999999166
C	-5.10352000044425	4.18926999948551	1.41392000259727
H	-3.17110999972489	7.21579000005100	-0.08760000039043
C	-4.69395000192446	6.57773999989903	1.28929999761685
O	2.29107999995368	-2.93934999851239	2.20417999995765
O	1.62491998456461	-0.76016000937960	3.26643001043390
H	8.05581999781759	3.74571000157987	1.30456000248256
H	4.96773999765656	4.22799000162243	-1.58013000209719
C	4.38191999958691	6.28016000146636	-1.43759999993054
C	2.58685000026422	6.81094999903334	0.08738000022291
C	1.46585999938108	5.01005000128826	1.34493000043572
H	-3.58359004398266	-5.03001998427652	3.31527005597662
S	-2.23490999686799	-1.70811999824783	-2.25539999926971
C	-4.27802999929056	-0.71730000208014	1.76718000030140
C	-6.47621000231993	0.11399000007612	1.06204000006556

102
3b_5 (TS1-1’)

C	-5.66539998403837	-1.57104000107526	2.56929001574639
C	-5.78231993878081	-2.92722999326225	2.85674005119866

H	-8.19273000026991	2.51891999869551	0.81793000282040
H	-6.20283001468927	-0.84316999855710	3.18028999203320
C	-5.98048000078547	1.03598999846221	1.14253999179143
H	-3.22308000071564	5.93598000108699	4.20981000239055
H	-6.41835002945628	-3.22961000325919	3.69091997484448
C	-4.28968006714962	-3.44938000443398	1.06069994512769
C	4.08322002401749	-1.92355986208637	-3.26974995132675
H	-3.97601999825399	4.99293000135961	-0.14453000156711
H	-6.14995999735176	6.10532000013109	-0.42639999713222
C	-5.09409999756750	-3.90112999880126	2.11275000282321
H	-3.73734997037565	-4.15380999617539	0.43924002682939
C	-4.15702001359838	-2.09055000186422	0.77818998850285
H	-5.19009449754324	-4.95313717178858	2.34190539010608
H	-3.51877204999413	-0.14658964344874	-2.73658319877957
H	3.82546000248152	7.42685000095319	2.25086000260307
C	3.48302999294826	-0.76144999805885	-2.75979999806834
C	3.47795003206833	-2.66680010369846	-4.28400036919297
H	5.03823998893384	-2.25957003951464	-2.86221998560231
C	-4.84004999530796	-1.12204000002348	1.52262999650114
H	4.00019029836059	-2.26207819407834	1.36258857522450
H	-3.51263998917773	-1.79052000029922	-0.04494998994160
C	-7.31501000141840	4.38749000219384	0.21215000005787
C	4.19490999518146	0.04850999456861	-1.74699001195855
C	2.23232009853242	-0.39329004138150	-3.28749989707887
C	2.24662011313312	-2.28414985653473	-4.83324981278260
H	3.98698995625719	-3.55979998481869	-4.64606987136631
H	0.47428919296285	3.35562548645349	-1.44417233068518
C	-6.11285000125623	5.07650000027230	-0.06595000208989
H	-6.91792000160632	0.50730999809716	1.32330000160877
C	3.52787999629325	0.71427001392480	-0.68197000890926
C	5.57281000382404	0.18945998318790	-1.79557999038424
C	1.63721969997422	-1.13704022065762	-4.29921007601719
H	1.72657997678336	0.50055007637771	-2.93366001398431
H	1.77352556054365	-2.85662399156179	-5.61800790103799
C	-0.19587997908274	3.64597991560914	-0.64962009973306
O	2.13007000429626	0.62617999808191	-0.63817999544073
C	4.16705999988054	1.45026999744131	0.30793000301928
H	6.12395000102676	-0.25828999522823	-2.62352000279127
C	6.29849000179940	0.87567999909674	-0.79580000025072
H	0.66402014037842	-0.80757993693178	-4.66437002283287
H	-3.75587999725162	4.02841999653392	2.74363000017556
H	1.14972000463250	3.94343000234960	4.03790998964682
O	-1.45410002606133	-0.12194999092962	-3.63944998772978
C	-1.31615006789702	2.82643009654359	-0.32581992830473
C	-0.00143994332635	4.76507997606975	0.14459015953260
P	1.50030000115223	-0.35086000374819	0.48106999841914
C	5.60015000036737	1.48906000080369	0.29724999889227
C	3.36525000455234	2.16518001029024	1.33682999947437
C	7.71678999856592	0.93779000072392	-0.83341000205973
H	-1.21062000015963	7.34926000340129	3.76813000113085
C	-4.89193000378214	4.45440000163649	0.09311000210243
C	-2.57434001894172	5.70547002133142	3.36354998393184
C	3.72570000173772	6.35731000045657	2.06018999841074
H	5.14048999780103	6.36127999951999	0.41116000131804
H	2.25710999918725	6.04812000243679	3.60121000081442
C	-7.27250000065169	3.07392000055424	0.62583000145158
O	-2.70739999694666	-2.23646000015972	-3.14521999948149
H	-8.27339000040287	4.89252999913403	0.08353999983844

O	-1.47088993721488	1.62715994956649	-1.03258000335352
C	-2.28753005401581	3.16927004246697	0.60167986203428
H	0.83561996830972	5.43147002109387	-0.07041005281866
C	-0.86288000376101	5.08933000926188	1.21802997761174
O	2.23271001670509	0.11341000764293	1.83954999776106
N	-0.03199999704501	-0.10731999483853	0.49276999916874
N	2.03608998309177	-1.87564000927293	0.24308000471253
C	6.36228999742395	2.07526000232510	1.34020999873769
C	3.51360999710505	3.57588000137293	1.55943999732470
C	2.39355997584307	1.49062998106761	2.06565000882050
H	8.23546000164024	0.47306999917810	-1.67405999933135
C	8.42629999882953	1.54030999992843	0.18273999763869
P	-1.38528002258591	0.21390000736094	-0.24380001802925
C	-3.56438996347766	2.41321996685549	0.69319007384449
C	-2.03848997443753	4.30094997329613	1.44731003470176
C	-0.59654002670157	6.19775002519962	2.06472996384797
S	2.78232001044334	-2.84891999485885	1.35202999578502
H	1.32232000266512	-2.60768999695027	-0.64105000225842
C	7.74077000225544	2.09929999867079	1.28491999771089
H	5.84664999957139	2.49751000128251	2.20116000180669
C	4.37658999922178	4.39742999767443	0.78573000330103
C	2.71270001187447	4.20203999860725	2.57137999601493
C	1.54784001730705	2.09509001525200	3.03545998641555
H	9.51623000137439	1.57034000079540	0.14768000279226
O	-2.37639999176418	0.36349000484112	1.03169001447250
N	-1.89255000465648	-0.91141000088586	-1.24305999515601
C	-3.59185000476295	1.04368000244953	0.92565000365652
C	-4.81134999982736	3.10789000503379	0.53387999145813
C	-2.87588999197878	4.63825999384083	2.54261001385679
H	0.29611000481019	6.79473999703549	1.86909000397490
C	-1.43085998041029	6.50046997539439	3.11890002473341
O	2.85941999784419	-4.15071000100181	0.70470999784585
O	2.20164000003314	-2.71712000029961	2.67825000147126
H	8.30517999844906	2.54144000211878	2.10681000204914
H	4.95914000062074	3.94859999869354	-0.01711999889235
C	4.47647999743057	5.75228000226047	1.02625999843032
C	2.86108999341511	5.59410000362264	2.81325000066755
C	1.75867998130216	3.44213998693581	3.28378002191476
H	0.80207086459602	1.50584483769203	3.54636312145454
S	-2.36599999473393	-0.85883999873194	-2.76904000000971
C	-4.79429999939220	0.32097000154130	1.19003999406281
C	-6.03216999970047	2.40101999744962	0.78378999601219

102

3b_5 (TS1-2')

H	-5.88637229401531	1.67074488638763	3.86131467272458
H	-6.21966000171070	3.50848000278409	-4.07859999998796
H	-1.69207000015405	2.05302000161682	-5.57091000144963
C	-5.22395999791386	3.20724999763508	-4.41041000081289
C	-4.17642002314395	0.75687998841451	1.04052999876470
C	-4.67610000044060	2.59114999227056	-2.08377999937028
H	-6.04998003320304	3.79020999187400	2.52479999066198
H	-5.58127000016636	3.66349999939871	-6.47598000063525
C	1.60213001907348	-4.26886013048203	-0.07599993751862
H	0.30695000657286	5.93097000173792	-4.24617999039668
C	-4.30455999995089	1.91858000155315	0.26873999918272
H	-5.06286001067212	3.94466999464739	0.28761999259522
C	-4.74099004958745	0.66816998368431	2.31310995128649
H	-4.61831997404280	-0.26824999459925	2.85700001665273

H	-3.64413998405734	-0.10629999406148	0.64592000268377
H	-3.71706580767362	-2.16419807263155	-1.72215560583864
H	-3.31559999981894	2.91124000078166	-7.20879000139483
C	2.70251999274219	-4.14101997097730	0.78669998040642
C	0.53256004641327	-5.10448998742989	0.24001996298687
H	1.57603998409575	-3.72213987511281	-1.01547009380758
C	-4.86932000085134	3.28845999853955	-5.73939999835625
H	7.32713999682791	2.03224000061168	-4.99271000257559
C	-4.97855998313888	3.01027001224508	0.84631001407174
C	-3.58403000257004	2.87012999976540	-6.15233999864446
C	3.85530000294774	-3.25813000415555	0.50523999775944
C	2.67924997093710	-4.87874998864801	1.98253001855726
C	0.51043998165815	-5.85471000226428	1.42543000861230
H	-0.30363002312393	-5.14351999178359	-0.45731999574010
H	1.48046798692489	-1.16162403110537	-3.48029817452967
H	-5.67264000128468	2.91600999951612	-1.78023999827187
H	2.92284462616987	0.31855055195509	3.40232018334851
C	3.69309000254387	-1.95344999995881	-0.03118999749466
C	5.14636999810478	-3.62619000331017	0.84129999857718
C	1.61192000130221	-5.71942995067124	2.28695000515835
H	3.48955000773085	-4.75381000882787	2.70228999075186
H	-0.32562546409579	-6.49368194367327	1.67028160891133
C	1.14042008750593	-0.14311016053903	-3.58691959104874
O	2.39432999841473	-1.56215000474372	-0.39035999805477
C	4.71214999418186	-1.02993999881524	-0.19508999902423
H	5.32995000150376	-4.63145999950690	1.22499000222159
C	6.23094999747606	-2.72164999840655	0.76692999870499
H	1.63990000878808	-6.26807002575636	3.22903999708827
O	-2.04021999892773	-3.66405999906941	-1.60098000466864
C	6.73899000264535	1.68050000184436	-4.14394999664071
H	2.54995999480658	5.31728999724877	-5.15903001178077
C	-0.12971005384356	0.24904014128051	-3.07199027511932
C	1.89622987236218	0.86083007530356	-4.17628042537312
P	1.65707998849666	-0.56989997825854	0.63555000429933
C	6.01843999900970	-1.38760999956121	0.28180999800876
C	4.44576000545408	0.29102000124805	-0.82132000049318
C	7.52503000016649	-3.09441000199523	1.21731000004946
O	-3.70741999571798	-3.31200000262322	0.24529999915821
H	5.72657000083877	3.55631999738926	-3.85420999894786
C	-5.53091993438257	2.91963001567597	2.11956000408131
H	7.55444999809070	-0.33170000096892	-4.21452000266597
H	3.97710000111786	3.93244000101311	-2.19814999979798
C	-5.44405000614702	1.73914000056733	2.87736001898755
C	-2.67154000369859	2.39074999530443	-5.23568999989664
C	0.61768999999438	4.88527000012341	-4.26192000404876
H	-1.21028000536537	4.19184999569706	-3.38629000795999
O	-0.88409000702690	-0.70437006852605	-2.36140997135359
C	-0.67990000460640	1.51095994314088	-3.23581992330289
H	2.86617002704557	0.61102999217558	-4.60903989903807
C	1.46304000408115	2.20430997784607	-4.23727998237969
O	2.62033000244897	0.72451999307526	0.69832999649786
N	0.25154000200693	-0.27336001101755	0.05356999330416
N	1.56953000779289	-1.07841001831247	2.17240999396028
C	7.09578999799898	-0.46666000110998	0.34442999921890
C	5.23803999507482	0.74008999511386	-1.93346000420007
C	3.43599000777301	1.13158000774068	-0.36493998905502
H	7.67661999781770	-4.11592000052481	1.57216000048814
C	8.55843999815663	-2.18339999964436	1.23905000176361

P	-1.07004997952258	-0.46158000488960	-0.77125998548259
C	-2.09488998624549	1.78543002018228	-2.87338998423404
C	0.15453005271475	2.54927001460387	-3.76621990248691
C	2.30303004491358	3.22459000530573	-4.75722989882044
S	2.63693999739329	-1.00261999504521	3.43172000205479
H	0.41643999776411	-1.73468999821777	2.51310000254123
C	8.33318999908131	-0.85390999850166	0.81673000215715
H	6.93529000040934	0.56361000184808	0.03036999834264
C	6.14673000290146	-0.11036999716942	-2.61729999775505
C	5.07403000061901	2.08031999850700	-2.41292000163590
C	3.25253999039148	2.46694999818088	-0.82617001288014
H	9.54398999740194	-2.47934999990675	1.60123000144798
O	-1.69606999468432	1.03549000403478	-0.63841999740546
N	-2.07138003164078	-1.53578997581422	-0.18675001577775
C	-2.56671999725055	1.57135000657506	-1.58576000037938
C	-3.00096000031078	2.30276000159421	-3.85850000485503
C	-0.23469001834143	3.91317999807664	-3.78199004014970
H	3.29185999689141	2.94396000069014	-5.12549000890192
C	1.89158998745073	4.53924999368534	-4.76922002879445
O	3.86045999898599	-1.73785000033199	3.16024000160137
O	1.83602999793288	-1.33214999998978	4.60200000122719
H	9.14123000142327	-0.12309000004257	0.87167000062921
H	6.25754999988748	-1.14209000001293	-2.28635000234245
C	6.87300000150661	0.34579000033638	-3.69802000064034
C	5.85463999615135	2.52716999710358	-3.51195000393450
C	4.10729000564244	2.91918000518117	-1.81502998823742
H	2.46728488014687	3.07711564037095	-0.40612471218038
S	-2.88109998415075	-2.75306001488558	-0.83217998862635
C	-3.84554999789365	2.01296000042652	-1.13628000095253
C	-4.31055000392909	2.71096999617396	-3.44319000244954
